# Tianma Formula Alleviates Dementia via ACER2-Mediated Sphingolipid Signaling Pathway Involving A*β*

**DOI:** 10.1155/2021/6029237

**Published:** 2021-08-04

**Authors:** Haochang Lin, Sha Wu, Zhiying Weng, Hongyan Wang, Rui Shi, Menghua Tian, Youlan Wang, Haiyan He, Yuchuan Wang, Xuan Liu, Zhimin Jian, Fuqin Wei, Peng Wang, Liuyi Zhang, Yi Liu, Qiuzhe Guo, Chen Chen, Weimin Yang

**Affiliations:** ^1^School of Pharmaceutical Science and Yunnan Key Laboratory of Pharmacology for Natural Products, Kunming Medical University, Kunming 650500, China; ^2^Zhaotong Institute of Tianma, Zhaotong 657000, China; ^3^Kunming Institute of Medical Sciences, Kunming 650011, China; ^4^College of Agronomy and Life Sciences, Zhaotong University, Zhaotong 657000, China; ^5^Shanghai University of Traditional Chinese Medicine, Shanghai 201203, China; ^6^Department of Cardiovascular Surgery, The 1st Affiliated Hospital of Kunming Medical University, Kunmin 650032, China

## Abstract

**Objective:**

To reveal the molecular mechanism of the antagonistic effect of traditional Chinese medicine Tianma formula (TF) on dementia including vascular dementia (VaD) and Alzheimer's disease (AD) and to provide a scientific basis for the study of traditional Chinese medicine for prevention and treatment of dementia.

**Method:**

The TF was derived from the concerted application of traditional Chinese medicine. We detected the pharmacological effect of TF in VaD rats. The molecular mechanism of TF was examined by APP/PS1 mice in vivo, *Caenorhabditis elegans* (*C. elegans*) in vitro, ELISA, pathological assay via HE staining, and transcriptome. Based on RNA-seq analysis in VaD rats, the differentially expressed genes (DEGs) were identified and then verified by quantitative PCR (qPCR) and ELISA. The molecular mechanisms of TF on dementia were further confirmed by network pharmacology and molecular docking finally.

**Results:**

The Morris water maze showed that TF could improve the cognitive memory function of the VaD rats. The ELISA and histological analysis suggested that TF could protect the hippocampus via reducing tau and IL-6 levels and increasing SYN expression. Meanwhile, it could protect the neurological function by alleviating A*β* deposition in APP/PS1 mice and *C. elegans*. In the RNA-seq analysis, 3 sphingolipid metabolism pathway-related genes, ADORA3, FCER1G, and ACER2, and another 5 nerve-related genes in 45 key DEGs were identified, so it indicated that the protection mechanism of TF was mainly associated with the sphingolipid metabolism pathway. In the qPCR assay, compared with the model group, the mRNA expressions of the 8 genes mentioned above were upregulated, and these results were consistent with RNA-seq. The protein and mRNA levels of ACER2 were also upregulated. Also, the results of network pharmacology analysis and molecular docking were consistent with those of RNA-seq analysis.

**Conclusion:**

TF alleviates dementia by reducing A*β* deposition via the ACER2-mediated sphingolipid signaling pathway.

## 1. Introduction

Dementia is an acquired neurodegeneration in multiple cognitive domains severely affecting social or occupational function [1]. Advancing age is the main risk factor, and due to the aging of the world population and lack of effective treatments, the number of affected individuals is anticipated to triple by 2050 at a cost approaching $4 trillion [2]. Dementia is frequently linked to greater than 1 type of neuropathy, usually AD with VaD [1]. AD, the most common cause of dementia and accounting for about 60 to 80% of cases [3], is an age-associated, neurodegenerative disorder, histopathologically characterized by senile plaque (SP; composed mostly of A*β* peptide surrounded by dystrophic neurites) and intracellular neurofibrillary tangles (NFTs), composed of hyperphosphorylated tau protein levels, leading to loss of synapses in the selected regions of the brain and, eventually, neuronal death. Clinically, AD has many characteristics, the most significant of which is progressive cognitive decline, including memory loss and advanced executive function [4]. VaD, one of the most common causes of dementia after AD, causing around 15% of cases [5], is a kind of cerebrovascular disease, characterized by cerebral hemorrhage, ischemia, hypoxia, and other cerebrovascular factors, causing insufficient blood supply to the brain and further damage of brain tissues, mainly the hippocampus and cortex [2], resulting in learning and memory dysfunction of human beings. The clinical manifestations of the disease are similar to those of other dementia [6]. Despite the importance of the disease, diagnosis is hindered by the lack of clear diagnostic criteria and effective specific biomarkers. Thus, there is an urgent requirement to find specific and effective biomarkers to improve diagnosis and detect them as soon as possible, enhance the efficiency of treatment, discover pathological features, and monitor clinical trials [7]. At present, the strategy of clinical treatment of dementia is mainly symptomatic treatment, including cholinesterase inhibitors (galantamine, donepezil, and carbamate), N-methyl-D-aspartate antagonist memantine, and some traditional Chinese medicine [2, 8–11]. However, the chemical drugs are single-target therapy, having great individual differences after administration, and only effective for some patients. Consequently, researching and developing traditional Chinese medicine is necessary for dementia treatment due to its multichannel, multilink, multitarget, and less toxicity.

TF is composed of Tianma (*Gastrodia elata*), *Panax notoginseng* (PN), Acorus tatarinowii (AT), Coleus forskohlii (Col), and peanut shell extract (PS, mainly luteolin). Previous studies have shown that Tianma has an important neuroprotective effect on cerebral ischemia-reperfusion injury [12, 13] and exhibits the effects of brain protection, antiplatelet, and antineuroinflammation [14]. PN exerts targeted therapeutic effects of A*β*, while being used in the treatment of neurological diseases in the clinic [15, 16]. The main active ingredient of Col is forskolin, which has been shown to induce hyperphosphorylation of tau protein in hippocampal neurons [17] and plays a crucial role in the recovery of memory function and nerve growth [18–20]. New lignans attenuate the cognitive deterioration of A*β* transgenic flies discovered in AT [21], as well as AT can improve the neurotoxicity of A*β* protein to the body [22, 23]. Luteolin has great potential in the treatment of nerve-related diseases [24], which can improve cognitive impairment [25] and protect the hippocampus from nerve injury by inhibiting nerve inflammation [26].

The rapid development of network pharmacology and bioinformatics technology provides an excellent theoretical basis for elucidating the action mechanism of traditional Chinese medicine formulations. For example, RNA-seq is used to analyze the effects of drugs and diseases on gene structure and expression, to find the targets, pathway, functional regulation, and even pathological processes of diseases and drugs. Network pharmacology explains the regulatory mechanism of multipathway and multitarget treatment of diseases from the micro- to macrolevel. Therefore, we better account for the theoretical basis and medication guidance of traditional Chinese medicine formula for dementia.

In this study, we investigated whether TF alleviated VaD and AD by analyzing the histopathological changes to obtain the optimal dose of TF. Also, the molecular mechanism of TF for VaD was revealed through using RNA-Seq, as well as supported by network pharmacology. In the end, experiments were adopted to verify conclusions from the abovementioned analysis. Accordingly, a firm foundation was provided for the development of TF on dementia.

## 2. Materials and Methods

### 2.1. Animals

APP (PS1) B6 transgenic AD mice were purchased from the Nanjing Biomedical Research Institute of Nanjing University. CL4176 (SMG-1TS [pAF29(MYO-3/A1-42/let UTR) + pRF4 (ROL-6 (SUL0069))]) *Caenorhabditis elegans* (*C. elegans*) and their food bacteria OP50 were obtained from the Kunming Institute of Botany, Chinese Academy of Sciences. Male Sprague Dawley rats (265 ± 15 g, 16 weeks old) were purchased from the Laboratory Animal Services Centre of Kunming Medical University. All animal experiments were conducted under the authority of a license issued by the Government of Yunnan Province and approval from the Animal Experimentation Ethics Committee, Kunming Medical University (approval license number: SCXK K2015-002).

### 2.2. Reagents

Tianma was purchased from Zhaotong Xiaocaoba town in Yunnan Province; PN was purchased from Wenshan in Yunnan Province; and Col was purchased from India. Isoflurane was obtained from RWD company (217170701, China); topical chloramphenicol eye drops was obtained from Meidakanghuakang company (17022828, China); and preparation of medium AGAR peptone and cholesterol reagents were from Sigma Company. GAPDH primer was provided by Sangon Biotech company (B661204, China); the PrimeScript RT kit was from Takara company (RR820A, RR047A, Japan); Synaptophysin (SYN) and the Tau ELISA kit were from Signalway Antibody company (134370105); TNF-*α* and the IL6 ELISA kit were from Abclonal company (9670027500 and 9670026100); and the ACER2 ELISA kit was from mlbio Company (37357A, Shanghai, China).

### 2.3. Preparation of Traditional Chinese Medicine and Drug Administration

Tianma, PN, AT, Col, and PS extract were extracted by ethanol.

VaD rats were divided into 5 groups: sham, VaD, VaD + donepezil (1 mg/kg, i.g.), VaD + TF-low dose (TF-low-dose group, 0.18 g/kg, i.g.), and VaD + TF-high dose (TF-high-dose group, 0.746 g/kg, i.g.).

APP/PS1 B6 mice were divided into 3 groups: sham, AD, and AD + TF-high dose (TF).

*C. elegans* were divided into 2 groups: control and TF.

Drug administration was carried out four weeks after surgery through the intragastrical administration in rats (the sham and model groups were given sterile water). APP/PS1 transgenic AD mice were fed with TF for 8 months.

### 2.4. 2-VO Surgery

Animals received either bilateral common carotid artery occlusion (cerebral hypoperfusion; 2-VO) or sham operation under isoflurane (5% induction; 2% maintenance) anesthesia, as previously described [27]. The rats were placed on a heating pad until they recovered from anesthesia at 37°C after surgery. A midline neck incision was made, and both the left and right common carotid arteries were carefully isolated from the surrounding muscle and adjacent nerve bundles using aseptic surgical techniques. Each artery was doubly ligated (2-VO) with 4–0 silk sutures to prevent blood flow. The sham surgery included similar procedures, but no ligation. Animals received topical chloramphenicol eye drops on the midline neck incision before intraperitoneal injection of sodium nitroprusside solution for injection.

### 2.5. Morris Water Maze

The hippocampus-dependent neurocognitive function of all rats and mice was evaluated using the Morris water maze test, as previously described [28]. For the collection of descriptive data, the pool was divided into 4 quadrants by the analytical software (Ethovision 2.0, Noldus, Wagenigen, the Netherlands). The hidden platform was placed in the first quadrant. The rats were trained continuously for 5 days, 4 times a day, 2 times in the morning, and 2 times in the afternoon, and the order of quadrants per day was different from that of the previous day. The escape latency (time to reach the platform) was generally used to assess the learning and memory of the rats. The specified time is 90 s (mice are 60 s). If they did not find a platform within the 90 s, we would help them find the platform and stay on it for 10 seconds. That was adopted to evaluate the learning and memory of rats.

After the test of gaining the hidden platform, the retention of spatial memory was assessed in the spatial probe test. After the end of the place navigation test, the hidden platform was removed and all rats were placed into the pool facing the wall of the pool from the third quadrant (opposite quadrant of the first quadrant). Each rat swam for 90 s. The swimming distance, the distance of the first quadrant of space exploration, time of the first quadrant of space exploration, the distance of the rats of the target quadrant, and time spend of the rats of the target quadrant served as the detection indexes of this experiment.

The hippocampus-dependent spatial learning and memory of all mice were evaluated using the Morris water maze test, as previously described.

### 2.6. A*β*-Induced *C. elegans* Paralysis Assay

The CL4176 *C. elegans* was resuscitated, and the *C. elegans* was placed in a culture plate containing fresh OP50 and cultured in an incubator at 16°C. After the *C. elegans* oviposition, the newborn *C. elegans* were synchronized to make them in the L1 phase. When the larvae were cultured at 16°C for about 48 hours, the larvae developed to the L3 stage and were immediately incubated at 25°C. 20 hours after heating, the paralysis behavior was observed and recorded, and the time and number of paralysis were observed and recorded every 2 hours. If the *C. elegans* could only move the head but not the torso, it means the *C. elegans* was paralyzed. The *C. elegans* were repeated more than 3 times at each drug concentration, and the total number of them was more than 100. Statistics of the number of *C. elegans* and the total number of them paralyzed on each plate were analyzed.

### 2.7. RNA-Seq

RNA-seq transcriptome library was prepared using the TruSeq^TM^ RNA sample preparation Kit from Illumina (San Diego, CA) using 5 *μ*g of total RNA. After quantified by TBS380, paired-end RNA-seq sequencing library was sequenced with the Illumina HiSeq 4000 (2 × 150 bp read length). Then, clean reads were separately aligned to the reference genome with an orientation mode using TopHat (http://tophat.cbcb.umd.edu/,version2.0.0) software. The mapping criterion of bowtie was as follows: sequencing reads should be uniquely matched to the genome allowing up to 2 mismatches, without insertions or deletions. Reference genome (GCol_000001895.5, https://www.ncbi.nlm.nih.gov/assembly/GCol_000001895.5/) and gene model annotation (https://www.ncbi.nlm.nih.gov/assembly/GCol_000001895.5) files were downloaded from the NCBI database directly. RSEM (http://deweylab.biostat.wisc.edu/rsem/) [29] was used to quantify gene abundances. The samples with large deviation values A1, A2, B4, C3, and C5 were eliminated through Principal Component Analysis (PCA). Genes with Fold Change >2 and *P* value <0.05 found by DESeq2 [30] were assigned as differentially expressed. Additionally, functional enrichment analyses including GO and KEGG were performed to identify which DEGs were significantly enriched in GO terms and metabolic pathways at Bonferroni-corrected *P* value ≤0.05 compared with the whole transcriptome background. GO functional enrichment and KEGG pathway analysis were carried out by Goatools [31] (https://github.com/tanghaibao/Goatools) and KOBAS (http://kobas.cbi.pku.edu.cn/home.do) [32], and *P* value ≤0.05 was used as a cutoff. The RNA-seq raw data could be obtained from the Sequence Read Archive (SRA) database (https://www.ncbi.nlm.nih.gov/sra/?term=,SUB8718091).

### 2.8. Histopathological Analysis

Histological staining was performed using previously described methods [33]. In hippocampal tissues, sections were cut in the sagittal plane at a thickness of 3 um and mounted on slides. Sections were deparaffinized and rehydrated. Sections were stained with hematoxylin-eosin (HE) for morphological analysis. Digital images were obtained using a Nikon microscope (Japan).

### 2.9. qPCR with Reverse Transcription

Total RNA was isolated from the cerebral cortex samples in three groups and reverse-transcribed using a PrimeScript RT kit (Takara). All qPCRs were run on the Applied Biosystems 7500 Real-Time PCR system. Gapdh was used as an internal control to normalize the data across different samples. Primers used for qPCR were as follows: ACER2 forward: 5′-GGTGGATTGGTGCGAGGACAAC-3′; ACER2 reverse: 5′GTGGCGTACTGGCGGAACAAG-3′; PDK4 forward: 5′-TCGAGGCCACCGTCGTCTTG-3′; PDK4 reverse: 5′-GCGTTGGAGCAGTGGAGTATGTG-3′; MAFF forward: 5′-GCGAGCTGAGCG AGAACACG-3′; MAFF reverse: 5′-GTCACCTCCTCCGCCGACAG-3′; Diaph3 forward: 5′-CGGCAGAGTCTCAGTCCAATGTC-3′; Diaph3 reverse: 5′-TGGCGACTGGAGTCCTGGTG-3′; CDCA7 forward: 5′-CACAGAGGAGGAGGAGGACGAAG-3′; CDCA7 reverse: 5′-GCGCCGACTTCTGG ACACATC-3′; DIAPH3 forward: 5′-ACGAGGTAGTGACGCTGTGGTAC-3′; DIAPH3 reverse: 5′-CTT CTTGGTCGCCAGCTCTGC-3′; FCER1G forward: 5′-ATCCCAGCGGTGATCTTGTTCTTG-3′; FCER1G reverse: 5′-TCGACAGTAGAGCAGGGTAAGGAC-3′; ADORA3 forward: 5′-TTGCCGTCAGCCTGGAGGTC-3′; and ADORA3 reverse: 5′-TGACTCGCAGGTATCGGTCTACAG-3′.

### 2.10. ELISA Assay

ELISA was used to detect the concentration of TNF-*α* and IL-6 in serum, tau, and SYN in the hippocampus, and ACER2 levels in the cerebral cortex of VaD rats were detected by using the ELISA kit. The specific operations were carried out according to the instructions of each kit, taking ACER2 as an example. The cerebral cortex tissues were lysed in lysis buffer containing PBS, phosphatase, and protease inhibitors with ultrasonic crushing. After centrifugation at 5,000 g for 10 min, the supernatant and ACER2 standard were dispensed into the wells of an ELISA plate. Then, we dispensed 100 uL of an HRP-conjugated antibody solution into the wells, sealed the wells, and incubated the plate at 37°C for 60 min. Next, we discarded the solution in the wells and washed the wells with wash solution 4 times. Finally, we added 50 ul substrate A and 50 ul substrate B to each well. We gently mixed and incubated for 15 minutes at 37°C before adding 50 *μ*l stop solution to each well. We then read the Optical Density (O.D.) of each well at 450 nm (Multiskan GO, Thermo, Waltham, MA, USA).

### 2.11. Network Pharmacology

#### 2.11.1. Target Prediction

In this study, the related components and targets of PN and AT were obtained from the TCMSP database (http://tcmspw.com/tcmsp.php), and the screening conditions were set as oral bioavailability (OB) ≥30% and drug-likeliness (DL) ≥0.18. The Gene Symbol was acquired from the UniProt database (https://www.uniprot.org). The related targets (score ≥ 10) of each component of Col and Tianma were acquired from the Batman-TCM database [34] (http://bionet.ncpsb.org/batman-tcm/), The related structures of PS, which were acquired by literature searching (Table S1), were obtained from the Pubchem database [35] (https://pubchem.ncbi.nlm.nih.gov), and the related targets (probability ≥ 0.5) were gained from SwissTargetPrediction [36] (http://www.swisstargetprediction.ch/). VaD disease-related genes were obtained from the Genecards database [37] (https://www.genecards.org) by searching the keyword “Dementia, Vascular”.

#### 2.11.2. GO and KEGG Functional Enrichment Analysis

The common targets of TF and VaD were analyzed by the DAVID database [38] for functional enrichment of GO and KEGG.

### 2.12. Molecular Docking

The crystal structures of key targets were obtained from the RCSB protein database (PDB) [39]. Then, according to the best effective resolution, the crystal structure of each protein is selected. The 3D structures of active compounds of TF were obtained from PubChem and ChemSpider [40] databases. In addition, Pymol (version: 1.8.2.2) [41] was adopted for the preprocessing of the structure file and the visualization of the docking results, and AutoDockTool 1.5.6 and Autodock Vina [42] software were used for docking.

### 2.13. Statistical Analysis

All data values are reported as mean ± S.E.M. (standard error of the mean). Statistical analysis was performed using SigmaStat 3.5. The escape latency was analyzed by two-way analysis of variance (ANOVA), a *t*-test was used to compare the two groups, and one-way ANOVA followed Dunn's, Fisher-LSD, and Student–Newman–Keuls (SNK) method were used for multiple comparisons. Differences were considered statistically significant at *P* < 0.05.

## 3. Results

### 3.1. TF Enhances Spatial Cognitive Function in VaD Rats

**Figure 1:**
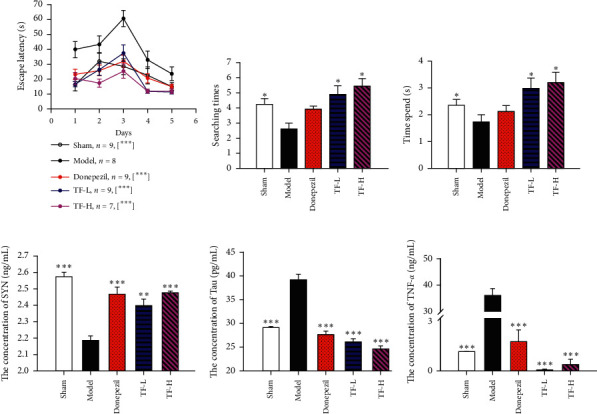
Effect of TF in VaD rats. (a) Escape latency; (b) searching times of the platform quadrant; (c) time spent (d) protein levels of SYN were detected via ELISA; (e) Tau, and (f) TNF-*α*; the escape latency was analyzed by the two-way ANOVA-Tukey method, the searching times of the platform quadrant were analyzed by one-way ANOVA-Dunn's method, and the time spent and protein levels of SYN, Tau, and TNF-*α* were analyzed by the one-way ANOVA-SNK method; ^*∗*^*P* < 0.05, ^*∗∗*^*P* < 0.01, and ^*∗∗∗*^*P* < 0.001 vs. model group.

We first established the VaD rat model and investigated the ability of spatial learning and memory of 5 groups of rats via the Morris water maze. Strikingly, compared with the sham group, the escape latency (*P* < 0.001, Figure 1(a)), searching times (*P* < 0.05, Figure 1(b)), and spend time (*P* < 0.05, Figure 1(c)) of the platform quadrant in the model group (Figures 1(a)–1(f)) were significantly altered; thus, the model was established successfully. By contrast, the escape latency (Figure 1(a)) was markedly reduced in the TF-low-dose group (TF-L, *P* < 0.001) and TF-high-dose group (TF-H, *P* < 0.001), while the searching times and spend time were raised in donepezil, TF-L, and TF-H groups (*P* < 0.05), indicating a dose-dependent therapeutic effect of TF in VaD. In addition, the content of SYN, as an important indicator for studying neuroplasticity and synaptic remodeling [43, 44], was remarkably increased, as well as tau and TNF-*α* were lessened in the sham, donepezil, TF-L, and TF-H groups (*P* < 0.001, Figure 1(e); *P* < 0.001, Figure 1(d)). To sum up, these data reveal that TF exerts a great effect on nerve growth and repair [45], tau [46], and neuroinflammatory [47] on the treatment of VaD and TF-H is optimal for VaD as well. Therefore, we used a dose of 0.746 g/kg/d (TF-H, referred to as “TF”) for the following experiments in this study.

### 3.2. TF Exerts a Positive Efficacy on Improving Nervous Function

**Figure 2:**
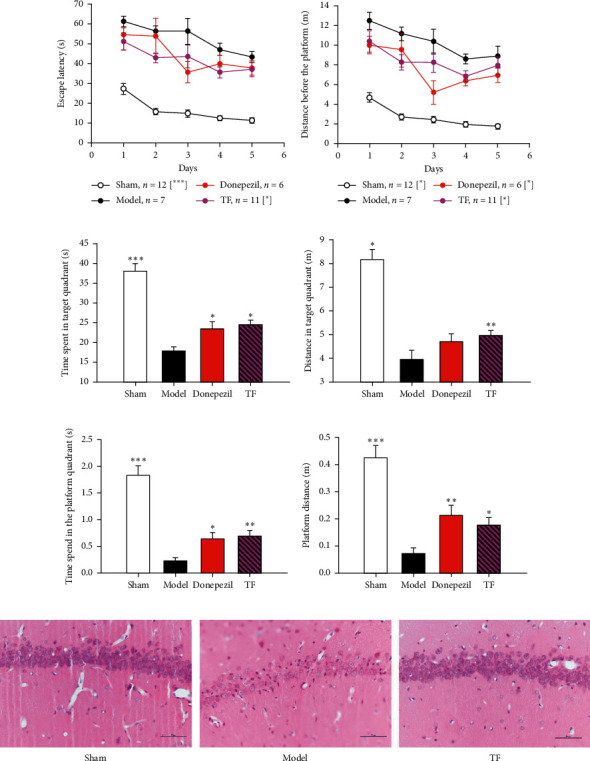
Behavorial and pathological reverification of TF in the VaD model. (a) escape latency; (b) distance before the platform; (c) time spent in the target quadrant; (d) distance in the target quadrant; (e) time spend in the platform quadrant; (f) platform distance; and (g) HE staining images of the hippocampus in each group. 400*x* under the lens; the escape latency was analyzed by two-way ANOVA-Fisher LSD, and the remaining data were statistically analyzed by the Fisher-LSD method in one-way ANOVA; ^*∗*^*P* < 0.05, ^*∗∗*^*P* < 0.01, and ^*∗∗∗*^*P* < 0.001 vs. model group.

Intending to revalidate the TF with better preventive and therapeutic effects on dementia, we established a VaD rat model once again. Strikingly, compared with the sham group, the escape latency, distance, distance of the first quadrant of space exploration, time of the first quadrant of space exploration, distance of the rats of the target quadrant, and time spent of the rats of the target quadrant of the model group (Figures 2(a)–2(f)) were significantly altered (*P* < 0.001). Notably, compared with the model group, significantly, the escape latency and distance (Figures 2(a) and 2(b)) were reduced, as well as the time spend and distance in the target quadrant and the distance and time spent in the platform quadrant were raised in the donepezil group (*P* < 0.05), while the TF group exhibited a decrease in the escape latency and distance and an increase in the distance and time spent in the first quadrant and the distance and time spent in the platform quadrant (*P* < 0.05). To sum up, these data reveal that TF exerts a good effect on VaD.

### 3.3. TF Shows a Protective Effect on the *Hippocampus*

The purpose of researching is to find whether TF exerts a better protective effect on hippocampus neuronal ischemia/hypoxia stress in VaD rats with nerve injury in the CA1 region induced by 2-VO surgery. We performed HE staining in the hippocampus of rats in each group to observe the neurons, which could be seen under the microscope (Figure 2(g)). In the model group, the neurons in the hippocampus CA1 region were visible with nuclear pyknosis, deep staining, and unclear nucleolus, the arrangement of cells was disordered and nerve fibers were sparse, and there was reduction in neuronal density, while morphologically, neurons with round shape were normally seen throughout the hippocampus in the sham group and TF group. Compared with the model group, the neurons in the hippocampus CA1 region of the sham group were arranged neatly and compactly, with round or oval cells, obvious nucleus, and more number. The arrangement of cells in the CA1 area in the TF group was more orderly than that in the model group, and the number of cells in the TF group was more than that in the model group, indicating that TF exerts a protective effect on the hippocampus in the VaD rat model.

### 3.4. TF Improves the Behavior in AD Mice and Delays the Paralysis Time of *C. elegans*

**Figure 3:**
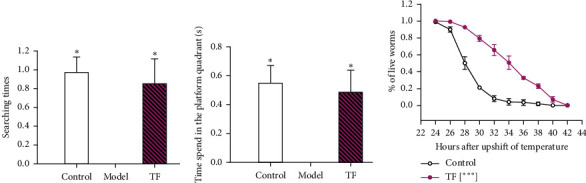
Effect of TF in AD mice and C. elegans. (a) The search times of the TF-treated AD rat model; (b) time spent; and (c) TF delayed the paralysis time of C *elegans*; the abovementioned data were analyzed by the SNK method in one-way ANOVA. ^*∗*^*P* < 0.05, ^*∗∗*^*P* < 0.01, and ^*∗∗∗*^*P* < 0.001 vs. model group.

We next validated whether TF physically impacts A*β* deposition. The effects of TF on AD mice were detected via the Morris water maze. Compared with AD mice, the searching times in the platform quadrant (Figure 3(a)) and the time spent in the platform quadrant (Figure 3(b)) for AD mice were significantly higher after TF treatment, manifesting that TF could improve the learning and memory ability of AD mice, relieve A*β* deposition, and delay aging. Besides, the *C. elegans* paralysis study based on the A*β* deposition *C. elegans* model was carried out. Compared with the model group (Figure 3(c)), TF administration significantly delayed the paralysis time of *C. elegans*. These results demonstrate that TF not only improves the cognitive function of learning and memory but also delays the deposition of A*β*.

### 3.5. DEGs Analysis Reveals the Regulatory Scope of TF

**Figure 4:**
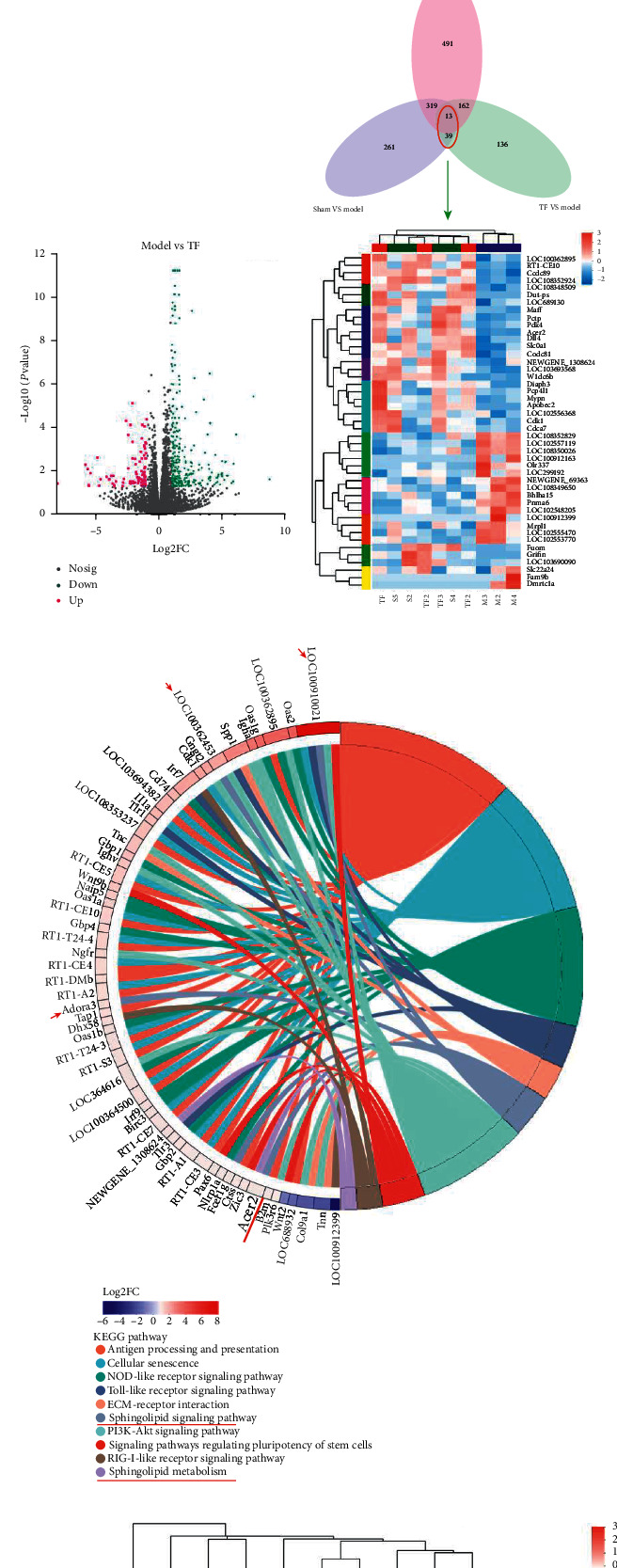
Distribution and screening of DEGs and functional enrichment analysis of VaD treated with TF. (a) Principal component analysis showed that there were significant differences among groups. (b) In the model group, 223 and 409 genes were upregulated and downregulated to the sham group, respectively. (c) In the TF group, 263 and 87 genes were upregulated and downregulated to the model group, respectively. (d) Venn diagram showed the overlap of DEGs in each group, and cluster analysis screened 45 core DEGs by the TF treatment of VaD. (e) Detailed relationship between DEGs and major pathways were annotated by the KEGG pathway by the Circos graph. DEGs in the VaD model group, with log2FC > 2, were chosen to be shown on the left side of the graph. Representative signaling pathways were shown on the right side. (f) Representative genes of the sphingolipid signaling pathway and sphingolipid metabolism related to VaD were presented by the heatmap based on the gene expression value (log2 FC). The red color indicated upregulation, and the blue indicated downregulation.

For further understanding the mechanism of TF against VaD, RNA-seq on the rat model of VaD treated with TF was carried out. There were significant differences among the sham group, VaD group, and TF group, and the biological repeatability was good in each group (Figure 4(a)). Compared with the sham group, the expression of 632 DEGs in the model group changed significantly, of which 223 DEGs were downregulated and 409 DEGs were upregulated (Figure 4(b)). TF regulated the expression of 350 DEGs in the VaD model, of which 263 DEGs were downregulated and 87 DEGs were upregulated (Figure 4(c)). Only 52 genes were coregulated by VaD surgery (model vs. sham) and TF therapy (model vs. TF) (Figure 4(d)) through the intersection of all DEGs in the three comparisons, indicating that TF not only ameliorates neural function by reversing abnormal gene expression caused by VaD but also repairs neural cognitive function by regulating other targets and multiple functional clusters. This information implies that TF treats VaD by influencing the development of the pathological process.

To study the function of the VaD rat model regulated by TF, we analyzed the KEGG function enrichment of DEGs in TF vs. model. We selected the major pathways involved in neurological diseases and showed the detailed relationship between them through the Circos diagram (Figure 4(e)). Pathway analysis classified genes heavily involved in antigen processing and presentation, cell adhesion molecules (CAMs), cellular senescence, the NOD-like receptor signaling pathway, the toll-like receptor signaling pathway, ECM-receptor interaction, the sphingolipid signaling pathway, the PI3K-Akt signaling pathway, signaling pathways regulating pluripotency of stem cells, the RIG-I-like receptor signaling pathway, and others, indicating that TF antagonizes VaD through signal transduction, inflammation and immunity, stem cells, the cellular process, and so on.

### 3.6. TF Improves Neurocognitive Function

**Figure 5:**
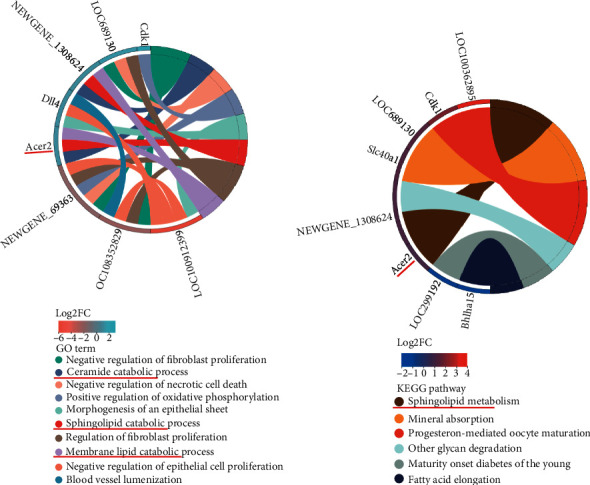
The results of functional enrichment analysis were based on 45 core DEGs. (a) The detailed relationship between the DEGs enriched through GO functional enrichment by the Circos diagram. The enriched DEGs were shown on the left side of the picture. The representative GO term has shown on the right and the DEG in sham vs. model and TF vs. model, with log2 FC > 2; Go terms were both *P* < 0.05 and significant TOP10. (b) Detailed relationship between DEGs and major pathways by KEGG functional enrichment via the Circos diagram. The enriched DEGs were shown on the left side of the picture. The representative KEGG pathways were shown on the right and the DEG in sham vs. model and TF vs. model, with log2 FC > 2; the KEGG pathway is *P* < 0.05.

In addition, we found that TF also regulated a few DEGs that are not significantly regulated in VaD surgery. To scientifically study the mechanism of TF in the treatment of VaD, we conducted a cluster analysis of 52 DEGs (Figure 5(a)) and screened 45 core DEGs that the VaD model could change the gene expression of the control group, while TF treatment could reverse their expression. The DEGs were classified by GO functional enrichment analysis. The highly enriched functional clusters by 45 core DEGs involved mainly in the negative regulation of fibroblast proliferation, ceramide catabolic process, negative regulation of necrotic cell death, positive regulation of oxidative phosphorylation, morphogenesis of an epithelial sheet, sphingolipid catabolic process, regulation of fibroblast proliferation, membrane lipid catabolic process, negative regulation of epithelial cell proliferation, and blood vessel lumenization, suggesting that TF works by repairing nerve and vascular damage and improving neurological function in VaD rats.

### 3.7. KEGG Pathway Enrichment Elucidates Key Signal Pathways

With the aim of better probing the signal transduction pathway regulated by TF in VaD therapy, 45 core DEGs were enriched and analyzed via KEGG pathway enrichment (Figure 5(b)). Pathway analysis classified genes heavily involved in the sphingolipid metabolism, mineral absorption, progesterone-mediated oocyte maturation, other glycan degradation, maturity onset diabetes of the young, and fatty acid elongation. Interestingly, the results of KEGG enrichment analysis had similar outcomes to those of GO enrichment analysis, related to the sphingolipid signaling pathway (Figures 4(e), 5(a), and 5(b)), as well as TF treatment could significantly enhance the expression of ACER2 while VaD could significantly reduce its expression. These data manifest that TF exerts an effective effect in the treatment of VaD through the regulation of the sphingolipid metabolism by ACER2.

### 3.8. The mRNA Expressions of the 6 Key DEGs' Assays by qPCR

**Figure 6:**
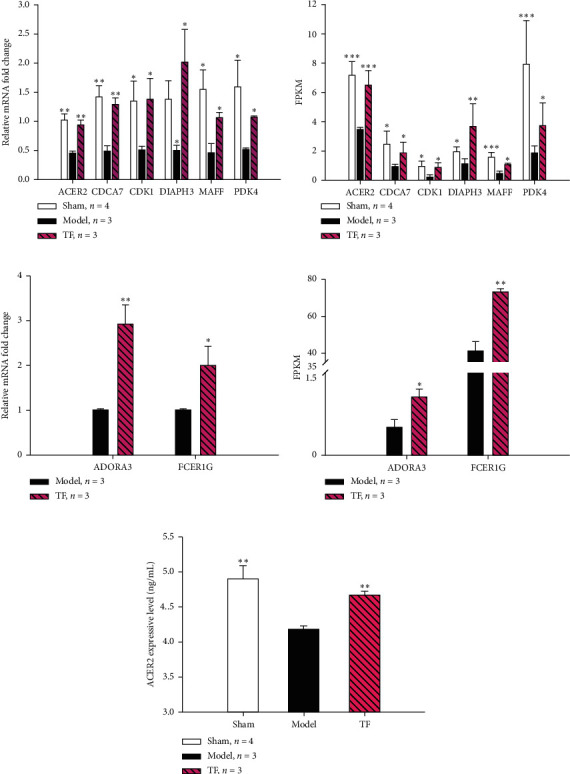
Experimental validation of the expression of DEGs regulated by TF in their protective effect and sphingolipid signaling/metabolism pathway. (a) The mRNA expression of 6 nerve-related core DEGs from TF by qPCR. Six key genes related to neurological function were screened by cluster analysis and literature searching. (b) The mRNA expression of 6 core DEGs via RNA-seq analysis. (c) The mRNA expression of ADORA3 and FCER1G by qPCR. (d) The mRNA expression of ADORA3 and FCER1G from RNA-seq analysis. (e) The protein expression of ACER2 by ELISA. The abovementioned data were analyzed by the SNK method in one-way ANOVA; ^*∗*^*P* < 0.05, ^*∗∗*^*P* < 0.01, and ^*∗∗∗*^*P* < 0.001 vs. model group.

To validate the efficacy of TF in the treatment of VaD on reduced neurological function, 6 of the 45 core genes of RNA-seq are closely related to neurological function through literature searching. Briefly, ACER2 is a key enzyme in the synthesis of SPH and S1P [48, 49]; S1P reduces apoptosis in neuroepithelial cells and regulates vascular development [50]; MAFF plays an important role in neuronal disease [51]; DIAPH3 involves in cell migration, axon induction, and neurogenesis [52]; CDCA7 regulates neurodevelopment [53]; and CDK1 activates neuronal stem cell mitosis [54]. Compared with the VaD model group, the expression of ACER2, PDK4, MAFF, CDCA7, CDK, and DIAPH3 in TF and sham groups were significantly increased via qPCR (Figure 6(a)), which were consistent with the results of RNA-seq (Figure 6(b)), confirming that TF can improve neurological function in VaD model rats by regulating the expression of neurological functional genes.

### 3.9. The Effect of TF on the Sphingolipid Signaling Pathway

The sphingolipid signaling pathway and sphingolipid metabolism were considered to be one of the key pathways of TF on VaD (Figures 4(e), 5(a), 5(b), 6(b), and 6(d)). Also, the sphingolipid metabolism is a part of the sphingolipid signaling pathway as well. Sphingolipids possess an extensive scope of functions, covering almost all major aspects of cell biology, including cell growth, cell cycle, cell death, cell senescence, inflammation, immune response, cell adhesion and migration, angiogenesis, nutrient uptake, and metabolism, and are drivers of neurodevelopment [55]. Aiming to validate the results of RNA-seq analysis, 3 DEGs in the sphingolipid signaling pathway and sphingolipid metabolism (Figures 4(e), 4(f), 5(a), and 5(b)), containing ADORA3, FCER1G, and ACER2 in the cerebral cortex, were validated via qPCR. The qPCR results (Figures 6(a) and 6(c)) were consistent with the RNA-seq data (Figures 6(b) and 6(d)), suggesting the RNA-seq data are reliable.

ACER2 was the key target of the sphingolipid signaling pathway of TF for VaD from Figures 4(e), 4(f), 5(a), and 5(b). VaD inhibited ACER2 transcription and increased its expression significantly after administrating TF (Figures 6(a) and 6(b)). Consistent with qPCR analysis, ACER2 protein levels were changed significantly through ELISA (Figure 6(e)). These results suggest that the treatment of VaD with TF promotes the expression of ACER2, ADORA3, and FCER1G, especially by ACER2, and activates the sphingolipid signaling/metabolism pathway to improve neurological function.

### 3.10. Network Pharmacological Analysis: TF Exhibited a Synergistic Effect on VaD Treatment and May Improve Neurocognitive Function through the Sphingolipid Signaling Pathway

**Figure 7:**
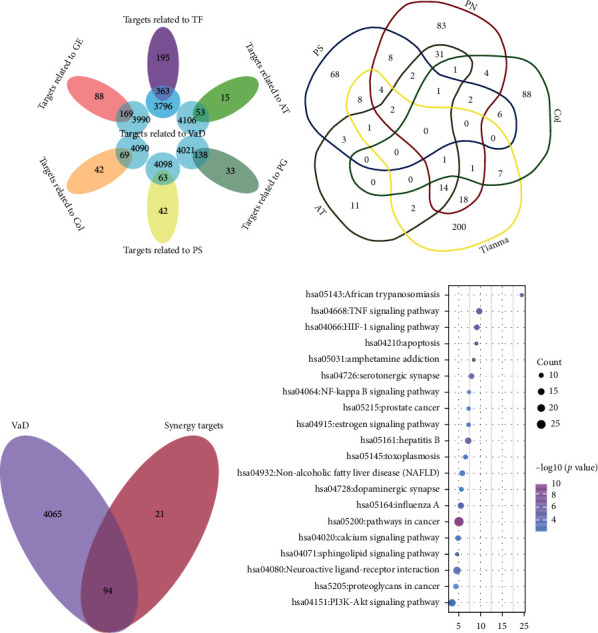
Network pharmacological analysis of TF and VaD. (a) Venn diagram showing the overlapping targets between TF including five traditional Chinese medicines and VaD-related targets; (b) Venn diagram exhibiting the overlapping synergetic targets between the five components in TF; (c) Venn diagram illustrating the overlapping synergetic therapeutic targets between TF and VaD; and (d) significantly representative enriched clusters of the KEGG pathway.

In order to confirm the therapeutic mechanism of TF on VaD, its complex multicompound and multitarget interactions were predicted and analyzed using network pharmacology. 564 regulatory targets were obtained from 49 compounds associated with TF via searching the databases of TCMSP, Pubchem, Batman-TCM, and Swiss Target Prediction (Table S1). According to the intersection of all potential targets in the five ingredients of TF and VaD (Figure 7(a), Tables S1, and S2), we discovered that 80.7% of the targets were coregulated by PN and VaD; 77.9% by AT and VaD; 65.8% by Tianma and VaD; 62.2% by Col and VaD; and 60% by PS and VaD; similarly, 65.1% were coregulated by TF and VaD. Also, 115 targets, coregulated via at least two ingredients of TF, were acquired (Figure 7(b)), of which 94 targets were associated with VaD (Figure 7(c)). Almost all of the 94 targets were implicated in the pathological process of VaD. These consequences hint that TF exerts powerful therapeutic effects on VaD.

To further explore the synergistic functional attribution and the signal transduction pathways implicated in the effects of TF regulation, 94 synergistic targets were analyzed by KEGG functional analysis. We selected the most significant pathways, and the details were shown in Figure 7(d). Pathway analysis classified targets heavily involved in the sphingolipid signaling pathway and other anti-inflammatory, immune, and nerve-related functional regulation pathways. Notably, consistent with RNA-seq results, the sphingolipid signaling pathway was a key pathway for the synergistic effect of TF for VaD via network pharmacological analysis. These data imply that the synergistic targets of TF are significantly related to the pathological process of VaD, and its synergetic therapeutic mechanism is physically concerned with the sphingolipid signaling pathway.

### 3.11. Molecular Docking: The Active Compounds of TF Have a Good Affinity for Sphingolipid and A*β*-Related Proteins

Virtual screening is one of the most important drug research and development technologies in addition to high-throughput screening, and molecular docking is an indispensable technical manner in the process of screening active compounds. It can helpfully predict the conformation of ligand-receptor binding. The more stable the binding is, the lower the binding energy is. The optimal binding values of the active compounds of the five components of TF with key targets were shown in Table 1. Moreover, cycloartenol, ethoxysanguinarine, ginsenoside *f*2, and clionasterol own the lowest binding energies to A*β* 14–46, APP1, FCER1G, and SPHK1, respectively.

**Figure 8:**
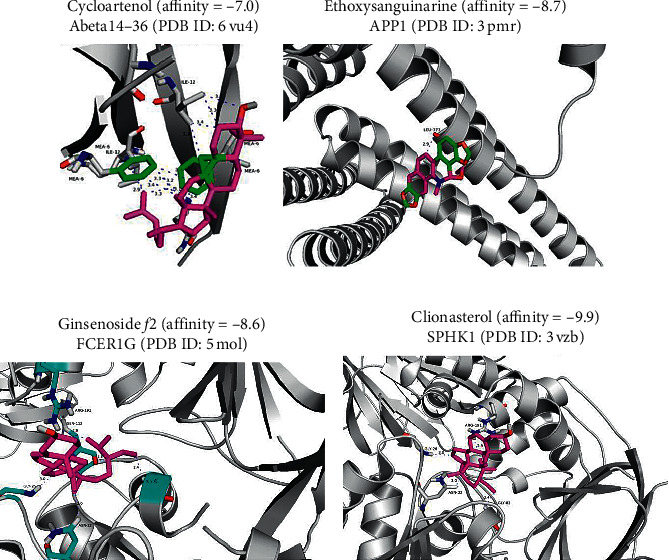
3D interaction diagrams of the lowest binding energy chemicals in the active sites of key antidementia targets in TF. (a) 3D interaction diagram of cycloartenol in the active site of A*β*14-46 (PDB ID: 6vu4). (b) 3D interaction diagram of ethoxysanguinarine in the active site of APP1 (PDB ID: 3pmr). (c) 3D interaction diagram of ginsenoside *f*2 in the active site of FCER1G (PDB ID: 5 mol). (d) 3D interaction diagram of clionasterol in the active site of SPHK1 (PDB ID: 3vzb).

To visualize the docking results, we had drawn a 3D interaction diagram of the key targets and their best matching compounds (Figure 8). Compared with other key targets, cycloartenol exposed a high affinity for A*β* 14–46, and the ligand formed twelve hydrogen bonds with the residues MEA6 and ILE12 of the target protein in the 3D interaction diagram (Figure 8(a)); Moreover, the 3D binding map of ethoxysanguinarine and APP1 revealed good binding stability, and it formed a hydrogen bond with protein residue LEU373 (Figure 8(b)); additionally, from the interaction diagram between ginsenoside *f*2 and FCER1G, it can also be seen that it binds to the residues such as ARG191, SER112, GLY26, ASN22, and GLY82 of the protein active site to form five hydrogen bonds (Figure 8(c)), indicating its stability. Similarly, the 3D docking diagram of clionasterol and SPHK1 shows that it forms hydrogen bonds with the four residues of GLY26, ARG191, ASN22, and GLY82 in the protein (Figure 8(d)), respectively, which has a strong affinity. The results of Table 1 and Figure 8 indicate that TF is well combined with A*β* 14–46 and SPHK1. It is proved that TF can regulate the sphingolipid metabolic pathway in the treatment of dementia, inhibit the expression of APP1, and delay the deposition of A*β*.

## 4. Discussion

**Figure 9:**
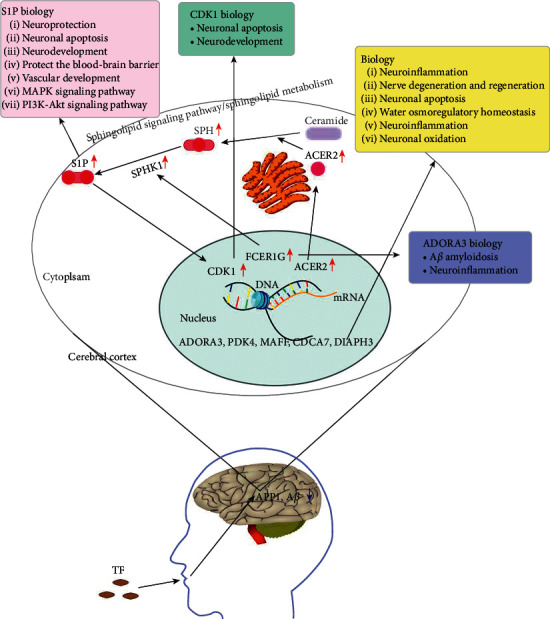
Mechanism diagram of TF against dementia. Results were derived from experimental demonstration, literature searching, and the KEGG PATHWAY database.

Tianma has been used in the treatment of nervous system diseases in China for thousands of years [57], and TF is based on the concerted application of traditional Chinese medicine to select the compound prescription of traditional Chinese medicine which is more effective for nervous system diseases. AD and VaD, with the classical neurofibrillary tangles and senile A*β* plaques of AD together with the cerebral infarcts of VaD, are radiological and neuropathological features that many patients with dementia have [58]. Also, A*β*/tau pathology plays a key role in the progression of cerebrovascular diseases such as AD and VaD [59]. APP (the amyloid precursor protein) plays a greater role in AD pathogenesis as the precursor for A*β* peptides: both the abnormal cleavage of APP leading to A*β* peptide accumulation and the disruption of APP physiological functions contribute to AD pathogenesis [60]. Presenilin-1 (PSEN1), the most frequently mutated gene in familial AD, contributes to AD pathogenesis by overproducing toxic A*β* species and enhancing tau phosphorylation [61]. The effects of TF had been verified by the pharmacodynamic study of VaD rats and AD mice (APP/PS1 mice). Then, the mechanism and efficacy of TF were further determined through ELIS, the A*β* deposition *C. elegans* model, and histopathological sections. On the basis of RNA-seq analysis, the molecular mechanism of TF on VaD was mainly the sphingolipid signaling pathway, which was further confirmed by network pharmacology. Finally, the conjecture (Figure 9) from RNA-seq and network pharmacology was validated by experimental verification (Figure 6) and molecular docking (Figure 8). A series of studies on VaD and AD with TF reveal that TF provides therapeutic benefits and offers new drug candidates for the prevention and treatment of dementia.

In order to verify the efficacy of TF on dementia including VaD and AD, TF significantly improved the spatial memory function of VaD model rats, and its curative effect was better than that of donepezil through the pharmacodynamic study of VaD rats. TF enhanced synaptic plasticity, nerve growth, and repair attenuated the deposit of tau and A*β* and neuroinflammation, as well as protected the hippocampus by inhibiting apoptosis under the pathological sections and ELISA of the hippocampus, APP/PS1 transgenic mice, and A*β* deposition *C. elegans* model. Although we determine that the treatment of TF has a positive effect on other types of dementia such as VaD and AD, it is not clear how they work together to achieve therapeutic results.

To illustrate the therapeutic mechanism of TF, RNA-seq was performed to analyze the molecular mechanism of TF on VaD. The DEGs of TF vs. model were screened by Venn analysis, and the direction of TF mechanism regulation was pointed out through KEGG enrichment analysis. However, the enrichment results obtained by TF vs. model DEGs were not pretty accurate. To accurately locate the therapeutic mechanism of TF, 45 core DEGs (VaD changed the gene expression of the control group, while TF treatment reversed the gene expression) were directly screened through cluster analysis. Thus, the sphingolipid signaling pathway was the key pathway (Figures 4(e), 5(a), and 5(b)) under the functional enrichment analysis of GO and KEGG based on the 45 core DEGs. Also, 6 DEGs, ACER2, PDK4, MAFF, CDCA7, DIAPH3, and DIAPH3, are closely related to the regulation of neurological function with searching the literature. We carried out qPCR assays on these 6 core DEGs and confirmed the efficacy of TF. The results of qPCR were consistent with those of RNA-seq (Figures 6(a) and 6(b)), which showed that the conclusion of RNA-seq analysis was reliable. We infer that the molecular mechanism of TF on VaD may regulate the sphingolipid signaling pathway and the expression of these neural-function-related genes, reducing neuronal apoptosis, inhibiting neuroinflammation, and promoting neuronal production and development.

With the objectives of supporting our inference, network pharmacology was performed to analyze the possible synergistic mechanism of TF. Remarkably, the possible synergistic mechanism of TF on VaD coincided with the results of RNA-seq analysis through Venn and enrichment analysis (Figures 7(d)); that is, the synergistic mechanisms of TF on VaD were associated with a sphingolipid signaling pathway, anti-inflammation, immunity, inhibition of apoptosis, and other related pathways.

Based on a series of analyses, the sphingolipid signaling pathway was the main pathway of TF on VaD. That was activated by verifying the expression of ACER2, ADORA3, and FCER1G at the transcriptional level by qPCR. Meanwhile, the sphingolipid metabolism can be enabled by ACER2. The sphingolipid signaling pathway is closely related to cell senescence, neuroinflammation, immune response, neuron apoptosis, angiogenesis, and protection of the blood-brain barrier [55]. The simplest sphingolipid is also a common precursor for the synthesis of more complex sphingolipids. The hydrolysis of ceramide to SPH requires the participation of the key hydrolase ACER2. In mammalian cells, SPH cannot be synthesized by an anabolic pathway, so it must be produced by catabolism, that is, ceramide hydrolysis, which requires the participation of acidic, neutral, and alkaline ceramidase subtypes, which are encoded by five different genes: ASAH1, ASAH2, ACER1, ACER2, and ACER3 [62, 63]. It is well documented that knockout of ACER2 can significantly reduce the levels of SPH and S1P, but knockout of ASAH1, ASAH2, ACER1, and ACER3 cannot reduce the levels of SPH and S1P [48, 49, 64, 65], manifesting that only the increase of ACER2 expression can effectively rise the content of SPH and S1P. SPH metabolites in sphingolipids, especially S1P, have a wide range of cellular functions and play important neurospecific roles, such as regulating the release of neurotransmitters [66]. Also, one of the important functions of S1P in the central nervous system is its role in nerve development and existence. Recent studies have validated that S1P is involved in the pathogenesis of AD, Parkinson's disease (PD), and Huntington's disease (HD), and the reduction of S1P level are closely correlative with AD [67, 68], PD [69], and HD [70]. It is well documented that S1P is quite important for vascular development by knocking out the Sphk (sphingosine kinase) gene, which is the key enzyme in the synthesis of S1P from SPH. Also, Sphk1/Sphk2 double deletion and S1P receptor can increase apoptosis and decrease mitosis in neuroepithelial cells, most significantly in the forebrain; these genes are highly expressed in the forebrain [50] and suppress A*β* secretion and deposition [71] and A*β-*induced working memory deficit detected [72], demonstrating that the S1P signal plays a crucial role in cortical neurogenesis, A*β* secretion, and deposition. In addition, regulating S1P production not only protects the blood-brain barrier (BBB is an early event that leads to many central nervous system diseases) [73] but also exerts neuroprotective effects [74]. S1P inhibits neuronal apoptosis by upregulating CDK1 as well [75]. FCER1G is defined as a risk factor for AD genetic nodes in late-onset AD, and there is a strong correlation between cortical A*β* amyloidosis and neuroinflammation [76]. At the same time, we verified the close relationship between TF and A*β*, APP1, FCER1G, and SPHK1 by molecular docking. These indicate that the sphingolipid signaling pathway is crucial for the therapeutic effects of TF. Thus, we verified that TF improved the function of spatial memory, inhibited neuronal apoptosis, protected hippocampal tissue and suppressed A*β* secretion and deposition, and activated the sphingolipid signaling pathway by increasing the expression of ACER2, ADORA3, and FCER1G and then increased downstream S1P and CDK1, inhibiting nerve cell apoptosis, Tau, A*β* amyloidosis, and neuroinflammation, further protecting nerve function and improving cognitive ability.

In this study, we confirmed that the sphingolipid signaling/metabolism pathway activated by ACER2 is the core therapeutic mechanism of TF. But, there may be multiple mechanisms for therapeutic regulation from the analysis of network pharmacology and RNA-seq. Hence, we need to further research the therapeutic mechanism of the sphingolipid signaling pathway and other pathways regulated by TF in the future. In previous studies, the relationship between sphingolipids and neurodegenerative diseases has been confirmed, but its regulatory mechanism in dementia-like VaD and AD is not clear. Also, we determine that the sphingolipid signaling/metabolism pathway activated by ACER2, ADORA3, and FCER1G may play a momentous role in the pathogenesis of VaD and A*β* amyloidosis.

In summary, TF upregulates the expression of ACER2, ADORA3, and FCER1G in the brain and activates the sphingolipid signaling pathway and sphingolipid metabolism. ACER2 promotes the synthesis of S1P, which is promoting the expression of CDK1, reducing neuronal apoptosis, regulating multiple nerve-related genes to protect the BBB, repairing cerebral vessels, inhibiting neuritis, and promoting neuronal production and development. The increase of FCER1G may delay A*β* deposition and inhibit neuritis, as well as multiple nerve-related genes, improving neurological function. Further studies on the bioactive components of TF would be beneficial to the development of dementia drugs. In this study, a combination of traditional Chinese medicine formula was proposed as the combined treatment of dementia, which provides a new prospect for clinical application and new drug development. Simultaneously, we also found that the increase of ACER2 could contribute to the improvement of SPH and S1P synthesis and the promotion of FER1G may lead to the relief of A*β* deposition, which is closely related to the pathogenesis of dementia, providing a potential new biomarker for the diagnosis of dementia and even mental diseases.

## Figures and Tables

**Table 1:** The results of molecular docking of active ingredients and key targets in TF (the binding energy less than −4.25 kcal·mol^−1^ indicates that the ligand has a certain binding activity to the receptor, less than −5.0 kcal·mol^−1^ has better binding activity, and less than −7.0 kcal·mol^−1^ has strong binding activity [56]).

Chemical name		A*β*14-46	APP1	FCER1G	SPHK1
PN	Beta-sitosterol	−6.6	−7.3	−7.2	−7.9
	Liquiritigenin	−5.8	−8.4	−6.4	−9
	Diisooctyl phthalate	−4	−5.2	−3.9	−6
	Ginsenoside *f*2	−5.6	−8.2	−8.6	−9.1
	Ginsenoside rh2	−5.8	−7.6	−8.4	−7.8
	Ethyl linoleate	−3.5	−3.5	−3.2	−5.4
	Stigmasterol	−6.4	−6.8	−6.6	−9.8

PN + PS	Quercetin	−4.6	−6.6	−7	−8.2

PS	Catechol	−3.2	−5.2	−5.2	−5.2
	Luteolin	−5.4	−8.3	−7.7	−8.7
	Phloroglucinol	−3	−4.9	−4.7	−5.3
	Pyrogallol	−3.2	−5.6	−5.6	−5.3

AT	(3R, 3As, 6R, 6aS)-3, 6-bis (3, 4-dimethoxyphenyl)-1, 3, 3a, 4, 6, 6a-hexahydrofuro [3, 4-c] furan	−5.4	−7.5	−6.7	−8.8
	8-Isopentenyl-kaempferol	−5.4	−8.4	−7.7	−7.5
	Cycloartenol	−7	−8.1	−7.2	−9.7
	Kaempferol	−5.5	−8.1	−7	−8.7

Col	(3r, 4ar, 6r, 6as, 10as, 10br)-3-Ethenyl-6-hydroxy-3, 4a, 7, 7, 10a-pentamethyldodecahydro-1h-naphtho [2, 1-b] pyran-1-one	−5.6	−7.5	−7.3	−7.3
	(3r, 4as, 5r, 6r, 6ar, 10r, 10as, 10br)-3-Ethenyl-5, 6, 10, 10b-tetrahydroxy-3, 4a, 7, 7, 10a-pentamethyl-5, 6, 6a, 8, 9, 10-hexahydro-2h-benzo [f] chromen-1-one	−5.1	−7	−7	−9.3
	[(3r, 4ar, 5s, 6s, 6as, 10as, 10bs)-3-Ethenyl-6,10b-dihydroxy-3, 4a, 7, 7, 10a-pentamethyl-1-oxo-5, 6, 6a, 8, 9, 10-hexahydro-2h-benzo [f] chromen-5-yl] acetate	−4.6	−6.9	−6.7	−7.5
	1, 9-Dideoxyforskolin	−5.3	−7.6	−6.7	−7
	7-Deacetyl-1, 9-dideoxyforskolin	−5.8	−7.7	−6.6	−7.4
	7-Deacetyl-1-deoxyforskolin	−4.8	−7.7	−6.6	−7.4
	7-Desacetylforskolin	−4.7	−7.3	−6.8	−7.5
	9alpha-Hydroxy-8, 13-epoxy-labd-14-en-11-one	−5.1	−7.3	−6.9	−7.2
	9-Deoxyforskolin	−4.9	−7.6	−6.7	−7.2
	Colistin	−4.5	−6.6	−6.5	−7.6
	Forskolin	−5.8	−7.7	−6.6	−7.4

Tianma	3-Hydroxybenzoic acid	−3.9	−5.3	−5.3	−5.8
	4-(4′-Hydroxybenzyloxy) benzyl methyl ether	−3.8	−4.7	−4.8	−5.6
	4-Ethoxymethylphenyl-4′-hydroxybenzylether	−5.2	−6.6	−5.4	−7.2
	4-Hydroxybenzaldehyde	−3.8	−4.7	−4.8	−5.6
	4-Hydroxybenzyl alcohol	−3.8	−4.8	−4.8	−5.3
	4-Hydroxybenzylamine	−3.4	−4.4	−4.7	−5
	7-Hydroxybiopterin	−4.2	−6.2	−6.9	−7.6
	20-Hexadecanoylingenol	−5	−7.5	−5.2	−6.7
	Bis (4-hydroxybenzyl) ether	−5.2	−7.5	−6.3	−7
	Citronellal	−4.1	−4.4	−4.3	−5.1
	Clionasterol	−6.4	−7.3	−7.2	−9.9
	Daucosterol	−6.6	−8	−7.4	−9.5
	Dauricine	−7	−8.3	−7.1	−8.7
	Ethoxysanguinarine	−6.2	−8.7	−8.1	−8.9
	Gastrodamine	−4.9	−7.6	−6.7	−7.2
	Gastrodin	−4.6	−5.6	−6.6	−7.6
	Gaultheroside A	−4.2	−7	−7	−8.2
	Suchilactone	−4.8	−7.6	−7.5	−8.1
	Sucrose	−4.1	−5.7	−6.4	−6.9
	Suffruticoside A	−5.2	−6.5	−7.3	−9.7
	Vanillin	−3.9	−5.2	−5.3	−5.8
	Vanillin acetate	−3.2	−5.2	−4.8	−5
	Vanillyl alcohol	−3.6	−4.6	−5.1	−5.8

## Data Availability

The data were obtained from PubChem database: https://pubchem.ncbi.nlm.nih.gov/; ChemSpider database: http://www.chemspider.com/; TCMSP database: http://tcmspw.com/tcmsp.php; SwissTargetPredict database: http://www.swisstargetprediction.ch/; BATMAN-TCM database: http://bionet.ncpsb.org/batman-tcm/; PDB database: https://www.rcsb.org/; STRING database: https://string-db.org/; SRA database: https://www.ncbi.nlm.nih.gov/sra/; DAVID database: https://david.ncifcrf.gov/tools.jsp; and Sequence Read Archive (SRA) database: https://www.ncbi.nlm.nih.gov/sra/?term=.

## Supplementary Materials

Supplementary Materials Supplementary Table: Table S1. Basic information of TF-related targets.
