# Supplementary material for: Tianma Formula Alleviates Dementia via ACER2-Mediated Sphingolipid Signaling Pathway Involving Aβ
Source: Evid Based Complement Alternat Med. 2021 Aug 4;2021:6029237. doi: 10.1155/2021/6029237 (PMC8357478; doi:10.1155/2021/6029237)
Supplement: Supplementary Materials — Supplementary Table: Table S1. Basic information of TF-related targets. [file 6029237.f1.docx]

Supplementary Material

Supplementary Table:

Table S1. Basic information of TF related targets

| Ingredient | Compound name | target |
| --- | --- | --- |
| PN | Mandenol | PTGS1 |
|  |  | PTGS2 |
|  |  | NCOA2 |
|  | Liquiritigenin | PTGS1 |
|  |  | ESR2 |
|  |  | PTGS2 |
|  |  | RXRA |
|  |  | ADRB2 |
|  |  | HSP90A |
|  |  | LACTBL1 |
|  |  | MAOB |
|  |  | SLC6A4 |
|  |  | PKIA |
|  | Diisooctyl phthalate | SCN5A |
|  |  | ADRB2 |
|  |  | CHRM3 |
|  | Beta-sitosterol | PGR |
|  |  | NCOA2 |
|  |  | PTGS1 |
|  |  | PTGS2 |
|  |  | HSP90A |
|  |  | KCNH2 |
|  |  | DRD1 |
|  |  | CHRM3 |
|  |  | CHRM1 |
|  |  | SCN5A |
|  |  | CHRM4 |
|  |  | PDE3A |
|  |  | HTR2A |
|  |  | ADRA1A |
|  |  | CHRM2 |
|  |  | ADRA1B |
|  |  | ADRB2 |
|  |  | CHRNA2 |
|  |  | SLC6A4 |
|  |  | OPRM1 |
|  |  | GABRA1 |
|  |  | BCL2 |
|  |  | BAX |
|  |  | CASP9 |
|  |  | JUN |
|  |  | CASP3 |
|  |  | CASP8 |
|  |  | PRKCA |
|  |  | TGFB1 |
|  |  | PON1 |
|  |  | MAP2 |
|  | Stigmasterol | PGR |
|  |  | NR3C2 |
|  |  | NCOA2 |
|  |  | ADH1C |
|  |  | RXRA |
|  |  | NCOA1 |
|  |  | PTGS1 |
|  |  | PTGS2 |
|  |  | ADRA2A |
|  |  | SLC6A2 |
|  |  | SLC6A3 |
|  |  | ADRB2 |
|  |  | AKR1B1 |
|  |  | PLAU |
|  |  | LTA4H |
|  |  | MAOB |
|  |  | MAOA |
|  |  | CTRB1 |
|  |  | CHRM3 |
|  |  | CHRM1 |
|  |  | ADRB1 |
|  |  | SCN5A |
|  |  | HTR2A |
|  |  | ADRA1A |
|  |  | CHRM2 |
|  |  | ADRA1B |
|  |  | GABRA1 |
|  | Ginsenoside rh2 | BAX |
|  |  | TNF |
|  |  | CASP3 |
|  |  | PTGS2 |
|  |  | NFKBIA |
|  |  | IL1B |
|  |  | CASP1 |
|  |  | IFNG |
|  |  | ADCYAP1 |
|  |  | PSMG1 |
|  |  | MAP2K4 |
|  |  | SLC2A4 |
|  | Quercetin | PTGS1 |
|  |  | AR |
|  |  | PPARG |
|  |  | PTGS2 |
|  |  | HSP90A |
|  |  | NCOA2 |
|  |  | DPP4 |
|  |  | AKR1B1 |
|  |  | PRSS1 |
|  |  | TOP2 |
|  |  | KCNH2 |
|  |  | SCN5A |
|  |  | ADRB2 |
|  |  | MMP3 |
|  |  | F7 |
|  |  | RXRA |
|  |  | ACHE |
|  |  | GABRA1 |
|  |  | MAOB |
|  |  | RELA |
|  |  | EGFR |
|  |  | AKT1 |
|  |  | VEGFA |
|  |  | CCND1 |
|  |  | BCL2 |
|  |  | BCL2L1 |
|  |  | FOS |
|  |  | CDKN1A |
|  |  | EIF6 |
|  |  | BAX |
|  |  | CASP9 |
|  |  | PLAU |
|  |  | MMP2 |
|  |  | MMP9 |
|  |  | MAPK1 |
|  |  | IL10 |
|  |  | EGF |
|  |  | RB1 |
|  |  | TNF |
|  |  | JUN |
|  |  | IL6 |
|  |  | AHSA1 |
|  |  | CASP3 |
|  |  | TP53 |
|  |  | ELK1 |
|  |  | NFKBIA |
|  |  | POR |
|  |  | ODC1 |
|  |  | XDH |
|  |  | CASP8 |
|  |  | TOP1 |
|  |  | RAF1 |
|  |  | SOD1 |
|  |  | PRKCA |
|  |  | MMP1 |
|  |  | HIF1A |
|  |  | STAT1 |
|  |  | RUNX1T1 |
|  |  | HSPA5 |
|  |  | ERBB2 |
|  |  | PPARG |
|  |  | ACACA |
|  |  | HMOX1 |
|  |  | CYP3A4 |
|  |  | CYP1A2 |
|  |  | CAV1 |
|  |  | MYC |
|  |  | F3 |
|  |  | GJA1 |
|  |  | CYP1A1 |
|  |  | ICAM1 |
|  |  | IL1B |
|  |  | CCL2 |
|  |  | SELE |
|  |  | VCAM1 |
|  |  | PTGER3 |
|  |  | CXCL8 |
|  |  | PRKCB |
|  |  | BIRC5 |
|  |  | DUOX2 |
|  |  | NOS3 |
|  |  | HSPB1 |
|  |  | TGFB1 |
|  |  | SULT1E1 |
|  |  | MGAM |
|  |  | IL2 |
|  |  | NR1I2 |
|  |  | CYP1B1 |
|  |  | CCNB1 |
|  |  | PLAT |
|  |  | THBD |
|  |  | SERPINE1 |
|  |  | COL1A1 |
|  |  | IFNG |
|  |  | ALOX5 |
|  |  | IL1A |
|  |  | MPO |
|  |  | TOP2A |
|  |  | NCF1 |
|  |  | ABCG2 |
|  |  | HAS2 |
|  |  | GSTP1 |
|  |  | NFE2L2 |
|  |  | NQO1 |
|  |  | PARP1 |
|  |  | AHR |
|  |  | PSMD3 |
|  |  | SLC2A4 |
|  |  | COL3A1 |
|  |  | CXCL11 |
|  |  | CXCL2 |
|  |  | DCAF5 |
|  |  | CHEK2 |
|  |  | INSR |
|  |  | CLDN4 |
|  |  | PPARA |
|  |  | PPARD |
|  |  | HSF1 |
|  |  | CRP |
|  |  | CXCL10 |
|  |  | CHUK |
|  |  | SPP1 |
|  |  | RUNX2 |
|  |  | RASSF1 |
|  |  | E2F1 |
|  |  | E2F2 |
|  |  | ACP3 |
|  |  | IGFBP3 |
|  |  | IGF2 |
|  |  | CD40LG |
|  |  | IRF1 |
|  |  | ERBB3 |
|  |  | PON1 |
|  |  | DIO1 |
|  |  | PCOLCE |
|  |  | NPEPPS |
|  |  | HK2 |
|  |  | NKX3-1 |
|  |  | RASA1 |
|  |  | GSTM1 |
|  |  | GSTM2 |
| AT | 8-Isopentenyl-kaempferol | ESR1 |
|  |  | AR |
|  |  | PPARG |
|  |  | PTGS2 |
|  |  | TOP2 |
|  |  | DPP4 |
|  |  | GSK3B |
|  |  | HSP90A |
|  |  | CDK2 |
|  |  | CHEK1 |
|  |  | PRSS1 |
|  |  | CALM |
|  |  | PTGS1 |
|  |  | SCN5A |
|  |  | F7 |
|  |  | KDR |
|  |  | RXRA |
|  |  | MAPK14 |
|  |  | CCNA2 |
|  |  | NCOA2 |
|  |  | GABRA1 |
|  |  | PDE3A |
|  |  | KCNH2 |
|  |  | PYGM |
|  | (1R,3aS,4R,6aS)-1,4-bis(3,4-dimethoxyphenyl)-1,3,3a,4,6,6a-hexahydrofuro[4,3-c]furan | CHRM3 |
|  |  | KCNH2 |
|  |  | SCN5A |
|  |  | PTGS2 |
|  |  | ADRA1B |
|  |  | ADRA1D |
|  |  | NCOA2 |
|  |  | CALM |
|  | Cycloartenol | NR3C2 |
|  | Kaempferol | NOS2 |
|  |  | PTGS1 |
|  |  | AR |
|  |  | PPARG |
|  |  | PTGS2 |
|  |  | HSP90A |
|  |  | NCOA2 |
|  |  | DPP4 |
|  |  | PRSS1 |
|  |  | PGR |
|  |  | CHRM1 |
|  |  | ACHE |
|  |  | SLC6A2 |
|  |  | CHRM2 |
|  |  | ADRA1B |
|  |  | GABRA1 |
|  |  | TOP2 |
|  |  | F7 |
|  |  | CALM |
|  |  | IKBKB |
|  |  | AKT1 |
|  |  | BCL2 |
|  |  | BAX |
|  |  | TNF |
|  |  | JUN |
|  |  | AHSA1 |
|  |  | CASP3 |
|  |  | MAPK8 |
|  |  | MMP1 |
|  |  | STAT1 |
|  |  | PPARG |
|  |  | HMOX1 |
|  |  | CYP3A4 |
|  |  | CYP1A2 |
|  |  | CYP1A1 |
|  |  | ICAM1 |
|  |  | SELE |
|  |  | VCAM1 |
|  |  | NR1I2 |
|  |  | CYP1B1 |
|  |  | ALOX5 |
|  |  | HAS2 |
|  |  | GSTP1 |
|  |  | AHR |
|  |  | PSMD3 |
|  |  | SLC2A4 |
|  |  | NR1I3 |
|  |  | INSR |
|  |  | DIO1 |
|  |  | PPP3CA |
|  |  | GSTM1 |
|  |  | GSTM2 |
|  |  | AKR1C3 |
|  |  | SLPI |
| Col | 7-Deacetyl-1-deoxyforskolin | NT5C2 |
|  |  | GCDH |
|  |  | POR |
|  |  | TXNRD1 |
|  |  | AIFM1 |
|  |  | XDH |
|  |  | ADK |
|  |  | ERO1B |
|  |  | CYB5R1 |
|  |  | ACADS |
|  |  | ACOX1 |
|  |  | ACAD8 |
|  |  | IMPDH1 |
|  |  | IVD |
|  |  | MAOB |
|  |  | CYB5R3 |
|  |  | ACADM |
|  |  | NOS1 |
|  |  | ENPP1 |
|  |  | NQO2 |
|  |  | FDXR |
|  |  | MAOA |
|  |  | NQO1 |
|  |  | DLD |
|  |  | GSR |
|  |  | DPYD |
|  |  | DAO |
|  |  | IL4I1 |
|  |  | IMPDH2 |
|  |  | GFER |
|  | 1,9-Dideoxyforskolin | FKBP1A |
|  |  | MAP2 |
|  |  | TUBA3C |
|  |  | TUBB1 |
|  |  | MTOR |
|  |  | TUBA3E |
|  |  | MAPT |
|  |  | TUBA4A |
|  |  | FGF2 |
|  |  | NR3C1 |
|  |  | TUBA1C |
|  |  | MAP4 |
|  |  | EHHADH |
|  |  | BCL2 |
|  |  | TUBA1B |
|  |  | TUBA3D |
|  |  | TUBA8 |
|  |  | NR1I2 |
|  |  | FKBP1B |
|  |  | TUBA1A |
|  | 7-Deacetyl-1,9-dideoxyforskolin | NT5C2 |
|  |  | GCDH |
|  |  | POR |
|  |  | TXNRD1 |
|  |  | AIFM1 |
|  |  | ACAD8 |
|  |  | APRT |
|  |  | AKT1 |
|  |  | ADRBK2 |
|  |  | TNK2 |
|  |  | PRKAA1 |
|  |  | PIM1 |
|  |  | HINT1 |
|  |  | PDE4B |
|  |  | ALK |
|  |  | SLC25A4 |
|  |  | ADK |
|  |  | ERO1B |
|  |  | CYB5R1 |
|  |  | ACADS |
|  |  | ACOX1 |
|  |  | NOS1 |
|  |  | AK9 |
|  |  | PRKAB1 |
|  |  | ACSS2 |
|  |  | ASNS |
|  |  | TUBB4B |
|  |  | PYGL |
|  |  | ASS1 |
|  |  | NAE1 |
|  |  | ABCB11 |
|  |  | IMPDH1 |
|  |  | IVD |
|  |  | MAOB |
|  |  | CYB5R3 |
|  |  | ACADM |
|  |  | DLD |
|  |  | IMPDH2 |
|  |  | TUBB3 |
|  |  | ACVRL1 |
|  |  | ABL2 |
|  |  | ABCC9 |
|  |  | ABL1 |
|  |  | ACVR1B |
|  |  | PRKAB2 |
|  |  | ABCB1 |
|  |  | ENPP1 |
|  |  | NQO2 |
|  |  | FDXR |
|  |  | MAOA |
|  |  | NQO1 |
|  |  | GFER |
|  |  | ABCC2 |
|  |  | ACVR1 |
|  |  | ADCY1 |
|  |  | ADRBK1 |
|  |  | CREB1 |
|  |  | AFG3L2 |
|  |  | ACSL1 |
|  |  | APAF1 |
|  |  | ABCG1 |
|  |  | GSR |
|  |  | DPYD |
|  |  | DAO |
|  |  | IL4I1 |
|  |  | XDH |
|  |  | ANKH |
|  |  | FBP1 |
|  |  | ASNA1 |
|  |  | AMHR2 |
|  |  | PDE4D |
|  |  | ACSS1 |
|  |  | CDK15 |
|  |  | ABCA1 |
|  |  | ABCC8 |
|  |  | ARAF |
|  | Forskolin | ADCY5 |
|  |  | BCL2 |
|  |  | TUBA1B |
|  |  | TUBA1A |
|  |  | PTGER4 |
|  |  | FGF2 |
|  |  | ADCY2 |
|  |  | NR1I2 |
|  |  | TUBB1 |
|  |  | FKBP1A |
|  |  | MTOR |
|  |  | GNAS |
|  |  | TUBA3C |
|  |  | TUBA4A |
|  |  | EHHADH |
|  |  | PTGER2 |
|  |  | MAP2 |
|  |  | MAPT |
|  |  | MAP4 |
|  |  | TUBA8 |
|  |  | PTGER3 |
|  |  | TUBA3E |
|  |  | TUBA1C |
|  |  | TUBA3D |
|  |  | GLRA3 |
|  |  | GABRB3 |
|  | 7-Desacetylforskolin | NT5C2 |
|  |  | GCDH |
|  |  | POR |
|  |  | TXNRD1 |
|  |  | AIFM1 |
|  |  | XDH |
|  |  | ADK |
|  |  | ERO1B |
|  |  | CYB5R1 |
|  |  | ACADS |
|  |  | ACOX1 |
|  |  | ACAD8 |
|  |  | IMPDH1 |
|  |  | IVD |
|  |  | MAOB |
|  |  | CYB5R3 |
|  |  | ACADM |
|  |  | NOS1 |
|  |  | ENPP1 |
|  |  | NQO2 |
|  |  | FDXR |
|  |  | MAOA |
|  |  | NQO1 |
|  |  | DLD |
|  |  | GSR |
|  |  | DPYD |
|  |  | DAO |
|  |  | IL4I1 |
|  |  | IMPDH2 |
|  |  | GFER |
|  | 9-Deoxyforskolin | FKBP1A |
|  |  | MAP2 |
|  |  | TUBA3C |
|  |  | TUBB1 |
|  |  | MTOR |
|  |  | TUBA3E |
|  |  | MAPT |
|  |  | TUBA4A |
|  |  | FGF2 |
|  |  | NR3C1 |
|  |  | TUBA1C |
|  |  | MAP4 |
|  |  | EHHADH |
|  |  | BCL2 |
|  |  | TUBA1B |
|  |  | TUBA3D |
|  |  | TUBA8 |
|  |  | NR1I2 |
|  |  | FKBP1B |
|  |  | TUBA1A |
|  | 9alpha-Hydroxy-8,13-epoxy-labd-14-EN-11-one | TERT |
|  | [(3R,4Ar,5S,6S,6aS,10aS,10bS)-3-ethenyl-6,10b-dihydroxy-3,4a,7,7,10a-pentamethyl-1-oxo-5,6,6a,8,9,10-hexahydro-2H-benzo[f]chromen-5-yl] acetate | MAP2 |
|  |  | MAPT |
|  |  | MAP4 |
|  |  | TUBA8 |
|  |  | PTGER3 |
|  |  | TUBA3E |
|  |  | TUBA1C |
|  |  | TUBA3D |
|  |  | GLRA3 |
|  |  | GABRB3 |
|  |  | BCL2 |
|  |  | TUBA1B |
|  |  | TUBA1A |
|  |  | PTGER4 |
|  |  | FGF2 |
|  |  | NR1I2 |
|  |  | TUBB1 |
|  |  | FKBP1A |
|  |  | MTOR |
|  |  | TUBA3C |
|  |  | TUBA4A |
|  |  | EHHADH |
|  |  | PTGER2 |
|  | (3R,4Ar,6R,6aS,10aS,10bR)-3-Ethenyl-6-hydroxy-3,4a,7,7,10a-pentamethyldodecahydro-1H-naphtho[2,1-b]pyran-1-one | TUBB3 |
|  |  | GSR |
|  |  | DPYD |
|  |  | MAOB |
|  |  | ACADS |
|  |  | IL4I1 |
|  |  | ACADM |
|  |  | ACAD8 |
|  |  | NT5C2 |
|  |  | GCDH |
|  |  | TUBB |
|  |  | TUBB6 |
|  |  | CYB5R3 |
|  |  | AIFM1 |
|  |  | NQO1 |
|  |  | NOS1 |
|  |  | ADK |
|  |  | ERO1B |
|  |  | TUBB4A |
|  |  | FDXR |
|  |  | TUBB8 |
|  |  | TUBB4B |
|  |  | TERT |
|  |  | DLD |
|  |  | IMPDH1 |
|  |  | IVD |
|  |  | POR |
|  |  | DAO |
|  |  | TUBB2B |
|  |  | ACOX1 |
|  |  | IMPDH2 |
|  |  | GFER |
|  |  | ENPP1 |
|  |  | NQO2 |
|  |  | CYB5R1 |
|  |  | TXNRD1 |
|  |  | MAOA |
|  |  | TUBB2A |
|  |  | XDH |
|  | Colistin |  |
|  | (3R,4As,5R,6R,6aR,10R,10aS,10bR)-3-ethenyl-5,6,10,10b-tetrahydroxy-3,4a,7,7,10a-pentamethyl-5,6,6a,8,9,10-hexahydro-2H-benzo[f]chromen-1-one | NT5C2 |
|  |  | GCDH |
|  |  | POR |
|  |  | TXNRD1 |
|  |  | AIFM1 |
|  |  | XDH |
|  |  | ADK |
|  |  | ERO1B |
|  |  | CYB5R1 |
|  |  | ACADS |
|  |  | ACOX1 |
|  |  | ACAD8 |
|  |  | IMPDH1 |
|  |  | IVD |
|  |  | MAOB |
|  |  | CYB5R3 |
|  |  | ACADM |
|  |  | NOS1 |
|  |  | ENPP1 |
|  |  | NQO2 |
|  |  | FDXR |
|  |  | MAOA |
|  |  | NQO1 |
|  |  | DLD |
|  |  | GSR |
|  |  | DPYD |
|  |  | DAO |
|  |  | IL4I1 |
|  |  | IMPDH2 |
|  |  | GFER |
| Tianma | Suffruticoside A | TOP2A |
|  |  | TOP2B |
|  | 7-Hydroxybiopterin | AKR1C3 |
|  |  | ESR2 |
|  |  | ESR1 |
|  |  | TH |
|  |  | PTGER1 |
|  |  | TPH1 |
|  |  | PAH |
|  |  | PTGER3 |
|  |  | REN |
|  |  | PTGFR |
|  |  | NOS3 |
|  | Suchilactone |  |
|  | 4-(4'-Hydroxybenzyloxy)Benzyl Methyl Ether | TYR |
|  |  | SEC14L3 |
|  |  | PPP2CA |
|  |  | PRKCA |
|  |  | NR1I2 |
|  |  | ALOX5 |
|  |  | PPP2CB |
|  |  | SEC14L2 |
|  |  | DGKA |
|  |  | PRKCB |
|  |  | SEC14L4 |
|  | 4-Hydroxybenzaldehyde | AKR1C1 |
|  |  | PTGS1 |
|  |  | PTGS2 |
|  |  | PDXK |
|  | Gastrodamine | GABRA2 |
|  |  | GABRD |
|  |  | PDE3A |
|  |  | GABRB1 |
|  |  | GABRG3 |
|  |  | TNF |
|  |  | GABRE |
|  |  | SCN2A |
|  |  | GABRA3 |
|  |  | GABRG1 |
|  |  | GABRP |
|  |  | GABRA4 |
|  |  | GABRB2 |
|  |  | ACHE |
|  |  | SCN4A |
|  |  | GABRQ |
|  |  | GABRB3 |
|  |  | GABRA5 |
|  |  | GABRA6 |
|  |  | GABRA1 |
|  |  | PDE4B |
|  |  | BCHE |
|  |  | GABRG2 |
|  | Clionasterol | ESR1 |
|  |  | PGR |
|  |  | VDR |
|  |  | CYP27B1 |
|  |  | GC |
|  |  | SNW1 |
|  |  | AR |
|  |  | NR3C1 |
|  |  | NFKB1 |
|  |  | AKR1C3 |
|  |  | CYP24A1 |
|  |  | GPBAR1 |
|  |  | SNAI2 |
|  |  | MED1 |
|  |  | SNAI1 |
|  |  | CYP3A4 |
|  |  | CALB1 |
|  |  | FGF23 |
|  |  | GFI1 |
|  |  | LANCL2 |
|  |  | WNT4 |
|  |  | TCF3 |
|  |  | BAX |
|  |  | KL |
|  |  | CYP2R1 |
|  |  | PML |
|  |  | B4GALT1 |
|  |  | S100G |
|  |  | CYP27A1 |
|  |  | KANK2 |
|  |  | IRX5 |
|  |  | RXRA |
|  |  | NR1H4 |
|  |  | TRIM24 |
|  |  | CYP17A1 |
|  |  | TRPV3 |
|  |  | OPRK1 |
|  |  | TRPM8 |
|  |  | TRPA1 |
|  | 4-Hydroxybenzyl Alcohol | PDXK |
|  |  | PNPO |
|  |  | IMPA1 |
|  |  | AKR1C1 |
|  |  | PTGS1 |
|  |  | PTGS2 |
|  |  | PDXP |
|  |  | TPI1 |
|  |  | GPI |
|  |  | PSAT1 |
|  |  | AOX1 |
|  |  | TKT |
|  |  | KDM3A |
|  |  | PDE5A |
|  |  | PDE4A |
|  |  | PDE10A |
|  |  | ADA |
|  | 3-Hydroxybenzoic Acid | AKR1C1 |
|  |  | PTGS1 |
|  |  | PTGS2 |
|  |  | PLA2G2E |
|  |  | AKR1C2 |
|  |  | PPARG |
|  |  | MPO |
|  |  | ALOX5 |
|  |  | IKBKB |
|  |  | CHUK |
|  |  | APOA2 |
|  |  | HSD17B2 |
|  |  | SERPINE1 |
|  |  | IL1B |
|  |  | IL13 |
|  |  | SERPINB7 |
|  |  | AKR1C3 |
|  |  | IFNG |
|  |  | AKR1C4 |
|  |  | TPMT |
|  |  | NDRG2 |
|  |  | NAMPT |
|  |  | RGCC |
|  |  | FNDC5 |
|  |  | NR1H3 |
|  |  | OSBPL8 |
|  |  | AVPR1A |
|  |  | IKBKE |
|  |  | ITGB3 |
|  |  | METRNL |
|  |  | MAPK9 |
|  |  | NCKAP1L |
|  |  | STX3 |
|  |  | OXER1 |
|  |  | PNPLA2 |
|  |  | TNF |
|  |  | PTGIS |
|  |  | HEG1 |
|  |  | ITGAV |
|  |  | PTPN2 |
|  |  | S100A8 |
|  |  | ALOX5AP |
|  |  | AVP |
|  |  | SLC6A4 |
|  |  | ROCK2 |
|  |  | BDKRB2 |
|  |  | NR1H2 |
|  |  | NEUROD2 |
|  |  | INS |
|  |  | ANAPC2 |
|  |  | WNT11 |
|  |  | ALOX15B |
|  |  | DHDH |
|  |  | CDC20 |
|  |  | DNAJA3 |
|  |  | ROCK1 |
|  |  | COL1A1 |
|  |  | NR1D1 |
|  |  | ABCA1 |
|  |  | NAPRT |
|  |  | MECOM |
|  |  | ABHD5 |
|  |  | SNCA |
|  |  | ADIPOQ |
|  |  | CPLX2 |
|  |  | PPARA |
|  |  | ALOX15 |
|  |  | ANXA1 |
|  |  | PAWR |
|  |  | AKR1B10 |
|  |  | S100A9 |
|  |  | FABP3 |
|  |  | ABCG1 |
|  |  | PRDM16 |
|  |  | CCM2L |
|  |  | ACAT1 |
|  |  | PLA2G1B |
|  |  | DDC |
|  |  | SLC7A11 |
|  |  | EPX |
|  |  | TBXAS1 |
|  | 20-Hexadecanoylingenol | PRKCA |
|  |  | PRKCD |
|  |  | PTGER4 |
|  |  | PTGER2 |
|  |  | PTGER3 |
|  |  | CD300A |
|  |  | CHGA |
|  |  | APOC2 |
|  |  | KIF14 |
|  |  | PRKCB |
|  |  | NR3C1 |
|  | Gaultheroside A | TOP2A |
|  |  | TOP2B |
|  | Dauricine | ACHE |
|  |  | CHRNA2 |
|  |  | HTR3A |
|  |  | CHRM2 |
|  |  | HTR2A |
|  |  | CHRM3 |
|  |  | HTR2B |
|  |  | ADRA2A |
|  |  | HTR1B |
|  |  | HTR2C |
|  |  | HTR1D |
|  |  | DRD2 |
|  |  | SLC18A2 |
|  |  | DRD4 |
|  |  | HTR1A |
|  |  | ADRA2C |
|  |  | CALY |
|  |  | DRD3 |
|  |  | DRD5 |
|  |  | DRD1 |
|  |  | BCHE |
|  |  | ADRA2B |
|  |  | ESR2 |
|  |  | OPRD1 |
|  |  | ESR1 |
|  |  | OPRK1 |
|  |  | OPRM1 |
|  | Gastrodin |  |
|  | Bis(4-Hydroxybenzyl) Ether | TYR |
|  | 4-Ethoxymethylphenyl-4'-Hydroxybenzylether | TYR |
|  |  | SEC14L3 |
|  |  | PPP2CA |
|  |  | PRKCA |
|  |  | NR1I2 |
|  |  | ALOX5 |
|  |  | PPP2CB |
|  |  | SEC14L2 |
|  |  | DGKA |
|  |  | PRKCB |
|  |  | SEC14L4 |
|  | Vanillin | TYR |
|  | Ethoxysanguinarine |  |
|  | Vanillyl Alcohol | TYR |
|  |  | LPL |
|  |  | PDXK |
|  | Sucrose | CXCR4 |
|  |  | NFKB2 |
|  |  | TNF |
|  |  | MMP9 |
|  |  | IFNG |
|  | Vanillin Acetate |  |
|  | Citronellal | ACHE |
|  |  | BCHE |
|  |  | COLQ |
|  |  | F12 |
|  |  | SLC5A7 |
|  |  | NRG1 |
|  |  | DMGDH |
|  |  | SIX3 |
|  |  | CRP |
|  |  | PRSS12 |
|  |  | SLC44A4 |
|  |  | ALDH7A1 |
|  |  | DNM3 |
|  |  | ASCL1 |
|  |  | ENPP6 |
|  |  | CDH8 |
|  |  | FNTA |
|  |  | AGRN |
|  |  | CHKA |
|  |  | CHDH |
|  |  | GRIN1 |
|  | Daucosterol |  |
|  | 4-Hydroxybenzylamine | OPRK1 |
|  |  | OPRM1 |
|  |  | WLS |
|  |  | SLC6A3 |
|  |  | DRD2 |
|  |  | SLC6A4 |
|  |  | DRD4 |
|  |  | HTR7 |
|  |  | HTR1A |
|  |  | DBH |
|  |  | DRD3 |
|  |  | DRD5 |
|  |  | DRD1 |
|  |  | SLC6A2 |
|  |  | ACTN3 |
|  |  | TAC1 |
|  |  | MC2R |
|  |  | CCL5 |
|  |  | PPP1R9B |
|  |  | MC1R |
|  |  | MC4R |
|  |  | FGF10 |
|  |  | EGR1 |
|  |  | VCP |
|  |  | AIF1 |
|  |  | HIF1A |
|  |  | BNIP3 |
|  |  | CRH |
|  |  | NEFL |
|  |  | UCN2 |
|  |  | TACR2 |
|  |  | MC5R |
|  |  | AQP1 |
|  |  | MC3R |
|  |  | TAC4 |
|  |  | CBFA2T3 |
|  |  | NEFH |
|  |  | CRHR2 |
| PS[1] | Luteolin | NOX4 |
|  |  | AKR1B1 |
|  |  | CDK5R1 |
|  |  | XDH |
|  |  | MAOA |
|  |  | FLT3 |
|  |  | CA2 |
|  |  | CCNB3 |
|  |  | ALOX5 |
|  |  | ADORA1 |
|  |  | CA7 |
|  |  | GLO1 |
|  |  | APP |
|  |  | SYK |
|  |  | GSK3B |
|  |  | PARP1 |
|  |  | TTR |
|  |  | MMP9 |
|  |  | CA12 |
|  |  | MMP2 |
|  |  | CA4 |
|  |  | MMP12 |
|  |  | CD38 |
|  |  | CYP1B1 |
|  |  | ABCG2 |
|  |  | AKR1B10 |
|  |  | TNKS2 |
|  |  | TNKS |
|  |  | TOP1 |
|  |  | ARG1 |
|  |  | CDK5 |
|  |  | CDK1 |
|  |  | CCNB1 |
|  |  | CCNB2 |
|  |  | PTPRS |
|  |  | ABCC1 |
|  |  | HSD17B1 |
|  |  | ACHE |
|  |  | CDK6 |
|  |  | ABCB1 |
|  | Catechol | CA2 |
|  |  | CA12 |
|  |  | CA5B |
|  | Phloroglucinol |  |
|  | Pyrogallol | FYN |
|  |  | EGFR |
|  |  | CA2 |
|  |  | CA1 |
|  |  | CA6 |
|  |  | NQO2 |
|  |  | CA12 |
|  |  | CA5B |
|  |  | CA5A |
|  |  | PTPN22 |
|  |  | CA4 |
|  | Quercetin | NOX4 |
|  |  | AVPR2 |
|  |  | AKR1B1 |
|  |  | XDH |
|  |  | MAOA |
|  |  | IGF1R |
|  |  | FLT3 |
|  |  | CYP19A1 |
|  |  | EGFR |
|  |  | F2 |
|  |  | CA2 |
|  |  | PIM1 |
|  |  | ALOX5 |
|  |  | AURKB |
|  |  | DRD4 |
|  |  | ADORA1 |
|  |  | CA7 |
|  |  | GLO1 |
|  |  | MPO |
|  |  | PIK3R1 |
|  |  | ADORA2A |
|  |  | DAPK1 |
|  |  | PYGL |
|  |  | CA1 |
|  |  | GSK3B |
|  |  | SRC |
|  |  | PTK2 |
|  |  | HSD17B2 |
|  |  | KDR |
|  |  | MMP13 |
|  |  | MMP3 |
|  |  | CA3 |
|  |  | ALOX15 |
|  |  | ABCC1 |
|  |  | PLK1 |
|  |  | CA6 |
|  |  | CDK1 |
|  |  | MMP9 |
|  |  | CA12 |
|  |  | MMP2 |
|  |  | PKN1 |
|  |  | CA14 |
|  |  | CA9 |
|  |  | CSNK2A1 |
|  |  | ALOX12 |
|  |  | MET |
|  |  | CA4 |
|  |  | NEK2 |
|  |  | CXCR1 |
|  |  | CAMK2B |
|  |  | ALK |
|  |  | AKT1 |
|  |  | ABCB1 |
|  |  | NEK6 |
|  |  | PLA2G1B |
|  |  | CA5A |
|  |  | BACE1 |
|  |  | CYP1B1 |
|  |  | AXL |
|  |  | ABCG2 |
|  |  | NUAK1 |
|  |  | AKR1C2 |
|  |  | AKR1C1 |
|  |  | AKR1C3 |
|  |  | AKR1C4 |
|  |  | CA13 |
|  |  | AKR1A1 |
|  |  | GPR35 |
|  |  | SYK |
|  |  | MAPT |
|  |  | KDM4E |
|  |  | TOP2A |
|  |  | INSR |
|  |  | ACHE |
|  |  | MYLK |
|  |  | PIK3CG |
|  |  | APEX1 |
|  |  | CDK5R1 CDK5 |
|  |  | CCNB3 CDK1 CCNB1 CCNB2 |
|  |  | ARG1 |
|  |  | PTPRS |
|  |  | ESR2 |
|  |  | MPG |
|  |  | SLC22A12 |
|  |  | CDK6 |
|  |  | CDK2 |

Table S2. Basic information of VaD related targets

| Num | Gene Symbol | Description | Relevance score |
| --- | --- | --- | --- |
| 1 | APOE | Apolipoprotein E | 92.07 |
| 2 | MAPT | Microtubule Associated Protein Tau | 89.83 |
| 3 | VEGFA | Vascular Endothelial Growth Factor A | 82.37 |
| 4 | APP | Amyloid Beta Precursor Protein | 77.83 |
| 5 | PSEN1 | Presenilin 1 | 72.49 |
| 6 | VCP | Valosin Containing Protein | 71.95 |
| 7 | SNCA | Synuclein Alpha | 70.49 |
| 8 | C9orf72 | C9orf72-SMCR8 Complex Subunit | 66.36 |
| 9 | TARDBP | TAR DNA Binding Protein | 64.27 |
| 10 | GRN | Granulin Precursor | 63.21 |
| 11 | SOD1 | Superoxide Dismutase 1 | 62.15 |
| 12 | SQSTM1 | Sequestosome 1 | 59.08 |
| 13 | PRNP | Prion Protein | 58.76 |
| 14 | ACE | Angiotensin I Converting Enzyme | 57.75 |
| 15 | PSEN2 | Presenilin 2 | 55.31 |
| 16 | IL6 | Interleukin 6 | 53.87 |
| 17 | CHMP2B | Charged Multivesicular Body Protein 2B | 53.51 |
| 18 | CST3 | Cystatin C | 52.63 |
| 19 | NOTCH3 | Notch Receptor 3 | 51.53 |
| 20 | ALB | Albumin | 51.41 |
| 21 | NOS3 | Nitric Oxide Synthase 3 | 50.54 |
| 22 | FUS | FUS RNA Binding Protein | 50.03 |
| 23 | LRRK2 | Leucine Rich Repeat Kinase 2 | 49.93 |
| 24 | GFAP | Glial Fibrillary Acidic Protein | 49.81 |
| 25 | SORL1 | Sortilin Related Receptor 1 | 49.66 |
| 26 | TREM2 | Triggering Receptor Expressed On Myeloid Cells 2 | 48.8 |
| 27 | GBA | Glucosylceramidase Beta | 48.48 |
| 28 | PDGFB | Platelet Derived Growth Factor Subunit B | 47.74 |
| 29 | TBK1 | TANK Binding Kinase 1 | 47.42 |
| 30 | PON1 | Paraoxonase 1 | 47.39 |
| 31 | PRKN | Parkin RBR E3 Ubiquitin Protein Ligase | 47.24 |
| 32 | TMEM106B | Transmembrane Protein 106B | 45 |
| 33 | ABCA7 | ATP Binding Cassette Subfamily A Member 7 | 44.76 |
| 34 | UBQLN2 | Ubiquilin 2 | 44.55 |
| 35 | VWF | Von Willebrand Factor | 43.92 |
| 36 | CHCHD10 | Coiled-Coil-Helix-Coiled-Coil-Helix Domain Containing 10 | 43.65 |
| 37 | HNRNPA2B1 | Heterogeneous Nuclear Ribonucleoprotein A2/B1 | 43.16 |
| 38 | SNCB | Synuclein Beta | 43.05 |
| 39 | HTRA1 | HtrA Serine Peptidase 1 | 42.99 |
| 40 | AKT1 | AKT Serine/Threonine Kinase 1 | 42.89 |
| 41 | ITM2B | Integral Membrane Protein 2B | 42.88 |
| 42 | MTHFR | Methylenetetrahydrofolate Reductase | 42.85 |
| 43 | TNF | Tumor Necrosis Factor | 42.85 |
| 44 | PINK1 | PTEN Induced Kinase 1 | 42.7 |
| 45 | CSF1R | Colony Stimulating Factor 1 Receptor | 42.59 |
| 46 | TTR | Transthyretin | 42.39 |
| 47 | COMT | Catechol-O-Methyltransferase | 42.21 |
| 48 | PIK3CA | Phosphatidylinositol-4,5-Bisphosphate 3-Kinase Catalytic Subunit Alpha | 42.07 |
| 49 | TP53 | Tumor Protein P53 | 41.65 |
| 50 | PARK7 | Parkinsonism Associated Deglycase | 41.62 |
| 51 | MT-ND1 | Mitochondrially Encoded NADH:Ubiquinone Oxidoreductase Core Subunit 1 | 41.31 |
| 52 | CTSD | Cathepsin D | 41.29 |
| 53 | UCHL1 | Ubiquitin C-Terminal Hydrolase L1 | 41.18 |
| 54 | HTR2A | 5-Hydroxytryptamine Receptor 2A | 40.75 |
| 55 | HTT | Huntingtin | 40.75 |
| 56 | NEFL | Neurofilament Light | 40.51 |
| 57 | IL10 | Interleukin 10 | 39.9 |
| 58 | KDR | Kinase Insert Domain Receptor | 39.51 |
| 59 | ATP13A2 | ATPase Cation Transporting 13A2 | 39.48 |
| 60 | HNRNPA1 | Heterogeneous Nuclear Ribonucleoprotein A1 | 39.32 |
| 61 | PDGFRB | Platelet Derived Growth Factor Receptor Beta | 38.74 |
| 62 | TUBA4A | Tubulin Alpha 4a | 38.33 |
| 63 | ATXN2 | Ataxin 2 | 38.3 |
| 64 | SERPINI1 | Serpin Family I Member 1 | 37.81 |
| 65 | ARSA | Arylsulfatase A | 37.58 |
| 66 | NPC1 | NPC Intracellular Cholesterol Transporter 1 | 37.39 |
| 67 | DNMT1 | DNA Methyltransferase 1 | 37.36 |
| 68 | SLC6A4 | Solute Carrier Family 6 Member 4 | 36.93 |
| 69 | MME | Membrane Metalloendopeptidase | 36.34 |
| 70 | CP | Ceruloplasmin | 35.93 |
| 71 | NOTCH1 | Notch Receptor 1 | 34.11 |
| 72 | ATXN3 | Ataxin 3 | 34.01 |
| 73 | GNAQ | G Protein Subunit Alpha Q | 33.98 |
| 74 | SNCAIP | Synuclein Alpha Interacting Protein | 33.98 |
| 75 | MT-ATP6 | Mitochondrially Encoded ATP Synthase Membrane Subunit 6 | 33.85 |
| 76 | ATN1 | Atrophin 1 | 33.85 |
| 77 | COL4A1 | Collagen Type IV Alpha 1 Chain | 33.51 |
| 78 | F2 | Coagulation Factor II, Thrombin | 33.25 |
| 79 | NPC2 | NPC Intracellular Cholesterol Transporter 2 | 33.04 |
| 80 | TYROBP | Transmembrane Immune Signaling Adaptor TYROBP | 33.04 |
| 81 | SOD2 | Superoxide Dismutase 2 | 32.82 |
| 82 | VCAM1 | Vascular Cell Adhesion Molecule 1 | 32.54 |
| 83 | MTOR | Mechanistic Target Of Rapamycin Kinase | 32.45 |
| 84 | SERPINA3 | Serpin Family A Member 3 | 32.28 |
| 85 | MPO | Myeloperoxidase | 32 |
| 86 | MIR132 | MicroRNA 132 | 31.86 |
| 87 | TRPM7 | Transient Receptor Potential Cation Channel Subfamily M Member 7 | 31.65 |
| 88 | JPH3 | Junctophilin 3 | 31.26 |
| 89 | IL1B | Interleukin 1 Beta | 31.11 |
| 90 | MFSD8 | Major Facilitator Superfamily Domain Containing 8 | 31.09 |
| 91 | TBP | TATA-Box Binding Protein | 31.02 |
| 92 | PLG | Plasminogen | 30.79 |
| 93 | FMR1 | FMRP Translational Regulator 1 | 30.77 |
| 94 | LDLR | Low Density Lipoprotein Receptor | 30.28 |
| 95 | LPL | Lipoprotein Lipase | 30.17 |
| 96 | CTNNB1 | Catenin Beta 1 | 30.06 |
| 97 | ATXN1 | Ataxin 1 | 30.04 |
| 98 | PDCD10 | Programmed Cell Death 10 | 29.38 |
| 99 | FGF2 | Fibroblast Growth Factor 2 | 29.29 |
| 100 | ACHE | Acetylcholinesterase (Cartwright Blood Group) | 29.19 |
| 101 | PLA2G6 | Phospholipase A2 Group VI | 29.03 |
| 102 | PLAU | Plasminogen Activator, Urokinase | 28.94 |
| 103 | F5 | Coagulation Factor V | 28.83 |
| 104 | NEFH | Neurofilament Heavy | 28.79 |
| 105 | MT-ND4 | Mitochondrially Encoded NADH:Ubiquinone Oxidoreductase Core Subunit 4 | 28.75 |
| 106 | MIR146A | MicroRNA 146a | 28.72 |
| 107 | CSTB | Cystatin B | 28.72 |
| 108 | ELN | Elastin | 28.49 |
| 109 | MIR659 | MicroRNA 659 | 28.37 |
| 110 | RPS27A | Ribosomal Protein S27a | 28.2 |
| 111 | KRAS | KRAS Proto-Oncogene, GTPase | 28.07 |
| 112 | HFE | Homeostatic Iron Regulator | 27.93 |
| 113 | SPG21 | SPG21 Abhydrolase Domain Containing, Maspardin | 27.88 |
| 114 | MTR | 5-Methyltetrahydrofolate-Homocysteine Methyltransferase | 27.68 |
| 115 | BDNF | Brain Derived Neurotrophic Factor | 27.64 |
| 116 | SCARB2 | Scavenger Receptor Class B Member 2 | 27.6 |
| 117 | CLN5 | CLN5 Intracellular Trafficking Protein | 27.49 |
| 118 | TREX1 | Three Prime Repair Exonuclease 1 | 27.29 |
| 119 | HRAS | HRas Proto-Oncogene, GTPase | 27.18 |
| 120 | ICAM1 | Intercellular Adhesion Molecule 1 | 27.15 |
| 121 | MT-CO1 | Mitochondrially Encoded Cytochrome C Oxidase I | 26.71 |
| 122 | AGER | Advanced Glycosylation End-Product Specific Receptor | 26.49 |
| 123 | TOMM40 | Translocase Of Outer Mitochondrial Membrane 40 | 26.36 |
| 124 | NHLRC1 | NHL Repeat Containing E3 Ubiquitin Protein Ligase 1 | 26.21 |
| 125 | CRP | C-Reactive Protein | 26.11 |
| 126 | ADAM10 | ADAM Metallopeptidase Domain 10 | 26.1 |
| 127 | EPM2A | EPM2A Glucan Phosphatase, Laforin | 26.1 |
| 128 | BCHE | Butyrylcholinesterase | 25.87 |
| 129 | CAV1 | Caveolin 1 | 25.83 |
| 130 | PTEN | Phosphatase And Tensin Homolog | 25.73 |
| 131 | MIR21 | MicroRNA 21 | 25.7 |
| 132 | LRP5 | LDL Receptor Related Protein 5 | 25.21 |
| 133 | MT-CO2 | Mitochondrially Encoded Cytochrome C Oxidase II | 25.2 |
| 134 | CHAT | Choline O-Acetyltransferase | 25.08 |
| 135 | JAG1 | Jagged Canonical Notch Ligand 1 | 25.05 |
| 136 | MIR29B1 | MicroRNA 29b-1 | 24.88 |
| 137 | DLL4 | Delta Like Canonical Notch Ligand 4 | 24.74 |
| 138 | INS | Insulin | 24.63 |
| 139 | CASP3 | Caspase 3 | 24.51 |
| 140 | ENG | Endoglin | 24.46 |
| 141 | DCTN1 | Dynactin Subunit 1 | 24.38 |
| 142 | SYP | Synaptophysin | 24.22 |
| 143 | GLA | Galactosidase Alpha | 24.08 |
| 144 | MMP9 | Matrix Metallopeptidase 9 | 23.78 |
| 145 | PSAP | Prosaposin | 23.54 |
| 146 | ECE1 | Endothelin Converting Enzyme 1 | 23.51 |
| 147 | NOS2 | Nitric Oxide Synthase 2 | 23.48 |
| 148 | SDHD | Succinate Dehydrogenase Complex Subunit D | 23.27 |
| 149 | TH | Tyrosine Hydroxylase | 23.24 |
| 150 | MAPK1 | Mitogen-Activated Protein Kinase 1 | 23.16 |
| 151 | MT-ND2 | Mitochondrially Encoded NADH:Ubiquinone Oxidoreductase Core Subunit 2 | 22.81 |
| 152 | PTGS2 | Prostaglandin-Endoperoxide Synthase 2 | 22.79 |
| 153 | DRD3 | Dopamine Receptor D3 | 22.77 |
| 154 | CAT | Catalase | 22.77 |
| 155 | NOTCH2 | Notch Receptor 2 | 22.7 |
| 156 | MAPK14 | Mitogen-Activated Protein Kinase 14 | 22.61 |
| 157 | PPARG | Peroxisome Proliferator Activated Receptor Gamma | 22.37 |
| 158 | IGF1 | Insulin Like Growth Factor 1 | 22.34 |
| 159 | CLN6 | CLN6 Transmembrane ER Protein | 22.32 |
| 160 | CCL2 | C-C Motif Chemokine Ligand 2 | 22.28 |
| 161 | COL4A2 | Collagen Type IV Alpha 2 Chain | 22.2 |
| 162 | NGF | Nerve Growth Factor | 22.12 |
| 163 | SLC6A3 | Solute Carrier Family 6 Member 3 | 22.12 |
| 164 | ERCC6 | ERCC Excision Repair 6, Chromatin Remodeling Factor | 22.01 |
| 165 | NDP | Norrin Cystine Knot Growth Factor NDP | 21.92 |
| 166 | PECAM1 | Platelet And Endothelial Cell Adhesion Molecule 1 | 21.85 |
| 167 | NEK1 | NIMA Related Kinase 1 | 21.82 |
| 168 | CXCR4 | C-X-C Motif Chemokine Receptor 4 | 21.8 |
| 169 | TYMP | Thymidine Phosphorylase | 21.78 |
| 170 | MAOB | Monoamine Oxidase B | 21.77 |
| 171 | ITGAM | Integrin Subunit Alpha M | 21.28 |
| 172 | PON2 | Paraoxonase 2 | 21.17 |
| 173 | PRODH | Proline Dehydrogenase 1 | 21.17 |
| 174 | A2M | Alpha-2-Macroglobulin | 21.05 |
| 175 | BACE1 | Beta-Secretase 1 | 21.02 |
| 176 | CBS | Cystathionine Beta-Synthase | 20.94 |
| 177 | MT-CYB | Mitochondrially Encoded Cytochrome B | 20.93 |
| 178 | SELE | Selectin E | 20.79 |
| 179 | CYP2D6 | Cytochrome P450 Family 2 Subfamily D Member 6 | 20.74 |
| 180 | ERBB4 | Erb-B2 Receptor Tyrosine Kinase 4 | 20.62 |
| 181 | IL1A | Interleukin 1 Alpha | 20.59 |
| 182 | DNM1L | Dynamin 1 Like | 20.54 |
| 183 | PANK2 | Pantothenate Kinase 2 | 20.53 |
| 184 | FTL | Ferritin Light Chain | 20.51 |
| 185 | TGFBR2 | Transforming Growth Factor Beta Receptor 2 | 20.46 |
| 186 | SDHB | Succinate Dehydrogenase Complex Iron Sulfur Subunit B | 20.43 |
| 187 | APOA1 | Apolipoprotein A1 | 20.4 |
| 188 | SPTLC1 | Serine Palmitoyltransferase Long Chain Base Subunit 1 | 20.36 |
| 189 | SELP | Selectin P | 20.35 |
| 190 | CAMK2G | Calcium/Calmodulin Dependent Protein Kinase II Gamma | 20.34 |
| 191 | CLN3 | CLN3 Lysosomal/Endosomal Transmembrane Protein, Battenin | 20.27 |
| 192 | OPTN | Optineurin | 20.26 |
| 193 | MIR34A | MicroRNA 34a | 20.25 |
| 194 | CACNA1A | Calcium Voltage-Gated Channel Subunit Alpha1 A | 20.21 |
| 195 | SETX | Senataxin | 20.2 |
| 196 | HMOX1 | Heme Oxygenase 1 | 20.16 |
| 197 | RBPJ | Recombination Signal Binding Protein For Immunoglobulin Kappa J Region | 20.16 |
| 198 | MT-TK | Mitochondrially Encoded TRNA-Lys (AAA/G) | 19.98 |
| 199 | SERPINE1 | Serpin Family E Member 1 | 19.97 |
| 200 | LRP1 | LDL Receptor Related Protein 1 | 19.95 |
| 201 | CHI3L1 | Chitinase 3 Like 1 | 19.9 |
| 202 | GSK3B | Glycogen Synthase Kinase 3 Beta | 19.87 |
| 203 | XPR1 | Xenotropic And Polytropic Retrovirus Receptor 1 | 19.79 |
| 204 | MIR29A | MicroRNA 29a | 19.7 |
| 205 | SNCG | Synuclein Gamma | 19.63 |
| 206 | FBN1 | Fibrillin 1 | 19.6 |
| 207 | ANG | Angiogenin | 19.56 |
| 208 | APOB | Apolipoprotein B | 19.52 |
| 209 | MT-ND6 | Mitochondrially Encoded NADH:Ubiquinone Oxidoreductase Core Subunit 6 | 19.51 |
| 210 | IFNG | Interferon Gamma | 19.48 |
| 211 | OLR1 | Oxidized Low Density Lipoprotein Receptor 1 | 19.46 |
| 212 | DRD2 | Dopamine Receptor D2 | 19.36 |
| 213 | MAP2 | Microtubule Associated Protein 2 | 19.36 |
| 214 | CLU | Clusterin | 19.25 |
| 215 | PLAT | Plasminogen Activator, Tissue Type | 19.24 |
| 216 | HCRT | Hypocretin Neuropeptide Precursor | 19.23 |
| 217 | SERPINC1 | Serpin Family C Member 1 | 19.18 |
| 218 | THBD | Thrombomodulin | 19.16 |
| 219 | SPTLC2 | Serine Palmitoyltransferase Long Chain Base Subunit 2 | 19.1 |
| 220 | UNC5C | Unc-5 Netrin Receptor C | 19.01 |
| 221 | LOX | Lysyl Oxidase | 18.96 |
| 222 | HTR1A | 5-Hydroxytryptamine Receptor 1A | 18.94 |
| 223 | GAPDH | Glyceraldehyde-3-Phosphate Dehydrogenase | 18.94 |
| 224 | NRP1 | Neuropilin 1 | 18.91 |
| 225 | NF1 | Neurofibromin 1 | 18.86 |
| 226 | EGF | Epidermal Growth Factor | 18.81 |
| 227 | ATXN7 | Ataxin 7 | 18.77 |
| 228 | AOC3 | Amine Oxidase Copper Containing 3 | 18.73 |
| 229 | SST | Somatostatin | 18.57 |
| 230 | EDN1 | Endothelin 1 | 18.54 |
| 231 | SAG | S-Antigen Visual Arrestin | 18.52 |
| 232 | SLC20A2 | Solute Carrier Family 20 Member 2 | 18.44 |
| 233 | PITX2 | Paired Like Homeodomain 2 | 18.43 |
| 234 | MIR106B | MicroRNA 106b | 18.38 |
| 235 | CD34 | CD34 Molecule | 18.36 |
| 236 | PCSK1N | Proprotein Convertase Subtilisin/Kexin Type 1 Inhibitor | 18.34 |
| 237 | AIF1 | Allograft Inflammatory Factor 1 | 18.24 |
| 238 | DNAJC5 | DnaJ Heat Shock Protein Family (Hsp40) Member C5 | 18.23 |
| 239 | CD40LG | CD40 Ligand | 18.22 |
| 240 | TSPO | Translocator Protein | 18.21 |
| 241 | CXCL8 | C-X-C Motif Chemokine Ligand 8 | 18.19 |
| 242 | PNPLA6 | Patatin Like Phospholipase Domain Containing 6 | 17.99 |
| 243 | MIR30E | MicroRNA 30e | 17.99 |
| 244 | OGG1 | 8-Oxoguanine DNA Glycosylase | 17.98 |
| 245 | TGFB2 | Transforming Growth Factor Beta 2 | 17.97 |
| 246 | FBLN5 | Fibulin 5 | 17.92 |
| 247 | TLR4 | Toll Like Receptor 4 | 17.91 |
| 248 | TGFB1 | Transforming Growth Factor Beta 1 | 17.91 |
| 249 | MT-ND5 | Mitochondrially Encoded NADH:Ubiquinone Oxidoreductase Core Subunit 5 | 17.87 |
| 250 | VPS13A | Vacuolar Protein Sorting 13 Homolog A | 17.78 |
| 251 | MIR143 | MicroRNA 143 | 17.78 |
| 252 | HMGCR | 3-Hydroxy-3-Methylglutaryl-CoA Reductase | 17.73 |
| 253 | POLG | DNA Polymerase Gamma, Catalytic Subunit | 17.67 |
| 254 | SMPD1 | Sphingomyelin Phosphodiesterase 1 | 17.65 |
| 255 | RETREG1 | Reticulophagy Regulator 1 | 17.65 |
| 256 | MT-TL1 | Mitochondrially Encoded TRNA-Leu (UUA/G) 1 | 17.51 |
| 257 | LEP | Leptin | 17.5 |
| 258 | SLC2A1 | Solute Carrier Family 2 Member 1 | 17.5 |
| 259 | ATP7A | ATPase Copper Transporting Alpha | 17.49 |
| 260 | LOC109504728 | Chromosome 9 Open Reading Frame 72 Repeat Instability Region | 17.47 |
| 261 | HSPA4 | Heat Shock Protein Family A (Hsp70) Member 4 | 17.44 |
| 262 | BPTF | Bromodomain PHD Finger Transcription Factor | 17.41 |
| 263 | MIR107 | MicroRNA 107 | 17.38 |
| 264 | NPY | Neuropeptide Y | 17.35 |
| 265 | ABCA1 | ATP Binding Cassette Subfamily A Member 1 | 17.33 |
| 266 | ALDH18A1 | Aldehyde Dehydrogenase 18 Family Member A1 | 17.32 |
| 267 | FN1 | Fibronectin 1 | 17.31 |
| 268 | CDH5 | Cadherin 5 | 17.14 |
| 269 | F3 | Coagulation Factor III, Tissue Factor | 17.13 |
| 270 | FIG4 | FIG4 Phosphoinositide 5-Phosphatase | 17.12 |
| 271 | CETP | Cholesteryl Ester Transfer Protein | 17.03 |
| 272 | CDK5 | Cyclin Dependent Kinase 5 | 16.99 |
| 273 | KNG1 | Kininogen 1 | 16.94 |
| 274 | HIF1A | Hypoxia Inducible Factor 1 Subunit Alpha | 16.93 |
| 275 | ADIPOQ | Adiponectin, C1Q And Collagen Domain Containing | 16.9 |
| 276 | CHRNA7 | Cholinergic Receptor Nicotinic Alpha 7 Subunit | 16.9 |
| 277 | SCN1A | Sodium Voltage-Gated Channel Alpha Subunit 1 | 16.87 |
| 278 | JAK2 | Janus Kinase 2 | 16.87 |
| 279 | AGT | Angiotensinogen | 16.85 |
| 280 | TIMP1 | TIMP Metallopeptidase Inhibitor 1 | 16.83 |
| 281 | GNA14 | G Protein Subunit Alpha 14 | 16.83 |
| 282 | PAX6 | Paired Box 6 | 16.81 |
| 283 | ATP6V0A2 | ATPase H+ Transporting V0 Subunit A2 | 16.73 |
| 284 | FGFR1 | Fibroblast Growth Factor Receptor 1 | 16.67 |
| 285 | TPP1 | Tripeptidyl Peptidase 1 | 16.63 |
| 286 | PTGS1 | Prostaglandin-Endoperoxide Synthase 1 | 16.61 |
| 287 | MAPK8 | Mitogen-Activated Protein Kinase 8 | 16.6 |
| 288 | JUN | Jun Proto-Oncogene, AP-1 Transcription Factor Subunit | 16.6 |
| 289 | IDE | Insulin Degrading Enzyme | 16.57 |
| 290 | CASP8 | Caspase 8 | 16.55 |
| 291 | MTRR | 5-Methyltetrahydrofolate-Homocysteine Methyltransferase Reductase | 16.53 |
| 292 | MAPK3 | Mitogen-Activated Protein Kinase 3 | 16.52 |
| 293 | AGTR1 | Angiotensin II Receptor Type 1 | 16.46 |
| 294 | MT-CO3 | Mitochondrially Encoded Cytochrome C Oxidase III | 16.44 |
| 295 | STXBP1 | Syntaxin Binding Protein 1 | 16.34 |
| 296 | ACTC1 | Actin Alpha Cardiac Muscle 1 | 16.32 |
| 297 | BMP6 | Bone Morphogenetic Protein 6 | 16.32 |
| 298 | CHRM1 | Cholinergic Receptor Muscarinic 1 | 16.31 |
| 299 | CSF1 | Colony Stimulating Factor 1 | 16.23 |
| 300 | MECP2 | Methyl-CpG Binding Protein 2 | 16.17 |
| 301 | NPPB | Natriuretic Peptide B | 16.16 |
| 302 | MASP2 | Mannan Binding Lectin Serine Peptidase 2 | 16.11 |
| 303 | CCR5 | C-C Motif Chemokine Receptor 5 | 16.1 |
| 304 | CD4 | CD4 Molecule | 16.06 |
| 305 | AD10 | Alzheimer Disease-10 | 16.05 |
| 306 | AD6 | Alzheimer Disease 6 | 16.05 |
| 307 | DBN1 | Drebrin 1 | 15.98 |
| 308 | PPT1 | Palmitoyl-Protein Thioesterase 1 | 15.97 |
| 309 | MIR328 | MicroRNA 328 | 15.94 |
| 310 | AD5 | Alzheimer Disease 5 | 15.89 |
| 311 | MIR298 | MicroRNA 298 | 15.84 |
| 312 | AD11 | Alzheimer Disease-11 | 15.84 |
| 313 | AD12 | Alzheimer Disease 12 | 15.84 |
| 314 | AD13 | Alzheimer Disease-13 | 15.84 |
| 315 | AD14 | Alzheimer Disease 14 | 15.84 |
| 316 | AD15 | Alzheimer Disease-15 | 15.84 |
| 317 | AD16 | Alzheimer Disease 16 | 15.84 |
| 318 | AD17 | Alzheimer Disease 17 | 15.84 |
| 319 | AD7 | Alzheimer Disease 7 | 15.84 |
| 320 | AD8 | Alzheimer Disease 8 | 15.84 |
| 321 | ZFYVE26 | Zinc Finger FYVE-Type Containing 26 | 15.82 |
| 322 | HEXA | Hexosaminidase Subunit Alpha | 15.82 |
| 323 | GDNF | Glial Cell Derived Neurotrophic Factor | 15.76 |
| 324 | NOX1 | NADPH Oxidase 1 | 15.76 |
| 325 | MIR144 | MicroRNA 144 | 15.72 |
| 326 | CRH | Corticotropin Releasing Hormone | 15.7 |
| 327 | PPARGC1A | PPARG Coactivator 1 Alpha | 15.68 |
| 328 | ATP7B | ATPase Copper Transporting Beta | 15.67 |
| 329 | MYORG | Myogenesis Regulating Glycosidase (Putative) | 15.66 |
| 330 | MT-TF | Mitochondrially Encoded TRNA-Phe (UUU/C) | 15.66 |
| 331 | ITIH4 | Inter-Alpha-Trypsin Inhibitor Heavy Chain 4 | 15.64 |
| 332 | GRIA3 | Glutamate Ionotropic Receptor AMPA Type Subunit 3 | 15.64 |
| 333 | APLNR | Apelin Receptor | 15.55 |
| 334 | NOS1 | Nitric Oxide Synthase 1 | 15.45 |
| 335 | CREB1 | CAMP Responsive Element Binding Protein 1 | 15.37 |
| 336 | MAOA | Monoamine Oxidase A | 15.33 |
| 337 | CDKN3 | Cyclin Dependent Kinase Inhibitor 3 | 15.3 |
| 338 | FZD4 | Frizzled Class Receptor 4 | 15.27 |
| 339 | PIK3R1 | Phosphoinositide-3-Kinase Regulatory Subunit 1 | 15.25 |
| 340 | PRDM10 | PR/SET Domain 10 | 15.22 |
| 341 | CXCL12 | C-X-C Motif Chemokine Ligand 12 | 15.22 |
| 342 | FAS | Fas Cell Surface Death Receptor | 15.21 |
| 343 | HLA-DQB1 | Major Histocompatibility Complex, Class II, DQ Beta 1 | 15.21 |
| 344 | ADA2 | Adenosine Deaminase 2 | 15.14 |
| 345 | FLNA | Filamin A | 15.1 |
| 346 | CDK1 | Cyclin Dependent Kinase 1 | 15.1 |
| 347 | MBTPS2 | Membrane Bound Transcription Factor Peptidase, Site 2 | 15.06 |
| 348 | HSPG2 | Heparan Sulfate Proteoglycan 2 | 15.03 |
| 349 | CTSF | Cathepsin F | 14.98 |
| 350 | CRYAB | Crystallin Alpha B | 14.93 |
| 351 | PGF | Placental Growth Factor | 14.92 |
| 352 | PRDM6 | PR/SET Domain 6 | 14.91 |
| 353 | NFKB1 | Nuclear Factor Kappa B Subunit 1 | 14.91 |
| 354 | CDKL5 | Cyclin Dependent Kinase Like 5 | 14.9 |
| 355 | HSP90AA1 | Heat Shock Protein 90 Alpha Family Class A Member 1 | 14.86 |
| 356 | NAGA | Alpha-N-Acetylgalactosaminidase | 14.85 |
| 357 | MYO7A | Myosin VIIA | 14.79 |
| 358 | GSN | Gelsolin | 14.78 |
| 359 | S100B | S100 Calcium Binding Protein B | 14.77 |
| 360 | CREBBP | CREB Binding Protein | 14.74 |
| 361 | IL1RN | Interleukin 1 Receptor Antagonist | 14.73 |
| 362 | DGUOK | Deoxyguanosine Kinase | 14.65 |
| 363 | MAP2K1 | Mitogen-Activated Protein Kinase Kinase 1 | 14.64 |
| 364 | MAPK10 | Mitogen-Activated Protein Kinase 10 | 14.61 |
| 365 | ESR1 | Estrogen Receptor 1 | 14.59 |
| 366 | CBSL | Cystathionine Beta-Synthase Like | 14.59 |
| 367 | PICALM | Phosphatidylinositol Binding Clathrin Assembly Protein | 14.58 |
| 368 | PITX3 | Paired Like Homeodomain 3 | 14.58 |
| 369 | MFN2 | Mitofusin 2 | 14.58 |
| 370 | CSF3 | Colony Stimulating Factor 3 | 14.57 |
| 371 | CYCS | Cytochrome C, Somatic | 14.52 |
| 372 | GJB2 | Gap Junction Protein Beta 2 | 14.39 |
| 373 | MYOCD | Myocardin | 14.36 |
| 374 | CLN8 | CLN8 Transmembrane ER And ERGIC Protein | 14.33 |
| 375 | WFS1 | Wolframin ER Transmembrane Glycoprotein | 14.32 |
| 376 | H2AC18 | H2A Clustered Histone 18 | 14.32 |
| 377 | MT-TS2 | Mitochondrially Encoded TRNA-Ser (AGU/C) 2 | 14.31 |
| 378 | MBP | Myelin Basic Protein | 14.31 |
| 379 | TAGLN | Transgelin | 14.31 |
| 380 | CD40 | CD40 Molecule | 14.27 |
| 381 | SIRT1 | Sirtuin 1 | 14.27 |
| 382 | MMP2 | Matrix Metallopeptidase 2 | 14.26 |
| 383 | RTN4 | Reticulon 4 | 14.26 |
| 384 | KCTD7 | Potassium Channel Tetramerization Domain Containing 7 | 14.25 |
| 385 | TGFB3 | Transforming Growth Factor Beta 3 | 14.25 |
| 386 | SMAD3 | SMAD Family Member 3 | 14.23 |
| 387 | YWHAQ | Tyrosine 3-Monooxygenase/Tryptophan 5-Monooxygenase Activation Protein Theta | 14.21 |
| 388 | PPARA | Peroxisome Proliferator Activated Receptor Alpha | 14.18 |
| 389 | REST | RE1 Silencing Transcription Factor | 14.16 |
| 390 | APOH | Apolipoprotein H | 14.11 |
| 391 | TF | Transferrin | 14.08 |
| 392 | GRIN2B | Glutamate Ionotropic Receptor NMDA Type Subunit 2B | 14.06 |
| 393 | TWNK | Twinkle MtDNA Helicase | 14.04 |
| 394 | PRPH2 | Peripherin 2 | 13.97 |
| 395 | TNFRSF1A | TNF Receptor Superfamily Member 1A | 13.97 |
| 396 | DNM1 | Dynamin 1 | 13.95 |
| 397 | APBB1 | Amyloid Beta Precursor Protein Binding Family B Member 1 | 13.94 |
| 398 | SPP1 | Secreted Phosphoprotein 1 | 13.93 |
| 399 | CNR1 | Cannabinoid Receptor 1 | 13.92 |
| 400 | DHFR | Dihydrofolate Reductase | 13.86 |
| 401 | IL2 | Interleukin 2 | 13.83 |
| 402 | EGFR | Epidermal Growth Factor Receptor | 13.82 |
| 403 | PRKCA | Protein Kinase C Alpha | 13.8 |
| 404 | PVALB | Parvalbumin | 13.79 |
| 405 | RPE65 | Retinoid Isomerohydrolase RPE65 | 13.77 |
| 406 | TSPAN12 | Tetraspanin 12 | 13.77 |
| 407 | SNAP25 | Synaptosome Associated Protein 25 | 13.75 |
| 408 | PRKCB | Protein Kinase C Beta | 13.66 |
| 409 | ESR2 | Estrogen Receptor 2 | 13.64 |
| 410 | DRD4 | Dopamine Receptor D4 | 13.63 |
| 411 | NRGN | Neurogranin | 13.63 |
| 412 | CFH | Complement Factor H | 13.62 |
| 413 | EPO | Erythropoietin | 13.59 |
| 414 | IL4 | Interleukin 4 | 13.58 |
| 415 | RELA | RELA Proto-Oncogene, NF-KB Subunit | 13.58 |
| 416 | RAF1 | Raf-1 Proto-Oncogene, Serine/Threonine Kinase | 13.57 |
| 417 | AQP4 | Aquaporin 4 | 13.52 |
| 418 | MIRLET7I | MicroRNA Let-7i | 13.48 |
| 419 | MIR125A | MicroRNA 125a | 13.47 |
| 420 | EFEMP2 | EGF Containing Fibulin Extracellular Matrix Protein 2 | 13.47 |
| 421 | ADAMTS2 | ADAM Metallopeptidase With Thrombospondin Type 1 Motif 2 | 13.42 |
| 422 | GRIN2A | Glutamate Ionotropic Receptor NMDA Type Subunit 2A | 13.41 |
| 423 | PIN1 | Peptidylprolyl Cis/Trans Isomerase, NIMA-Interacting 1 | 13.32 |
| 424 | MYC | MYC Proto-Oncogene, BHLH Transcription Factor | 13.3 |
| 425 | GSR | Glutathione-Disulfide Reductase | 13.27 |
| 426 | SYK | Spleen Associated Tyrosine Kinase | 13.22 |
| 427 | HSPD1 | Heat Shock Protein Family D (Hsp60) Member 1 | 13.2 |
| 428 | OCLN | Occludin | 13.2 |
| 429 | KIT | KIT Proto-Oncogene, Receptor Tyrosine Kinase | 13.07 |
| 430 | HLA-DRB1 | Major Histocompatibility Complex, Class II, DR Beta 1 | 13.07 |
| 431 | ERCC2 | ERCC Excision Repair 2, TFIIH Core Complex Helicase Subunit | 13.06 |
| 432 | VPS35 | VPS35 Retromer Complex Component | 13.05 |
| 433 | TLR2 | Toll Like Receptor 2 | 13.02 |
| 434 | XBP1 | X-Box Binding Protein 1 | 12.97 |
| 435 | CASP1 | Caspase 1 | 12.89 |
| 436 | NTF3 | Neurotrophin 3 | 12.88 |
| 437 | SLC1A3 | Solute Carrier Family 1 Member 3 | 12.88 |
| 438 | SURF1 | SURF1 Cytochrome C Oxidase Assembly Factor | 12.86 |
| 439 | SARS1 | Seryl-TRNA Synthetase 1 | 12.84 |
| 440 | CTSB | Cathepsin B | 12.84 |
| 441 | VSNL1 | Visinin Like 1 | 12.81 |
| 442 | NAGLU | N-Acetyl-Alpha-Glucosaminidase | 12.79 |
| 443 | APOC1 | Apolipoprotein C1 | 12.78 |
| 444 | SRC | SRC Proto-Oncogene, Non-Receptor Tyrosine Kinase | 12.76 |
| 445 | GLUL | Glutamate-Ammonia Ligase | 12.74 |
| 446 | RHOBTB2 | Rho Related BTB Domain Containing 2 | 12.74 |
| 447 | RELN | Reelin | 12.73 |
| 448 | NFE2L2 | Nuclear Factor, Erythroid 2 Like 2 | 12.73 |
| 449 | IL18 | Interleukin 18 | 12.7 |
| 450 | CALB2 | Calbindin 2 | 12.66 |
| 451 | CCL3 | C-C Motif Chemokine Ligand 3 | 12.63 |
| 452 | EGR1 | Early Growth Response 1 | 12.61 |
| 453 | ATP6V1E1 | ATPase H+ Transporting V1 Subunit E1 | 12.58 |
| 454 | DRD1 | Dopamine Receptor D1 | 12.51 |
| 455 | FYN | FYN Proto-Oncogene, Src Family Tyrosine Kinase | 12.51 |
| 456 | SGSH | N-Sulfoglucosamine Sulfohydrolase | 12.47 |
| 457 | TIA1 | TIA1 Cytotoxic Granule Associated RNA Binding Protein | 12.46 |
| 458 | MIR155 | MicroRNA 155 | 12.44 |
| 459 | PYCR1 | Pyrroline-5-Carboxylate Reductase 1 | 12.43 |
| 460 | CACNA1C | Calcium Voltage-Gated Channel Subunit Alpha1 C | 12.42 |
| 461 | ZNF408 | Zinc Finger Protein 408 | 12.42 |
| 462 | NTRK2 | Neurotrophic Receptor Tyrosine Kinase 2 | 12.41 |
| 463 | ADAM17 | ADAM Metallopeptidase Domain 17 | 12.38 |
| 464 | NTRK1 | Neurotrophic Receptor Tyrosine Kinase 1 | 12.31 |
| 465 | SLC1A2 | Solute Carrier Family 1 Member 2 | 12.3 |
| 466 | TNFRSF11B | TNF Receptor Superfamily Member 11b | 12.3 |
| 467 | TM2D3 | TM2 Domain Containing 3 | 12.26 |
| 468 | SERPINA1 | Serpin Family A Member 1 | 12.22 |
| 469 | HSPA5 | Heat Shock Protein Family A (Hsp70) Member 5 | 12.21 |
| 470 | PRDX1 | Peroxiredoxin 1 | 12.2 |
| 471 | VTN | Vitronectin | 12.19 |
| 472 | NOTCH4 | Notch Receptor 4 | 12.19 |
| 473 | PDGFRA | Platelet Derived Growth Factor Receptor Alpha | 12.18 |
| 474 | CCN2 | Cellular Communication Network Factor 2 | 12.17 |
| 475 | HSPA8 | Heat Shock Protein Family A (Hsp70) Member 8 | 12.13 |
| 476 | MIR126 | MicroRNA 126 | 12.1 |
| 477 | HP | Haptoglobin | 12.09 |
| 478 | MEF2C | Myocyte Enhancer Factor 2C | 12.09 |
| 479 | ADH1C | Alcohol Dehydrogenase 1C (Class I), Gamma Polypeptide | 12.02 |
| 480 | MT-TS1 | Mitochondrially Encoded TRNA-Ser (UCN) 1 | 11.98 |
| 481 | RBFOX3 | RNA Binding Fox-1 Homolog 3 | 11.98 |
| 482 | PTGIS | Prostaglandin I2 Synthase | 11.93 |
| 483 | POU1F1 | POU Class 1 Homeobox 1 | 11.93 |
| 484 | CASP9 | Caspase 9 | 11.93 |
| 485 | UBB | Ubiquitin B | 11.9 |
| 486 | MIR22 | MicroRNA 22 | 11.87 |
| 487 | SP1 | Sp1 Transcription Factor | 11.83 |
| 488 | STH | Saitohin | 11.79 |
| 489 | DLG4 | Discs Large MAGUK Scaffold Protein 4 | 11.76 |
| 490 | MT-TE | Mitochondrially Encoded TRNA-Glu (GAA/G) | 11.76 |
| 491 | APTX | Aprataxin | 11.75 |
| 492 | STAT1 | Signal Transducer And Activator Of Transcription 1 | 11.74 |
| 493 | MMACHC | Metabolism Of Cobalamin Associated C | 11.74 |
| 494 | BIN1 | Bridging Integrator 1 | 11.73 |
| 495 | TRIM9 | Tripartite Motif Containing 9 | 11.7 |
| 496 | NPEPPS | Aminopeptidase Puromycin Sensitive | 11.7 |
| 497 | LPA | Lipoprotein(A) | 11.7 |
| 498 | SLC18A2 | Solute Carrier Family 18 Member A2 | 11.68 |
| 499 | PF4 | Platelet Factor 4 | 11.66 |
| 500 | F8 | Coagulation Factor VIII | 11.65 |
| 501 | IL17A | Interleukin 17A | 11.64 |
| 502 | COX5A | Cytochrome C Oxidase Subunit 5A | 11.62 |
| 503 | INSR | Insulin Receptor | 11.62 |
| 504 | ERCC4 | ERCC Excision Repair 4, Endonuclease Catalytic Subunit | 11.62 |
| 505 | ALOX5 | Arachidonate 5-Lipoxygenase | 11.59 |
| 506 | TNK1 | Tyrosine Kinase Non Receptor 1 | 11.58 |
| 507 | NDUFS4 | NADH:Ubiquinone Oxidoreductase Subunit S4 | 11.58 |
| 508 | VIM | Vimentin | 11.55 |
| 509 | CALB1 | Calbindin 1 | 11.55 |
| 510 | TXN | Thioredoxin | 11.54 |
| 511 | DBH | Dopamine Beta-Hydroxylase | 11.52 |
| 512 | UBE2L3 | Ubiquitin Conjugating Enzyme E2 L3 | 11.52 |
| 513 | GRIN1 | Glutamate Ionotropic Receptor NMDA Type Subunit 1 | 11.49 |
| 514 | VLDLR | Very Low Density Lipoprotein Receptor | 11.49 |
| 515 | CAPN1 | Calpain 1 | 11.48 |
| 516 | MIR210 | MicroRNA 210 | 11.47 |
| 517 | NRXN1 | Neurexin 1 | 11.45 |
| 518 | ITPR1 | Inositol 1,4,5-Trisphosphate Receptor Type 1 | 11.42 |
| 519 | NRG1 | Neuregulin 1 | 11.38 |
| 520 | ATL1 | Atlastin GTPase 1 | 11.37 |
| 521 | TUBB | Tubulin Beta Class I | 11.27 |
| 522 | NGFR | Nerve Growth Factor Receptor | 11.27 |
| 523 | CRYAA | Crystallin Alpha A | 11.26 |
| 524 | CX3CR1 | C-X3-C Motif Chemokine Receptor 1 | 11.25 |
| 525 | IRS1 | Insulin Receptor Substrate 1 | 11.24 |
| 526 | IFT74 | Intraflagellar Transport 74 | 11.24 |
| 527 | FGF1 | Fibroblast Growth Factor 1 | 11.24 |
| 528 | PFN1 | Profilin 1 | 11.23 |
| 529 | ADM | Adrenomedullin | 11.21 |
| 530 | MIR29C | MicroRNA 29c | 11.2 |
| 531 | PLCB1 | Phospholipase C Beta 1 | 11.19 |
| 532 | ACTB | Actin Beta | 11.19 |
| 533 | FGF23 | Fibroblast Growth Factor 23 | 11.19 |
| 534 | UBQLN1 | Ubiquilin 1 | 11.19 |
| 535 | CHGA | Chromogranin A | 11.16 |
| 536 | MATR3 | Matrin 3 | 11.14 |
| 537 | CR1 | Complement C3b/C4b Receptor 1 (Knops Blood Group) | 11.13 |
| 538 | PLA2G4A | Phospholipase A2 Group IVA | 11.11 |
| 539 | NDE1 | NudE Neurodevelopment Protein 1 | 11.11 |
| 540 | C4A | Complement C4A (Rodgers Blood Group) | 11.09 |
| 541 | DKK1 | Dickkopf WNT Signaling Pathway Inhibitor 1 | 11.08 |
| 542 | DARS2 | Aspartyl-TRNA Synthetase 2, Mitochondrial | 11.06 |
| 543 | FOS | Fos Proto-Oncogene, AP-1 Transcription Factor Subunit | 11.06 |
| 544 | BECN1 | Beclin 1 | 11.02 |
| 545 | ACE2 | Angiotensin I Converting Enzyme 2 | 11.01 |
| 546 | CCR6 | C-C Motif Chemokine Receptor 6 | 11.01 |
| 547 | HUWE1 | HECT, UBA And WWE Domain Containing E3 Ubiquitin Protein Ligase 1 | 11.01 |
| 548 | CHIT1 | Chitinase 1 | 10.98 |
| 549 | MAP2K2 | Mitogen-Activated Protein Kinase Kinase 2 | 10.98 |
| 550 | ACTG1 | Actin Gamma 1 | 10.93 |
| 551 | CA4 | Carbonic Anhydrase 4 | 10.92 |
| 552 | RETN | Resistin | 10.9 |
| 553 | CALCA | Calcitonin Related Polypeptide Alpha | 10.86 |
| 554 | NQO1 | NAD(P)H Quinone Dehydrogenase 1 | 10.86 |
| 555 | LIPC | Lipase C, Hepatic Type | 10.85 |
| 556 | CHUK | Component Of Inhibitor Of Nuclear Factor Kappa B Kinase Complex | 10.84 |
| 557 | PRND | Prion Like Protein Doppel | 10.84 |
| 558 | TUBA1B | Tubulin Alpha 1b | 10.84 |
| 559 | CHM | CHM Rab Escort Protein | 10.82 |
| 560 | COG2 | Component Of Oligomeric Golgi Complex 2 | 10.81 |
| 561 | GAL | Galanin And GMAP Prepropeptide | 10.8 |
| 562 | RAB5A | RAB5A, Member RAS Oncogene Family | 10.79 |
| 563 | NOTCH2NLC | Notch 2 N-Terminal Like C | 10.78 |
| 564 | MAP3K5 | Mitogen-Activated Protein Kinase Kinase Kinase 5 | 10.77 |
| 565 | B2M | Beta-2-Microglobulin | 10.73 |
| 566 | SORT1 | Sortilin 1 | 10.72 |
| 567 | ATP5F1A | ATP Synthase F1 Subunit Alpha | 10.71 |
| 568 | ADORA2A | Adenosine A2a Receptor | 10.7 |
| 569 | TUBB2B | Tubulin Beta 2B Class IIb | 10.69 |
| 570 | DMPK | DM1 Protein Kinase | 10.68 |
| 571 | GRIA1 | Glutamate Ionotropic Receptor AMPA Type Subunit 1 | 10.67 |
| 572 | NSD1 | Nuclear Receptor Binding SET Domain Protein 1 | 10.67 |
| 573 | SMARCB1 | SWI/SNF Related, Matrix Associated, Actin Dependent Regulator Of Chromatin, Subfamily B, Member 1 | 10.67 |
| 574 | MIR15A | MicroRNA 15a | 10.67 |
| 575 | GRIA2 | Glutamate Ionotropic Receptor AMPA Type Subunit 2 | 10.66 |
| 576 | GAP43 | Growth Associated Protein 43 | 10.65 |
| 577 | KLK6 | Kallikrein Related Peptidase 6 | 10.65 |
| 578 | GBA2 | Glucosylceramidase Beta 2 | 10.65 |
| 579 | PHF1 | PHD Finger Protein 1 | 10.64 |
| 580 | LCAT | Lecithin-Cholesterol Acyltransferase | 10.63 |
| 581 | FURIN | Furin, Paired Basic Amino Acid Cleaving Enzyme | 10.63 |
| 582 | MT-ATP8 | Mitochondrially Encoded ATP Synthase Membrane Subunit 8 | 10.62 |
| 583 | HRH2 | Histamine Receptor H2 | 10.62 |
| 584 | SAA1 | Serum Amyloid A1 | 10.61 |
| 585 | DYRK1A | Dual Specificity Tyrosine Phosphorylation Regulated Kinase 1A | 10.61 |
| 586 | GNB3 | G Protein Subunit Beta 3 | 10.58 |
| 587 | CYP2C19 | Cytochrome P450 Family 2 Subfamily C Member 19 | 10.56 |
| 588 | ENO2 | Enolase 2 | 10.56 |
| 589 | C1R | Complement C1r | 10.56 |
| 590 | MT-TW | Mitochondrially Encoded TRNA-Trp (UGA/G) | 10.55 |
| 591 | NKX2-1 | NK2 Homeobox 1 | 10.55 |
| 592 | HMGB1 | High Mobility Group Box 1 | 10.54 |
| 593 | GRK2 | G Protein-Coupled Receptor Kinase 2 | 10.53 |
| 594 | VDAC1 | Voltage Dependent Anion Channel 1 | 10.51 |
| 595 | VHL | Von Hippel-Lindau Tumor Suppressor | 10.5 |
| 596 | TIMP3 | TIMP Metallopeptidase Inhibitor 3 | 10.48 |
| 597 | CALR | Calreticulin | 10.47 |
| 598 | ATF4 | Activating Transcription Factor 4 | 10.44 |
| 599 | DLL1 | Delta Like Canonical Notch Ligand 1 | 10.43 |
| 600 | PPIF | Peptidylprolyl Isomerase F | 10.43 |
| 601 | CAPN2 | Calpain 2 | 10.43 |
| 602 | VIP | Vasoactive Intestinal Peptide | 10.43 |
| 603 | OGDH | Oxoglutarate Dehydrogenase | 10.41 |
| 604 | CIB1 | Calcium And Integrin Binding 1 | 10.39 |
| 605 | ATM | ATM Serine/Threonine Kinase | 10.38 |
| 606 | PLD3 | Phospholipase D Family Member 3 | 10.36 |
| 607 | GSTO1 | Glutathione S-Transferase Omega 1 | 10.33 |
| 608 | ADD1 | Adducin 1 | 10.32 |
| 609 | LAMA3 | Laminin Subunit Alpha 3 | 10.32 |
| 610 | PIK3C3 | Phosphatidylinositol 3-Kinase Catalytic Subunit Type 3 | 10.32 |
| 611 | MIR124-1 | MicroRNA 124-1 | 10.29 |
| 612 | MEOX2 | Mesenchyme Homeobox 2 | 10.29 |
| 613 | AKT3 | AKT Serine/Threonine Kinase 3 | 10.28 |
| 614 | CHRNA4 | Cholinergic Receptor Nicotinic Alpha 4 Subunit | 10.27 |
| 615 | TBX1 | T-Box Transcription Factor 1 | 10.27 |
| 616 | MAP1B | Microtubule Associated Protein 1B | 10.26 |
| 617 | GRM5 | Glutamate Metabotropic Receptor 5 | 10.24 |
| 618 | BLVRB | Biliverdin Reductase B | 10.24 |
| 619 | MT3 | Metallothionein 3 | 10.24 |
| 620 | BAD | BCL2 Associated Agonist Of Cell Death | 10.23 |
| 621 | MALAT1 | Metastasis Associated Lung Adenocarcinoma Transcript 1 | 10.22 |
| 622 | MYLK | Myosin Light Chain Kinase | 10.22 |
| 623 | CDKN2B-AS1 | CDKN2B Antisense RNA 1 | 10.21 |
| 624 | MIR26B | MicroRNA 26b | 10.21 |
| 625 | RPS6KB1 | Ribosomal Protein S6 Kinase B1 | 10.19 |
| 626 | NEAT1 | Nuclear Paraspeckle Assembly Transcript 1 | 10.18 |
| 627 | SERPINE2 | Serpin Family E Member 2 | 10.17 |
| 628 | COLEC12 | Collectin Subfamily Member 12 | 10.15 |
| 629 | APAF1 | Apoptotic Peptidase Activating Factor 1 | 10.14 |
| 630 | SETD2 | SET Domain Containing 2, Histone Lysine Methyltransferase | 10.14 |
| 631 | CTLA4 | Cytotoxic T-Lymphocyte Associated Protein 4 | 10.13 |
| 632 | NTF4 | Neurotrophin 4 | 10.12 |
| 633 | FLI1 | Fli-1 Proto-Oncogene, ETS Transcription Factor | 10.11 |
| 634 | PTH | Parathyroid Hormone | 10.1 |
| 635 | BSG | Basigin (Ok Blood Group) | 10.08 |
| 636 | NCSTN | Nicastrin | 10.07 |
| 637 | APOD | Apolipoprotein D | 10.07 |
| 638 | C4B | Complement C4B (Chido Blood Group) | 10.06 |
| 639 | EIF2AK3 | Eukaryotic Translation Initiation Factor 2 Alpha Kinase 3 | 10.06 |
| 640 | DDIT3 | DNA Damage Inducible Transcript 3 | 10.05 |
| 641 | LRPAP1 | LDL Receptor Related Protein Associated Protein 1 | 10.05 |
| 642 | CAPN3 | Calpain 3 | 10.04 |
| 643 | PRL | Prolactin | 10.03 |
| 644 | PTPA | Protein Phosphatase 2 Phosphatase Activator | 10.01 |
| 645 | FABP3 | Fatty Acid Binding Protein 3 | 10.01 |
| 646 | SLC13A5 | Solute Carrier Family 13 Member 5 | 10 |
| 647 | POMC | Proopiomelanocortin | 9.98 |
| 648 | NR1H4 | Nuclear Receptor Subfamily 1 Group H Member 4 | 9.97 |
| 649 | CDR1 | Cerebellar Degeneration Related Protein 1 | 9.95 |
| 650 | WNT2 | Wnt Family Member 2 | 9.95 |
| 651 | CCND1 | Cyclin D1 | 9.94 |
| 652 | CHRNB2 | Cholinergic Receptor Nicotinic Beta 2 Subunit | 9.93 |
| 653 | SLC25A4 | Solute Carrier Family 25 Member 4 | 9.93 |
| 654 | APC | APC Regulator Of WNT Signaling Pathway | 9.93 |
| 655 | MIAT | Myocardial Infarction Associated Transcript | 9.92 |
| 656 | GPC1 | Glypican 1 | 9.91 |
| 657 | HNF1A | HNF1 Homeobox A | 9.89 |
| 658 | CRAT | Carnitine O-Acetyltransferase | 9.88 |
| 659 | APOC3 | Apolipoprotein C3 | 9.88 |
| 660 | ITGB3 | Integrin Subunit Beta 3 | 9.86 |
| 661 | PLCB4 | Phospholipase C Beta 4 | 9.85 |
| 662 | WNT8B | Wnt Family Member 8B | 9.84 |
| 663 | CNR2 | Cannabinoid Receptor 2 | 9.82 |
| 664 | CNGA3 | Cyclic Nucleotide Gated Channel Subunit Alpha 3 | 9.81 |
| 665 | FUCA1 | Alpha-L-Fucosidase 1 | 9.81 |
| 666 | DLST | Dihydrolipoamide S-Succinyltransferase | 9.8 |
| 667 | TUBB3 | Tubulin Beta 3 Class III | 9.8 |
| 668 | LRP6 | LDL Receptor Related Protein 6 | 9.79 |
| 669 | CASP7 | Caspase 7 | 9.78 |
| 670 | TNFRSF1B | TNF Receptor Superfamily Member 1B | 9.78 |
| 671 | EIF2AK2 | Eukaryotic Translation Initiation Factor 2 Alpha Kinase 2 | 9.76 |
| 672 | SEMA3A | Semaphorin 3A | 9.73 |
| 673 | TIMM8A | Translocase Of Inner Mitochondrial Membrane 8A | 9.72 |
| 674 | PIK3R2 | Phosphoinositide-3-Kinase Regulatory Subunit 2 | 9.72 |
| 675 | MAPK8IP1 | Mitogen-Activated Protein Kinase 8 Interacting Protein 1 | 9.71 |
| 676 | CNBP | CCHC-Type Zinc Finger Nucleic Acid Binding Protein | 9.7 |
| 677 | PRKCZ | Protein Kinase C Zeta | 9.69 |
| 678 | DPYSL2 | Dihydropyrimidinase Like 2 | 9.68 |
| 679 | RRM2B | Ribonucleotide Reductase Regulatory TP53 Inducible Subunit M2B | 9.65 |
| 680 | IGF2 | Insulin Like Growth Factor 2 | 9.64 |
| 681 | CSF2 | Colony Stimulating Factor 2 | 9.64 |
| 682 | RYR3 | Ryanodine Receptor 3 | 9.63 |
| 683 | ANXA5 | Annexin A5 | 9.63 |
| 684 | NTRK3 | Neurotrophic Receptor Tyrosine Kinase 3 | 9.59 |
| 685 | CCL5 | C-C Motif Chemokine Ligand 5 | 9.59 |
| 686 | PPP3R1 | Protein Phosphatase 3 Regulatory Subunit B, Alpha | 9.59 |
| 687 | TOR1A | Torsin Family 1 Member A | 9.58 |
| 688 | CYP46A1 | Cytochrome P450 Family 46 Subfamily A Member 1 | 9.58 |
| 689 | DVL1 | Dishevelled Segment Polarity Protein 1 | 9.57 |
| 690 | ITPR3 | Inositol 1,4,5-Trisphosphate Receptor Type 3 | 9.53 |
| 691 | GCG | Glucagon | 9.53 |
| 692 | HLA-DRA | Major Histocompatibility Complex, Class II, DR Alpha | 9.53 |
| 693 | IGF2R | Insulin Like Growth Factor 2 Receptor | 9.52 |
| 694 | BLMH | Bleomycin Hydrolase | 9.51 |
| 695 | PCNT | Pericentrin | 9.5 |
| 696 | HGSNAT | Heparan-Alpha-Glucosaminide N-Acetyltransferase | 9.49 |
| 697 | HSPB1 | Heat Shock Protein Family B (Small) Member 1 | 9.49 |
| 698 | IRS2 | Insulin Receptor Substrate 2 | 9.49 |
| 699 | GHRL | Ghrelin And Obestatin Prepropeptide | 9.48 |
| 700 | CALM1 | Calmodulin 1 | 9.48 |
| 701 | PSENEN | Presenilin Enhancer, Gamma-Secretase Subunit | 9.47 |
| 702 | PIK3CG | Phosphatidylinositol-4,5-Bisphosphate 3-Kinase Catalytic Subunit Gamma | 9.47 |
| 703 | NDRG2 | NDRG Family Member 2 | 9.47 |
| 704 | MAPK9 | Mitogen-Activated Protein Kinase 9 | 9.47 |
| 705 | SLC18A3 | Solute Carrier Family 18 Member A3 | 9.46 |
| 706 | PSMC6 | Proteasome 26S Subunit, ATPase 6 | 9.46 |
| 707 | ATXN10 | Ataxin 10 | 9.45 |
| 708 | MIR93 | MicroRNA 93 | 9.42 |
| 709 | APBA1 | Amyloid Beta Precursor Protein Binding Family A Member 1 | 9.41 |
| 710 | LAMP1 | Lysosomal Associated Membrane Protein 1 | 9.41 |
| 711 | LRP2 | LDL Receptor Related Protein 2 | 9.4 |
| 712 | EIF2S1 | Eukaryotic Translation Initiation Factor 2 Subunit Alpha | 9.4 |
| 713 | GLP1R | Glucagon Like Peptide 1 Receptor | 9.4 |
| 714 | CALM2 | Calmodulin 2 | 9.37 |
| 715 | RAB3A | RAB3A, Member RAS Oncogene Family | 9.36 |
| 716 | AATF | Apoptosis Antagonizing Transcription Factor | 9.35 |
| 717 | MT-RNR2 | Mitochondrially Encoded 16S RRNA | 9.35 |
| 718 | MMP3 | Matrix Metallopeptidase 3 | 9.34 |
| 719 | LRP8 | LDL Receptor Related Protein 8 | 9.34 |
| 720 | STAT3 | Signal Transducer And Activator Of Transcription 3 | 9.33 |
| 721 | F7 | Coagulation Factor VII | 9.33 |
| 722 | CASP6 | Caspase 6 | 9.33 |
| 723 | GRIN2C | Glutamate Ionotropic Receptor NMDA Type Subunit 2C | 9.32 |
| 724 | MMP1 | Matrix Metallopeptidase 1 | 9.3 |
| 725 | DISC1 | DISC1 Scaffold Protein | 9.3 |
| 726 | CALHM1 | Calcium Homeostasis Modulator 1 | 9.3 |
| 727 | ERN1 | Endoplasmic Reticulum To Nucleus Signaling 1 | 9.29 |
| 728 | PPID | Peptidylprolyl Isomerase D | 9.29 |
| 729 | RBM8A | RNA Binding Motif Protein 8A | 9.28 |
| 730 | DKK3 | Dickkopf WNT Signaling Pathway Inhibitor 3 | 9.28 |
| 731 | HAR1B | Highly Accelerated Region 1B | 9.26 |
| 732 | EXOC3L2 | Exocyst Complex Component 3 Like 2 | 9.26 |
| 733 | SRF | Serum Response Factor | 9.26 |
| 734 | TRAF2 | TNF Receptor Associated Factor 2 | 9.26 |
| 735 | CFLAR | CASP8 And FADD Like Apoptosis Regulator | 9.25 |
| 736 | CAST | Calpastatin | 9.24 |
| 737 | SOAT1 | Sterol O-Acyltransferase 1 | 9.23 |
| 738 | ADAMTS13 | ADAM Metallopeptidase With Thrombospondin Type 1 Motif 13 | 9.21 |
| 739 | NEFM | Neurofilament Medium | 9.21 |
| 740 | PMM2 | Phosphomannomutase 2 | 9.2 |
| 741 | DHCR24 | 24-Dehydrocholesterol Reductase | 9.19 |
| 742 | TFAP2A | Transcription Factor AP-2 Alpha | 9.18 |
| 743 | ADAMTS4 | ADAM Metallopeptidase With Thrombospondin Type 1 Motif 4 | 9.16 |
| 744 | CFAP410 | Cilia And Flagella Associated Protein 410 | 9.16 |
| 745 | NAMPT | Nicotinamide Phosphoribosyltransferase | 9.14 |
| 746 | CFTR | CF Transmembrane Conductance Regulator | 9.13 |
| 747 | STMN2 | Stathmin 2 | 9.1 |
| 748 | HLA-DRB5 | Major Histocompatibility Complex, Class II, DR Beta 5 | 9.09 |
| 749 | AGBL5 | ATP/GTP Binding Protein Like 5 | 9.09 |
| 750 | TNFRSF11A | TNF Receptor Superfamily Member 11a | 9.08 |
| 751 | ITGA4 | Integrin Subunit Alpha 4 | 9.07 |
| 752 | OGT | O-Linked N-Acetylglucosamine (GlcNAc) Transferase | 9.07 |
| 753 | THBS1 | Thrombospondin 1 | 9.06 |
| 754 | THBS4 | Thrombospondin 4 | 9.06 |
| 755 | FRMD4A | FERM Domain Containing 4A | 9.05 |
| 756 | HARS1 | Histidyl-TRNA Synthetase 1 | 9.05 |
| 757 | OPA1 | OPA1 Mitochondrial Dynamin Like GTPase | 9.04 |
| 758 | PROCR | Protein C Receptor | 9.04 |
| 759 | BACE2 | Beta-Secretase 2 | 9.03 |
| 760 | AARS2 | Alanyl-TRNA Synthetase 2, Mitochondrial | 9 |
| 761 | IFNB1 | Interferon Beta 1 | 8.99 |
| 762 | MSR1 | Macrophage Scavenger Receptor 1 | 8.99 |
| 763 | ADRA2B | Adrenoceptor Alpha 2B | 8.98 |
| 764 | PDE2A | Phosphodiesterase 2A | 8.98 |
| 765 | MIR9-1 | MicroRNA 9-1 | 8.98 |
| 766 | CASP2 | Caspase 2 | 8.97 |
| 767 | HEY2 | Hes Related Family BHLH Transcription Factor With YRPW Motif 2 | 8.96 |
| 768 | GM2A | GM2 Ganglioside Activator | 8.93 |
| 769 | ABCA2 | ATP Binding Cassette Subfamily A Member 2 | 8.92 |
| 770 | CDH1 | Cadherin 1 | 8.92 |
| 771 | CSNK1A1 | Casein Kinase 1 Alpha 1 | 8.91 |
| 772 | GNAO1 | G Protein Subunit Alpha O1 | 8.89 |
| 773 | RCAN1 | Regulator Of Calcineurin 1 | 8.88 |
| 774 | CHGB | Chromogranin B | 8.88 |
| 775 | ATP5PD | ATP Synthase Peripheral Stalk Subunit D | 8.88 |
| 776 | HAR1A | Highly Accelerated Region 1A | 8.88 |
| 777 | BAX | BCL2 Associated X, Apoptosis Regulator | 8.85 |
| 778 | COX4I1 | Cytochrome C Oxidase Subunit 4I1 | 8.82 |
| 779 | APH1A | Aph-1 Homolog A, Gamma-Secretase Subunit | 8.81 |
| 780 | MB | Myoglobin | 8.81 |
| 781 | FLNC | Filamin C | 8.8 |
| 782 | PLA2G3 | Phospholipase A2 Group III | 8.8 |
| 783 | TUBB2A | Tubulin Beta 2A Class IIa | 8.78 |
| 784 | CALM3 | Calmodulin 3 | 8.78 |
| 785 | SRSF7 | Serine And Arginine Rich Splicing Factor 7 | 8.76 |
| 786 | HSD17B10 | Hydroxysteroid 17-Beta Dehydrogenase 10 | 8.75 |
| 787 | PZP | PZP Alpha-2-Macroglobulin Like | 8.73 |
| 788 | FPR2 | Formyl Peptide Receptor 2 | 8.72 |
| 789 | GLRX | Glutaredoxin | 8.72 |
| 790 | TUBB4A | Tubulin Beta 4A Class IVa | 8.71 |
| 791 | APOC2 | Apolipoprotein C2 | 8.71 |
| 792 | PEBP1 | Phosphatidylethanolamine Binding Protein 1 | 8.7 |
| 793 | NRBF2 | Nuclear Receptor Binding Factor 2 | 8.69 |
| 794 | CSNK2A1 | Casein Kinase 2 Alpha 1 | 8.69 |
| 795 | TET2 | Tet Methylcytosine Dioxygenase 2 | 8.69 |
| 796 | PITRM1 | Pitrilysin Metallopeptidase 1 | 8.68 |
| 797 | ETHE1 | ETHE1 Persulfide Dioxygenase | 8.67 |
| 798 | SDHA | Succinate Dehydrogenase Complex Flavoprotein Subunit A | 8.67 |
| 799 | REN | Renin | 8.67 |
| 800 | ATP5PF | ATP Synthase Peripheral Stalk Subunit F6 | 8.66 |
| 801 | MIR128-1 | MicroRNA 128-1 | 8.65 |
| 802 | APLP2 | Amyloid Beta Precursor Like Protein 2 | 8.64 |
| 803 | PLCD1 | Phospholipase C Delta 1 | 8.61 |
| 804 | TAC1 | Tachykinin Precursor 1 | 8.61 |
| 805 | PADI2 | Peptidyl Arginine Deiminase 2 | 8.61 |
| 806 | ABCB1 | ATP Binding Cassette Subfamily B Member 1 | 8.6 |
| 807 | ABCD1 | ATP Binding Cassette Subfamily D Member 1 | 8.6 |
| 808 | F10 | Coagulation Factor X | 8.6 |
| 809 | DERL1 | Derlin 1 | 8.59 |
| 810 | MT-ND4L | Mitochondrially Encoded NADH:Ubiquinone Oxidoreductase Core Subunit 4L | 8.59 |
| 811 | CNTF | Ciliary Neurotrophic Factor | 8.58 |
| 812 | PMPCA | Peptidase, Mitochondrial Processing Subunit Alpha | 8.58 |
| 813 | CR2 | Complement C3d Receptor 2 | 8.58 |
| 814 | CTNNA3 | Catenin Alpha 3 | 8.58 |
| 815 | PSMB1 | Proteasome 20S Subunit Beta 1 | 8.56 |
| 816 | MIR142 | MicroRNA 142 | 8.56 |
| 817 | HGF | Hepatocyte Growth Factor | 8.56 |
| 818 | HTR6 | 5-Hydroxytryptamine Receptor 6 | 8.56 |
| 819 | CDH23 | Cadherin Related 23 | 8.55 |
| 820 | VPS26A | VPS26, Retromer Complex Component A | 8.54 |
| 821 | KLF4 | Kruppel Like Factor 4 | 8.53 |
| 822 | KLC1 | Kinesin Light Chain 1 | 8.53 |
| 823 | CCL4 | C-C Motif Chemokine Ligand 4 | 8.52 |
| 824 | DLL3 | Delta Like Canonical Notch Ligand 3 | 8.51 |
| 825 | CSNK2B | Casein Kinase 2 Beta | 8.51 |
| 826 | DKK2 | Dickkopf WNT Signaling Pathway Inhibitor 2 | 8.5 |
| 827 | TUBA1C | Tubulin Alpha 1c | 8.5 |
| 828 | KIDINS220 | Kinase D Interacting Substrate 220 | 8.48 |
| 829 | GSK3A | Glycogen Synthase Kinase 3 Alpha | 8.48 |
| 830 | CALML3 | Calmodulin Like 3 | 8.47 |
| 831 | CALML5 | Calmodulin Like 5 | 8.47 |
| 832 | CDK5R1 | Cyclin Dependent Kinase 5 Regulatory Subunit 1 | 8.47 |
| 833 | TPH1 | Tryptophan Hydroxylase 1 | 8.47 |
| 834 | MIR320A | MicroRNA 320a | 8.47 |
| 835 | QPCT | Glutaminyl-Peptide Cyclotransferase | 8.46 |
| 836 | CSNK1D | Casein Kinase 1 Delta | 8.45 |
| 837 | PABPN1 | Poly(A) Binding Protein Nuclear 1 | 8.44 |
| 838 | TTBK1 | Tau Tubulin Kinase 1 | 8.43 |
| 839 | AGRN | Agrin | 8.42 |
| 840 | COX6B1 | Cytochrome C Oxidase Subunit 6B1 | 8.42 |
| 841 | TM2D1 | TM2 Domain Containing 1 | 8.42 |
| 842 | ERCC8 | ERCC Excision Repair 8, CSA Ubiquitin Ligase Complex Subunit | 8.42 |
| 843 | TUBB6 | Tubulin Beta 6 Class V | 8.41 |
| 844 | PDE6B | Phosphodiesterase 6B | 8.41 |
| 845 | C19orf12 | Chromosome 19 Open Reading Frame 12 | 8.4 |
| 846 | MAP2K7 | Mitogen-Activated Protein Kinase Kinase 7 | 8.4 |
| 847 | NDUFS3 | NADH:Ubiquinone Oxidoreductase Core Subunit S3 | 8.4 |
| 848 | PPP3CA | Protein Phosphatase 3 Catalytic Subunit Alpha | 8.39 |
| 849 | APBB2 | Amyloid Beta Precursor Protein Binding Family B Member 2 | 8.37 |
| 850 | ADAM19 | ADAM Metallopeptidase Domain 19 | 8.37 |
| 851 | CD59 | CD59 Molecule (CD59 Blood Group) | 8.37 |
| 852 | CAV3 | Caveolin 3 | 8.37 |
| 853 | SLC25A6 | Solute Carrier Family 25 Member 6 | 8.37 |
| 854 | PSMC2 | Proteasome 26S Subunit, ATPase 2 | 8.37 |
| 855 | JCAD | Junctional Cadherin 5 Associated | 8.36 |
| 856 | SPHK2 | Sphingosine Kinase 2 | 8.35 |
| 857 | PREP | Prolyl Endopeptidase | 8.35 |
| 858 | PSMA5 | Proteasome 20S Subunit Alpha 5 | 8.35 |
| 859 | ATP5PB | ATP Synthase Peripheral Stalk-Membrane Subunit B | 8.32 |
| 860 | AD9 | Alzheimer Disease 9 | 8.32 |
| 861 | PSMC1 | Proteasome 26S Subunit, ATPase 1 | 8.32 |
| 862 | IFNA1 | Interferon Alpha 1 | 8.31 |
| 863 | HNRNPC | Heterogeneous Nuclear Ribonucleoprotein C | 8.31 |
| 864 | IDUA | Alpha-L-Iduronidase | 8.31 |
| 865 | APLP1 | Amyloid Beta Precursor Like Protein 1 | 8.31 |
| 866 | ERCC1 | ERCC Excision Repair 1, Endonuclease Non-Catalytic Subunit | 8.29 |
| 867 | CD68 | CD68 Molecule | 8.29 |
| 868 | NTS | Neurotensin | 8.28 |
| 869 | CSNK2A2 | Casein Kinase 2 Alpha 2 | 8.28 |
| 870 | PROC | Protein C, Inactivator Of Coagulation Factors Va And VIIIa | 8.28 |
| 871 | MSN | Moesin | 8.27 |
| 872 | CD8A | CD8a Molecule | 8.27 |
| 873 | CDK5RAP2 | CDK5 Regulatory Subunit Associated Protein 2 | 8.26 |
| 874 | TUBA3D | Tubulin Alpha 3d | 8.26 |
| 875 | CSNK1E | Casein Kinase 1 Epsilon | 8.26 |
| 876 | AZIN2 | Antizyme Inhibitor 2 | 8.25 |
| 877 | NES | Nestin | 8.25 |
| 878 | MAZ | MYC Associated Zinc Finger Protein | 8.25 |
| 879 | APBB3 | Amyloid Beta Precursor Protein Binding Family B Member 3 | 8.23 |
| 880 | UQCRC1 | Ubiquinol-Cytochrome C Reductase Core Protein 1 | 8.22 |
| 881 | PSCA | Prostate Stem Cell Antigen | 8.22 |
| 882 | MIR197 | MicroRNA 197 | 8.22 |
| 883 | SREK1IP1 | SREK1 Interacting Protein 1 | 8.22 |
| 884 | REG1A | Regenerating Family Member 1 Alpha | 8.21 |
| 885 | CSNK1A1L | Casein Kinase 1 Alpha 1 Like | 8.21 |
| 886 | DNMBP | Dynamin Binding Protein | 8.21 |
| 887 | KIF5B | Kinesin Family Member 5B | 8.2 |
| 888 | FASLG | Fas Ligand | 8.2 |
| 889 | PCDH11X | Protocadherin 11 X-Linked | 8.19 |
| 890 | NAE1 | NEDD8 Activating Enzyme E1 Subunit 1 | 8.19 |
| 891 | MIR133B | MicroRNA 133b | 8.18 |
| 892 | AVP | Arginine Vasopressin | 8.18 |
| 893 | APBA3 | Amyloid Beta Precursor Protein Binding Family A Member 3 | 8.18 |
| 894 | GSAP | Gamma-Secretase Activating Protein | 8.18 |
| 895 | BACE1-AS | BACE1 Antisense RNA | 8.17 |
| 896 | PRPH | Peripherin | 8.17 |
| 897 | TNFRSF21 | TNF Receptor Superfamily Member 21 | 8.15 |
| 898 | TMED10 | Transmembrane P24 Trafficking Protein 10 | 8.14 |
| 899 | APH1B | Aph-1 Homolog B, Gamma-Secretase Subunit | 8.14 |
| 900 | DOCK3 | Dedicator Of Cytokinesis 3 | 8.14 |
| 901 | CH25H | Cholesterol 25-Hydroxylase | 8.14 |
| 902 | XDH | Xanthine Dehydrogenase | 8.14 |
| 903 | MIR181C | MicroRNA 181c | 8.14 |
| 904 | KCNIP3 | Potassium Voltage-Gated Channel Interacting Protein 3 | 8.13 |
| 905 | GATA1 | GATA Binding Protein 1 | 8.12 |
| 906 | TSHZ3 | Teashirt Zinc Finger Homeobox 3 | 8.1 |
| 907 | SNHG3 | Small Nucleolar RNA Host Gene 3 | 8.1 |
| 908 | LINC01772 | Long Intergenic Non-Protein Coding RNA 1772 | 8.1 |
| 909 | CTSA | Cathepsin A | 8.1 |
| 910 | IRS4 | Insulin Receptor Substrate 4 | 8.09 |
| 911 | NR3C1 | Nuclear Receptor Subfamily 3 Group C Member 1 | 8.09 |
| 912 | RTN3 | Reticulon 3 | 8.09 |
| 913 | NOX4 | NADPH Oxidase 4 | 8.08 |
| 914 | TFCP2 | Transcription Factor CP2 | 8.06 |
| 915 | AR | Androgen Receptor | 8.05 |
| 916 | VDAC2 | Voltage Dependent Anion Channel 2 | 8.04 |
| 917 | PCMT1 | Protein-L-Isoaspartate (D-Aspartate) O-Methyltransferase | 8.04 |
| 918 | TMEFF2 | Transmembrane Protein With EGF Like And Two Follistatin Like Domains 2 | 8.03 |
| 919 | COX6C | Cytochrome C Oxidase Subunit 6C | 8.03 |
| 920 | JAG2 | Jagged Canonical Notch Ligand 2 | 8.02 |
| 921 | GPR3 | G Protein-Coupled Receptor 3 | 8.01 |
| 922 | ATP5PO | ATP Synthase Peripheral Stalk Subunit OSCP | 8 |
| 923 | SERPINF1 | Serpin Family F Member 1 | 7.98 |
| 924 | PSMA1 | Proteasome 20S Subunit Alpha 1 | 7.97 |
| 925 | DKK4 | Dickkopf WNT Signaling Pathway Inhibitor 4 | 7.97 |
| 926 | TGFA | Transforming Growth Factor Alpha | 7.97 |
| 927 | ADCY10 | Adenylate Cyclase 10 | 7.97 |
| 928 | MTNR1A | Melatonin Receptor 1A | 7.97 |
| 929 | CYP27A1 | Cytochrome P450 Family 27 Subfamily A Member 1 | 7.97 |
| 930 | MARK1 | Microtubule Affinity Regulating Kinase 1 | 7.97 |
| 931 | APBA2 | Amyloid Beta Precursor Protein Binding Family A Member 2 | 7.97 |
| 932 | SNAP91 | Synaptosome Associated Protein 91 | 7.97 |
| 933 | CLSTN1 | Calsyntenin 1 | 7.97 |
| 934 | RALGPS2 | Ral GEF With PH Domain And SH3 Binding Motif 2 | 7.97 |
| 935 | DCHS2 | Dachsous Cadherin-Related 2 | 7.97 |
| 936 | BCYRN1 | Brain Cytoplasmic RNA 1 | 7.97 |
| 937 | LINC01080 | Long Intergenic Non-Protein Coding RNA 1080 | 7.97 |
| 938 | LINC01616 | Long Intergenic Non-Protein Coding RNA 1616 | 7.97 |
| 939 | KLK8 | Kallikrein Related Peptidase 8 | 7.95 |
| 940 | RNF146 | Ring Finger Protein 146 | 7.95 |
| 941 | ATP5F1C | ATP Synthase F1 Subunit Gamma | 7.95 |
| 942 | SOX2-OT | SOX2 Overlapping Transcript | 7.95 |
| 943 | NEDD4 | NEDD4 E3 Ubiquitin Protein Ligase | 7.94 |
| 944 | PIK3R4 | Phosphoinositide-3-Kinase Regulatory Subunit 4 | 7.92 |
| 945 | MARK4 | Microtubule Affinity Regulating Kinase 4 | 7.92 |
| 946 | ANKS1B | Ankyrin Repeat And Sterile Alpha Motif Domain Containing 1B | 7.92 |
| 947 | FRAT1 | FRAT Regulator Of WNT Signaling Pathway 1 | 7.92 |
| 948 | COL25A1 | Collagen Type XXV Alpha 1 Chain | 7.92 |
| 949 | SLC39A1 | Solute Carrier Family 39 Member 1 | 7.92 |
| 950 | SLC30A6 | Solute Carrier Family 30 Member 6 | 7.92 |
| 951 | SORCS3 | Sortilin Related VPS10 Domain Containing Receptor 3 | 7.92 |
| 952 | SEPTIN1 | Septin 1 | 7.92 |
| 953 | MROH8 | Maestro Heat Like Repeat Family Member 8 | 7.92 |
| 954 | MALRD1 | MAM And LDL Receptor Class A Domain Containing 1 | 7.92 |
| 955 | ASAH2B | N-Acylsphingosine Amidohydrolase 2B | 7.92 |
| 956 | MIR363 | MicroRNA 363 | 7.92 |
| 957 | MIR511 | MicroRNA 511 | 7.92 |
| 958 | LRP1-AS | LRP1 Antisense RNA | 7.92 |
| 959 | LOC111258525 | NOS1 1f And 1g Alternate Promoter Region | 7.92 |
| 960 | SULT1A3 | Sulfotransferase Family 1A Member 3 | 7.92 |
| 961 | C3 | Complement C3 | 7.89 |
| 962 | SRRT | Serrate, RNA Effector Molecule | 7.89 |
| 963 | ADRA1A | Adrenoceptor Alpha 1A | 7.88 |
| 964 | ADAR | Adenosine Deaminase RNA Specific | 7.88 |
| 965 | TJP1 | Tight Junction Protein 1 | 7.87 |
| 966 | TYR | Tyrosinase | 7.86 |
| 967 | MC4R | Melanocortin 4 Receptor | 7.85 |
| 968 | GBE1 | 1,4-Alpha-Glucan Branching Enzyme 1 | 7.85 |
| 969 | ATP1A2 | ATPase Na+/K+ Transporting Subunit Alpha 2 | 7.83 |
| 970 | PEX6 | Peroxisomal Biogenesis Factor 6 | 7.83 |
| 971 | ARSH | Arylsulfatase Family Member H | 7.83 |
| 972 | MRAP | Melanocortin 2 Receptor Accessory Protein | 7.82 |
| 973 | MIR98 | MicroRNA 98 | 7.81 |
| 974 | RAB7A | RAB7A, Member RAS Oncogene Family | 7.81 |
| 975 | FBN2 | Fibrillin 2 | 7.81 |
| 976 | DDX41 | DEAD-Box Helicase 41 | 7.79 |
| 977 | NLRP3 | NLR Family Pyrin Domain Containing 3 | 7.78 |
| 978 | ITGAL | Integrin Subunit Alpha L | 7.76 |
| 979 | MIR433 | MicroRNA 433 | 7.74 |
| 980 | RAB8A | RAB8A, Member RAS Oncogene Family | 7.74 |
| 981 | VAPB | VAMP Associated Protein B And C | 7.74 |
| 982 | HEY1 | Hes Related Family BHLH Transcription Factor With YRPW Motif 1 | 7.74 |
| 983 | TIMP2 | TIMP Metallopeptidase Inhibitor 2 | 7.73 |
| 984 | WASHC5 | WASH Complex Subunit 5 | 7.69 |
| 985 | ADA | Adenosine Deaminase | 7.68 |
| 986 | MAG | Myelin Associated Glycoprotein | 7.66 |
| 987 | AHI1 | Abelson Helper Integration Site 1 | 7.65 |
| 988 | ADORA1 | Adenosine A1 Receptor | 7.64 |
| 989 | EIF4G1 | Eukaryotic Translation Initiation Factor 4 Gamma 1 | 7.64 |
| 990 | PEX1 | Peroxisomal Biogenesis Factor 1 | 7.63 |
| 991 | PEX19 | Peroxisomal Biogenesis Factor 19 | 7.63 |
| 992 | EDNRB | Endothelin Receptor Type B | 7.63 |
| 993 | HNRNPDL | Heterogeneous Nuclear Ribonucleoprotein D Like | 7.62 |
| 994 | UBC | Ubiquitin C | 7.61 |
| 995 | ASAH1 | N-Acylsphingosine Amidohydrolase 1 | 7.61 |
| 996 | GPT | Glutamic--Pyruvic Transaminase | 7.6 |
| 997 | MT-TQ | Mitochondrially Encoded TRNA-Gln (CAA/G) | 7.6 |
| 998 | UFD1 | Ubiquitin Recognition Factor In ER Associated Degradation 1 | 7.6 |
| 999 | PCNA | Proliferating Cell Nuclear Antigen | 7.59 |
| 1000 | PML | PML Nuclear Body Scaffold | 7.58 |
| 1001 | HPGDS | Hematopoietic Prostaglandin D Synthase | 7.57 |
| 1002 | PRTN3 | Proteinase 3 | 7.57 |
| 1003 | OXT | Oxytocin/Neurophysin I Prepropeptide | 7.56 |
| 1004 | EPRS1 | Glutamyl-Prolyl-TRNA Synthetase 1 | 7.54 |
| 1005 | EHMT1 | Euchromatic Histone Lysine Methyltransferase 1 | 7.53 |
| 1006 | STING1 | Stimulator Of Interferon Response CGAMP Interactor 1 | 7.52 |
| 1007 | CDC42 | Cell Division Cycle 42 | 7.52 |
| 1008 | PLP1 | Proteolipid Protein 1 | 7.52 |
| 1009 | GRM1 | Glutamate Metabotropic Receptor 1 | 7.52 |
| 1010 | KCNMA1 | Potassium Calcium-Activated Channel Subfamily M Alpha 1 | 7.51 |
| 1011 | PPP2R2B | Protein Phosphatase 2 Regulatory Subunit Bbeta | 7.51 |
| 1012 | IFIH1 | Interferon Induced With Helicase C Domain 1 | 7.5 |
| 1013 | FGA | Fibrinogen Alpha Chain | 7.49 |
| 1014 | IAPP | Islet Amyloid Polypeptide | 7.49 |
| 1015 | RD3 | Retinal Degeneration 3, GUCY2D Regulator | 7.44 |
| 1016 | MT-TH | Mitochondrially Encoded TRNA-His (CAU/C) | 7.44 |
| 1017 | FCGR2A | Fc Fragment Of IgG Receptor IIa | 7.43 |
| 1018 | JAM2 | Junctional Adhesion Molecule 2 | 7.43 |
| 1019 | F2R | Coagulation Factor II Thrombin Receptor | 7.42 |
| 1020 | CYP1A1 | Cytochrome P450 Family 1 Subfamily A Member 1 | 7.37 |
| 1021 | P2RY12 | Purinergic Receptor P2Y12 | 7.37 |
| 1022 | SOX2 | SRY-Box Transcription Factor 2 | 7.36 |
| 1023 | GALC | Galactosylceramidase | 7.35 |
| 1024 | FBXO7 | F-Box Protein 7 | 7.35 |
| 1025 | CRX | Cone-Rod Homeobox | 7.35 |
| 1026 | FZD5 | Frizzled Class Receptor 5 | 7.35 |
| 1027 | MT-ND3 | Mitochondrially Encoded NADH:Ubiquinone Oxidoreductase Core Subunit 3 | 7.34 |
| 1028 | BRAF | B-Raf Proto-Oncogene, Serine/Threonine Kinase | 7.34 |
| 1029 | KIF7 | Kinesin Family Member 7 | 7.29 |
| 1030 | TFAM | Transcription Factor A, Mitochondrial | 7.29 |
| 1031 | ACO1 | Aconitase 1 | 7.28 |
| 1032 | GCH1 | GTP Cyclohydrolase 1 | 7.27 |
| 1033 | OSM | Oncostatin M | 7.26 |
| 1034 | MIR24-1 | MicroRNA 24-1 | 7.26 |
| 1035 | MMP14 | Matrix Metallopeptidase 14 | 7.25 |
| 1036 | EDNRA | Endothelin Receptor Type A | 7.23 |
| 1037 | GTF2I | General Transcription Factor IIi | 7.22 |
| 1038 | DNAJC13 | DnaJ Heat Shock Protein Family (Hsp40) Member C13 | 7.21 |
| 1039 | CX3CL1 | C-X3-C Motif Chemokine Ligand 1 | 7.21 |
| 1040 | FTH1 | Ferritin Heavy Chain 1 | 7.2 |
| 1041 | SERPIND1 | Serpin Family D Member 1 | 7.19 |
| 1042 | CDH2 | Cadherin 2 | 7.18 |
| 1043 | CXCL10 | C-X-C Motif Chemokine Ligand 10 | 7.17 |
| 1044 | BCR | BCR Activator Of RhoGEF And GTPase | 7.16 |
| 1045 | GLB1 | Galactosidase Beta 1 | 7.16 |
| 1046 | KL | Klotho | 7.15 |
| 1047 | CYP3A4 | Cytochrome P450 Family 3 Subfamily A Member 4 | 7.15 |
| 1048 | MIR30A | MicroRNA 30a | 7.14 |
| 1049 | PARP1 | Poly(ADP-Ribose) Polymerase 1 | 7.14 |
| 1050 | DRD5 | Dopamine Receptor D5 | 7.13 |
| 1051 | M6PR | Mannose-6-Phosphate Receptor, Cation Dependent | 7.13 |
| 1052 | DDC | Dopa Decarboxylase | 7.13 |
| 1053 | NT5E | 5'-Nucleotidase Ecto | 7.13 |
| 1054 | RNASEH2C | Ribonuclease H2 Subunit C | 7.11 |
| 1055 | BCL2 | BCL2 Apoptosis Regulator | 7.11 |
| 1056 | CXCR3 | C-X-C Motif Chemokine Receptor 3 | 7.09 |
| 1057 | RAB7B | RAB7B, Member RAS Oncogene Family | 7.06 |
| 1058 | IDO1 | Indoleamine 2,3-Dioxygenase 1 | 7.06 |
| 1059 | ADRB2 | Adrenoceptor Beta 2 | 7.05 |
| 1060 | LMNA | Lamin A/C | 7.05 |
| 1061 | MST1R | Macrophage Stimulating 1 Receptor | 7.03 |
| 1062 | PTPN22 | Protein Tyrosine Phosphatase Non-Receptor Type 22 | 7.02 |
| 1063 | TREM1 | Triggering Receptor Expressed On Myeloid Cells 1 | 7.02 |
| 1064 | SAMHD1 | SAM And HD Domain Containing Deoxynucleoside Triphosphate Triphosphohydrolase 1 | 7.01 |
| 1065 | PTPRC | Protein Tyrosine Phosphatase Receptor Type C | 7.01 |
| 1066 | LYST | Lysosomal Trafficking Regulator | 7 |
| 1067 | GJA1 | Gap Junction Protein Alpha 1 | 7 |
| 1068 | AQP1 | Aquaporin 1 (Colton Blood Group) | 7 |
| 1069 | LMX1B | LIM Homeobox Transcription Factor 1 Beta | 6.98 |
| 1070 | CD33 | CD33 Molecule | 6.98 |
| 1071 | TCF4 | Transcription Factor 4 | 6.98 |
| 1072 | CD55 | CD55 Molecule (Cromer Blood Group) | 6.95 |
| 1073 | COL1A1 | Collagen Type I Alpha 1 Chain | 6.94 |
| 1074 | PRCD | Photoreceptor Disc Component | 6.94 |
| 1075 | ANGPTL4 | Angiopoietin Like 4 | 6.93 |
| 1076 | CYBB | Cytochrome B-245 Beta Chain | 6.92 |
| 1077 | DNMT3A | DNA Methyltransferase 3 Alpha | 6.91 |
| 1078 | RNASEH2A | Ribonuclease H2 Subunit A | 6.86 |
| 1079 | CD44 | CD44 Molecule (Indian Blood Group) | 6.85 |
| 1080 | MADD | MAP Kinase Activating Death Domain | 6.84 |
| 1081 | ENPP1 | Ectonucleotide Pyrophosphatase/Phosphodiesterase 1 | 6.83 |
| 1082 | DNASE1L3 | Deoxyribonuclease 1 Like 3 | 6.82 |
| 1083 | ANXA2 | Annexin A2 | 6.81 |
| 1084 | ATP6V1A | ATPase H+ Transporting V1 Subunit A | 6.81 |
| 1085 | SMARCA4 | SWI/SNF Related, Matrix Associated, Actin Dependent Regulator Of Chromatin, Subfamily A, Member 4 | 6.81 |
| 1086 | AFP | Alpha Fetoprotein | 6.81 |
| 1087 | U2AF1 | U2 Small Nuclear RNA Auxiliary Factor 1 | 6.8 |
| 1088 | XK | X-Linked Kx Blood Group | 6.77 |
| 1089 | PRPF6 | Pre-MRNA Processing Factor 6 | 6.76 |
| 1090 | MIR198 | MicroRNA 198 | 6.76 |
| 1091 | EP300 | E1A Binding Protein P300 | 6.76 |
| 1092 | MGMT | O-6-Methylguanine-DNA Methyltransferase | 6.75 |
| 1093 | WDTC1 | WD And Tetratricopeptide Repeats 1 | 6.74 |
| 1094 | TFEB | Transcription Factor EB | 6.74 |
| 1095 | TRIM21 | Tripartite Motif Containing 21 | 6.73 |
| 1096 | NFKBIA | NFKB Inhibitor Alpha | 6.72 |
| 1097 | TBL1X | Transducin Beta Like 1 X-Linked | 6.71 |
| 1098 | RNF216 | Ring Finger Protein 216 | 6.71 |
| 1099 | ITGA2 | Integrin Subunit Alpha 2 | 6.7 |
| 1100 | GNAS | GNAS Complex Locus | 6.7 |
| 1101 | IL6R | Interleukin 6 Receptor | 6.7 |
| 1102 | CISD2 | CDGSH Iron Sulfur Domain 2 | 6.68 |
| 1103 | RHAG | Rh Associated Glycoprotein | 6.68 |
| 1104 | HSPA1A | Heat Shock Protein Family A (Hsp70) Member 1A | 6.68 |
| 1105 | OPRM1 | Opioid Receptor Mu 1 | 6.63 |
| 1106 | RIN2 | Ras And Rab Interactor 2 | 6.63 |
| 1107 | CGA | Glycoprotein Hormones, Alpha Polypeptide | 6.62 |
| 1108 | F13A1 | Coagulation Factor XIII A Chain | 6.61 |
| 1109 | MYH9 | Myosin Heavy Chain 9 | 6.61 |
| 1110 | GALNS | Galactosamine (N-Acetyl)-6-Sulfatase | 6.61 |
| 1111 | CNTNAP2 | Contactin Associated Protein 2 | 6.57 |
| 1112 | GPX1 | Glutathione Peroxidase 1 | 6.56 |
| 1113 | RNASEH2B | Ribonuclease H2 Subunit B | 6.55 |
| 1114 | GLS | Glutaminase | 6.53 |
| 1115 | RPS3A | Ribosomal Protein S3A | 6.5 |
| 1116 | LTA | Lymphotoxin Alpha | 6.49 |
| 1117 | PGK1 | Phosphoglycerate Kinase 1 | 6.49 |
| 1118 | ABCC8 | ATP Binding Cassette Subfamily C Member 8 | 6.47 |
| 1119 | IARS2 | Isoleucyl-TRNA Synthetase 2, Mitochondrial | 6.47 |
| 1120 | GIGYF2 | GRB10 Interacting GYF Protein 2 | 6.44 |
| 1121 | CXCL1 | C-X-C Motif Chemokine Ligand 1 | 6.43 |
| 1122 | CD36 | CD36 Molecule | 6.4 |
| 1123 | MPP5 | Membrane Palmitoylated Protein 5 | 6.4 |
| 1124 | RECK | Reversion Inducing Cysteine Rich Protein With Kazal Motifs | 6.39 |
| 1125 | FXN | Frataxin | 6.39 |
| 1126 | NPTX2 | Neuronal Pentraxin 2 | 6.38 |
| 1127 | CACNA1D | Calcium Voltage-Gated Channel Subunit Alpha1 D | 6.36 |
| 1128 | ITGB1 | Integrin Subunit Beta 1 | 6.36 |
| 1129 | F9 | Coagulation Factor IX | 6.34 |
| 1130 | MT-TC | Mitochondrially Encoded TRNA-Cys (UGU/C) | 6.34 |
| 1131 | CLCN6 | Chloride Voltage-Gated Channel 6 | 6.33 |
| 1132 | LTBP4 | Latent Transforming Growth Factor Beta Binding Protein 4 | 6.32 |
| 1133 | MOG | Myelin Oligodendrocyte Glycoprotein | 6.31 |
| 1134 | XPO1 | Exportin 1 | 6.31 |
| 1135 | PCSK9 | Proprotein Convertase Subtilisin/Kexin Type 9 | 6.31 |
| 1136 | ELANE | Elastase, Neutrophil Expressed | 6.3 |
| 1137 | RAPGEF2 | Rap Guanine Nucleotide Exchange Factor 2 | 6.3 |
| 1138 | LAMP2 | Lysosomal Associated Membrane Protein 2 | 6.3 |
| 1139 | COL18A1 | Collagen Type XVIII Alpha 1 Chain | 6.29 |
| 1140 | HEG1 | Heart Development Protein With EGF Like Domains 1 | 6.29 |
| 1141 | PLA2G1B | Phospholipase A2 Group IB | 6.29 |
| 1142 | AGXT | Alanine--Glyoxylate And Serine--Pyruvate Aminotransferase | 6.27 |
| 1143 | KIAA1549 | KIAA1549 | 6.25 |
| 1144 | IL2RA | Interleukin 2 Receptor Subunit Alpha | 6.24 |
| 1145 | NPLOC4 | NPL4 Homolog, Ubiquitin Recognition Factor | 6.24 |
| 1146 | MIF | Macrophage Migration Inhibitory Factor | 6.23 |
| 1147 | MIR195 | MicroRNA 195 | 6.22 |
| 1148 | ADRA2A | Adrenoceptor Alpha 2A | 6.22 |
| 1149 | MT-RNR1 | Mitochondrially Encoded 12S RRNA | 6.21 |
| 1150 | MIR342 | MicroRNA 342 | 6.21 |
| 1151 | FCGR3B | Fc Fragment Of IgG Receptor IIIb | 6.19 |
| 1152 | ABCB6 | ATP Binding Cassette Subfamily B Member 6 (Langereis Blood Group) | 6.19 |
| 1153 | FRRS1L | Ferric Chelate Reductase 1 Like | 6.19 |
| 1154 | MT-TL2 | Mitochondrially Encoded TRNA-Leu (CUN) 2 | 6.19 |
| 1155 | CXCL9 | C-X-C Motif Chemokine Ligand 9 | 6.19 |
| 1156 | HRH1 | Histamine Receptor H1 | 6.19 |
| 1157 | DDR2 | Discoidin Domain Receptor Tyrosine Kinase 2 | 6.18 |
| 1158 | PLA2G7 | Phospholipase A2 Group VII | 6.18 |
| 1159 | PDYN | Prodynorphin | 6.18 |
| 1160 | RAP2A | RAP2A, Member Of RAS Oncogene Family | 6.17 |
| 1161 | MCOLN1 | Mucolipin 1 | 6.17 |
| 1162 | FOXO1 | Forkhead Box O1 | 6.17 |
| 1163 | CD46 | CD46 Molecule | 6.16 |
| 1164 | GABRG2 | Gamma-Aminobutyric Acid Type A Receptor Subunit Gamma2 | 6.15 |
| 1165 | FGFR2 | Fibroblast Growth Factor Receptor 2 | 6.14 |
| 1166 | GCDH | Glutaryl-CoA Dehydrogenase | 6.14 |
| 1167 | MDH1 | Malate Dehydrogenase 1 | 6.13 |
| 1168 | TLR3 | Toll Like Receptor 3 | 6.12 |
| 1169 | CD79A | CD79a Molecule | 6.12 |
| 1170 | APOL1 | Apolipoprotein L1 | 6.1 |
| 1171 | PODXL | Podocalyxin Like | 6.1 |
| 1172 | DPP4 | Dipeptidyl Peptidase 4 | 6.1 |
| 1173 | MIR20B | MicroRNA 20b | 6.1 |
| 1174 | TSC2 | TSC Complex Subunit 2 | 6.09 |
| 1175 | MAPKAPK3 | MAPK Activated Protein Kinase 3 | 6.09 |
| 1176 | NR4A2 | Nuclear Receptor Subfamily 4 Group A Member 2 | 6.09 |
| 1177 | PLTP | Phospholipid Transfer Protein | 6.09 |
| 1178 | FGFR3 | Fibroblast Growth Factor Receptor 3 | 6.08 |
| 1179 | PTGDS | Prostaglandin D2 Synthase | 6.06 |
| 1180 | CNTN2 | Contactin 2 | 6.05 |
| 1181 | ERBB3 | Erb-B2 Receptor Tyrosine Kinase 3 | 6.04 |
| 1182 | FGF4 | Fibroblast Growth Factor 4 | 6.04 |
| 1183 | ITGB2 | Integrin Subunit Beta 2 | 6.04 |
| 1184 | MIR196A1 | MicroRNA 196a-1 | 6.03 |
| 1185 | RAC1 | Rac Family Small GTPase 1 | 6.02 |
| 1186 | RNU4ATAC | RNA, U4atac Small Nuclear (U12-Dependent Splicing) | 6.02 |
| 1187 | ARR3 | Arrestin 3 | 6.02 |
| 1188 | ADCYAP1 | Adenylate Cyclase Activating Polypeptide 1 | 6.01 |
| 1189 | ROCK1 | Rho Associated Coiled-Coil Containing Protein Kinase 1 | 6 |
| 1190 | PTX3 | Pentraxin 3 | 5.98 |
| 1191 | ICOSLG | Inducible T Cell Costimulator Ligand | 5.96 |
| 1192 | ENO1 | Enolase 1 | 5.95 |
| 1193 | GSTM1 | Glutathione S-Transferase Mu 1 | 5.95 |
| 1194 | TERT | Telomerase Reverse Transcriptase | 5.94 |
| 1195 | H19 | H19 Imprinted Maternally Expressed Transcript | 5.93 |
| 1196 | ARSG | Arylsulfatase G | 5.93 |
| 1197 | GAD2 | Glutamate Decarboxylase 2 | 5.92 |
| 1198 | GPHA2 | Glycoprotein Hormone Subunit Alpha 2 | 5.89 |
| 1199 | NOP53 | NOP53 Ribosome Biogenesis Factor | 5.88 |
| 1200 | MS4A4A | Membrane Spanning 4-Domains A4A | 5.88 |
| 1201 | CDKN1B | Cyclin Dependent Kinase Inhibitor 1B | 5.86 |
| 1202 | APOA2 | Apolipoprotein A2 | 5.86 |
| 1203 | GREM1 | Gremlin 1, DAN Family BMP Antagonist | 5.85 |
| 1204 | SLC6A2 | Solute Carrier Family 6 Member 2 | 5.85 |
| 1205 | CUL2 | Cullin 2 | 5.84 |
| 1206 | TLR8 | Toll Like Receptor 8 | 5.83 |
| 1207 | TLR1 | Toll Like Receptor 1 | 5.83 |
| 1208 | KIAA0319L | KIAA0319 Like | 5.83 |
| 1209 | CDKN1A | Cyclin Dependent Kinase Inhibitor 1A | 5.83 |
| 1210 | SERPINH1 | Serpin Family H Member 1 | 5.83 |
| 1211 | CFI | Complement Factor I | 5.82 |
| 1212 | SPG7 | SPG7 Matrix AAA Peptidase Subunit, Paraplegin | 5.82 |
| 1213 | MAF | MAF BZIP Transcription Factor | 5.82 |
| 1214 | SMN1 | Survival Of Motor Neuron 1, Telomeric | 5.82 |
| 1215 | TCOF1 | Treacle Ribosome Biogenesis Factor 1 | 5.81 |
| 1216 | IL13 | Interleukin 13 | 5.8 |
| 1217 | RET | Ret Proto-Oncogene | 5.79 |
| 1218 | AKT2 | AKT Serine/Threonine Kinase 2 | 5.78 |
| 1219 | IGFBP1 | Insulin Like Growth Factor Binding Protein 1 | 5.78 |
| 1220 | HDAC9 | Histone Deacetylase 9 | 5.77 |
| 1221 | APCS | Amyloid P Component, Serum | 5.76 |
| 1222 | DCX | Doublecortin | 5.76 |
| 1223 | CS | Citrate Synthase | 5.75 |
| 1224 | ULK1 | Unc-51 Like Autophagy Activating Kinase 1 | 5.75 |
| 1225 | SHANK3 | SH3 And Multiple Ankyrin Repeat Domains 3 | 5.74 |
| 1226 | F2RL1 | F2R Like Trypsin Receptor 1 | 5.74 |
| 1227 | SUGCT | Succinyl-CoA:Glutarate-CoA Transferase | 5.71 |
| 1228 | PPBP | Pro-Platelet Basic Protein | 5.7 |
| 1229 | GABRR3 | Gamma-Aminobutyric Acid Type A Receptor Subunit Rho3 | 5.7 |
| 1230 | TLR5 | Toll Like Receptor 5 | 5.7 |
| 1231 | PRKCD | Protein Kinase C Delta | 5.69 |
| 1232 | SLC17A5 | Solute Carrier Family 17 Member 5 | 5.69 |
| 1233 | APEX1 | Apurinic/Apyrimidinic Endodeoxyribonuclease 1 | 5.69 |
| 1234 | WNT10A | Wnt Family Member 10A | 5.69 |
| 1235 | TKT | Transketolase | 5.69 |
| 1236 | HHEX | Hematopoietically Expressed Homeobox | 5.68 |
| 1237 | BMP4 | Bone Morphogenetic Protein 4 | 5.68 |
| 1238 | PIK3CB | Phosphatidylinositol-4,5-Bisphosphate 3-Kinase Catalytic Subunit Beta | 5.68 |
| 1239 | ITGA2B | Integrin Subunit Alpha 2b | 5.68 |
| 1240 | CD9 | CD9 Molecule | 5.68 |
| 1241 | PLAUR | Plasminogen Activator, Urokinase Receptor | 5.66 |
| 1242 | IGF1R | Insulin Like Growth Factor 1 Receptor | 5.65 |
| 1243 | CLDN5 | Claudin 5 | 5.65 |
| 1244 | ALMS1 | ALMS1 Centrosome And Basal Body Associated Protein | 5.64 |
| 1245 | DNAH8 | Dynein Axonemal Heavy Chain 8 | 5.64 |
| 1246 | UBE2D2 | Ubiquitin Conjugating Enzyme E2 D2 | 5.64 |
| 1247 | CCK | Cholecystokinin | 5.63 |
| 1248 | NSF | N-Ethylmaleimide Sensitive Factor, Vesicle Fusing ATPase | 5.63 |
| 1249 | TAF15 | TATA-Box Binding Protein Associated Factor 15 | 5.62 |
| 1250 | PGR | Progesterone Receptor | 5.61 |
| 1251 | JAZF1 | JAZF Zinc Finger 1 | 5.61 |
| 1252 | SDHC | Succinate Dehydrogenase Complex Subunit C | 5.61 |
| 1253 | LTF | Lactotransferrin | 5.61 |
| 1254 | DNMT3B | DNA Methyltransferase 3 Beta | 5.59 |
| 1255 | HK1 | Hexokinase 1 | 5.59 |
| 1256 | SCN8A | Sodium Voltage-Gated Channel Alpha Subunit 8 | 5.58 |
| 1257 | PTK2 | Protein Tyrosine Kinase 2 | 5.57 |
| 1258 | SLC12A2 | Solute Carrier Family 12 Member 2 | 5.57 |
| 1259 | OAT | Ornithine Aminotransferase | 5.57 |
| 1260 | C1QBP | Complement C1q Binding Protein | 5.56 |
| 1261 | PIGW | Phosphatidylinositol Glycan Anchor Biosynthesis Class W | 5.55 |
| 1262 | CLEC7A | C-Type Lectin Domain Containing 7A | 5.54 |
| 1263 | FOXG1 | Forkhead Box G1 | 5.54 |
| 1264 | MIR15B | MicroRNA 15b | 5.54 |
| 1265 | KIF5A | Kinesin Family Member 5A | 5.54 |
| 1266 | TAF1 | TATA-Box Binding Protein Associated Factor 1 | 5.54 |
| 1267 | PLA2G2A | Phospholipase A2 Group IIA | 5.53 |
| 1268 | PLCG1 | Phospholipase C Gamma 1 | 5.53 |
| 1269 | TAT | Tyrosine Aminotransferase | 5.52 |
| 1270 | CCR3 | C-C Motif Chemokine Receptor 3 | 5.52 |
| 1271 | LMF1 | Lipase Maturation Factor 1 | 5.52 |
| 1272 | SGK1 | Serum/Glucocorticoid Regulated Kinase 1 | 5.52 |
| 1273 | SOS1 | SOS Ras/Rac Guanine Nucleotide Exchange Factor 1 | 5.51 |
| 1274 | SCO2 | Synthesis Of Cytochrome C Oxidase 2 | 5.5 |
| 1275 | NAA50 | N-Alpha-Acetyltransferase 50, NatE Catalytic Subunit | 5.5 |
| 1276 | OTX2 | Orthodenticle Homeobox 2 | 5.49 |
| 1277 | CTSL | Cathepsin L | 5.48 |
| 1278 | ITGAX | Integrin Subunit Alpha X | 5.48 |
| 1279 | SMN2 | Survival Of Motor Neuron 2, Centromeric | 5.48 |
| 1280 | SHBG | Sex Hormone Binding Globulin | 5.47 |
| 1281 | YY1 | YY1 Transcription Factor | 5.47 |
| 1282 | KLF2 | Kruppel Like Factor 2 | 5.47 |
| 1283 | RB1 | RB Transcriptional Corepressor 1 | 5.47 |
| 1284 | HOTAIR | HOX Transcript Antisense RNA | 5.45 |
| 1285 | CSTA | Cystatin A | 5.45 |
| 1286 | ISL1 | ISL LIM Homeobox 1 | 5.45 |
| 1287 | UGCG | UDP-Glucose Ceramide Glucosyltransferase | 5.45 |
| 1288 | OLIG2 | Oligodendrocyte Transcription Factor 2 | 5.45 |
| 1289 | GRIA4 | Glutamate Ionotropic Receptor AMPA Type Subunit 4 | 5.44 |
| 1290 | FOLH1 | Folate Hydrolase 1 | 5.44 |
| 1291 | TLR10 | Toll Like Receptor 10 | 5.43 |
| 1292 | ATP6AP2 | ATPase H+ Transporting Accessory Protein 2 | 5.42 |
| 1293 | PSMD4 | Proteasome 26S Subunit, Non-ATPase 4 | 5.42 |
| 1294 | MIRLET7G | MicroRNA Let-7g | 5.4 |
| 1295 | BRCA2 | BRCA2 DNA Repair Associated | 5.39 |
| 1296 | SCG2 | Secretogranin II | 5.39 |
| 1297 | CERS3 | Ceramide Synthase 3 | 5.38 |
| 1298 | MBL2 | Mannose Binding Lectin 2 | 5.38 |
| 1299 | SCP2 | Sterol Carrier Protein 2 | 5.37 |
| 1300 | NAT2 | N-Acetyltransferase 2 | 5.36 |
| 1301 | HDAC4 | Histone Deacetylase 4 | 5.36 |
| 1302 | BLOC1S1 | Biogenesis Of Lysosomal Organelles Complex 1 Subunit 1 | 5.36 |
| 1303 | VCL | Vinculin | 5.36 |
| 1304 | NCAM1 | Neural Cell Adhesion Molecule 1 | 5.36 |
| 1305 | FGF14 | Fibroblast Growth Factor 14 | 5.35 |
| 1306 | FLT3 | Fms Related Receptor Tyrosine Kinase 3 | 5.35 |
| 1307 | GAD1 | Glutamate Decarboxylase 1 | 5.35 |
| 1308 | HDAC1 | Histone Deacetylase 1 | 5.35 |
| 1309 | BGLAP | Bone Gamma-Carboxyglutamate Protein | 5.35 |
| 1310 | PDSS1 | Decaprenyl Diphosphate Synthase Subunit 1 | 5.35 |
| 1311 | SMC3 | Structural Maintenance Of Chromosomes 3 | 5.34 |
| 1312 | DNM2 | Dynamin 2 | 5.33 |
| 1313 | GH1 | Growth Hormone 1 | 5.33 |
| 1314 | MAT2A | Methionine Adenosyltransferase 2A | 5.32 |
| 1315 | ADRA1D | Adrenoceptor Alpha 1D | 5.31 |
| 1316 | FZD1 | Frizzled Class Receptor 1 | 5.31 |
| 1317 | SH3TC2 | SH3 Domain And Tetratricopeptide Repeats 2 | 5.3 |
| 1318 | WNT4 | Wnt Family Member 4 | 5.3 |
| 1319 | SETDB1 | SET Domain Bifurcated Histone Lysine Methyltransferase 1 | 5.29 |
| 1320 | MMP13 | Matrix Metallopeptidase 13 | 5.29 |
| 1321 | TPO | Thyroid Peroxidase | 5.29 |
| 1322 | WNT7B | Wnt Family Member 7B | 5.28 |
| 1323 | PAPPA | Pappalysin 1 | 5.27 |
| 1324 | ATRX | ATRX Chromatin Remodeler | 5.27 |
| 1325 | PLVAP | Plasmalemma Vesicle Associated Protein | 5.27 |
| 1326 | WT1 | WT1 Transcription Factor | 5.27 |
| 1327 | FGB | Fibrinogen Beta Chain | 5.26 |
| 1328 | POLR2A | RNA Polymerase II Subunit A | 5.25 |
| 1329 | SOX18 | SRY-Box Transcription Factor 18 | 5.25 |
| 1330 | CGAS | Cyclic GMP-AMP Synthase | 5.24 |
| 1331 | PES1 | Pescadillo Ribosomal Biogenesis Factor 1 | 5.24 |
| 1332 | GORAB | Golgin, RAB6 Interacting | 5.24 |
| 1333 | EZH2 | Enhancer Of Zeste 2 Polycomb Repressive Complex 2 Subunit | 5.23 |
| 1334 | VAMP2 | Vesicle Associated Membrane Protein 2 | 5.23 |
| 1335 | HTRA2 | HtrA Serine Peptidase 2 | 5.23 |
| 1336 | SRSF2 | Serine And Arginine Rich Splicing Factor 2 | 5.22 |
| 1337 | EPHA3 | EPH Receptor A3 | 5.21 |
| 1338 | CYC1 | Cytochrome C1 | 5.19 |
| 1339 | FLVCR1 | FLVCR Heme Transporter 1 | 5.19 |
| 1340 | HLA-B | Major Histocompatibility Complex, Class I, B | 5.17 |
| 1341 | MMUT | Methylmalonyl-CoA Mutase | 5.17 |
| 1342 | GLI3 | GLI Family Zinc Finger 3 | 5.16 |
| 1343 | SOX10 | SRY-Box Transcription Factor 10 | 5.16 |
| 1344 | XIAP | X-Linked Inhibitor Of Apoptosis | 5.15 |
| 1345 | LIPG | Lipase G, Endothelial Type | 5.15 |
| 1346 | BAMBI | BMP And Activin Membrane Bound Inhibitor | 5.15 |
| 1347 | MIR30B | MicroRNA 30b | 5.15 |
| 1348 | ADRA1B | Adrenoceptor Alpha 1B | 5.14 |
| 1349 | MIR424 | MicroRNA 424 | 5.14 |
| 1350 | CYP27B1 | Cytochrome P450 Family 27 Subfamily B Member 1 | 5.13 |
| 1351 | SPARC | Secreted Protein Acidic And Cysteine Rich | 5.13 |
| 1352 | IGFBP3 | Insulin Like Growth Factor Binding Protein 3 | 5.12 |
| 1353 | BTK | Bruton Tyrosine Kinase | 5.12 |
| 1354 | MPV17 | Mitochondrial Inner Membrane Protein MPV17 | 5.11 |
| 1355 | EXO1 | Exonuclease 1 | 5.11 |
| 1356 | H4-16 | H4 Histone 16 | 5.11 |
| 1357 | RARB | Retinoic Acid Receptor Beta | 5.11 |
| 1358 | TNFSF4 | TNF Superfamily Member 4 | 5.1 |
| 1359 | TUG1 | Taurine Up-Regulated 1 | 5.1 |
| 1360 | SCN9A | Sodium Voltage-Gated Channel Alpha Subunit 9 | 5.1 |
| 1361 | ADAMTSL1 | ADAMTS Like 1 | 5.1 |
| 1362 | TNFSF11 | TNF Superfamily Member 11 | 5.1 |
| 1363 | CD209 | CD209 Molecule | 5.1 |
| 1364 | FH | Fumarate Hydratase | 5.09 |
| 1365 | ADRB3 | Adrenoceptor Beta 3 | 5.09 |
| 1366 | EPG5 | Ectopic P-Granules Autophagy Protein 5 Homolog | 5.09 |
| 1367 | IGFBP7 | Insulin Like Growth Factor Binding Protein 7 | 5.08 |
| 1368 | LGR4 | Leucine Rich Repeat Containing G Protein-Coupled Receptor 4 | 5.08 |
| 1369 | MT-TV | Mitochondrially Encoded TRNA-Val (GUN) | 5.08 |
| 1370 | PIGQ | Phosphatidylinositol Glycan Anchor Biosynthesis Class Q | 5.07 |
| 1371 | THY1 | Thy-1 Cell Surface Antigen | 5.07 |
| 1372 | WNT7A | Wnt Family Member 7A | 5.07 |
| 1373 | WNT3A | Wnt Family Member 3A | 5.07 |
| 1374 | OGA | O-GlcNAcase | 5.06 |
| 1375 | TSPAN2 | Tetraspanin 2 | 5.06 |
| 1376 | ZNF621 | Zinc Finger Protein 621 | 5.06 |
| 1377 | KLHDC7A | Kelch Domain Containing 7A | 5.06 |
| 1378 | PIK3C2A | Phosphatidylinositol-4-Phosphate 3-Kinase Catalytic Subunit Type 2 Alpha | 5.05 |
| 1379 | CTCF | CCCTC-Binding Factor | 5.05 |
| 1380 | UBE3A | Ubiquitin Protein Ligase E3A | 5.04 |
| 1381 | PEX3 | Peroxisomal Biogenesis Factor 3 | 5.04 |
| 1382 | CD38 | CD38 Molecule | 5.04 |
| 1383 | VAPA | VAMP Associated Protein A | 5.03 |
| 1384 | ARVCF | ARVCF Delta Catenin Family Member | 5.02 |
| 1385 | WNT16 | Wnt Family Member 16 | 5.02 |
| 1386 | CFB | Complement Factor B | 5.01 |
| 1387 | ETV6 | ETS Variant Transcription Factor 6 | 5.01 |
| 1388 | DGCR8 | DGCR8 Microprocessor Complex Subunit | 5.01 |
| 1389 | RUNX1 | RUNX Family Transcription Factor 1 | 5.01 |
| 1390 | WNT10B | Wnt Family Member 10B | 5.01 |
| 1391 | DAO | D-Amino Acid Oxidase | 5.01 |
| 1392 | RAB38 | RAB38, Member RAS Oncogene Family | 5.01 |
| 1393 | ABT1 | Activator Of Basal Transcription 1 | 5 |
| 1394 | SMARCA2 | SWI/SNF Related, Matrix Associated, Actin Dependent Regulator Of Chromatin, Subfamily A, Member 2 | 5 |
| 1395 | MFN1 | Mitofusin 1 | 5 |
| 1396 | CD63 | CD63 Molecule | 5 |
| 1397 | IRF3 | Interferon Regulatory Factor 3 | 5 |
| 1398 | ADSL | Adenylosuccinate Lyase | 5 |
| 1399 | ADRB1 | Adrenoceptor Beta 1 | 5 |
| 1400 | ANGPT4 | Angiopoietin 4 | 4.99 |
| 1401 | POU4F1 | POU Class 4 Homeobox 1 | 4.99 |
| 1402 | SPG11 | SPG11 Vesicle Trafficking Associated, Spatacsin | 4.99 |
| 1403 | CYBRD1 | Cytochrome B Reductase 1 | 4.98 |
| 1404 | MMRN1 | Multimerin 1 | 4.98 |
| 1405 | TNFSF13B | TNF Superfamily Member 13b | 4.97 |
| 1406 | CAMP | Cathelicidin Antimicrobial Peptide | 4.96 |
| 1407 | ATP2B3 | ATPase Plasma Membrane Ca2+ Transporting 3 | 4.96 |
| 1408 | SMAD2 | SMAD Family Member 2 | 4.96 |
| 1409 | SUMO1 | Small Ubiquitin Like Modifier 1 | 4.96 |
| 1410 | ARHGEF1 | Rho Guanine Nucleotide Exchange Factor 1 | 4.95 |
| 1411 | MPZ | Myelin Protein Zero | 4.95 |
| 1412 | TLR6 | Toll Like Receptor 6 | 4.95 |
| 1413 | PDP1 | Pyruvate Dehyrogenase Phosphatase Catalytic Subunit 1 | 4.93 |
| 1414 | MIR212 | MicroRNA 212 | 4.93 |
| 1415 | SPP2 | Secreted Phosphoprotein 2 | 4.92 |
| 1416 | SCT | Secretin | 4.92 |
| 1417 | KMT2A | Lysine Methyltransferase 2A | 4.92 |
| 1418 | RPL18 | Ribosomal Protein L18 | 4.92 |
| 1419 | BCL2L1 | BCL2 Like 1 | 4.92 |
| 1420 | UNG | Uracil DNA Glycosylase | 4.91 |
| 1421 | COX10 | Cytochrome C Oxidase Assembly Factor Heme A:Farnesyltransferase COX10 | 4.9 |
| 1422 | IL3 | Interleukin 3 | 4.89 |
| 1423 | FGR | FGR Proto-Oncogene, Src Family Tyrosine Kinase | 4.89 |
| 1424 | MIR103A1 | MicroRNA 103a-1 | 4.88 |
| 1425 | LYN | LYN Proto-Oncogene, Src Family Tyrosine Kinase | 4.88 |
| 1426 | CRYZL1 | Crystallin Zeta Like 1 | 4.87 |
| 1427 | GUSB | Glucuronidase Beta | 4.87 |
| 1428 | PENK | Proenkephalin | 4.87 |
| 1429 | CNOT3 | CCR4-NOT Transcription Complex Subunit 3 | 4.86 |
| 1430 | PRKAG2 | Protein Kinase AMP-Activated Non-Catalytic Subunit Gamma 2 | 4.86 |
| 1431 | WNT11 | Wnt Family Member 11 | 4.85 |
| 1432 | KAT2A | Lysine Acetyltransferase 2A | 4.85 |
| 1433 | LOC642361 | Uncharacterized LOC642361 | 4.84 |
| 1434 | GAN | Gigaxonin | 4.84 |
| 1435 | SLC25A37 | Solute Carrier Family 25 Member 37 | 4.84 |
| 1436 | YIF1A | Yip1 Interacting Factor Homolog A, Membrane Trafficking Protein | 4.84 |
| 1437 | BMP7 | Bone Morphogenetic Protein 7 | 4.84 |
| 1438 | GABARAPL2 | GABA Type A Receptor Associated Protein Like 2 | 4.83 |
| 1439 | NNMT | Nicotinamide N-Methyltransferase | 4.83 |
| 1440 | RSPO1 | R-Spondin 1 | 4.83 |
| 1441 | FIS1 | Fission, Mitochondrial 1 | 4.83 |
| 1442 | GREM2 | Gremlin 2, DAN Family BMP Antagonist | 4.82 |
| 1443 | EPHA4 | EPH Receptor A4 | 4.81 |
| 1444 | INA | Internexin Neuronal Intermediate Filament Protein Alpha | 4.81 |
| 1445 | ISG15 | ISG15 Ubiquitin Like Modifier | 4.81 |
| 1446 | MIRLET7D | MicroRNA Let-7d | 4.8 |
| 1447 | GRIK1 | Glutamate Ionotropic Receptor Kainate Type Subunit 1 | 4.8 |
| 1448 | SWI5 | SWI5 Homologous Recombination Repair Protein | 4.79 |
| 1449 | AKR1B1 | Aldo-Keto Reductase Family 1 Member B | 4.79 |
| 1450 | ROGDI | Rogdi Atypical Leucine Zipper | 4.78 |
| 1451 | TPPP | Tubulin Polymerization Promoting Protein | 4.78 |
| 1452 | LIN7A | Lin-7 Homolog A, Crumbs Cell Polarity Complex Component | 4.78 |
| 1453 | GGCT | Gamma-Glutamylcyclotransferase | 4.78 |
| 1454 | MIR376A1 | MicroRNA 376a-1 | 4.78 |
| 1455 | CDCP2 | CUB Domain Containing Protein 2 | 4.78 |
| 1456 | PPT2 | Palmitoyl-Protein Thioesterase 2 | 4.76 |
| 1457 | AURKA | Aurora Kinase A | 4.76 |
| 1458 | TBXA2R | Thromboxane A2 Receptor | 4.76 |
| 1459 | RUNX2 | RUNX Family Transcription Factor 2 | 4.76 |
| 1460 | CERK | Ceramide Kinase | 4.74 |
| 1461 | AHR | Aryl Hydrocarbon Receptor | 4.74 |
| 1462 | EEF1A1 | Eukaryotic Translation Elongation Factor 1 Alpha 1 | 4.74 |
| 1463 | CARD9 | Caspase Recruitment Domain Family Member 9 | 4.74 |
| 1464 | MMADHC | Metabolism Of Cobalamin Associated D | 4.73 |
| 1465 | CAMK2A | Calcium/Calmodulin Dependent Protein Kinase II Alpha | 4.73 |
| 1466 | SFPQ | Splicing Factor Proline And Glutamine Rich | 4.72 |
| 1467 | COL1A2 | Collagen Type I Alpha 2 Chain | 4.71 |
| 1468 | ADD2 | Adducin 2 | 4.7 |
| 1469 | RARA | Retinoic Acid Receptor Alpha | 4.7 |
| 1470 | TGIF1 | TGFB Induced Factor Homeobox 1 | 4.69 |
| 1471 | F12 | Coagulation Factor XII | 4.69 |
| 1472 | PCBP2 | Poly(RC) Binding Protein 2 | 4.69 |
| 1473 | RASA2 | RAS P21 Protein Activator 2 | 4.68 |
| 1474 | SIGMAR1 | Sigma Non-Opioid Intracellular Receptor 1 | 4.67 |
| 1475 | DCAF8 | DDB1 And CUL4 Associated Factor 8 | 4.67 |
| 1476 | HNF4A | Hepatocyte Nuclear Factor 4 Alpha | 4.67 |
| 1477 | WNT9B | Wnt Family Member 9B | 4.67 |
| 1478 | IQCB1 | IQ Motif Containing B1 | 4.66 |
| 1479 | LRP12 | LDL Receptor Related Protein 12 | 4.66 |
| 1480 | ETS1 | ETS Proto-Oncogene 1, Transcription Factor | 4.65 |
| 1481 | CNMD | Chondromodulin | 4.64 |
| 1482 | TFRC | Transferrin Receptor | 4.64 |
| 1483 | HAMP | Hepcidin Antimicrobial Peptide | 4.64 |
| 1484 | WNT2B | Wnt Family Member 2B | 4.63 |
| 1485 | SCN10A | Sodium Voltage-Gated Channel Alpha Subunit 10 | 4.62 |
| 1486 | ABCD4 | ATP Binding Cassette Subfamily D Member 4 | 4.62 |
| 1487 | POU5F1 | POU Class 5 Homeobox 1 | 4.62 |
| 1488 | LIF | LIF Interleukin 6 Family Cytokine | 4.61 |
| 1489 | CPOX | Coproporphyrinogen Oxidase | 4.61 |
| 1490 | WNT5B | Wnt Family Member 5B | 4.61 |
| 1491 | WNT9A | Wnt Family Member 9A | 4.61 |
| 1492 | ATF6 | Activating Transcription Factor 6 | 4.61 |
| 1493 | TACR1 | Tachykinin Receptor 1 | 4.59 |
| 1494 | SOD3 | Superoxide Dismutase 3 | 4.59 |
| 1495 | BAP1 | BRCA1 Associated Protein 1 | 4.59 |
| 1496 | DGCR2 | DiGeorge Syndrome Critical Region Gene 2 | 4.58 |
| 1497 | WNT8A | Wnt Family Member 8A | 4.58 |
| 1498 | WNT6 | Wnt Family Member 6 | 4.58 |
| 1499 | KIF21A | Kinesin Family Member 21A | 4.58 |
| 1500 | CCT3 | Chaperonin Containing TCP1 Subunit 3 | 4.57 |
| 1501 | LYPD1 | LY6/PLAUR Domain Containing 1 | 4.57 |
| 1502 | ALS2 | Alsin Rho Guanine Nucleotide Exchange Factor ALS2 | 4.57 |
| 1503 | CDC6 | Cell Division Cycle 6 | 4.56 |
| 1504 | HOOK1 | Hook Microtubule Tethering Protein 1 | 4.55 |
| 1505 | PTBP1 | Polypyrimidine Tract Binding Protein 1 | 4.55 |
| 1506 | ADRA2C | Adrenoceptor Alpha 2C | 4.55 |
| 1507 | TMEM70 | Transmembrane Protein 70 | 4.55 |
| 1508 | CDH17 | Cadherin 17 | 4.55 |
| 1509 | TLR9 | Toll Like Receptor 9 | 4.54 |
| 1510 | MYOM2 | Myomesin 2 | 4.53 |
| 1511 | CDC34 | Cell Division Cycle 34, Ubiqiutin Conjugating Enzyme | 4.53 |
| 1512 | FEN1 | Flap Structure-Specific Endonuclease 1 | 4.53 |
| 1513 | CYP1A2 | Cytochrome P450 Family 1 Subfamily A Member 2 | 4.53 |
| 1514 | ALOX12 | Arachidonate 12-Lipoxygenase, 12S Type | 4.53 |
| 1515 | PER2 | Period Circadian Regulator 2 | 4.52 |
| 1516 | COQ2 | Coenzyme Q2, Polyprenyltransferase | 4.52 |
| 1517 | CHD1 | Chromodomain Helicase DNA Binding Protein 1 | 4.51 |
| 1518 | MTHFSD | Methenyltetrahydrofolate Synthetase Domain Containing | 4.51 |
| 1519 | GJB1 | Gap Junction Protein Beta 1 | 4.5 |
| 1520 | KANSL1 | KAT8 Regulatory NSL Complex Subunit 1 | 4.5 |
| 1521 | CFL1 | Cofilin 1 | 4.5 |
| 1522 | HPRT1 | Hypoxanthine Phosphoribosyltransferase 1 | 4.5 |
| 1523 | PRKCH | Protein Kinase C Eta | 4.49 |
| 1524 | PTHLH | Parathyroid Hormone Like Hormone | 4.49 |
| 1525 | LMO2 | LIM Domain Only 2 | 4.49 |
| 1526 | GAS1 | Growth Arrest Specific 1 | 4.48 |
| 1527 | TNNI3 | Troponin I3, Cardiac Type | 4.48 |
| 1528 | GABARAPL1 | GABA Type A Receptor Associated Protein Like 1 | 4.48 |
| 1529 | PDLIM5 | PDZ And LIM Domain 5 | 4.47 |
| 1530 | RBP4 | Retinol Binding Protein 4 | 4.47 |
| 1531 | ALOX5AP | Arachidonate 5-Lipoxygenase Activating Protein | 4.46 |
| 1532 | TPK1 | Thiamin Pyrophosphokinase 1 | 4.46 |
| 1533 | SLC11A1 | Solute Carrier Family 11 Member 1 | 4.46 |
| 1534 | AMPH | Amphiphysin | 4.45 |
| 1535 | CDC14A | Cell Division Cycle 14A | 4.44 |
| 1536 | OPRK1 | Opioid Receptor Kappa 1 | 4.44 |
| 1537 | CDC20 | Cell Division Cycle 20 | 4.44 |
| 1538 | C1orf210 | Chromosome 1 Open Reading Frame 210 | 4.44 |
| 1539 | CUX1 | Cut Like Homeobox 1 | 4.42 |
| 1540 | NRTN | Neurturin | 4.42 |
| 1541 | SLC17A6 | Solute Carrier Family 17 Member 6 | 4.42 |
| 1542 | PON3 | Paraoxonase 3 | 4.41 |
| 1543 | STK39 | Serine/Threonine Kinase 39 | 4.41 |
| 1544 | DCTN2 | Dynactin Subunit 2 | 4.41 |
| 1545 | HBEGF | Heparin Binding EGF Like Growth Factor | 4.41 |
| 1546 | SGCD | Sarcoglycan Delta | 4.41 |
| 1547 | ATG7 | Autophagy Related 7 | 4.41 |
| 1548 | FAR1 | Fatty Acyl-CoA Reductase 1 | 4.4 |
| 1549 | FZD3 | Frizzled Class Receptor 3 | 4.4 |
| 1550 | C1S | Complement C1s | 4.4 |
| 1551 | SLC19A1 | Solute Carrier Family 19 Member 1 | 4.4 |
| 1552 | TRPV1 | Transient Receptor Potential Cation Channel Subfamily V Member 1 | 4.4 |
| 1553 | NDUFV2 | NADH:Ubiquinone Oxidoreductase Core Subunit V2 | 4.4 |
| 1554 | AHSG | Alpha 2-HS Glycoprotein | 4.4 |
| 1555 | TPM3 | Tropomyosin 3 | 4.39 |
| 1556 | KCNJ5 | Potassium Inwardly Rectifying Channel Subfamily J Member 5 | 4.39 |
| 1557 | NSMCE2 | NSE2 (MMS21) Homolog, SMC5-SMC6 Complex SUMO Ligase | 4.38 |
| 1558 | IL5 | Interleukin 5 | 4.38 |
| 1559 | MEG3 | Maternally Expressed 3 | 4.37 |
| 1560 | RBL2 | RB Transcriptional Corepressor Like 2 | 4.36 |
| 1561 | CAMTA1 | Calmodulin Binding Transcription Activator 1 | 4.36 |
| 1562 | KALRN | Kalirin RhoGEF Kinase | 4.36 |
| 1563 | RAB27A | RAB27A, Member RAS Oncogene Family | 4.35 |
| 1564 | APPL1 | Adaptor Protein, Phosphotyrosine Interacting With PH Domain And Leucine Zipper 1 | 4.35 |
| 1565 | GNE | Glucosamine (UDP-N-Acetyl)-2-Epimerase/N-Acetylmannosamine Kinase | 4.35 |
| 1566 | YWHAB | Tyrosine 3-Monooxygenase/Tryptophan 5-Monooxygenase Activation Protein Beta | 4.35 |
| 1567 | SLC2A3 | Solute Carrier Family 2 Member 3 | 4.34 |
| 1568 | UCP1 | Uncoupling Protein 1 | 4.34 |
| 1569 | CCR1 | C-C Motif Chemokine Receptor 1 | 4.33 |
| 1570 | PHOX2A | Paired Like Homeobox 2A | 4.31 |
| 1571 | NPS | Neuropeptide S | 4.31 |
| 1572 | HECTD4 | HECT Domain E3 Ubiquitin Protein Ligase 4 | 4.31 |
| 1573 | ADH5 | Alcohol Dehydrogenase 5 (Class III), Chi Polypeptide | 4.31 |
| 1574 | CUL3 | Cullin 3 | 4.29 |
| 1575 | IL17C | Interleukin 17C | 4.29 |
| 1576 | HPS4 | HPS4 Biogenesis Of Lysosomal Organelles Complex 3 Subunit 2 | 4.29 |
| 1577 | RIPK1 | Receptor Interacting Serine/Threonine Kinase 1 | 4.29 |
| 1578 | ITGA3 | Integrin Subunit Alpha 3 | 4.28 |
| 1579 | DDOST | Dolichyl-Diphosphooligosaccharide--Protein Glycosyltransferase Non-Catalytic Subunit | 4.28 |
| 1580 | TNKS | Tankyrase | 4.28 |
| 1581 | PSMD5 | Proteasome 26S Subunit, Non-ATPase 5 | 4.28 |
| 1582 | IL7 | Interleukin 7 | 4.27 |
| 1583 | MMP12 | Matrix Metallopeptidase 12 | 4.27 |
| 1584 | SDHAF2 | Succinate Dehydrogenase Complex Assembly Factor 2 | 4.26 |
| 1585 | GK | Glycerol Kinase | 4.26 |
| 1586 | GLYAT | Glycine-N-Acyltransferase | 4.26 |
| 1587 | LHX3 | LIM Homeobox 3 | 4.26 |
| 1588 | KIFAP3 | Kinesin Associated Protein 3 | 4.26 |
| 1589 | NEUROG2 | Neurogenin 2 | 4.26 |
| 1590 | CSH1 | Chorionic Somatomammotropin Hormone 1 | 4.26 |
| 1591 | CLEC4E | C-Type Lectin Domain Family 4 Member E | 4.26 |
| 1592 | CLEC6A | C-Type Lectin Domain Containing 6A | 4.26 |
| 1593 | ABHD12 | Abhydrolase Domain Containing 12, Lysophospholipase | 4.24 |
| 1594 | HDAC8 | Histone Deacetylase 8 | 4.24 |
| 1595 | LOC110806262 | Solute Carrier Family 6 Member 4 Gene Promoter | 4.24 |
| 1596 | RYR1 | Ryanodine Receptor 1 | 4.23 |
| 1597 | ICAM2 | Intercellular Adhesion Molecule 2 | 4.23 |
| 1598 | TRH | Thyrotropin Releasing Hormone | 4.23 |
| 1599 | ATOX1 | Antioxidant 1 Copper Chaperone | 4.22 |
| 1600 | ATP5F1B | ATP Synthase F1 Subunit Beta | 4.22 |
| 1601 | SLC8A1 | Solute Carrier Family 8 Member A1 | 4.21 |
| 1602 | ARSB | Arylsulfatase B | 4.21 |
| 1603 | DLEU2 | Deleted In Lymphocytic Leukemia 2 | 4.2 |
| 1604 | TRAF3 | TNF Receptor Associated Factor 3 | 4.2 |
| 1605 | FABP7 | Fatty Acid Binding Protein 7 | 4.2 |
| 1606 | MRPS16 | Mitochondrial Ribosomal Protein S16 | 4.2 |
| 1607 | FSCN1 | Fascin Actin-Bundling Protein 1 | 4.2 |
| 1608 | RAB29 | RAB29, Member RAS Oncogene Family | 4.2 |
| 1609 | YWHAZ | Tyrosine 3-Monooxygenase/Tryptophan 5-Monooxygenase Activation Protein Zeta | 4.2 |
| 1610 | NR4A3 | Nuclear Receptor Subfamily 4 Group A Member 3 | 4.2 |
| 1611 | EFHC2 | EF-Hand Domain Containing 2 | 4.2 |
| 1612 | MMAA | Metabolism Of Cobalamin Associated A | 4.2 |
| 1613 | ATPAF2 | ATP Synthase Mitochondrial F1 Complex Assembly Factor 2 | 4.2 |
| 1614 | GAS6 | Growth Arrest Specific 6 | 4.19 |
| 1615 | TMEM107 | Transmembrane Protein 107 | 4.19 |
| 1616 | ATP5F1E | ATP Synthase F1 Subunit Epsilon | 4.18 |
| 1617 | MDK | Midkine | 4.18 |
| 1618 | SERPINF2 | Serpin Family F Member 2 | 4.17 |
| 1619 | RAB11A | RAB11A, Member RAS Oncogene Family | 4.17 |
| 1620 | POLG2 | DNA Polymerase Gamma 2, Accessory Subunit | 4.17 |
| 1621 | TNFSF10 | TNF Superfamily Member 10 | 4.16 |
| 1622 | LGMN | Legumain | 4.16 |
| 1623 | PRKCG | Protein Kinase C Gamma | 4.16 |
| 1624 | CENPB | Centromere Protein B | 4.16 |
| 1625 | ELAVL4 | ELAV Like RNA Binding Protein 4 | 4.16 |
| 1626 | OPRD1 | Opioid Receptor Delta 1 | 4.16 |
| 1627 | DPYSL5 | Dihydropyrimidinase Like 5 | 4.15 |
| 1628 | C4B_2 | Complement Component 4B (Chido Blood Group), Copy 2 | 4.15 |
| 1629 | CCL21 | C-C Motif Chemokine Ligand 21 | 4.15 |
| 1630 | PRNT | Prion Locus LncRNA, Testis Expressed | 4.15 |
| 1631 | BRCA1 | BRCA1 DNA Repair Associated | 4.14 |
| 1632 | PGAP1 | Post-GPI Attachment To Proteins Inositol Deacylase 1 | 4.14 |
| 1633 | PTK2B | Protein Tyrosine Kinase 2 Beta | 4.14 |
| 1634 | HEPH | Hephaestin | 4.14 |
| 1635 | MUC1 | Mucin 1, Cell Surface Associated | 4.14 |
| 1636 | GRP | Gastrin Releasing Peptide | 4.13 |
| 1637 | HCCS | Holocytochrome C Synthase | 4.13 |
| 1638 | DDHD2 | DDHD Domain Containing 2 | 4.13 |
| 1639 | TNFRSF10A | TNF Receptor Superfamily Member 10a | 4.13 |
| 1640 | SLC2A9 | Solute Carrier Family 2 Member 9 | 4.12 |
| 1641 | SELENBP1 | Selenium Binding Protein 1 | 4.11 |
| 1642 | TTPA | Alpha Tocopherol Transfer Protein | 4.11 |
| 1643 | STX1A | Syntaxin 1A | 4.1 |
| 1644 | CHCHD2 | Coiled-Coil-Helix-Coiled-Coil-Helix Domain Containing 2 | 4.09 |
| 1645 | CACNB2 | Calcium Voltage-Gated Channel Auxiliary Subunit Beta 2 | 4.08 |
| 1646 | IL12RB1 | Interleukin 12 Receptor Subunit Beta 1 | 4.07 |
| 1647 | CCR2 | C-C Motif Chemokine Receptor 2 | 4.07 |
| 1648 | ERCC3 | ERCC Excision Repair 3, TFIIH Core Complex Helicase Subunit | 4.07 |
| 1649 | PAFAH1B1 | Platelet Activating Factor Acetylhydrolase 1b Regulatory Subunit 1 | 4.07 |
| 1650 | MKI67 | Marker Of Proliferation Ki-67 | 4.07 |
| 1651 | PARL | Presenilin Associated Rhomboid Like | 4.06 |
| 1652 | SRCIN1 | SRC Kinase Signaling Inhibitor 1 | 4.06 |
| 1653 | ACAD9 | Acyl-CoA Dehydrogenase Family Member 9 | 4.05 |
| 1654 | ERCC5 | ERCC Excision Repair 5, Endonuclease | 4.04 |
| 1655 | ROCK2 | Rho Associated Coiled-Coil Containing Protein Kinase 2 | 4.04 |
| 1656 | ATP1B1 | ATPase Na+/K+ Transporting Subunit Beta 1 | 4.04 |
| 1657 | KITLG | KIT Ligand | 4.04 |
| 1658 | KCNJ11 | Potassium Inwardly Rectifying Channel Subfamily J Member 11 | 4.03 |
| 1659 | CER1 | Cerberus 1, DAN Family BMP Antagonist | 4.02 |
| 1660 | DAND5 | DAN Domain BMP Antagonist Family Member 5 | 4.02 |
| 1661 | MIR379 | MicroRNA 379 | 4.02 |
| 1662 | PRD | Primary Retinal Dysplasia | 4.02 |
| 1663 | MMD | Monocyte To Macrophage Differentiation Associated | 4.02 |
| 1664 | DDX58 | DExD/H-Box Helicase 58 | 4.01 |
| 1665 | GSS | Glutathione Synthetase | 4.01 |
| 1666 | ACP5 | Acid Phosphatase 5, Tartrate Resistant | 4.01 |
| 1667 | SRSF9 | Serine And Arginine Rich Splicing Factor 9 | 4.01 |
| 1668 | IL16 | Interleukin 16 | 4 |
| 1669 | BNIP3 | BCL2 Interacting Protein 3 | 4 |
| 1670 | TJP2 | Tight Junction Protein 2 | 3.99 |
| 1671 | SMARCA1 | SWI/SNF Related, Matrix Associated, Actin Dependent Regulator Of Chromatin, Subfamily A, Member 1 | 3.98 |
| 1672 | YWHAE | Tyrosine 3-Monooxygenase/Tryptophan 5-Monooxygenase Activation Protein Epsilon | 3.97 |
| 1673 | PDCD6IP | Programmed Cell Death 6 Interacting Protein | 3.97 |
| 1674 | CXCL13 | C-X-C Motif Chemokine Ligand 13 | 3.97 |
| 1675 | RXRB | Retinoid X Receptor Beta | 3.97 |
| 1676 | ABCC9 | ATP Binding Cassette Subfamily C Member 9 | 3.96 |
| 1677 | SLC31A1 | Solute Carrier Family 31 Member 1 | 3.96 |
| 1678 | CHMP4B | Charged Multivesicular Body Protein 4B | 3.96 |
| 1679 | NR3C2 | Nuclear Receptor Subfamily 3 Group C Member 2 | 3.95 |
| 1680 | IL10RA | Interleukin 10 Receptor Subunit Alpha | 3.95 |
| 1681 | SPON1 | Spondin 1 | 3.94 |
| 1682 | MIR200B | MicroRNA 200b | 3.94 |
| 1683 | APOA5 | Apolipoprotein A5 | 3.94 |
| 1684 | IL15 | Interleukin 15 | 3.94 |
| 1685 | CNNM2 | Cyclin And CBS Domain Divalent Metal Cation Transport Mediator 2 | 3.93 |
| 1686 | SOX3 | SRY-Box Transcription Factor 3 | 3.93 |
| 1687 | NEDD4L | NEDD4 Like E3 Ubiquitin Protein Ligase | 3.93 |
| 1688 | GABBR1 | Gamma-Aminobutyric Acid Type B Receptor Subunit 1 | 3.93 |
| 1689 | BNIP3L | BCL2 Interacting Protein 3 Like | 3.93 |
| 1690 | COX15 | Cytochrome C Oxidase Assembly Homolog COX15 | 3.92 |
| 1691 | KIF3A | Kinesin Family Member 3A | 3.91 |
| 1692 | PDPK1 | 3-Phosphoinositide Dependent Protein Kinase 1 | 3.91 |
| 1693 | PNKP | Polynucleotide Kinase 3'-Phosphatase | 3.91 |
| 1694 | TNNT2 | Troponin T2, Cardiac Type | 3.91 |
| 1695 | MLXIPL | MLX Interacting Protein Like | 3.91 |
| 1696 | OTOF | Otoferlin | 3.9 |
| 1697 | OPA3 | Outer Mitochondrial Membrane Lipid Metabolism Regulator OPA3 | 3.9 |
| 1698 | DROSHA | Drosha Ribonuclease III | 3.89 |
| 1699 | KRT7 | Keratin 7 | 3.89 |
| 1700 | PPP2CA | Protein Phosphatase 2 Catalytic Subunit Alpha | 3.89 |
| 1701 | MLN | Motilin | 3.88 |
| 1702 | LGALS1 | Galectin 1 | 3.88 |
| 1703 | IDS | Iduronate 2-Sulfatase | 3.88 |
| 1704 | GAL3ST1 | Galactose-3-O-Sulfotransferase 1 | 3.88 |
| 1705 | NR1H2 | Nuclear Receptor Subfamily 1 Group H Member 2 | 3.87 |
| 1706 | UBE2D1 | Ubiquitin Conjugating Enzyme E2 D1 | 3.87 |
| 1707 | RMC1 | Regulator Of MON1-CCZ1 | 3.86 |
| 1708 | GRAP2 | GRB2 Related Adaptor Protein 2 | 3.86 |
| 1709 | ARHGEF10 | Rho Guanine Nucleotide Exchange Factor 10 | 3.86 |
| 1710 | RING1 | Ring Finger Protein 1 | 3.86 |
| 1711 | ARIH1 | Ariadne RBR E3 Ubiquitin Protein Ligase 1 | 3.86 |
| 1712 | THAP1 | THAP Domain Containing 1 | 3.86 |
| 1713 | POTEF | POTE Ankyrin Domain Family Member F | 3.86 |
| 1714 | MIR331 | MicroRNA 331 | 3.86 |
| 1715 | PSMB8 | Proteasome 20S Subunit Beta 8 | 3.84 |
| 1716 | SLC12A6 | Solute Carrier Family 12 Member 6 | 3.83 |
| 1717 | LGALS3 | Galectin 3 | 3.82 |
| 1718 | RGS2 | Regulator Of G Protein Signaling 2 | 3.82 |
| 1719 | BRD4 | Bromodomain Containing 4 | 3.81 |
| 1720 | COX17 | Cytochrome C Oxidase Copper Chaperone COX17 | 3.81 |
| 1721 | COMP | Cartilage Oligomeric Matrix Protein | 3.8 |
| 1722 | MX1 | MX Dynamin Like GTPase 1 | 3.79 |
| 1723 | CCL11 | C-C Motif Chemokine Ligand 11 | 3.79 |
| 1724 | LCN2 | Lipocalin 2 | 3.78 |
| 1725 | ABL1 | ABL Proto-Oncogene 1, Non-Receptor Tyrosine Kinase | 3.77 |
| 1726 | SACS | Sacsin Molecular Chaperone | 3.76 |
| 1727 | PDE10A | Phosphodiesterase 10A | 3.76 |
| 1728 | OTC | Ornithine Carbamoyltransferase | 3.75 |
| 1729 | PTPN1 | Protein Tyrosine Phosphatase Non-Receptor Type 1 | 3.74 |
| 1730 | FGG | Fibrinogen Gamma Chain | 3.73 |
| 1731 | SGCE | Sarcoglycan Epsilon | 3.73 |
| 1732 | EGR2 | Early Growth Response 2 | 3.72 |
| 1733 | DDB2 | Damage Specific DNA Binding Protein 2 | 3.71 |
| 1734 | YWHAG | Tyrosine 3-Monooxygenase/Tryptophan 5-Monooxygenase Activation Protein Gamma | 3.71 |
| 1735 | GHRH | Growth Hormone Releasing Hormone | 3.7 |
| 1736 | ESS2 | Ess-2 Splicing Factor Homolog | 3.7 |
| 1737 | TNFAIP3 | TNF Alpha Induced Protein 3 | 3.7 |
| 1738 | MIR106A | MicroRNA 106a | 3.7 |
| 1739 | FERD3L | Fer3 Like BHLH Transcription Factor | 3.69 |
| 1740 | RAPGEF3 | Rap Guanine Nucleotide Exchange Factor 3 | 3.68 |
| 1741 | LMNB1 | Lamin B1 | 3.68 |
| 1742 | ID1 | Inhibitor Of DNA Binding 1, HLH Protein | 3.68 |
| 1743 | GNAL | G Protein Subunit Alpha L | 3.68 |
| 1744 | UBQLN4 | Ubiquilin 4 | 3.68 |
| 1745 | IRF4 | Interferon Regulatory Factor 4 | 3.67 |
| 1746 | PEX7 | Peroxisomal Biogenesis Factor 7 | 3.66 |
| 1747 | SLC1A1 | Solute Carrier Family 1 Member 1 | 3.65 |
| 1748 | BCS1L | BCS1 Homolog, Ubiquinol-Cytochrome C Reductase Complex Chaperone | 3.64 |
| 1749 | DNAJB6 | DnaJ Heat Shock Protein Family (Hsp40) Member B6 | 3.64 |
| 1750 | SREBF2 | Sterol Regulatory Element Binding Transcription Factor 2 | 3.64 |
| 1751 | CUBN | Cubilin | 3.64 |
| 1752 | HSPB6 | Heat Shock Protein Family B (Small) Member 6 | 3.63 |
| 1753 | YME1L1 | YME1 Like 1 ATPase | 3.63 |
| 1754 | ZDHHC8 | Zinc Finger DHHC-Type Palmitoyltransferase 8 | 3.63 |
| 1755 | BICD2 | BICD Cargo Adaptor 2 | 3.63 |
| 1756 | COX14 | Cytochrome C Oxidase Assembly Factor COX14 | 3.63 |
| 1757 | AGTR2 | Angiotensin II Receptor Type 2 | 3.62 |
| 1758 | HSPB2 | Heat Shock Protein Family B (Small) Member 2 | 3.62 |
| 1759 | MTHFD1L | Methylenetetrahydrofolate Dehydrogenase (NADP+ Dependent) 1 Like | 3.62 |
| 1760 | IL1R1 | Interleukin 1 Receptor Type 1 | 3.62 |
| 1761 | ETFDH | Electron Transfer Flavoprotein Dehydrogenase | 3.62 |
| 1762 | MTX1 | Metaxin 1 | 3.62 |
| 1763 | DCT | Dopachrome Tautomerase | 3.62 |
| 1764 | MIR346 | MicroRNA 346 | 3.61 |
| 1765 | GLO1 | Glyoxalase I | 3.61 |
| 1766 | LBR | Lamin B Receptor | 3.61 |
| 1767 | DCAF17 | DDB1 And CUL4 Associated Factor 17 | 3.61 |
| 1768 | PDIA6 | Protein Disulfide Isomerase Family A Member 6 | 3.59 |
| 1769 | PRKCE | Protein Kinase C Epsilon | 3.59 |
| 1770 | FADD | Fas Associated Via Death Domain | 3.59 |
| 1771 | ALPL | Alkaline Phosphatase, Biomineralization Associated | 3.58 |
| 1772 | VPS54 | VPS54 Subunit Of GARP Complex | 3.58 |
| 1773 | SRSF6 | Serine And Arginine Rich Splicing Factor 6 | 3.58 |
| 1774 | ACAD8 | Acyl-CoA Dehydrogenase Family Member 8 | 3.58 |
| 1775 | RO60 | Ro60, Y RNA Binding Protein | 3.57 |
| 1776 | CDK2 | Cyclin Dependent Kinase 2 | 3.57 |
| 1777 | L1CAM | L1 Cell Adhesion Molecule | 3.56 |
| 1778 | IDH1 | Isocitrate Dehydrogenase (NADP(+)) 1 | 3.56 |
| 1779 | HTR3A | 5-Hydroxytryptamine Receptor 3A | 3.56 |
| 1780 | SMARCD2 | SWI/SNF Related, Matrix Associated, Actin Dependent Regulator Of Chromatin, Subfamily D, Member 2 | 3.56 |
| 1781 | SLC32A1 | Solute Carrier Family 32 Member 1 | 3.56 |
| 1782 | POLRMT | RNA Polymerase Mitochondrial | 3.56 |
| 1783 | NR4A1 | Nuclear Receptor Subfamily 4 Group A Member 1 | 3.55 |
| 1784 | PCSK6 | Proprotein Convertase Subtilisin/Kexin Type 6 | 3.55 |
| 1785 | XPA | XPA, DNA Damage Recognition And Repair Factor | 3.53 |
| 1786 | TRAPPC9 | Trafficking Protein Particle Complex 9 | 3.53 |
| 1787 | TRPM8 | Transient Receptor Potential Cation Channel Subfamily M Member 8 | 3.53 |
| 1788 | TRPM6 | Transient Receptor Potential Cation Channel Subfamily M Member 6 | 3.52 |
| 1789 | CA2 | Carbonic Anhydrase 2 | 3.52 |
| 1790 | FGGY | FGGY Carbohydrate Kinase Domain Containing | 3.52 |
| 1791 | PDE5A | Phosphodiesterase 5A | 3.51 |
| 1792 | ZKSCAN3 | Zinc Finger With KRAB And SCAN Domains 3 | 3.51 |
| 1793 | CD2AP | CD2 Associated Protein | 3.5 |
| 1794 | SMCR8 | SMCR8-C9orf72 Complex Subunit | 3.5 |
| 1795 | PRKD1 | Protein Kinase D1 | 3.5 |
| 1796 | RTN4R | Reticulon 4 Receptor | 3.49 |
| 1797 | SCARB1 | Scavenger Receptor Class B Member 1 | 3.49 |
| 1798 | HTR1B | 5-Hydroxytryptamine Receptor 1B | 3.48 |
| 1799 | OXTR | Oxytocin Receptor | 3.48 |
| 1800 | CST6 | Cystatin E/M | 3.48 |
| 1801 | ANXA11 | Annexin A11 | 3.47 |
| 1802 | SALL1 | Spalt Like Transcription Factor 1 | 3.46 |
| 1803 | SRCAP | Snf2 Related CREBBP Activator Protein | 3.45 |
| 1804 | PDGFC | Platelet Derived Growth Factor C | 3.45 |
| 1805 | HIP1 | Huntingtin Interacting Protein 1 | 3.44 |
| 1806 | SLC17A7 | Solute Carrier Family 17 Member 7 | 3.44 |
| 1807 | GARS1 | Glycyl-TRNA Synthetase 1 | 3.44 |
| 1808 | HDAC6 | Histone Deacetylase 6 | 3.43 |
| 1809 | DISP1 | Dispatched RND Transporter Family Member 1 | 3.43 |
| 1810 | HRH3 | Histamine Receptor H3 | 3.43 |
| 1811 | SLC18A1 | Solute Carrier Family 18 Member A1 | 3.43 |
| 1812 | MIR330 | MicroRNA 330 | 3.43 |
| 1813 | MIR206 | MicroRNA 206 | 3.42 |
| 1814 | MRE11 | MRE11 Homolog, Double Strand Break Repair Nuclease | 3.42 |
| 1815 | CEP78 | Centrosomal Protein 78 | 3.42 |
| 1816 | IRF1 | Interferon Regulatory Factor 1 | 3.42 |
| 1817 | ACADS | Acyl-CoA Dehydrogenase Short Chain | 3.42 |
| 1818 | HPS5 | HPS5 Biogenesis Of Lysosomal Organelles Complex 2 Subunit 2 | 3.42 |
| 1819 | GAMT | Guanidinoacetate N-Methyltransferase | 3.41 |
| 1820 | PRKACA | Protein Kinase CAMP-Activated Catalytic Subunit Alpha | 3.41 |
| 1821 | HLA-G | Major Histocompatibility Complex, Class I, G | 3.41 |
| 1822 | GPIHBP1 | Glycosylphosphatidylinositol Anchored High Density Lipoprotein Binding Protein 1 | 3.41 |
| 1823 | C5AR1 | Complement C5a Receptor 1 | 3.4 |
| 1824 | GABRA1 | Gamma-Aminobutyric Acid Type A Receptor Subunit Alpha1 | 3.4 |
| 1825 | KMO | Kynurenine 3-Monooxygenase | 3.4 |
| 1826 | CST7 | Cystatin F | 3.4 |
| 1827 | SLN | Sarcolipin | 3.4 |
| 1828 | GLE1 | GLE1 RNA Export Mediator | 3.4 |
| 1829 | MAN2B1 | Mannosidase Alpha Class 2B Member 1 | 3.4 |
| 1830 | VDR | Vitamin D Receptor | 3.39 |
| 1831 | TG | Thyroglobulin | 3.39 |
| 1832 | KLK3 | Kallikrein Related Peptidase 3 | 3.39 |
| 1833 | GNS | Glucosamine (N-Acetyl)-6-Sulfatase | 3.38 |
| 1834 | TGM2 | Transglutaminase 2 | 3.38 |
| 1835 | ETFA | Electron Transfer Flavoprotein Subunit Alpha | 3.38 |
| 1836 | GDAP1 | Ganglioside Induced Differentiation Associated Protein 1 | 3.38 |
| 1837 | GABRR2 | Gamma-Aminobutyric Acid Type A Receptor Subunit Rho2 | 3.38 |
| 1838 | MBD1 | Methyl-CpG Binding Domain Protein 1 | 3.38 |
| 1839 | HOMER1 | Homer Scaffold Protein 1 | 3.38 |
| 1840 | IFT57 | Intraflagellar Transport 57 | 3.38 |
| 1841 | APEX2 | Apurinic/Apyrimidinic Endodeoxyribonuclease 2 | 3.38 |
| 1842 | OMD | Osteomodulin | 3.38 |
| 1843 | CST4 | Cystatin S | 3.38 |
| 1844 | ADGRA3 | Adhesion G Protein-Coupled Receptor A3 | 3.38 |
| 1845 | HMX2 | H6 Family Homeobox 2 | 3.38 |
| 1846 | PROCA1 | Protein Interacting With Cyclin A1 | 3.38 |
| 1847 | DGCR5 | DiGeorge Syndrome Critical Region Gene 5 | 3.38 |
| 1848 | MIR30D | MicroRNA 30d | 3.38 |
| 1849 | IL18R1 | Interleukin 18 Receptor 1 | 3.38 |
| 1850 | DGKE | Diacylglycerol Kinase Epsilon | 3.38 |
| 1851 | ELAVL1 | ELAV Like RNA Binding Protein 1 | 3.37 |
| 1852 | ACKR1 | Atypical Chemokine Receptor 1 (Duffy Blood Group) | 3.37 |
| 1853 | DUSP1 | Dual Specificity Phosphatase 1 | 3.37 |
| 1854 | POLR2L | RNA Polymerase II, I And III Subunit L | 3.36 |
| 1855 | IL12A | Interleukin 12A | 3.36 |
| 1856 | DUSP19 | Dual Specificity Phosphatase 19 | 3.36 |
| 1857 | ZUP1 | Zinc Finger Containing Ubiquitin Peptidase 1 | 3.35 |
| 1858 | PPIA | Peptidylprolyl Isomerase A | 3.35 |
| 1859 | IFNAR1 | Interferon Alpha And Beta Receptor Subunit 1 | 3.34 |
| 1860 | ARNTL | Aryl Hydrocarbon Receptor Nuclear Translocator Like | 3.33 |
| 1861 | RCVRN | Recoverin | 3.33 |
| 1862 | RERE | Arginine-Glutamic Acid Dipeptide Repeats | 3.33 |
| 1863 | NMNAT1 | Nicotinamide Nucleotide Adenylyltransferase 1 | 3.33 |
| 1864 | GPM6B | Glycoprotein M6B | 3.33 |
| 1865 | H1-1 | H1.1 Linker Histone, Cluster Member | 3.33 |
| 1866 | MIR99A | MicroRNA 99a | 3.33 |
| 1867 | SLC13A4 | Solute Carrier Family 13 Member 4 | 3.33 |
| 1868 | PDE3A | Phosphodiesterase 3A | 3.32 |
| 1869 | SLC25A24 | Solute Carrier Family 25 Member 24 | 3.31 |
| 1870 | TRPA1 | Transient Receptor Potential Cation Channel Subfamily A Member 1 | 3.31 |
| 1871 | CACNA1E | Calcium Voltage-Gated Channel Subunit Alpha1 E | 3.31 |
| 1872 | VKORC1 | Vitamin K Epoxide Reductase Complex Subunit 1 | 3.3 |
| 1873 | ADIRF | Adipogenesis Regulatory Factor | 3.3 |
| 1874 | CACNA1G | Calcium Voltage-Gated Channel Subunit Alpha1 G | 3.3 |
| 1875 | OPRL1 | Opioid Related Nociceptin Receptor 1 | 3.3 |
| 1876 | STIM1 | Stromal Interaction Molecule 1 | 3.29 |
| 1877 | SMARCE1 | SWI/SNF Related, Matrix Associated, Actin Dependent Regulator Of Chromatin, Subfamily E, Member 1 | 3.29 |
| 1878 | GRB2 | Growth Factor Receptor Bound Protein 2 | 3.29 |
| 1879 | HNRNPH1 | Heterogeneous Nuclear Ribonucleoprotein H1 | 3.29 |
| 1880 | RPL34 | Ribosomal Protein L34 | 3.28 |
| 1881 | MLNR | Motilin Receptor | 3.28 |
| 1882 | LIN9 | Lin-9 DREAM MuvB Core Complex Component | 3.28 |
| 1883 | JRK | Jrk Helix-Turn-Helix Protein | 3.28 |
| 1884 | FBXL3 | F-Box And Leucine Rich Repeat Protein 3 | 3.27 |
| 1885 | TMEM14A | Transmembrane Protein 14A | 3.27 |
| 1886 | ASAH2 | N-Acylsphingosine Amidohydrolase 2 | 3.27 |
| 1887 | AVEN | Apoptosis And Caspase Activation Inhibitor | 3.26 |
| 1888 | SIRT3 | Sirtuin 3 | 3.26 |
| 1889 | HSP90B1 | Heat Shock Protein 90 Beta Family Member 1 | 3.25 |
| 1890 | NMB | Neuromedin B | 3.25 |
| 1891 | NCF1 | Neutrophil Cytosolic Factor 1 | 3.25 |
| 1892 | TRPC1 | Transient Receptor Potential Cation Channel Subfamily C Member 1 | 3.24 |
| 1893 | BIRC3 | Baculoviral IAP Repeat Containing 3 | 3.24 |
| 1894 | CFAP47 | Cilia And Flagella Associated Protein 47 | 3.24 |
| 1895 | HPSE | Heparanase | 3.24 |
| 1896 | HTR1D | 5-Hydroxytryptamine Receptor 1D | 3.24 |
| 1897 | SCO1 | Synthesis Of Cytochrome C Oxidase 1 | 3.24 |
| 1898 | CYP2J2 | Cytochrome P450 Family 2 Subfamily J Member 2 | 3.23 |
| 1899 | KIF1C | Kinesin Family Member 1C | 3.23 |
| 1900 | HLA-DRB3 | Major Histocompatibility Complex, Class II, DR Beta 3 | 3.23 |
| 1901 | GYPA | Glycophorin A (MNS Blood Group) | 3.23 |
| 1902 | SERPING1 | Serpin Family G Member 1 | 3.23 |
| 1903 | MMP7 | Matrix Metallopeptidase 7 | 3.22 |
| 1904 | TERC | Telomerase RNA Component | 3.22 |
| 1905 | CASR | Calcium Sensing Receptor | 3.22 |
| 1906 | IREB2 | Iron Responsive Element Binding Protein 2 | 3.21 |
| 1907 | TPPP3 | Tubulin Polymerization Promoting Protein Family Member 3 | 3.21 |
| 1908 | POLD1 | DNA Polymerase Delta 1, Catalytic Subunit | 3.21 |
| 1909 | NOL3 | Nucleolar Protein 3 | 3.2 |
| 1910 | HLA-A | Major Histocompatibility Complex, Class I, A | 3.2 |
| 1911 | L2HGDH | L-2-Hydroxyglutarate Dehydrogenase | 3.19 |
| 1912 | ATF2 | Activating Transcription Factor 2 | 3.19 |
| 1913 | HPGD | 15-Hydroxyprostaglandin Dehydrogenase | 3.18 |
| 1914 | MS4A6E | Membrane Spanning 4-Domains A6E | 3.18 |
| 1915 | ACTA1 | Actin Alpha 1, Skeletal Muscle | 3.18 |
| 1916 | PANK4 | Pantothenate Kinase 4 (Inactive) | 3.18 |
| 1917 | JMJD1C | Jumonji Domain Containing 1C | 3.18 |
| 1918 | CDKAL1 | CDK5 Regulatory Subunit Associated Protein 1 Like 1 | 3.17 |
| 1919 | RBM12 | RNA Binding Motif Protein 12 | 3.17 |
| 1920 | BTNL2 | Butyrophilin Like 2 | 3.16 |
| 1921 | TRPM2 | Transient Receptor Potential Cation Channel Subfamily M Member 2 | 3.15 |
| 1922 | CYP11B2 | Cytochrome P450 Family 11 Subfamily B Member 2 | 3.15 |
| 1923 | ARL6IP5 | ADP Ribosylation Factor Like GTPase 6 Interacting Protein 5 | 3.15 |
| 1924 | PLD1 | Phospholipase D1 | 3.14 |
| 1925 | ADNP | Activity Dependent Neuroprotector Homeobox | 3.14 |
| 1926 | ELOVL4 | ELOVL Fatty Acid Elongase 4 | 3.14 |
| 1927 | VPS13B | Vacuolar Protein Sorting 13 Homolog B | 3.14 |
| 1928 | SEL1L | SEL1L Adaptor Subunit Of ERAD E3 Ubiquitin Ligase | 3.14 |
| 1929 | AP1G1 | Adaptor Related Protein Complex 1 Subunit Gamma 1 | 3.14 |
| 1930 | RILPL1 | Rab Interacting Lysosomal Protein Like 1 | 3.14 |
| 1931 | SPCS2 | Signal Peptidase Complex Subunit 2 | 3.14 |
| 1932 | HEXB | Hexosaminidase Subunit Beta | 3.14 |
| 1933 | CYB5R3 | Cytochrome B5 Reductase 3 | 3.14 |
| 1934 | YAP1 | Yes1 Associated Transcriptional Regulator | 3.13 |
| 1935 | ABCC1 | ATP Binding Cassette Subfamily C Member 1 | 3.13 |
| 1936 | PEMT | Phosphatidylethanolamine N-Methyltransferase | 3.13 |
| 1937 | NOP56 | NOP56 Ribonucleoprotein | 3.12 |
| 1938 | IL12B | Interleukin 12B | 3.12 |
| 1939 | CYB5A | Cytochrome B5 Type A | 3.12 |
| 1940 | PTPRN | Protein Tyrosine Phosphatase Receptor Type N | 3.12 |
| 1941 | PRX | Periaxin | 3.12 |
| 1942 | TMEM11 | Transmembrane Protein 11 | 3.12 |
| 1943 | VCPKMT | Valosin Containing Protein Lysine Methyltransferase | 3.12 |
| 1944 | FOXP2 | Forkhead Box P2 | 3.11 |
| 1945 | PEX12 | Peroxisomal Biogenesis Factor 12 | 3.11 |
| 1946 | GHSR | Growth Hormone Secretagogue Receptor | 3.11 |
| 1947 | GSTP1 | Glutathione S-Transferase Pi 1 | 3.1 |
| 1948 | SLC19A3 | Solute Carrier Family 19 Member 3 | 3.09 |
| 1949 | KIF1A | Kinesin Family Member 1A | 3.09 |
| 1950 | CYB5B | Cytochrome B5 Type B | 3.09 |
| 1951 | TBCK | TBC1 Domain Containing Kinase | 3.09 |
| 1952 | CST5 | Cystatin D | 3.09 |
| 1953 | STX8 | Syntaxin 8 | 3.09 |
| 1954 | PLP2 | Proteolipid Protein 2 | 3.09 |
| 1955 | CST9L | Cystatin 9 Like | 3.09 |
| 1956 | RILP | Rab Interacting Lysosomal Protein | 3.09 |
| 1957 | RRP15 | Ribosomal RNA Processing 15 Homolog | 3.09 |
| 1958 | PTCD1 | Pentatricopeptide Repeat Domain 1 | 3.09 |
| 1959 | TMEM147 | Transmembrane Protein 147 | 3.09 |
| 1960 | YIPF6 | Yip1 Domain Family Member 6 | 3.09 |
| 1961 | TEFM | Transcription Elongation Factor, Mitochondrial | 3.09 |
| 1962 | MRO | Maestro | 3.09 |
| 1963 | TMEM128 | Transmembrane Protein 128 | 3.09 |
| 1964 | TMEM134 | Transmembrane Protein 134 | 3.09 |
| 1965 | ATP5MC1 | ATP Synthase Membrane Subunit C Locus 1 | 3.09 |
| 1966 | ERG28 | Ergosterol Biosynthesis 28 Homolog | 3.09 |
| 1967 | SELENOK | Selenoprotein K | 3.09 |
| 1968 | ABHD18 | Abhydrolase Domain Containing 18 | 3.09 |
| 1969 | LOC101927752 | Uncharacterized LOC101927752 | 3.09 |
| 1970 | DACT1 | Dishevelled Binding Antagonist Of Beta Catenin 1 | 3.09 |
| 1971 | KAT2B | Lysine Acetyltransferase 2B | 3.09 |
| 1972 | DHPS | Deoxyhypusine Synthase | 3.09 |
| 1973 | GYPC | Glycophorin C (Gerbich Blood Group) | 3.08 |
| 1974 | NQO2 | N-Ribosyldihydronicotinamide:Quinone Reductase 2 | 3.07 |
| 1975 | SYN2 | Synapsin II | 3.07 |
| 1976 | TYMS | Thymidylate Synthetase | 3.07 |
| 1977 | HNRNPH2 | Heterogeneous Nuclear Ribonucleoprotein H2 | 3.07 |
| 1978 | IKBKB | Inhibitor Of Nuclear Factor Kappa B Kinase Subunit Beta | 3.06 |
| 1979 | MEG8 | Maternally Expressed 8, Small Nucleolar RNA Host Gene | 3.06 |
| 1980 | AMBP | Alpha-1-Microglobulin/Bikunin Precursor | 3.04 |
| 1981 | MAP3K3 | Mitogen-Activated Protein Kinase Kinase Kinase 3 | 3.03 |
| 1982 | TCF7L2 | Transcription Factor 7 Like 2 | 3.03 |
| 1983 | PLOD1 | Procollagen-Lysine,2-Oxoglutarate 5-Dioxygenase 1 | 3.02 |
| 1984 | PDE4A | Phosphodiesterase 4A | 3.02 |
| 1985 | CDH13 | Cadherin 13 | 3.02 |
| 1986 | BCAR1 | BCAR1 Scaffold Protein, Cas Family Member | 3.02 |
| 1987 | PRICKLE1 | Prickle Planar Cell Polarity Protein 1 | 3.02 |
| 1988 | FMN2 | Formin 2 | 3.01 |
| 1989 | CGB7 | Chorionic Gonadotropin Subunit Beta 7 | 3.01 |
| 1990 | IL1RAPL2 | Interleukin 1 Receptor Accessory Protein Like 2 | 3.01 |
| 1991 | TNC | Tenascin C | 3.01 |
| 1992 | PTRH2 | Peptidyl-TRNA Hydrolase 2 | 3.01 |
| 1993 | SOCS3 | Suppressor Of Cytokine Signaling 3 | 3 |
| 1994 | NFATC1 | Nuclear Factor Of Activated T Cells 1 | 3 |
| 1995 | NFKB2 | Nuclear Factor Kappa B Subunit 2 | 3 |
| 1996 | FAF2 | Fas Associated Factor Family Member 2 | 2.99 |
| 1997 | CD14 | CD14 Molecule | 2.99 |
| 1998 | RPGRIP1L | RPGRIP1 Like | 2.99 |
| 1999 | CSK | C-Terminal Src Kinase | 2.98 |
| 2000 | TRPM4 | Transient Receptor Potential Cation Channel Subfamily M Member 4 | 2.98 |
| 2001 | NPM1 | Nucleophosmin 1 | 2.98 |
| 2002 | CLEC4M | C-Type Lectin Domain Family 4 Member M | 2.98 |
| 2003 | ALPP | Alkaline Phosphatase, Placental | 2.97 |
| 2004 | MIR574 | MicroRNA 574 | 2.96 |
| 2005 | SBF2 | SET Binding Factor 2 | 2.96 |
| 2006 | S100A4 | S100 Calcium Binding Protein A4 | 2.94 |
| 2007 | GDF1 | Growth Differentiation Factor 1 | 2.94 |
| 2008 | E2F1 | E2F Transcription Factor 1 | 2.94 |
| 2009 | TDP1 | Tyrosyl-DNA Phosphodiesterase 1 | 2.94 |
| 2010 | NEIL1 | Nei Like DNA Glycosylase 1 | 2.94 |
| 2011 | CCIN | Calicin | 2.94 |
| 2012 | AP1B1 | Adaptor Related Protein Complex 1 Subunit Beta 1 | 2.93 |
| 2013 | IL17RA | Interleukin 17 Receptor A | 2.93 |
| 2014 | EZR | Ezrin | 2.91 |
| 2015 | XRCC1 | X-Ray Repair Cross Complementing 1 | 2.91 |
| 2016 | SPAG8 | Sperm Associated Antigen 8 | 2.9 |
| 2017 | ATP2C1 | ATPase Secretory Pathway Ca2+ Transporting 1 | 2.9 |
| 2018 | HSD11B1 | Hydroxysteroid 11-Beta Dehydrogenase 1 | 2.9 |
| 2019 | TWIST2 | Twist Family BHLH Transcription Factor 2 | 2.89 |
| 2020 | DNTT | DNA Nucleotidylexotransferase | 2.89 |
| 2021 | PLXNA1 | Plexin A1 | 2.89 |
| 2022 | SSB | Small RNA Binding Exonuclease Protection Factor La | 2.87 |
| 2023 | LEPR | Leptin Receptor | 2.87 |
| 2024 | FZD8 | Frizzled Class Receptor 8 | 2.86 |
| 2025 | GYPE | Glycophorin E (MNS Blood Group) | 2.86 |
| 2026 | EPB41 | Erythrocyte Membrane Protein Band 4.1 | 2.86 |
| 2027 | IL34 | Interleukin 34 | 2.86 |
| 2028 | PNLIP | Pancreatic Lipase | 2.86 |
| 2029 | BCL6 | BCL6 Transcription Repressor | 2.85 |
| 2030 | STK11 | Serine/Threonine Kinase 11 | 2.85 |
| 2031 | MIR125B1 | MicroRNA 125b-1 | 2.85 |
| 2032 | SLC13A3 | Solute Carrier Family 13 Member 3 | 2.84 |
| 2033 | ADI1 | Acireductone Dioxygenase 1 | 2.84 |
| 2034 | NRIP2 | Nuclear Receptor Interacting Protein 2 | 2.84 |
| 2035 | FGF8 | Fibroblast Growth Factor 8 | 2.84 |
| 2036 | PTH1R | Parathyroid Hormone 1 Receptor | 2.84 |
| 2037 | IGFBP2 | Insulin Like Growth Factor Binding Protein 2 | 2.84 |
| 2038 | CENPF | Centromere Protein F | 2.83 |
| 2039 | SFTPC | Surfactant Protein C | 2.83 |
| 2040 | IRF6 | Interferon Regulatory Factor 6 | 2.83 |
| 2041 | H1-2 | H1.2 Linker Histone, Cluster Member | 2.83 |
| 2042 | CRK | CRK Proto-Oncogene, Adaptor Protein | 2.83 |
| 2043 | CBLIF | Cobalamin Binding Intrinsic Factor | 2.83 |
| 2044 | SPPL2B | Signal Peptide Peptidase Like 2B | 2.83 |
| 2045 | CTSG | Cathepsin G | 2.83 |
| 2046 | UBTF | Upstream Binding Transcription Factor | 2.81 |
| 2047 | ATP1A3 | ATPase Na+/K+ Transporting Subunit Alpha 3 | 2.8 |
| 2048 | MIR27A | MicroRNA 27a | 2.8 |
| 2049 | HSD3B7 | Hydroxy-Delta-5-Steroid Dehydrogenase, 3 Beta- And Steroid Delta-Isomerase 7 | 2.8 |
| 2050 | MNT | MAX Network Transcriptional Repressor | 2.8 |
| 2051 | TPT1 | Tumor Protein, Translationally-Controlled 1 | 2.79 |
| 2052 | MLC1 | Modulator Of VRAC Current 1 | 2.78 |
| 2053 | RYR2 | Ryanodine Receptor 2 | 2.78 |
| 2054 | IGLON5 | IgLON Family Member 5 | 2.78 |
| 2055 | CCR8 | C-C Motif Chemokine Receptor 8 | 2.77 |
| 2056 | EFNB1 | Ephrin B1 | 2.77 |
| 2057 | ADCY5 | Adenylate Cyclase 5 | 2.77 |
| 2058 | HDAC5 | Histone Deacetylase 5 | 2.77 |
| 2059 | CLOCK | Clock Circadian Regulator | 2.76 |
| 2060 | FUCA2 | Alpha-L-Fucosidase 2 | 2.76 |
| 2061 | POU3F3 | POU Class 3 Homeobox 3 | 2.76 |
| 2062 | TMEM126B | Transmembrane Protein 126B | 2.76 |
| 2063 | OPALIN | Oligodendrocytic Myelin Paranodal And Inner Loop Protein | 2.76 |
| 2064 | SLC38A11 | Solute Carrier Family 38 Member 11 | 2.76 |
| 2065 | FUCA1P1 | Alpha-L-Fucosidase 1 Pseudogene 1 | 2.76 |
| 2066 | KLK1 | Kallikrein 1 | 2.76 |
| 2067 | EFEMP1 | EGF Containing Fibulin Extracellular Matrix Protein 1 | 2.76 |
| 2068 | RNF19A | Ring Finger Protein 19A, RBR E3 Ubiquitin Protein Ligase | 2.76 |
| 2069 | DSCAM | DS Cell Adhesion Molecule | 2.75 |
| 2070 | GPX3 | Glutathione Peroxidase 3 | 2.75 |
| 2071 | GAST | Gastrin | 2.75 |
| 2072 | APOL2 | Apolipoprotein L2 | 2.75 |
| 2073 | HSF1 | Heat Shock Transcription Factor 1 | 2.74 |
| 2074 | HSPA9 | Heat Shock Protein Family A (Hsp70) Member 9 | 2.74 |
| 2075 | SLC19A2 | Solute Carrier Family 19 Member 2 | 2.74 |
| 2076 | HGS | Hepatocyte Growth Factor-Regulated Tyrosine Kinase Substrate | 2.74 |
| 2077 | FTO | FTO Alpha-Ketoglutarate Dependent Dioxygenase | 2.73 |
| 2078 | CACNA1S | Calcium Voltage-Gated Channel Subunit Alpha1 S | 2.73 |
| 2079 | TP73 | Tumor Protein P73 | 2.73 |
| 2080 | SLC40A1 | Solute Carrier Family 40 Member 1 | 2.73 |
| 2081 | IL11 | Interleukin 11 | 2.73 |
| 2082 | EFNA1 | Ephrin A1 | 2.73 |
| 2083 | PRKAR1A | Protein Kinase CAMP-Dependent Type I Regulatory Subunit Alpha | 2.73 |
| 2084 | STUB1 | STIP1 Homology And U-Box Containing Protein 1 | 2.72 |
| 2085 | TWIST1 | Twist Family BHLH Transcription Factor 1 | 2.72 |
| 2086 | LINC-PINT | Long Intergenic Non-Protein Coding RNA, P53 Induced Transcript | 2.72 |
| 2087 | SETD1A | SET Domain Containing 1A, Histone Lysine Methyltransferase | 2.72 |
| 2088 | ALDH2 | Aldehyde Dehydrogenase 2 Family Member | 2.72 |
| 2089 | PAH | Phenylalanine Hydroxylase | 2.72 |
| 2090 | WAS | WASP Actin Nucleation Promoting Factor | 2.72 |
| 2091 | GABARAP | GABA Type A Receptor-Associated Protein | 2.72 |
| 2092 | RAB6A | RAB6A, Member RAS Oncogene Family | 2.71 |
| 2093 | PTGER4 | Prostaglandin E Receptor 4 | 2.71 |
| 2094 | CIZ1 | CDKN1A Interacting Zinc Finger Protein 1 | 2.71 |
| 2095 | PIK3CD | Phosphatidylinositol-4,5-Bisphosphate 3-Kinase Catalytic Subunit Delta | 2.71 |
| 2096 | ARG1 | Arginase 1 | 2.71 |
| 2097 | UHRF1 | Ubiquitin Like With PHD And Ring Finger Domains 1 | 2.7 |
| 2098 | THRB | Thyroid Hormone Receptor Beta | 2.7 |
| 2099 | CRHR1 | Corticotropin Releasing Hormone Receptor 1 | 2.7 |
| 2100 | CYP19A1 | Cytochrome P450 Family 19 Subfamily A Member 1 | 2.69 |
| 2101 | PEX2 | Peroxisomal Biogenesis Factor 2 | 2.69 |
| 2102 | PEX5 | Peroxisomal Biogenesis Factor 5 | 2.69 |
| 2103 | GNPTAB | N-Acetylglucosamine-1-Phosphate Transferase Subunits Alpha And Beta | 2.69 |
| 2104 | PVT1 | Pvt1 Oncogene | 2.69 |
| 2105 | KCNC3 | Potassium Voltage-Gated Channel Subfamily C Member 3 | 2.69 |
| 2106 | PDE4D | Phosphodiesterase 4D | 2.69 |
| 2107 | EHD1 | EH Domain Containing 1 | 2.68 |
| 2108 | POSTN | Periostin | 2.68 |
| 2109 | RGS4 | Regulator Of G Protein Signaling 4 | 2.68 |
| 2110 | ACP1 | Acid Phosphatase 1 | 2.68 |
| 2111 | SH2D1A | SH2 Domain Containing 1A | 2.67 |
| 2112 | CEP104 | Centrosomal Protein 104 | 2.67 |
| 2113 | PRKAA2 | Protein Kinase AMP-Activated Catalytic Subunit Alpha 2 | 2.66 |
| 2114 | MSMB | Microseminoprotein Beta | 2.66 |
| 2115 | NDUFS2 | NADH:Ubiquinone Oxidoreductase Core Subunit S2 | 2.65 |
| 2116 | TIAM1 | TIAM Rac1 Associated GEF 1 | 2.64 |
| 2117 | CNKSR2 | Connector Enhancer Of Kinase Suppressor Of Ras 2 | 2.64 |
| 2118 | DYNC1H1 | Dynein Cytoplasmic 1 Heavy Chain 1 | 2.64 |
| 2119 | TKTL1 | Transketolase Like 1 | 2.64 |
| 2120 | PRSS3 | Serine Protease 3 | 2.64 |
| 2121 | FCGR2B | Fc Fragment Of IgG Receptor IIb | 2.63 |
| 2122 | THTPA | Thiamine Triphosphatase | 2.63 |
| 2123 | SPRN | Shadow Of Prion Protein | 2.63 |
| 2124 | CGB3 | Chorionic Gonadotropin Subunit Beta 3 | 2.63 |
| 2125 | CXCR2 | C-X-C Motif Chemokine Receptor 2 | 2.62 |
| 2126 | PDSS2 | Decaprenyl Diphosphate Synthase Subunit 2 | 2.61 |
| 2127 | ZBTB33 | Zinc Finger And BTB Domain Containing 33 | 2.61 |
| 2128 | YBX1 | Y-Box Binding Protein 1 | 2.6 |
| 2129 | CHRFAM7A | CHRNA7 (Exons 5-10) And FAM7A (Exons A-E) Fusion | 2.6 |
| 2130 | GABBR2 | Gamma-Aminobutyric Acid Type B Receptor Subunit 2 | 2.6 |
| 2131 | CCL7 | C-C Motif Chemokine Ligand 7 | 2.6 |
| 2132 | STK24 | Serine/Threonine Kinase 24 | 2.59 |
| 2133 | HNF1B | HNF1 Homeobox B | 2.59 |
| 2134 | ST13 | ST13 Hsp70 Interacting Protein | 2.58 |
| 2135 | AMFR | Autocrine Motility Factor Receptor | 2.58 |
| 2136 | KCNQ2 | Potassium Voltage-Gated Channel Subfamily Q Member 2 | 2.58 |
| 2137 | SLC11A2 | Solute Carrier Family 11 Member 2 | 2.57 |
| 2138 | SENP7 | SUMO Specific Peptidase 7 | 2.57 |
| 2139 | NDUFB8 | NADH:Ubiquinone Oxidoreductase Subunit B8 | 2.57 |
| 2140 | HMGN1 | High Mobility Group Nucleosome Binding Domain 1 | 2.57 |
| 2141 | CD200 | CD200 Molecule | 2.56 |
| 2142 | F11R | F11 Receptor | 2.55 |
| 2143 | NANP | N-Acetylneuraminic Acid Phosphatase | 2.55 |
| 2144 | TFR2 | Transferrin Receptor 2 | 2.55 |
| 2145 | HLA-DMA | Major Histocompatibility Complex, Class II, DM Alpha | 2.55 |
| 2146 | SLAMF1 | Signaling Lymphocytic Activation Molecule Family Member 1 | 2.55 |
| 2147 | CUL1 | Cullin 1 | 2.55 |
| 2148 | CIITA | Class II Major Histocompatibility Complex Transactivator | 2.55 |
| 2149 | PTPN3 | Protein Tyrosine Phosphatase Non-Receptor Type 3 | 2.54 |
| 2150 | FOXH1 | Forkhead Box H1 | 2.54 |
| 2151 | TFB1M | Transcription Factor B1, Mitochondrial | 2.54 |
| 2152 | LRAT | Lecithin Retinol Acyltransferase | 2.54 |
| 2153 | ITM2A | Integral Membrane Protein 2A | 2.53 |
| 2154 | CFP | Complement Factor Properdin | 2.53 |
| 2155 | S100A9 | S100 Calcium Binding Protein A9 | 2.52 |
| 2156 | HMOX2 | Heme Oxygenase 2 | 2.52 |
| 2157 | PPARD | Peroxisome Proliferator Activated Receptor Delta | 2.52 |
| 2158 | PYY | Peptide YY | 2.51 |
| 2159 | CTSH | Cathepsin H | 2.51 |
| 2160 | IDH2 | Isocitrate Dehydrogenase (NADP(+)) 2 | 2.5 |
| 2161 | TCERG1 | Transcription Elongation Regulator 1 | 2.5 |
| 2162 | KDSR | 3-Ketodihydrosphingosine Reductase | 2.5 |
| 2163 | ABCG2 | ATP Binding Cassette Subfamily G Member 2 (Junior Blood Group) | 2.5 |
| 2164 | MIR92A1 | MicroRNA 92a-1 | 2.5 |
| 2165 | HDAC3 | Histone Deacetylase 3 | 2.5 |
| 2166 | HPX | Hemopexin | 2.5 |
| 2167 | OSBPL11 | Oxysterol Binding Protein Like 11 | 2.5 |
| 2168 | LUZP1 | Leucine Zipper Protein 1 | 2.5 |
| 2169 | TALDO1 | Transaldolase 1 | 2.48 |
| 2170 | MAPK11 | Mitogen-Activated Protein Kinase 11 | 2.48 |
| 2171 | GNPDA2 | Glucosamine-6-Phosphate Deaminase 2 | 2.48 |
| 2172 | SOCS1 | Suppressor Of Cytokine Signaling 1 | 2.47 |
| 2173 | HDAC2 | Histone Deacetylase 2 | 2.47 |
| 2174 | HSPB8 | Heat Shock Protein Family B (Small) Member 8 | 2.47 |
| 2175 | GNRH1 | Gonadotropin Releasing Hormone 1 | 2.46 |
| 2176 | CYP17A1 | Cytochrome P450 Family 17 Subfamily A Member 1 | 2.45 |
| 2177 | SFTPD | Surfactant Protein D | 2.45 |
| 2178 | TBL1XR1 | TBL1X Receptor 1 | 2.44 |
| 2179 | PEX14 | Peroxisomal Biogenesis Factor 14 | 2.44 |
| 2180 | ZMPSTE24 | Zinc Metallopeptidase STE24 | 2.44 |
| 2181 | EEA1 | Early Endosome Antigen 1 | 2.44 |
| 2182 | SIRPB1 | Signal Regulatory Protein Beta 1 | 2.43 |
| 2183 | KCNA1 | Potassium Voltage-Gated Channel Subfamily A Member 1 | 2.43 |
| 2184 | HCST | Hematopoietic Cell Signal Transducer | 2.43 |
| 2185 | NGDN | Neuroguidin | 2.43 |
| 2186 | CEBPD | CCAAT Enhancer Binding Protein Delta | 2.43 |
| 2187 | VPS28 | VPS28 Subunit Of ESCRT-I | 2.43 |
| 2188 | FOXO3 | Forkhead Box O3 | 2.42 |
| 2189 | MCF2 | MCF.2 Cell Line Derived Transforming Sequence | 2.42 |
| 2190 | GDF15 | Growth Differentiation Factor 15 | 2.42 |
| 2191 | PEX10 | Peroxisomal Biogenesis Factor 10 | 2.42 |
| 2192 | PEX26 | Peroxisomal Biogenesis Factor 26 | 2.42 |
| 2193 | PEX13 | Peroxisomal Biogenesis Factor 13 | 2.42 |
| 2194 | PEX16 | Peroxisomal Biogenesis Factor 16 | 2.42 |
| 2195 | ADIPOR1 | Adiponectin Receptor 1 | 2.41 |
| 2196 | S100A6 | S100 Calcium Binding Protein A6 | 2.41 |
| 2197 | ST8SIA4 | ST8 Alpha-N-Acetyl-Neuraminide Alpha-2,8-Sialyltransferase 4 | 2.4 |
| 2198 | GLG1 | Golgi Glycoprotein 1 | 2.4 |
| 2199 | LARS1 | Leucyl-TRNA Synthetase 1 | 2.39 |
| 2200 | CDH4 | Cadherin 4 | 2.39 |
| 2201 | CD19 | CD19 Molecule | 2.39 |
| 2202 | ERVW-1 | Endogenous Retrovirus Group W Member 1, Envelope | 2.38 |
| 2203 | DMD | Dystrophin | 2.38 |
| 2204 | SYNJ1 | Synaptojanin 1 | 2.38 |
| 2205 | JAK1 | Janus Kinase 1 | 2.38 |
| 2206 | CTTN | Cortactin | 2.37 |
| 2207 | TRIP4 | Thyroid Hormone Receptor Interactor 4 | 2.37 |
| 2208 | MIR148B | MicroRNA 148b | 2.37 |
| 2209 | FRS2 | Fibroblast Growth Factor Receptor Substrate 2 | 2.36 |
| 2210 | CA1 | Carbonic Anhydrase 1 | 2.36 |
| 2211 | CEP63 | Centrosomal Protein 63 | 2.36 |
| 2212 | PYCR3 | Pyrroline-5-Carboxylate Reductase 3 | 2.36 |
| 2213 | CD200R1 | CD200 Receptor 1 | 2.36 |
| 2214 | SEMA4F | Ssemaphorin 4F | 2.35 |
| 2215 | ME3 | Malic Enzyme 3 | 2.35 |
| 2216 | HCK | HCK Proto-Oncogene, Src Family Tyrosine Kinase | 2.34 |
| 2217 | HNMT | Histamine N-Methyltransferase | 2.34 |
| 2218 | PYCR2 | Pyrroline-5-Carboxylate Reductase 2 | 2.34 |
| 2219 | PGAM1 | Phosphoglycerate Mutase 1 | 2.34 |
| 2220 | COMMD1 | Copper Metabolism Domain Containing 1 | 2.34 |
| 2221 | GAS2 | Growth Arrest Specific 2 | 2.34 |
| 2222 | ANO3 | Anoctamin 3 | 2.34 |
| 2223 | MIEF2 | Mitochondrial Elongation Factor 2 | 2.34 |
| 2224 | UBXN6 | UBX Domain Protein 6 | 2.34 |
| 2225 | PTGIR | Prostaglandin I2 Receptor | 2.34 |
| 2226 | LTBP1 | Latent Transforming Growth Factor Beta Binding Protein 1 | 2.34 |
| 2227 | JAK3 | Janus Kinase 3 | 2.34 |
| 2228 | TBXAS1 | Thromboxane A Synthase 1 | 2.33 |
| 2229 | ATP2A2 | ATPase Sarcoplasmic/Endoplasmic Reticulum Ca2+ Transporting 2 | 2.33 |
| 2230 | WIPF1 | WAS/WASL Interacting Protein Family Member 1 | 2.33 |
| 2231 | HADH | Hydroxyacyl-CoA Dehydrogenase | 2.32 |
| 2232 | NDUFS8 | NADH:Ubiquinone Oxidoreductase Core Subunit S8 | 2.32 |
| 2233 | NDUFV1 | NADH:Ubiquinone Oxidoreductase Core Subunit V1 | 2.32 |
| 2234 | NDUFA1 | NADH:Ubiquinone Oxidoreductase Subunit A1 | 2.32 |
| 2235 | SI | Sucrase-Isomaltase | 2.32 |
| 2236 | SLC25A19 | Solute Carrier Family 25 Member 19 | 2.32 |
| 2237 | DHTKD1 | Dehydrogenase E1 And Transketolase Domain Containing 1 | 2.32 |
| 2238 | DDB1 | Damage Specific DNA Binding Protein 1 | 2.32 |
| 2239 | OTX1 | Orthodenticle Homeobox 1 | 2.32 |
| 2240 | NDUFAF2 | NADH:Ubiquinone Oxidoreductase Complex Assembly Factor 2 | 2.32 |
| 2241 | SLC25A18 | Solute Carrier Family 25 Member 18 | 2.32 |
| 2242 | GNPTG | N-Acetylglucosamine-1-Phosphate Transferase Subunit Gamma | 2.32 |
| 2243 | TTC3 | Tetratricopeptide Repeat Domain 3 | 2.32 |
| 2244 | TMEM126A | Transmembrane Protein 126A | 2.32 |
| 2245 | BRWD1 | Bromodomain And WD Repeat Domain Containing 1 | 2.32 |
| 2246 | MFF | Mitochondrial Fission Factor | 2.32 |
| 2247 | TKTL2 | Transketolase Like 2 | 2.32 |
| 2248 | MDFIC | MyoD Family Inhibitor Domain Containing | 2.32 |
| 2249 | FOXB1 | Forkhead Box B1 | 2.32 |
| 2250 | FCRL3 | Fc Receptor Like 3 | 2.32 |
| 2251 | TEX2 | Testis Expressed 2 | 2.32 |
| 2252 | YRDC | YrdC N6-Threonylcarbamoyltransferase Domain Containing | 2.32 |
| 2253 | TENT5A | Terminal Nucleotidyltransferase 5A | 2.32 |
| 2254 | PLAC4 | Placenta Enriched 4 | 2.32 |
| 2255 | DPP6 | Dipeptidyl Peptidase Like 6 | 2.32 |
| 2256 | RAP2B | RAP2B, Member Of RAS Oncogene Family | 2.31 |
| 2257 | MMP8 | Matrix Metallopeptidase 8 | 2.31 |
| 2258 | EGR3 | Early Growth Response 3 | 2.31 |
| 2259 | TTF2 | Transcription Termination Factor 2 | 2.3 |
| 2260 | SURF4 | Surfeit 4 | 2.3 |
| 2261 | DMAP1 | DNA Methyltransferase 1 Associated Protein 1 | 2.3 |
| 2262 | HMGN4 | High Mobility Group Nucleosomal Binding Domain 4 | 2.3 |
| 2263 | TMEM119 | Transmembrane Protein 119 | 2.3 |
| 2264 | HNRNPA3 | Heterogeneous Nuclear Ribonucleoprotein A3 | 2.3 |
| 2265 | DTNBP1 | Dystrobrevin Binding Protein 1 | 2.3 |
| 2266 | SERPINB5 | Serpin Family B Member 5 | 2.3 |
| 2267 | CHRM3 | Cholinergic Receptor Muscarinic 3 | 2.29 |
| 2268 | CHMP5 | Charged Multivesicular Body Protein 5 | 2.29 |
| 2269 | ORM1 | Orosomucoid 1 | 2.29 |
| 2270 | LBP | Lipopolysaccharide Binding Protein | 2.29 |
| 2271 | FANCD2 | FA Complementation Group D2 | 2.29 |
| 2272 | APOL4 | Apolipoprotein L4 | 2.29 |
| 2273 | GLUD1 | Glutamate Dehydrogenase 1 | 2.28 |
| 2274 | ECE2 | Endothelin Converting Enzyme 2 | 2.28 |
| 2275 | VCAN | Versican | 2.27 |
| 2276 | TCN2 | Transcobalamin 2 | 2.27 |
| 2277 | P4HB | Prolyl 4-Hydroxylase Subunit Beta | 2.27 |
| 2278 | DGCR6 | DiGeorge Syndrome Critical Region Gene 6 | 2.26 |
| 2279 | POLR3A | RNA Polymerase III Subunit A | 2.26 |
| 2280 | DLD | Dihydrolipoamide Dehydrogenase | 2.26 |
| 2281 | SAA4 | Serum Amyloid A4, Constitutive | 2.25 |
| 2282 | MED12 | Mediator Complex Subunit 12 | 2.25 |
| 2283 | PTGER2 | Prostaglandin E Receptor 2 | 2.25 |
| 2284 | PEPD | Peptidase D | 2.25 |
| 2285 | SLC3A2 | Solute Carrier Family 3 Member 2 | 2.25 |
| 2286 | FMNL1 | Formin Like 1 | 2.24 |
| 2287 | P3H3 | Prolyl 3-Hydroxylase 3 | 2.24 |
| 2288 | UBE4A | Ubiquitination Factor E4A | 2.24 |
| 2289 | IGFALS | Insulin Like Growth Factor Binding Protein Acid Labile Subunit | 2.22 |
| 2290 | FKBP1A | FKBP Prolyl Isomerase 1A | 2.22 |
| 2291 | PAWR | Pro-Apoptotic WT1 Regulator | 2.21 |
| 2292 | TPI1 | Triosephosphate Isomerase 1 | 2.21 |
| 2293 | TUBA1A | Tubulin Alpha 1a | 2.21 |
| 2294 | BAK1 | BCL2 Antagonist/Killer 1 | 2.21 |
| 2295 | ALYREF | Aly/REF Export Factor | 2.21 |
| 2296 | ABRA | Actin Binding Rho Activating Protein | 2.2 |
| 2297 | SREBF1 | Sterol Regulatory Element Binding Transcription Factor 1 | 2.2 |
| 2298 | STS | Steroid Sulfatase | 2.2 |
| 2299 | CLEC5A | C-Type Lectin Domain Containing 5A | 2.19 |
| 2300 | NECTIN2 | Nectin Cell Adhesion Molecule 2 | 2.19 |
| 2301 | ZBTB38 | Zinc Finger And BTB Domain Containing 38 | 2.19 |
| 2302 | SACM1L | SAC1 Like Phosphatidylinositide Phosphatase | 2.19 |
| 2303 | ALK | ALK Receptor Tyrosine Kinase | 2.18 |
| 2304 | TPM1 | Tropomyosin 1 | 2.18 |
| 2305 | CCL1 | C-C Motif Chemokine Ligand 1 | 2.18 |
| 2306 | SDC2 | Syndecan 2 | 2.18 |
| 2307 | UTS2R | Urotensin 2 Receptor | 2.18 |
| 2308 | HTR2C | 5-Hydroxytryptamine Receptor 2C | 2.18 |
| 2309 | CFD | Complement Factor D | 2.18 |
| 2310 | ARC | Activity Regulated Cytoskeleton Associated Protein | 2.17 |
| 2311 | PRPF4 | Pre-MRNA Processing Factor 4 | 2.17 |
| 2312 | AIFM1 | Apoptosis Inducing Factor Mitochondria Associated 1 | 2.17 |
| 2313 | KCNN3 | Potassium Calcium-Activated Channel Subfamily N Member 3 | 2.17 |
| 2314 | TRAF6 | TNF Receptor Associated Factor 6 | 2.17 |
| 2315 | POU3F4 | POU Class 3 Homeobox 4 | 2.17 |
| 2316 | CASP10 | Caspase 10 | 2.16 |
| 2317 | CYP7B1 | Cytochrome P450 Family 7 Subfamily B Member 1 | 2.16 |
| 2318 | NOX5 | NADPH Oxidase 5 | 2.16 |
| 2319 | CHMP6 | Charged Multivesicular Body Protein 6 | 2.16 |
| 2320 | MYRF | Myelin Regulatory Factor | 2.16 |
| 2321 | LDHA | Lactate Dehydrogenase A | 2.16 |
| 2322 | TNFSF14 | TNF Superfamily Member 14 | 2.16 |
| 2323 | TREML1 | Triggering Receptor Expressed On Myeloid Cells Like 1 | 2.15 |
| 2324 | CLDN1 | Claudin 1 | 2.15 |
| 2325 | CD58 | CD58 Molecule | 2.15 |
| 2326 | UTRN | Utrophin | 2.15 |
| 2327 | RXRA | Retinoid X Receptor Alpha | 2.15 |
| 2328 | PRKAB1 | Protein Kinase AMP-Activated Non-Catalytic Subunit Beta 1 | 2.14 |
| 2329 | GRIN2D | Glutamate Ionotropic Receptor NMDA Type Subunit 2D | 2.14 |
| 2330 | SYT1 | Synaptotagmin 1 | 2.14 |
| 2331 | MS4A6A | Membrane Spanning 4-Domains A6A | 2.14 |
| 2332 | KRT31 | Keratin 31 | 2.14 |
| 2333 | IL9 | Interleukin 9 | 2.14 |
| 2334 | GRIK2 | Glutamate Ionotropic Receptor Kainate Type Subunit 2 | 2.14 |
| 2335 | HADHB | Hydroxyacyl-CoA Dehydrogenase Trifunctional Multienzyme Complex Subunit Beta | 2.13 |
| 2336 | FHOD1 | Formin Homology 2 Domain Containing 1 | 2.13 |
| 2337 | SIN3A | SIN3 Transcription Regulator Family Member A | 2.13 |
| 2338 | CXCL11 | C-X-C Motif Chemokine Ligand 11 | 2.13 |
| 2339 | IQGAP1 | IQ Motif Containing GTPase Activating Protein 1 | 2.12 |
| 2340 | TACR3 | Tachykinin Receptor 3 | 2.12 |
| 2341 | EPHB2 | EPH Receptor B2 | 2.12 |
| 2342 | BMI1 | BMI1 Proto-Oncogene, Polycomb Ring Finger | 2.12 |
| 2343 | HSP90AB1 | Heat Shock Protein 90 Alpha Family Class B Member 1 | 2.12 |
| 2344 | ELOC | Elongin C | 2.11 |
| 2345 | NCOR1 | Nuclear Receptor Corepressor 1 | 2.11 |
| 2346 | CHRM2 | Cholinergic Receptor Muscarinic 2 | 2.11 |
| 2347 | LSM4 | LSM4 Homolog, U6 Small Nuclear RNA And MRNA Degradation Associated | 2.1 |
| 2348 | PRDX2 | Peroxiredoxin 2 | 2.1 |
| 2349 | KIF11 | Kinesin Family Member 11 | 2.1 |
| 2350 | SNX14 | Sorting Nexin 14 | 2.1 |
| 2351 | CD27 | CD27 Molecule | 2.09 |
| 2352 | HMGA1 | High Mobility Group AT-Hook 1 | 2.09 |
| 2353 | EIF4E | Eukaryotic Translation Initiation Factor 4E | 2.08 |
| 2354 | MIP | Major Intrinsic Protein Of Lens Fiber | 2.08 |
| 2355 | FKBP5 | FKBP Prolyl Isomerase 5 | 2.08 |
| 2356 | RPSA | Ribosomal Protein SA | 2.08 |
| 2357 | MAPKAPK2 | MAPK Activated Protein Kinase 2 | 2.07 |
| 2358 | NID1 | Nidogen 1 | 2.07 |
| 2359 | SERPINB1 | Serpin Family B Member 1 | 2.07 |
| 2360 | DAAM1 | Dishevelled Associated Activator Of Morphogenesis 1 | 2.06 |
| 2361 | ADIPOR2 | Adiponectin Receptor 2 | 2.06 |
| 2362 | HIP1R | Huntingtin Interacting Protein 1 Related | 2.06 |
| 2363 | KLF5 | Kruppel Like Factor 5 | 2.06 |
| 2364 | CHKA | Choline Kinase Alpha | 2.06 |
| 2365 | TGM1 | Transglutaminase 1 | 2.05 |
| 2366 | NFU1 | NFU1 Iron-Sulfur Cluster Scaffold | 2.05 |
| 2367 | CPLX1 | Complexin 1 | 2.05 |
| 2368 | SEC24C | SEC24 Homolog C, COPII Coat Complex Component | 2.04 |
| 2369 | SPTAN1 | Spectrin Alpha, Non-Erythrocytic 1 | 2.04 |
| 2370 | IL4R | Interleukin 4 Receptor | 2.04 |
| 2371 | SUMF1 | Sulfatase Modifying Factor 1 | 2.04 |
| 2372 | BAIAP2L1 | BAR/IMD Domain Containing Adaptor Protein 2 Like 1 | 2.04 |
| 2373 | SCN2A | Sodium Voltage-Gated Channel Alpha Subunit 2 | 2.03 |
| 2374 | CHMP2A | Charged Multivesicular Body Protein 2A | 2.03 |
| 2375 | MAD1L1 | Mitotic Arrest Deficient 1 Like 1 | 2.03 |
| 2376 | ANXA1 | Annexin A1 | 2.03 |
| 2377 | PICK1 | Protein Interacting With PRKCA 1 | 2.03 |
| 2378 | CHERP | Calcium Homeostasis Endoplasmic Reticulum Protein | 2.03 |
| 2379 | LIPA | Lipase A, Lysosomal Acid Type | 2.03 |
| 2380 | TOR2A | Torsin Family 2 Member A | 2.03 |
| 2381 | PFKFB3 | 6-Phosphofructo-2-Kinase/Fructose-2,6-Biphosphatase 3 | 2.02 |
| 2382 | lnc-ZNF296-6 |  | 2.02 |
| 2383 | NONHSAG026010.2 |  | 2.02 |
| 2384 | SRD5A1 | Steroid 5 Alpha-Reductase 1 | 2.02 |
| 2385 | UBE2N | Ubiquitin Conjugating Enzyme E2 N | 2.01 |
| 2386 | SVIP | Small VCP Interacting Protein | 2.01 |
| 2387 | SUOX | Sulfite Oxidase | 2.01 |
| 2388 | MT-TP | Mitochondrially Encoded TRNA-Pro (CCN) | 2.01 |
| 2389 | GPX2 | Glutathione Peroxidase 2 | 2 |
| 2390 | GYS1 | Glycogen Synthase 1 | 1.99 |
| 2391 | VIPR1 | Vasoactive Intestinal Peptide Receptor 1 | 1.99 |
| 2392 | DIAPH1 | Diaphanous Related Formin 1 | 1.99 |
| 2393 | AP5Z1 | Adaptor Related Protein Complex 5 Subunit Zeta 1 | 1.99 |
| 2394 | SRSF1 | Serine And Arginine Rich Splicing Factor 1 | 1.99 |
| 2395 | PLEC | Plectin | 1.99 |
| 2396 | SYNPO | Synaptopodin | 1.99 |
| 2397 | SPTLC3 | Serine Palmitoyltransferase Long Chain Base Subunit 3 | 1.99 |
| 2398 | GCLM | Glutamate-Cysteine Ligase Modifier Subunit | 1.98 |
| 2399 | BAIAP2 | BAR/IMD Domain Containing Adaptor Protein 2 | 1.98 |
| 2400 | SMC1A | Structural Maintenance Of Chromosomes 1A | 1.98 |
| 2401 | MIRLET7E | MicroRNA Let-7e | 1.98 |
| 2402 | MIR335 | MicroRNA 335 | 1.98 |
| 2403 | RHEB | Ras Homolog, MTORC1 Binding | 1.98 |
| 2404 | IRF7 | Interferon Regulatory Factor 7 | 1.98 |
| 2405 | IDH3A | Isocitrate Dehydrogenase (NAD(+)) 3 Catalytic Subunit Alpha | 1.98 |
| 2406 | ANGPTL1 | Angiopoietin Like 1 | 1.98 |
| 2407 | CGB5 | Chorionic Gonadotropin Subunit Beta 5 | 1.98 |
| 2408 | ROS1 | ROS Proto-Oncogene 1, Receptor Tyrosine Kinase | 1.98 |
| 2409 | MSBP2 | Minisatellite Binding Protein 2 | 1.97 |
| 2410 | MTAP | Methylthioadenosine Phosphorylase | 1.97 |
| 2411 | ACER3 | Alkaline Ceramidase 3 | 1.96 |
| 2412 | TRPM3 | Transient Receptor Potential Cation Channel Subfamily M Member 3 | 1.96 |
| 2413 | FAT4 | FAT Atypical Cadherin 4 | 1.96 |
| 2414 | GPC6 | Glypican 6 | 1.95 |
| 2415 | PDLIM3 | PDZ And LIM Domain 3 | 1.95 |
| 2416 | SCG5 | Secretogranin V | 1.95 |
| 2417 | DDX39B | DExD-Box Helicase 39B | 1.95 |
| 2418 | CHD4 | Chromodomain Helicase DNA Binding Protein 4 | 1.95 |
| 2419 | ID3 | Inhibitor Of DNA Binding 3, HLH Protein | 1.94 |
| 2420 | NCS1 | Neuronal Calcium Sensor 1 | 1.94 |
| 2421 | PLB1 | Phospholipase B1 | 1.94 |
| 2422 | CHRM4 | Cholinergic Receptor Muscarinic 4 | 1.94 |
| 2423 | IGHMBP2 | Immunoglobulin Mu DNA Binding Protein 2 | 1.93 |
| 2424 | TCF21 | Transcription Factor 21 | 1.93 |
| 2425 | POLB | DNA Polymerase Beta | 1.93 |
| 2426 | CARD16 | Caspase Recruitment Domain Family Member 16 | 1.92 |
| 2427 | CERS2 | Ceramide Synthase 2 | 1.92 |
| 2428 | MARCKS | Myristoylated Alanine Rich Protein Kinase C Substrate | 1.92 |
| 2429 | MLX | MAX Dimerization Protein MLX | 1.92 |
| 2430 | PRKCQ | Protein Kinase C Theta | 1.91 |
| 2431 | ORAI1 | ORAI Calcium Release-Activated Calcium Modulator 1 | 1.91 |
| 2432 | CXADR | CXADR Ig-Like Cell Adhesion Molecule | 1.91 |
| 2433 | GRIN3A | Glutamate Ionotropic Receptor NMDA Type Subunit 3A | 1.91 |
| 2434 | TMEM230 | Transmembrane Protein 230 | 1.91 |
| 2435 | BSCL2 | BSCL2 Lipid Droplet Biogenesis Associated, Seipin | 1.91 |
| 2436 | MSRA | Methionine Sulfoxide Reductase A | 1.9 |
| 2437 | PRF1 | Perforin 1 | 1.9 |
| 2438 | RFX2 | Regulatory Factor X2 | 1.9 |
| 2439 | HK2 | Hexokinase 2 | 1.9 |
| 2440 | SPPL2A | Signal Peptide Peptidase Like 2A | 1.9 |
| 2441 | TCN1 | Transcobalamin 1 | 1.9 |
| 2442 | MDH2 | Malate Dehydrogenase 2 | 1.9 |
| 2443 | ARRB2 | Arrestin Beta 2 | 1.9 |
| 2444 | FER | FER Tyrosine Kinase | 1.89 |
| 2445 | LTC4S | Leukotriene C4 Synthase | 1.89 |
| 2446 | CTSC | Cathepsin C | 1.89 |
| 2447 | FMNL2 | Formin Like 2 | 1.89 |
| 2448 | CBLL1 | Cbl Proto-Oncogene Like 1 | 1.89 |
| 2449 | SUPT5H | SPT5 Homolog, DSIF Elongation Factor Subunit | 1.88 |
| 2450 | NEDD8 | NEDD8 Ubiquitin Like Modifier | 1.88 |
| 2451 | AGK | Acylglycerol Kinase | 1.88 |
| 2452 | CKAP5 | Cytoskeleton Associated Protein 5 | 1.88 |
| 2453 | PLCG2 | Phospholipase C Gamma 2 | 1.88 |
| 2454 | SERPINB2 | Serpin Family B Member 2 | 1.88 |
| 2455 | PAICS | Phosphoribosylaminoimidazole Carboxylase And Phosphoribosylaminoimidazolesuccinocarboxamide Synthase | 1.88 |
| 2456 | UBE3C | Ubiquitin Protein Ligase E3C | 1.88 |
| 2457 | NCL | Nucleolin | 1.87 |
| 2458 | MBD2 | Methyl-CpG Binding Domain Protein 2 | 1.87 |
| 2459 | SNAI2 | Snail Family Transcriptional Repressor 2 | 1.87 |
| 2460 | PEG3 | Paternally Expressed 3 | 1.87 |
| 2461 | S100A8 | S100 Calcium Binding Protein A8 | 1.87 |
| 2462 | SLC5A7 | Solute Carrier Family 5 Member 7 | 1.87 |
| 2463 | SPINT2 | Serine Peptidase Inhibitor, Kunitz Type 2 | 1.87 |
| 2464 | GC | GC Vitamin D Binding Protein | 1.87 |
| 2465 | CELF1 | CUGBP Elav-Like Family Member 1 | 1.86 |
| 2466 | MIR138-1 | MicroRNA 138-1 | 1.86 |
| 2467 | STEAP3 | STEAP3 Metalloreductase | 1.86 |
| 2468 | NR1I2 | Nuclear Receptor Subfamily 1 Group I Member 2 | 1.86 |
| 2469 | MT-TN | Mitochondrially Encoded TRNA-Asn (AAU/C) | 1.86 |
| 2470 | STX10 | Syntaxin 10 | 1.86 |
| 2471 | ABCG5 | ATP Binding Cassette Subfamily G Member 5 | 1.86 |
| 2472 | KYNU | Kynureninase | 1.86 |
| 2473 | ATP1A1 | ATPase Na+/K+ Transporting Subunit Alpha 1 | 1.86 |
| 2474 | KCNN1 | Potassium Calcium-Activated Channel Subfamily N Member 1 | 1.86 |
| 2475 | FNBP4 | Formin Binding Protein 4 | 1.86 |
| 2476 | B4GALNT2 | Beta-1,4-N-Acetyl-Galactosaminyltransferase 2 | 1.85 |
| 2477 | KAT5 | Lysine Acetyltransferase 5 | 1.85 |
| 2478 | ID2 | Inhibitor Of DNA Binding 2 | 1.85 |
| 2479 | CYFIP2 | Cytoplasmic FMR1 Interacting Protein 2 | 1.85 |
| 2480 | NGB | Neuroglobin | 1.85 |
| 2481 | GPD2 | Glycerol-3-Phosphate Dehydrogenase 2 | 1.85 |
| 2482 | XRCC5 | X-Ray Repair Cross Complementing 5 | 1.85 |
| 2483 | MAP3K14 | Mitogen-Activated Protein Kinase Kinase Kinase 14 | 1.84 |
| 2484 | CCS | Copper Chaperone For Superoxide Dismutase | 1.84 |
| 2485 | TNIK | TRAF2 And NCK Interacting Kinase | 1.84 |
| 2486 | OR4D10 | Olfactory Receptor Family 4 Subfamily D Member 10 | 1.84 |
| 2487 | ACKR3 | Atypical Chemokine Receptor 3 | 1.84 |
| 2488 | TOMM20 | Translocase Of Outer Mitochondrial Membrane 20 | 1.84 |
| 2489 | UBE3B | Ubiquitin Protein Ligase E3B | 1.84 |
| 2490 | CEBPB | CCAAT Enhancer Binding Protein Beta | 1.84 |
| 2491 | SYNCRIP | Synaptotagmin Binding Cytoplasmic RNA Interacting Protein | 1.84 |
| 2492 | ARHGAP35 | Rho GTPase Activating Protein 35 | 1.84 |
| 2493 | NPAS3 | Neuronal PAS Domain Protein 3 | 1.83 |
| 2494 | CNTN4 | Contactin 4 | 1.83 |
| 2495 | DUSP13 | Dual Specificity Phosphatase 13 | 1.83 |
| 2496 | CCNE1 | Cyclin E1 | 1.83 |
| 2497 | GAA | Glucosidase Alpha, Acid | 1.82 |
| 2498 | RBM10 | RNA Binding Motif Protein 10 | 1.82 |
| 2499 | ENPP2 | Ectonucleotide Pyrophosphatase/Phosphodiesterase 2 | 1.82 |
| 2500 | FZD2 | Frizzled Class Receptor 2 | 1.82 |
| 2501 | SLURP1 | Secreted LY6/PLAUR Domain Containing 1 | 1.82 |
| 2502 | RPL35 | Ribosomal Protein L35 | 1.81 |
| 2503 | SERPINI2 | Serpin Family I Member 2 | 1.81 |
| 2504 | TRDMT1 | TRNA Aspartic Acid Methyltransferase 1 | 1.81 |
| 2505 | MICAL1 | Microtubule Associated Monooxygenase, Calponin And LIM Domain Containing 1 | 1.81 |
| 2506 | VPS11 | VPS11 Core Subunit Of CORVET And HOPS Complexes | 1.81 |
| 2507 | RIT2 | Ras Like Without CAAX 2 | 1.81 |
| 2508 | PAX3 | Paired Box 3 | 1.81 |
| 2509 | MIR137 | MicroRNA 137 | 1.81 |
| 2510 | GRIN3B | Glutamate Ionotropic Receptor NMDA Type Subunit 3B | 1.8 |
| 2511 | IL10RB | Interleukin 10 Receptor Subunit Beta | 1.8 |
| 2512 | LINGO1 | Leucine Rich Repeat And Ig Domain Containing 1 | 1.8 |
| 2513 | ARF1 | ADP Ribosylation Factor 1 | 1.8 |
| 2514 | KEAP1 | Kelch Like ECH Associated Protein 1 | 1.8 |
| 2515 | PALM2AKAP2 | PALM2 And AKAP2 Fusion | 1.8 |
| 2516 | GFM1 | G Elongation Factor Mitochondrial 1 | 1.8 |
| 2517 | PURA | Purine Rich Element Binding Protein A | 1.8 |
| 2518 | SLC2A4 | Solute Carrier Family 2 Member 4 | 1.79 |
| 2519 | SCFD1 | Sec1 Family Domain Containing 1 | 1.79 |
| 2520 | HTR7 | 5-Hydroxytryptamine Receptor 7 | 1.79 |
| 2521 | SLC4A1 | Solute Carrier Family 4 Member 1 (Diego Blood Group) | 1.79 |
| 2522 | HNRNPK | Heterogeneous Nuclear Ribonucleoprotein K | 1.79 |
| 2523 | MAP2K6 | Mitogen-Activated Protein Kinase Kinase 6 | 1.79 |
| 2524 | ALDH1A1 | Aldehyde Dehydrogenase 1 Family Member A1 | 1.79 |
| 2525 | TAAR6 | Trace Amine Associated Receptor 6 | 1.79 |
| 2526 | RANGAP1 | Ran GTPase Activating Protein 1 | 1.79 |
| 2527 | ALDH16A1 | Aldehyde Dehydrogenase 16 Family Member A1 | 1.79 |
| 2528 | ASS1 | Argininosuccinate Synthase 1 | 1.79 |
| 2529 | LMAN1 | Lectin, Mannose Binding 1 | 1.78 |
| 2530 | IGFBP5 | Insulin Like Growth Factor Binding Protein 5 | 1.78 |
| 2531 | NAIP | NLR Family Apoptosis Inhibitory Protein | 1.78 |
| 2532 | XRCC6 | X-Ray Repair Cross Complementing 6 | 1.77 |
| 2533 | CSRP1 | Cysteine And Glycine Rich Protein 1 | 1.77 |
| 2534 | SET | SET Nuclear Proto-Oncogene | 1.77 |
| 2535 | MED31 | Mediator Complex Subunit 31 | 1.77 |
| 2536 | SPTSSA | Serine Palmitoyltransferase Small Subunit A | 1.77 |
| 2537 | SERTAD3 | SERTA Domain Containing 3 | 1.77 |
| 2538 | SHB | SH2 Domain Containing Adaptor Protein B | 1.77 |
| 2539 | DEFB103B | Defensin Beta 103B | 1.77 |
| 2540 | HTR4 | 5-Hydroxytryptamine Receptor 4 | 1.77 |
| 2541 | GPNMB | Glycoprotein Nmb | 1.76 |
| 2542 | STX6 | Syntaxin 6 | 1.76 |
| 2543 | MFGE8 | Milk Fat Globule EGF And Factor V/VIII Domain Containing | 1.76 |
| 2544 | GNAI1 | G Protein Subunit Alpha I1 | 1.76 |
| 2545 | MCCC1 | Methylcrotonoyl-CoA Carboxylase 1 | 1.76 |
| 2546 | HJV | Hemojuvelin BMP Co-Receptor | 1.76 |
| 2547 | PTGES | Prostaglandin E Synthase | 1.75 |
| 2548 | AHSA1 | Activator Of HSP90 ATPase Activity 1 | 1.75 |
| 2549 | PIKFYVE | Phosphoinositide Kinase, FYVE-Type Zinc Finger Containing | 1.75 |
| 2550 | SYNJ2 | Synaptojanin 2 | 1.75 |
| 2551 | NUP85 | Nucleoporin 85 | 1.75 |
| 2552 | GZMM | Granzyme M | 1.75 |
| 2553 | SERAC1 | Serine Active Site Containing 1 | 1.75 |
| 2554 | CEBPA | CCAAT Enhancer Binding Protein Alpha | 1.75 |
| 2555 | TSC22D3 | TSC22 Domain Family Member 3 | 1.75 |
| 2556 | DDIT4 | DNA Damage Inducible Transcript 4 | 1.75 |
| 2557 | H4C1 | H4 Clustered Histone 1 | 1.75 |
| 2558 | ELP4 | Elongator Acetyltransferase Complex Subunit 4 | 1.75 |
| 2559 | CCKBR | Cholecystokinin B Receptor | 1.75 |
| 2560 | NLRP1 | NLR Family Pyrin Domain Containing 1 | 1.74 |
| 2561 | ERLIN2 | ER Lipid Raft Associated 2 | 1.74 |
| 2562 | RPLP1 | Ribosomal Protein Lateral Stalk Subunit P1 | 1.74 |
| 2563 | NRXN2 | Neurexin 2 | 1.74 |
| 2564 | CTSZ | Cathepsin Z | 1.74 |
| 2565 | SYN3 | Synapsin III | 1.73 |
| 2566 | HPS1 | HPS1 Biogenesis Of Lysosomal Organelles Complex 3 Subunit 1 | 1.72 |
| 2567 | NRXN3 | Neurexin 3 | 1.72 |
| 2568 | REEP1 | Receptor Accessory Protein 1 | 1.72 |
| 2569 | FMNL3 | Formin Like 3 | 1.72 |
| 2570 | STX16 | Syntaxin 16 | 1.72 |
| 2571 | P4HTM | Prolyl 4-Hydroxylase, Transmembrane | 1.71 |
| 2572 | SERPINA7 | Serpin Family A Member 7 | 1.71 |
| 2573 | FES | FES Proto-Oncogene, Tyrosine Kinase | 1.71 |
| 2574 | HAO1 | Hydroxyacid Oxidase 1 | 1.71 |
| 2575 | CSF3R | Colony Stimulating Factor 3 Receptor | 1.71 |
| 2576 | RSU1 | Ras Suppressor Protein 1 | 1.7 |
| 2577 | CCRL2 | C-C Motif Chemokine Receptor Like 2 | 1.7 |
| 2578 | CDR2 | Cerebellar Degeneration Related Protein 2 | 1.7 |
| 2579 | SALL4 | Spalt Like Transcription Factor 4 | 1.7 |
| 2580 | SCAP | SREBF Chaperone | 1.7 |
| 2581 | ATP13A3 | ATPase 13A3 | 1.7 |
| 2582 | PNMA2 | PNMA Family Member 2 | 1.7 |
| 2583 | AGPAT2 | 1-Acylglycerol-3-Phosphate O-Acyltransferase 2 | 1.7 |
| 2584 | TBC1D24 | TBC1 Domain Family Member 24 | 1.7 |
| 2585 | CTSK | Cathepsin K | 1.7 |
| 2586 | KARS1 | Lysyl-TRNA Synthetase 1 | 1.7 |
| 2587 | CYP7A1 | Cytochrome P450 Family 7 Subfamily A Member 1 | 1.7 |
| 2588 | EPHA6 | EPH Receptor A6 | 1.69 |
| 2589 | GPR37 | G Protein-Coupled Receptor 37 | 1.69 |
| 2590 | NLGN4X | Neuroligin 4 X-Linked | 1.69 |
| 2591 | STC2 | Stanniocalcin 2 | 1.69 |
| 2592 | RPS6KA3 | Ribosomal Protein S6 Kinase A3 | 1.69 |
| 2593 | SEPTIN7 | Septin 7 | 1.69 |
| 2594 | PRKAA1 | Protein Kinase AMP-Activated Catalytic Subunit Alpha 1 | 1.68 |
| 2595 | PDE4B | Phosphodiesterase 4B | 1.68 |
| 2596 | MCFD2 | Multiple Coagulation Factor Deficiency 2, ER Cargo Receptor Complex Subunit | 1.68 |
| 2597 | DAPK1 | Death Associated Protein Kinase 1 | 1.68 |
| 2598 | PIK3R5 | Phosphoinositide-3-Kinase Regulatory Subunit 5 | 1.68 |
| 2599 | AUH | AU RNA Binding Methylglutaconyl-CoA Hydratase | 1.68 |
| 2600 | SLC6A20 | Solute Carrier Family 6 Member 20 | 1.68 |
| 2601 | PCBP4 | Poly(RC) Binding Protein 4 | 1.68 |
| 2602 | TADA2B | Transcriptional Adaptor 2B | 1.68 |
| 2603 | RBM45 | RNA Binding Motif Protein 45 | 1.68 |
| 2604 | SCAF4 | SR-Related CTD Associated Factor 4 | 1.68 |
| 2605 | RNASE13 | Ribonuclease A Family Member 13 (Inactive) | 1.68 |
| 2606 | FIP1L1 | Factor Interacting With PAPOLA And CPSF1 | 1.68 |
| 2607 | ZNF569 | Zinc Finger Protein 569 | 1.67 |
| 2608 | ATXN2L | Ataxin 2 Like | 1.67 |
| 2609 | PHB | Prohibitin | 1.67 |
| 2610 | FBLN1 | Fibulin 1 | 1.67 |
| 2611 | XRCC2 | X-Ray Repair Cross Complementing 2 | 1.67 |
| 2612 | KDM1A | Lysine Demethylase 1A | 1.67 |
| 2613 | CTRL | Chymotrypsin Like | 1.67 |
| 2614 | GRM8 | Glutamate Metabotropic Receptor 8 | 1.66 |
| 2615 | FANCM | FA Complementation Group M | 1.66 |
| 2616 | DEK | DEK Proto-Oncogene | 1.66 |
| 2617 | TBX18 | T-Box Transcription Factor 18 | 1.66 |
| 2618 | WIPI1 | WD Repeat Domain, Phosphoinositide Interacting 1 | 1.66 |
| 2619 | ZBTB24 | Zinc Finger And BTB Domain Containing 24 | 1.66 |
| 2620 | DLGAP1 | DLG Associated Protein 1 | 1.66 |
| 2621 | ENDOG | Endonuclease G | 1.65 |
| 2622 | PKM | Pyruvate Kinase M1/2 | 1.65 |
| 2623 | PLCB3 | Phospholipase C Beta 3 | 1.65 |
| 2624 | PHGDH | Phosphoglycerate Dehydrogenase | 1.65 |
| 2625 | NFKBIB | NFKB Inhibitor Beta | 1.65 |
| 2626 | SEPTIN4 | Septin 4 | 1.65 |
| 2627 | PHKG2 | Phosphorylase Kinase Catalytic Subunit Gamma 2 | 1.64 |
| 2628 | PRKACG | Protein Kinase CAMP-Activated Catalytic Subunit Gamma | 1.64 |
| 2629 | UBA1 | Ubiquitin Like Modifier Activating Enzyme 1 | 1.64 |
| 2630 | SKAP2 | Src Kinase Associated Phosphoprotein 2 | 1.64 |
| 2631 | TRAP1 | TNF Receptor Associated Protein 1 | 1.64 |
| 2632 | FSHR | Follicle Stimulating Hormone Receptor | 1.64 |
| 2633 | DIO2 | Iodothyronine Deiodinase 2 | 1.64 |
| 2634 | UBA52 | Ubiquitin A-52 Residue Ribosomal Protein Fusion Product 1 | 1.64 |
| 2635 | EWSR1 | EWS RNA Binding Protein 1 | 1.63 |
| 2636 | CLTC | Clathrin Heavy Chain | 1.63 |
| 2637 | ADH1A | Alcohol Dehydrogenase 1A (Class I), Alpha Polypeptide | 1.63 |
| 2638 | COPE | COPI Coat Complex Subunit Epsilon | 1.63 |
| 2639 | CA10 | Carbonic Anhydrase 10 | 1.63 |
| 2640 | CTNND2 | Catenin Delta 2 | 1.63 |
| 2641 | MAPRE3 | Microtubule Associated Protein RP/EB Family Member 3 | 1.63 |
| 2642 | GAK | Cyclin G Associated Kinase | 1.62 |
| 2643 | ID4 | Inhibitor Of DNA Binding 4, HLH Protein | 1.62 |
| 2644 | SRPX2 | Sushi Repeat Containing Protein X-Linked 2 | 1.62 |
| 2645 | CPT1C | Carnitine Palmitoyltransferase 1C | 1.62 |
| 2646 | SLX4 | SLX4 Structure-Specific Endonuclease Subunit | 1.62 |
| 2647 | PPCS | Phosphopantothenoylcysteine Synthetase | 1.62 |
| 2648 | ST3GAL4 | ST3 Beta-Galactoside Alpha-2,3-Sialyltransferase 4 | 1.61 |
| 2649 | PFKM | Phosphofructokinase, Muscle | 1.61 |
| 2650 | SLC39A8 | Solute Carrier Family 39 Member 8 | 1.61 |
| 2651 | SPRY4-AS1 | SPRY4 Antisense RNA 1 | 1.61 |
| 2652 | POT1 | Protection Of Telomeres 1 | 1.61 |
| 2653 | IGFBP4 | Insulin Like Growth Factor Binding Protein 4 | 1.61 |
| 2654 | CXCR6 | C-X-C Motif Chemokine Receptor 6 | 1.61 |
| 2655 | GSTO2 | Glutathione S-Transferase Omega 2 | 1.61 |
| 2656 | LRRC37A3 | Leucine Rich Repeat Containing 37 Member A3 | 1.61 |
| 2657 | QKI | QKI, KH Domain Containing RNA Binding | 1.61 |
| 2658 | UHRF2 | Ubiquitin Like With PHD And Ring Finger Domains 2 | 1.61 |
| 2659 | NBEAL2 | Neurobeachin Like 2 | 1.6 |
| 2660 | GPR182 | G Protein-Coupled Receptor 182 | 1.6 |
| 2661 | CHL1 | Cell Adhesion Molecule L1 Like | 1.6 |
| 2662 | HIPK3 | Homeodomain Interacting Protein Kinase 3 | 1.6 |
| 2663 | RECQL4 | RecQ Like Helicase 4 | 1.6 |
| 2664 | SIAH1 | Siah E3 Ubiquitin Protein Ligase 1 | 1.6 |
| 2665 | SF3B6 | Splicing Factor 3b Subunit 6 | 1.6 |
| 2666 | RMDN3 | Regulator Of Microtubule Dynamics 3 | 1.6 |
| 2667 | CDH11 | Cadherin 11 | 1.59 |
| 2668 | AQP5 | Aquaporin 5 | 1.59 |
| 2669 | CD151 | CD151 Molecule (Raph Blood Group) | 1.59 |
| 2670 | SRR | Serine Racemase | 1.59 |
| 2671 | SCD | Stearoyl-CoA Desaturase | 1.59 |
| 2672 | SYVN1 | Synoviolin 1 | 1.59 |
| 2673 | TPH2 | Tryptophan Hydroxylase 2 | 1.59 |
| 2674 | SDCBP | Syndecan Binding Protein | 1.59 |
| 2675 | HTR2B | 5-Hydroxytryptamine Receptor 2B | 1.59 |
| 2676 | PDE1C | Phosphodiesterase 1C | 1.59 |
| 2677 | MAP4K4 | Mitogen-Activated Protein Kinase Kinase Kinase Kinase 4 | 1.59 |
| 2678 | TMOD2 | Tropomodulin 2 | 1.58 |
| 2679 | GLIS1 | GLIS Family Zinc Finger 1 | 1.58 |
| 2680 | FAF1 | Fas Associated Factor 1 | 1.58 |
| 2681 | WRN | WRN RecQ Like Helicase | 1.58 |
| 2682 | H1-4 | H1.4 Linker Histone, Cluster Member | 1.58 |
| 2683 | NR1H3 | Nuclear Receptor Subfamily 1 Group H Member 3 | 1.58 |
| 2684 | DGKQ | Diacylglycerol Kinase Theta | 1.58 |
| 2685 | RAB10 | RAB10, Member RAS Oncogene Family | 1.58 |
| 2686 | NNAT | Neuronatin | 1.58 |
| 2687 | PRSS1 | Serine Protease 1 | 1.57 |
| 2688 | RPS13 | Ribosomal Protein S13 | 1.57 |
| 2689 | ARTN | Artemin | 1.57 |
| 2690 | CNP | 2',3'-Cyclic Nucleotide 3' Phosphodiesterase | 1.57 |
| 2691 | NFATC2 | Nuclear Factor Of Activated T Cells 2 | 1.57 |
| 2692 | MIR483 | MicroRNA 483 | 1.57 |
| 2693 | FCGR3A | Fc Fragment Of IgG Receptor IIIa | 1.57 |
| 2694 | SLC25A11 | Solute Carrier Family 25 Member 11 | 1.56 |
| 2695 | LOC111365141 | NOS2 5' Regulatory Region | 1.56 |
| 2696 | IMMT | Inner Membrane Mitochondrial Protein | 1.56 |
| 2697 | ALDOA | Aldolase, Fructose-Bisphosphate A | 1.56 |
| 2698 | TRAF1 | TNF Receptor Associated Factor 1 | 1.56 |
| 2699 | MFSD2A | Major Facilitator Superfamily Domain Containing 2A | 1.56 |
| 2700 | BST1 | Bone Marrow Stromal Cell Antigen 1 | 1.55 |
| 2701 | PSPN | Persephin | 1.55 |
| 2702 | HACD1 | 3-Hydroxyacyl-CoA Dehydratase 1 | 1.55 |
| 2703 | FBXL5 | F-Box And Leucine Rich Repeat Protein 5 | 1.55 |
| 2704 | CNOT8 | CCR4-NOT Transcription Complex Subunit 8 | 1.55 |
| 2705 | POR | Cytochrome P450 Oxidoreductase | 1.55 |
| 2706 | CNTFR | Ciliary Neurotrophic Factor Receptor | 1.55 |
| 2707 | SPTBN1 | Spectrin Beta, Non-Erythrocytic 1 | 1.55 |
| 2708 | SAFB | Scaffold Attachment Factor B | 1.55 |
| 2709 | CSMD1 | CUB And Sushi Multiple Domains 1 | 1.55 |
| 2710 | SQLE | Squalene Epoxidase | 1.55 |
| 2711 | QDPR | Quinoid Dihydropteridine Reductase | 1.55 |
| 2712 | CDC25C | Cell Division Cycle 25C | 1.54 |
| 2713 | FAM20C | FAM20C Golgi Associated Secretory Pathway Kinase | 1.54 |
| 2714 | PPP1R9B | Protein Phosphatase 1 Regulatory Subunit 9B | 1.54 |
| 2715 | PHEX | Phosphate Regulating Endopeptidase Homolog X-Linked | 1.54 |
| 2716 | HLA-DRB4 | Major Histocompatibility Complex, Class II, DR Beta 4 | 1.54 |
| 2717 | HOXD13 | Homeobox D13 | 1.54 |
| 2718 | ANXA7 | Annexin A7 | 1.54 |
| 2719 | NR2F1 | Nuclear Receptor Subfamily 2 Group F Member 1 | 1.54 |
| 2720 | RPS6 | Ribosomal Protein S6 | 1.54 |
| 2721 | H4C6 | H4 Clustered Histone 6 | 1.53 |
| 2722 | CSN1S1 | Casein Alpha S1 | 1.53 |
| 2723 | PCBP1 | Poly(RC) Binding Protein 1 | 1.53 |
| 2724 | GTPBP2 | GTP Binding Protein 2 | 1.53 |
| 2725 | SUCLG1 | Succinate-CoA Ligase GDP/ADP-Forming Subunit Alpha | 1.53 |
| 2726 | H6PD | Hexose-6-Phosphate Dehydrogenase/Glucose 1-Dehydrogenase | 1.53 |
| 2727 | DYSF | Dysferlin | 1.53 |
| 2728 | LIPN | Lipase Family Member N | 1.53 |
| 2729 | SPRY4 | Sprouty RTK Signaling Antagonist 4 | 1.53 |
| 2730 | CCDC88A | Coiled-Coil Domain Containing 88A | 1.52 |
| 2731 | RNF4 | Ring Finger Protein 4 | 1.52 |
| 2732 | MECOM | MDS1 And EVI1 Complex Locus | 1.52 |
| 2733 | DNAJC21 | DnaJ Heat Shock Protein Family (Hsp40) Member C21 | 1.52 |
| 2734 | RPS19BP1 | Ribosomal Protein S19 Binding Protein 1 | 1.52 |
| 2735 | PGA4 | Pepsinogen A4 | 1.52 |
| 2736 | MT-TY | Mitochondrially Encoded TRNA-Tyr (UAU/C) | 1.52 |
| 2737 | AAK1 | AP2 Associated Kinase 1 | 1.52 |
| 2738 | SYT11 | Synaptotagmin 11 | 1.52 |
| 2739 | MAP3K11 | Mitogen-Activated Protein Kinase Kinase Kinase 11 | 1.52 |
| 2740 | PHLDB2 | Pleckstrin Homology Like Domain Family B Member 2 | 1.52 |
| 2741 | CYGB | Cytoglobin | 1.52 |
| 2742 | LARS2 | Leucyl-TRNA Synthetase 2, Mitochondrial | 1.51 |
| 2743 | CELF4 | CUGBP Elav-Like Family Member 4 | 1.51 |
| 2744 | SETD5 | SET Domain Containing 5 | 1.51 |
| 2745 | KRBOX4 | KRAB Box Domain Containing 4 | 1.51 |
| 2746 | COL6A2 | Collagen Type VI Alpha 2 Chain | 1.51 |
| 2747 | ALOX15 | Arachidonate 15-Lipoxygenase | 1.5 |
| 2748 | MARS1 | Methionyl-TRNA Synthetase 1 | 1.5 |
| 2749 | NUDT1 | Nudix Hydrolase 1 | 1.5 |
| 2750 | AIMP2 | Aminoacyl TRNA Synthetase Complex Interacting Multifunctional Protein 2 | 1.5 |
| 2751 | SYT12 | Synaptotagmin 12 | 1.5 |
| 2752 | SNHG1 | Small Nucleolar RNA Host Gene 1 | 1.5 |
| 2753 | SMPD2 | Sphingomyelin Phosphodiesterase 2 | 1.5 |
| 2754 | WIPI2 | WD Repeat Domain, Phosphoinositide Interacting 2 | 1.5 |
| 2755 | MBNL1 | Muscleblind Like Splicing Regulator 1 | 1.5 |
| 2756 | SSBP1 | Single Stranded DNA Binding Protein 1 | 1.49 |
| 2757 | GRK5 | G Protein-Coupled Receptor Kinase 5 | 1.49 |
| 2758 | MCTP2 | Multiple C2 And Transmembrane Domain Containing 2 | 1.49 |
| 2759 | SP3 | Sp3 Transcription Factor | 1.49 |
| 2760 | RTN4IP1 | Reticulon 4 Interacting Protein 1 | 1.49 |
| 2761 | PPP1CB | Protein Phosphatase 1 Catalytic Subunit Beta | 1.49 |
| 2762 | NLGN1 | Neuroligin 1 | 1.48 |
| 2763 | GRIK5 | Glutamate Ionotropic Receptor Kainate Type Subunit 5 | 1.48 |
| 2764 | FAN1 | FANCD2 And FANCI Associated Nuclease 1 | 1.48 |
| 2765 | TYRP1 | Tyrosinase Related Protein 1 | 1.48 |
| 2766 | ARIH2 | Ariadne RBR E3 Ubiquitin Protein Ligase 2 | 1.48 |
| 2767 | AMBRA1 | Autophagy And Beclin 1 Regulator 1 | 1.48 |
| 2768 | SNX27 | Sorting Nexin 27 | 1.48 |
| 2769 | RANBP17 | RAN Binding Protein 17 | 1.48 |
| 2770 | TIMM10 | Translocase Of Inner Mitochondrial Membrane 10 | 1.48 |
| 2771 | TIMM22 | Translocase Of Inner Mitochondrial Membrane 22 | 1.48 |
| 2772 | ENTR1 | Endosome Associated Trafficking Regulator 1 | 1.48 |
| 2773 | TIMM29 | Translocase Of Inner Mitochondrial Membrane 29 | 1.48 |
| 2774 | DPY19L2P2 | DPY19L2 Pseudogene 2 | 1.48 |
| 2775 | UCHL1-AS1 | UCHL1 Antisense RNA 1 (Head To Head) | 1.48 |
| 2776 | ARF6 | ADP Ribosylation Factor 6 | 1.48 |
| 2777 | KLHDC4 | Kelch Domain Containing 4 | 1.48 |
| 2778 | MYH10 | Myosin Heavy Chain 10 | 1.48 |
| 2779 | MAP2K3 | Mitogen-Activated Protein Kinase Kinase 3 | 1.47 |
| 2780 | CDC5L | Cell Division Cycle 5 Like | 1.47 |
| 2781 | GHR | Growth Hormone Receptor | 1.47 |
| 2782 | NPY1R | Neuropeptide Y Receptor Y1 | 1.47 |
| 2783 | ALAS2 | 5'-Aminolevulinate Synthase 2 | 1.46 |
| 2784 | BEST2 | Bestrophin 2 | 1.46 |
| 2785 | BRD1 | Bromodomain Containing 1 | 1.46 |
| 2786 | PSMA7 | Proteasome 20S Subunit Alpha 7 | 1.46 |
| 2787 | MIR200A | MicroRNA 200a | 1.46 |
| 2788 | LINC00841 | Long Intergenic Non-Protein Coding RNA 841 | 1.45 |
| 2789 | MNDA | Myeloid Cell Nuclear Differentiation Antigen | 1.45 |
| 2790 | AMOT | Angiomotin | 1.45 |
| 2791 | CD70 | CD70 Molecule | 1.45 |
| 2792 | CANX | Calnexin | 1.44 |
| 2793 | JPH4 | Junctophilin 4 | 1.44 |
| 2794 | MIR497 | MicroRNA 497 | 1.44 |
| 2795 | PLXNA2 | Plexin A2 | 1.44 |
| 2796 | C5AR2 | Complement Component 5a Receptor 2 | 1.44 |
| 2797 | DGKH | Diacylglycerol Kinase Eta | 1.44 |
| 2798 | ASTN2 | Astrotactin 2 | 1.44 |
| 2799 | GOT2 | Glutamic-Oxaloacetic Transaminase 2 | 1.44 |
| 2800 | CYP20A1 | Cytochrome P450 Family 20 Subfamily A Member 1 | 1.44 |
| 2801 | LRP1B | LDL Receptor Related Protein 1B | 1.43 |
| 2802 | LNX1 | Ligand Of Numb-Protein X 1 | 1.43 |
| 2803 | GRM4 | Glutamate Metabotropic Receptor 4 | 1.43 |
| 2804 | MIR486-1 | MicroRNA 486-1 | 1.43 |
| 2805 | PLA2G2D | Phospholipase A2 Group IID | 1.43 |
| 2806 | PNOC | Prepronociceptin | 1.43 |
| 2807 | PGAM5 | PGAM Family Member 5, Mitochondrial Serine/Threonine Protein Phosphatase | 1.43 |
| 2808 | FBXO48 | F-Box Protein 48 | 1.43 |
| 2809 | MANBA | Mannosidase Beta | 1.42 |
| 2810 | EVPL | Envoplakin | 1.42 |
| 2811 | H2BC21 | H2B Clustered Histone 21 | 1.42 |
| 2812 | PER1 | Period Circadian Regulator 1 | 1.42 |
| 2813 | WWOX | WW Domain Containing Oxidoreductase | 1.42 |
| 2814 | EEF2 | Eukaryotic Translation Elongation Factor 2 | 1.42 |
| 2815 | NBPF3 | NBPF Member 3 | 1.42 |
| 2816 | YJU2 | YJU2 Splicing Factor Homolog | 1.42 |
| 2817 | RORA | RAR Related Orphan Receptor A | 1.41 |
| 2818 | PSMD9 | Proteasome 26S Subunit, Non-ATPase 9 | 1.41 |
| 2819 | NEU3 | Neuraminidase 3 | 1.41 |
| 2820 | FAAH | Fatty Acid Amide Hydrolase | 1.41 |
| 2821 | CYFIP1 | Cytoplasmic FMR1 Interacting Protein 1 | 1.41 |
| 2822 | TBR1 | T-Box Brain Transcription Factor 1 | 1.41 |
| 2823 | ABCB7 | ATP Binding Cassette Subfamily B Member 7 | 1.41 |
| 2824 | CPQ | Carboxypeptidase Q | 1.4 |
| 2825 | SPR | Sepiapterin Reductase | 1.4 |
| 2826 | SH3GL2 | SH3 Domain Containing GRB2 Like 2, Endophilin A1 | 1.4 |
| 2827 | CPE | Carboxypeptidase E | 1.4 |
| 2828 | FECH | Ferrochelatase | 1.4 |
| 2829 | LRG1 | Leucine Rich Alpha-2-Glycoprotein 1 | 1.4 |
| 2830 | EXTL3 | Exostosin Like Glycosyltransferase 3 | 1.4 |
| 2831 | FANCB | FA Complementation Group B | 1.4 |
| 2832 | FARSB | Phenylalanyl-TRNA Synthetase Subunit Beta | 1.4 |
| 2833 | SLC1A4 | Solute Carrier Family 1 Member 4 | 1.39 |
| 2834 | LMOD1 | Leiomodin 1 | 1.39 |
| 2835 | PTGER3 | Prostaglandin E Receptor 3 | 1.38 |
| 2836 | SIPA1L2 | Signal Induced Proliferation Associated 1 Like 2 | 1.38 |
| 2837 | SORCS1 | Sortilin Related VPS10 Domain Containing Receptor 1 | 1.38 |
| 2838 | MCM4 | Minichromosome Maintenance Complex Component 4 | 1.38 |
| 2839 | IMPA1 | Inositol Monophosphatase 1 | 1.38 |
| 2840 | GNAI3 | G Protein Subunit Alpha I3 | 1.38 |
| 2841 | PDE3B | Phosphodiesterase 3B | 1.38 |
| 2842 | MEIS1 | Meis Homeobox 1 | 1.38 |
| 2843 | SP4 | Sp4 Transcription Factor | 1.38 |
| 2844 | SNAI1 | Snail Family Transcriptional Repressor 1 | 1.37 |
| 2845 | BLZF1 | Basic Leucine Zipper Nuclear Factor 1 | 1.37 |
| 2846 | SNX3 | Sorting Nexin 3 | 1.37 |
| 2847 | SMPX | Small Muscle Protein X-Linked | 1.37 |
| 2848 | NARS2 | Asparaginyl-TRNA Synthetase 2, Mitochondrial | 1.37 |
| 2849 | RTN2 | Reticulon 2 | 1.37 |
| 2850 | ZEB1 | Zinc Finger E-Box Binding Homeobox 1 | 1.37 |
| 2851 | MUS81 | MUS81 Structure-Specific Endonuclease Subunit | 1.37 |
| 2852 | ROBO1 | Roundabout Guidance Receptor 1 | 1.37 |
| 2853 | LIG1 | DNA Ligase 1 | 1.36 |
| 2854 | AGL | Amylo-Alpha-1, 6-Glucosidase, 4-Alpha-Glucanotransferase | 1.36 |
| 2855 | ALDH4A1 | Aldehyde Dehydrogenase 4 Family Member A1 | 1.36 |
| 2856 | MPG | N-Methylpurine DNA Glycosylase | 1.36 |
| 2857 | PPP1R3C | Protein Phosphatase 1 Regulatory Subunit 3C | 1.36 |
| 2858 | WDR45B | WD Repeat Domain 45B | 1.36 |
| 2859 | FAM117B | Family With Sequence Similarity 117 Member B | 1.36 |
| 2860 | FCSK | Fucose Kinase | 1.36 |
| 2861 | PPP1R27 | Protein Phosphatase 1 Regulatory Subunit 27 | 1.36 |
| 2862 | DIPK1A | Divergent Protein Kinase Domain 1A | 1.36 |
| 2863 | CYP26A1 | Cytochrome P450 Family 26 Subfamily A Member 1 | 1.36 |
| 2864 | HMBS | Hydroxymethylbilane Synthase | 1.36 |
| 2865 | DLX5 | Distal-Less Homeobox 5 | 1.36 |
| 2866 | PEX11B | Peroxisomal Biogenesis Factor 11 Beta | 1.36 |
| 2867 | DLX6 | Distal-Less Homeobox 6 | 1.36 |
| 2868 | LRP10 | LDL Receptor Related Protein 10 | 1.35 |
| 2869 | NOX3 | NADPH Oxidase 3 | 1.35 |
| 2870 | NUP133 | Nucleoporin 133 | 1.35 |
| 2871 | DDX6 | DEAD-Box Helicase 6 | 1.35 |
| 2872 | PSAT1 | Phosphoserine Aminotransferase 1 | 1.35 |
| 2873 | CRY2 | Cryptochrome Circadian Regulator 2 | 1.35 |
| 2874 | REM1 | RRAD And GEM Like GTPase 1 | 1.35 |
| 2875 | PAK2 | P21 (RAC1) Activated Kinase 2 | 1.35 |
| 2876 | KHDRBS1 | KH RNA Binding Domain Containing, Signal Transduction Associated 1 | 1.35 |
| 2877 | TCP1 | T-Complex 1 | 1.34 |
| 2878 | KYAT1 | Kynurenine Aminotransferase 1 | 1.34 |
| 2879 | CCT7 | Chaperonin Containing TCP1 Subunit 7 | 1.34 |
| 2880 | ACTBL2 | Actin Beta Like 2 | 1.34 |
| 2881 | ETS2 | ETS Proto-Oncogene 2, Transcription Factor | 1.34 |
| 2882 | MAGI2 | Membrane Associated Guanylate Kinase, WW And PDZ Domain Containing 2 | 1.34 |
| 2883 | FBXW7 | F-Box And WD Repeat Domain Containing 7 | 1.34 |
| 2884 | RELB | RELB Proto-Oncogene, NF-KB Subunit | 1.34 |
| 2885 | PTGER1 | Prostaglandin E Receptor 1 | 1.33 |
| 2886 | SF1 | Splicing Factor 1 | 1.33 |
| 2887 | GGH | Gamma-Glutamyl Hydrolase | 1.33 |
| 2888 | ARHGEF12 | Rho Guanine Nucleotide Exchange Factor 12 | 1.33 |
| 2889 | IL18BP | Interleukin 18 Binding Protein | 1.33 |
| 2890 | PTGFR | Prostaglandin F Receptor | 1.33 |
| 2891 | GPI | Glucose-6-Phosphate Isomerase | 1.33 |
| 2892 | GPHN | Gephyrin | 1.32 |
| 2893 | CNIH3 | Cornichon Family AMPA Receptor Auxiliary Protein 3 | 1.32 |
| 2894 | ANO10 | Anoctamin 10 | 1.32 |
| 2895 | CDKN2A-DT | CDKN2A Divergent Transcript | 1.32 |
| 2896 | USP24 | Ubiquitin Specific Peptidase 24 | 1.32 |
| 2897 | TIMM9 | Translocase Of Inner Mitochondrial Membrane 9 | 1.32 |
| 2898 | CHCHD4 | Coiled-Coil-Helix-Coiled-Coil-Helix Domain Containing 4 | 1.32 |
| 2899 | PCA3 | Prostate Cancer Associated 3 | 1.32 |
| 2900 | LINC01734 | Long Intergenic Non-Protein Coding RNA 1734 | 1.32 |
| 2901 | DFFB | DNA Fragmentation Factor Subunit Beta | 1.32 |
| 2902 | SEM1 | SEM1 26S Proteasome Complex Subunit | 1.32 |
| 2903 | PER3 | Period Circadian Regulator 3 | 1.32 |
| 2904 | NDUFAB1 | NADH:Ubiquinone Oxidoreductase Subunit AB1 | 1.32 |
| 2905 | CD163 | CD163 Molecule | 1.32 |
| 2906 | EEF1A2 | Eukaryotic Translation Elongation Factor 1 Alpha 2 | 1.32 |
| 2907 | LETM1 | Leucine Zipper And EF-Hand Containing Transmembrane Protein 1 | 1.32 |
| 2908 | CAMK1D | Calcium/Calmodulin Dependent Protein Kinase ID | 1.32 |
| 2909 | CRIM1 | Cysteine Rich Transmembrane BMP Regulator 1 | 1.31 |
| 2910 | MICB | MHC Class I Polypeptide-Related Sequence B | 1.31 |
| 2911 | SYNGR1 | Synaptogyrin 1 | 1.31 |
| 2912 | ST8SIA2 | ST8 Alpha-N-Acetyl-Neuraminide Alpha-2,8-Sialyltransferase 2 | 1.31 |
| 2913 | TAF4 | TATA-Box Binding Protein Associated Factor 4 | 1.31 |
| 2914 | ZNF395 | Zinc Finger Protein 395 | 1.31 |
| 2915 | SMAD5-AS1 | SMAD5 Antisense RNA 1 | 1.31 |
| 2916 | TRAF3IP2-AS1 | TRAF3IP2 Antisense RNA 1 | 1.31 |
| 2917 | LYZ | Lysozyme | 1.31 |
| 2918 | ABCG1 | ATP Binding Cassette Subfamily G Member 1 | 1.31 |
| 2919 | UGT8 | UDP Glycosyltransferase 8 | 1.31 |
| 2920 | CCKAR | Cholecystokinin A Receptor | 1.3 |
| 2921 | RAN | RAN, Member RAS Oncogene Family | 1.3 |
| 2922 | FASN | Fatty Acid Synthase | 1.3 |
| 2923 | CHN2 | Chimerin 2 | 1.3 |
| 2924 | GDI1 | GDP Dissociation Inhibitor 1 | 1.3 |
| 2925 | CLIP1 | CAP-Gly Domain Containing Linker Protein 1 | 1.3 |
| 2926 | UHMK1 | U2AF Homology Motif Kinase 1 | 1.29 |
| 2927 | ABI3 | ABI Family Member 3 | 1.29 |
| 2928 | ACAT1 | Acetyl-CoA Acetyltransferase 1 | 1.29 |
| 2929 | SIRT6 | Sirtuin 6 | 1.29 |
| 2930 | FDX1 | Ferredoxin 1 | 1.29 |
| 2931 | PRIMPOL | Primase And DNA Directed Polymerase | 1.29 |
| 2932 | UBE2A | Ubiquitin Conjugating Enzyme E2 A | 1.29 |
| 2933 | ABAT | 4-Aminobutyrate Aminotransferase | 1.29 |
| 2934 | ABCC4 | ATP Binding Cassette Subfamily C Member 4 | 1.29 |
| 2935 | AMACR | Alpha-Methylacyl-CoA Racemase | 1.29 |
| 2936 | AP5B1 | Adaptor Related Protein Complex 5 Subunit Beta 1 | 1.29 |
| 2937 | GRIP1 | Glutamate Receptor Interacting Protein 1 | 1.29 |
| 2938 | MEGF10 | Multiple EGF Like Domains 10 | 1.29 |
| 2939 | COG5 | Component Of Oligomeric Golgi Complex 5 | 1.29 |
| 2940 | PTS | 6-Pyruvoyltetrahydropterin Synthase | 1.28 |
| 2941 | NDEL1 | NudE Neurodevelopment Protein 1 Like 1 | 1.28 |
| 2942 | TMPRSS6 | Transmembrane Serine Protease 6 | 1.28 |
| 2943 | VTI1A | Vesicle Transport Through Interaction With T-SNAREs 1A | 1.28 |
| 2944 | PRDX5 | Peroxiredoxin 5 | 1.28 |
| 2945 | BAG1 | BAG Cochaperone 1 | 1.28 |
| 2946 | PRDM5 | PR/SET Domain 5 | 1.28 |
| 2947 | HEXD | Hexosaminidase D | 1.28 |
| 2948 | SLC8A1-AS1 | SLC8A1 Antisense RNA 1 | 1.28 |
| 2949 | F8A1 | Coagulation Factor VIII Associated 1 | 1.27 |
| 2950 | IMPDH2 | Inosine Monophosphate Dehydrogenase 2 | 1.27 |
| 2951 | DOCK4 | Dedicator Of Cytokinesis 4 | 1.27 |
| 2952 | INPP5D | Inositol Polyphosphate-5-Phosphatase D | 1.27 |
| 2953 | CXCR1 | C-X-C Motif Chemokine Receptor 1 | 1.27 |
| 2954 | SLC22A3 | Solute Carrier Family 22 Member 3 | 1.27 |
| 2955 | SRP54 | Signal Recognition Particle 54 | 1.27 |
| 2956 | GRM3 | Glutamate Metabotropic Receptor 3 | 1.26 |
| 2957 | SAR1B | Secretion Associated Ras Related GTPase 1B | 1.26 |
| 2958 | KCNH2 | Potassium Voltage-Gated Channel Subfamily H Member 2 | 1.26 |
| 2959 | SH3GL3 | SH3 Domain Containing GRB2 Like 3, Endophilin A3 | 1.26 |
| 2960 | LINC01721 | Long Intergenic Non-Protein Coding RNA 1721 | 1.25 |
| 2961 | HERPUD1 | Homocysteine Inducible ER Protein With Ubiquitin Like Domain 1 | 1.25 |
| 2962 | RPLP0 | Ribosomal Protein Lateral Stalk Subunit P0 | 1.25 |
| 2963 | AP2B1 | Adaptor Related Protein Complex 2 Subunit Beta 1 | 1.25 |
| 2964 | BCL2L11 | BCL2 Like 11 | 1.24 |
| 2965 | PDE11A | Phosphodiesterase 11A | 1.24 |
| 2966 | ACOX1 | Acyl-CoA Oxidase 1 | 1.24 |
| 2967 | GRIK4 | Glutamate Ionotropic Receptor Kainate Type Subunit 4 | 1.24 |
| 2968 | CALY | Calcyon Neuron Specific Vesicular Protein | 1.24 |
| 2969 | HIVEP1 | HIVEP Zinc Finger 1 | 1.24 |
| 2970 | DNAJC14 | DnaJ Heat Shock Protein Family (Hsp40) Member C14 | 1.24 |
| 2971 | GPRIN1 | G Protein Regulated Inducer Of Neurite Outgrowth 1 | 1.24 |
| 2972 | YLPM1 | YLP Motif Containing 1 | 1.24 |
| 2973 | F8A2 | Coagulation Factor VIII Associated 2 | 1.24 |
| 2974 | MIR137HG | MIR137 Host Gene | 1.24 |
| 2975 | ATP1B3 | ATPase Na+/K+ Transporting Subunit Beta 3 | 1.24 |
| 2976 | PTPRQ | Protein Tyrosine Phosphatase Receptor Type Q | 1.24 |
| 2977 | STEAP1 | STEAP Family Member 1 | 1.24 |
| 2978 | PRKDC | Protein Kinase, DNA-Activated, Catalytic Subunit | 1.23 |
| 2979 | PTK7 | Protein Tyrosine Kinase 7 (Inactive) | 1.23 |
| 2980 | DNAJB1 | DnaJ Heat Shock Protein Family (Hsp40) Member B1 | 1.23 |
| 2981 | SFMBT1 | Scm Like With Four Mbt Domains 1 | 1.23 |
| 2982 | BTRC | Beta-Transducin Repeat Containing E3 Ubiquitin Protein Ligase | 1.23 |
| 2983 | DDX1 | DEAD-Box Helicase 1 | 1.23 |
| 2984 | WNT1 | Wnt Family Member 1 | 1.23 |
| 2985 | MSRB3 | Methionine Sulfoxide Reductase B3 | 1.23 |
| 2986 | TRMU | TRNA 5-Methylaminomethyl-2-Thiouridylate Methyltransferase | 1.23 |
| 2987 | PTPN13 | Protein Tyrosine Phosphatase Non-Receptor Type 13 | 1.23 |
| 2988 | BAG2 | BAG Cochaperone 2 | 1.22 |
| 2989 | CCL8 | C-C Motif Chemokine Ligand 8 | 1.22 |
| 2990 | LAMB1 | Laminin Subunit Beta 1 | 1.22 |
| 2991 | ABCD3 | ATP Binding Cassette Subfamily D Member 3 | 1.22 |
| 2992 | INO80 | INO80 Complex ATPase Subunit | 1.22 |
| 2993 | DUSP3 | Dual Specificity Phosphatase 3 | 1.22 |
| 2994 | BMPER | BMP Binding Endothelial Regulator | 1.22 |
| 2995 | SHOC2 | SHOC2 Leucine Rich Repeat Scaffold Protein | 1.22 |
| 2996 | GSTT1 | Glutathione S-Transferase Theta 1 | 1.21 |
| 2997 | ACR | Acrosin | 1.21 |
| 2998 | ANK1 | Ankyrin 1 | 1.21 |
| 2999 | SERPINB9 | Serpin Family B Member 9 | 1.21 |
| 3000 | CDH10 | Cadherin 10 | 1.21 |
| 3001 | GYS2 | Glycogen Synthase 2 | 1.21 |
| 3002 | NOS1AP | Nitric Oxide Synthase 1 Adaptor Protein | 1.21 |
| 3003 | TNFRSF19 | TNF Receptor Superfamily Member 19 | 1.21 |
| 3004 | GALNT2 | Polypeptide N-Acetylgalactosaminyltransferase 2 | 1.21 |
| 3005 | GPR15 | G Protein-Coupled Receptor 15 | 1.21 |
| 3006 | MIR125B2 | MicroRNA 125b-2 | 1.2 |
| 3007 | CHRNA5 | Cholinergic Receptor Nicotinic Alpha 5 Subunit | 1.2 |
| 3008 | RBM4 | RNA Binding Motif Protein 4 | 1.2 |
| 3009 | FNDC5 | Fibronectin Type III Domain Containing 5 | 1.2 |
| 3010 | BRK1 | BRICK1 Subunit Of SCAR/WAVE Actin Nucleating Complex | 1.2 |
| 3011 | MIR138-2 | MicroRNA 138-2 | 1.2 |
| 3012 | NUDT6 | Nudix Hydrolase 6 | 1.19 |
| 3013 | NLGN2 | Neuroligin 2 | 1.19 |
| 3014 | SKP1 | S-Phase Kinase Associated Protein 1 | 1.19 |
| 3015 | PFKL | Phosphofructokinase, Liver Type | 1.18 |
| 3016 | MTHFD1 | Methylenetetrahydrofolate Dehydrogenase, Cyclohydrolase And Formyltetrahydrofolate Synthetase 1 | 1.18 |
| 3017 | PIK3C2B | Phosphatidylinositol-4-Phosphate 3-Kinase Catalytic Subunit Type 2 Beta | 1.18 |
| 3018 | FGF18 | Fibroblast Growth Factor 18 | 1.18 |
| 3019 | GAB2 | GRB2 Associated Binding Protein 2 | 1.18 |
| 3020 | MDC1 | Mediator Of DNA Damage Checkpoint 1 | 1.18 |
| 3021 | SLC12A5 | Solute Carrier Family 12 Member 5 | 1.17 |
| 3022 | MYT1L | Myelin Transcription Factor 1 Like | 1.17 |
| 3023 | USP2 | Ubiquitin Specific Peptidase 2 | 1.17 |
| 3024 | COL6A1 | Collagen Type VI Alpha 1 Chain | 1.17 |
| 3025 | FOXQ1 | Forkhead Box Q1 | 1.17 |
| 3026 | HUS1B | HUS1 Checkpoint Clamp Component B | 1.17 |
| 3027 | TGFB1I1 | Transforming Growth Factor Beta 1 Induced Transcript 1 | 1.17 |
| 3028 | LRSAM1 | Leucine Rich Repeat And Sterile Alpha Motif Containing 1 | 1.17 |
| 3029 | SCRG1 | Stimulator Of Chondrogenesis 1 | 1.17 |
| 3030 | EFNA3 | Ephrin A3 | 1.17 |
| 3031 | ACACA | Acetyl-CoA Carboxylase Alpha | 1.17 |
| 3032 | CLCN2 | Chloride Voltage-Gated Channel 2 | 1.17 |
| 3033 | IGFBP6 | Insulin Like Growth Factor Binding Protein 6 | 1.17 |
| 3034 | CHRNA3 | Cholinergic Receptor Nicotinic Alpha 3 Subunit | 1.17 |
| 3035 | PLXNA3 | Plexin A3 | 1.17 |
| 3036 | SHROOM4 | Shroom Family Member 4 | 1.17 |
| 3037 | MCM7 | Minichromosome Maintenance Complex Component 7 | 1.17 |
| 3038 | NEUROD6 | Neuronal Differentiation 6 | 1.16 |
| 3039 | NDUFS1 | NADH:Ubiquinone Oxidoreductase Core Subunit S1 | 1.16 |
| 3040 | COQ10A | Coenzyme Q10A | 1.16 |
| 3041 | IFNGR2 | Interferon Gamma Receptor 2 | 1.16 |
| 3042 | NCK2 | NCK Adaptor Protein 2 | 1.16 |
| 3043 | NMBR | Neuromedin B Receptor | 1.16 |
| 3044 | IGF2BP2 | Insulin Like Growth Factor 2 MRNA Binding Protein 2 | 1.16 |
| 3045 | SERPINA6 | Serpin Family A Member 6 | 1.16 |
| 3046 | PCM1 | Pericentriolar Material 1 | 1.16 |
| 3047 | YWHAH | Tyrosine 3-Monooxygenase/Tryptophan 5-Monooxygenase Activation Protein Eta | 1.15 |
| 3048 | GABRB1 | Gamma-Aminobutyric Acid Type A Receptor Subunit Beta1 | 1.15 |
| 3049 | CDK20 | Cyclin Dependent Kinase 20 | 1.15 |
| 3050 | PSMC4 | Proteasome 26S Subunit, ATPase 4 | 1.15 |
| 3051 | GCNT2 | Glucosaminyl (N-Acetyl) Transferase 2 (I Blood Group) | 1.15 |
| 3052 | EME1 | Essential Meiotic Structure-Specific Endonuclease 1 | 1.15 |
| 3053 | RBM17 | RNA Binding Motif Protein 17 | 1.15 |
| 3054 | IER5L | Immediate Early Response 5 Like | 1.15 |
| 3055 | PMCH | Pro-Melanin Concentrating Hormone | 1.15 |
| 3056 | GABRB2 | Gamma-Aminobutyric Acid Type A Receptor Subunit Beta2 | 1.15 |
| 3057 | KCNQ3 | Potassium Voltage-Gated Channel Subfamily Q Member 3 | 1.15 |
| 3058 | GLRA2 | Glycine Receptor Alpha 2 | 1.15 |
| 3059 | GRM7 | Glutamate Metabotropic Receptor 7 | 1.15 |
| 3060 | NTSR1 | Neurotensin Receptor 1 | 1.15 |
| 3061 | NRG3 | Neuregulin 3 | 1.15 |
| 3062 | DLG2 | Discs Large MAGUK Scaffold Protein 2 | 1.15 |
| 3063 | TET3 | Tet Methylcytosine Dioxygenase 3 | 1.15 |
| 3064 | PIGL | Phosphatidylinositol Glycan Anchor Biosynthesis Class L | 1.14 |
| 3065 | SLC35A2 | Solute Carrier Family 35 Member A2 | 1.14 |
| 3066 | KPNB1 | Karyopherin Subunit Beta 1 | 1.14 |
| 3067 | PI4KA | Phosphatidylinositol 4-Kinase Alpha | 1.14 |
| 3068 | DLG1 | Discs Large MAGUK Scaffold Protein 1 | 1.14 |
| 3069 | HNRNPL | Heterogeneous Nuclear Ribonucleoprotein L | 1.13 |
| 3070 | ELF5 | E74 Like ETS Transcription Factor 5 | 1.13 |
| 3071 | RPS3 | Ribosomal Protein S3 | 1.13 |
| 3072 | ALDH1L1 | Aldehyde Dehydrogenase 1 Family Member L1 | 1.13 |
| 3073 | RAD23B | RAD23 Homolog B, Nucleotide Excision Repair Protein | 1.13 |
| 3074 | RPS5 | Ribosomal Protein S5 | 1.13 |
| 3075 | USP9X | Ubiquitin Specific Peptidase 9 X-Linked | 1.13 |
| 3076 | ZNRD2 | Zinc Ribbon Domain Containing 2 | 1.12 |
| 3077 | GDF6 | Growth Differentiation Factor 6 | 1.12 |
| 3078 | NSDHL | NAD(P) Dependent Steroid Dehydrogenase-Like | 1.12 |
| 3079 | GRPR | Gastrin Releasing Peptide Receptor | 1.12 |
| 3080 | SF3B4 | Splicing Factor 3b Subunit 4 | 1.12 |
| 3081 | NSFL1C | NSFL1 Cofactor | 1.12 |
| 3082 | H3C14 | H3 Clustered Histone 14 | 1.12 |
| 3083 | DDX3X | DEAD-Box Helicase 3 X-Linked | 1.12 |
| 3084 | ANK3 | Ankyrin 3 | 1.11 |
| 3085 | VIPR2 | Vasoactive Intestinal Peptide Receptor 2 | 1.11 |
| 3086 | NLGN3 | Neuroligin 3 | 1.11 |
| 3087 | SERPINA4 | Serpin Family A Member 4 | 1.11 |
| 3088 | RASD2 | RASD Family Member 2 | 1.11 |
| 3089 | SORCS2 | Sortilin Related VPS10 Domain Containing Receptor 2 | 1.11 |
| 3090 | B3GALT4 | Beta-1,3-Galactosyltransferase 4 | 1.11 |
| 3091 | AMIGO2 | Adhesion Molecule With Ig Like Domain 2 | 1.11 |
| 3092 | AP3B1 | Adaptor Related Protein Complex 3 Subunit Beta 1 | 1.1 |
| 3093 | RUNX3 | RUNX Family Transcription Factor 3 | 1.1 |
| 3094 | SATB2 | SATB Homeobox 2 | 1.1 |
| 3095 | REEP2 | Receptor Accessory Protein 2 | 1.1 |
| 3096 | GRK3 | G Protein-Coupled Receptor Kinase 3 | 1.1 |
| 3097 | NONO | Non-POU Domain Containing Octamer Binding | 1.1 |
| 3098 | UBE2G2 | Ubiquitin Conjugating Enzyme E2 G2 | 1.1 |
| 3099 | ARFGAP1 | ADP Ribosylation Factor GTPase Activating Protein 1 | 1.1 |
| 3100 | XPNPEP1 | X-Prolyl Aminopeptidase 1 | 1.1 |
| 3101 | ELOVL1 | ELOVL Fatty Acid Elongase 1 | 1.1 |
| 3102 | MRPL44 | Mitochondrial Ribosomal Protein L44 | 1.1 |
| 3103 | EHD4 | EH Domain Containing 4 | 1.1 |
| 3104 | ATXN3L | Ataxin 3 Like | 1.1 |
| 3105 | ASPRV1 | Aspartic Peptidase Retroviral Like 1 | 1.1 |
| 3106 | TIMELESS | Timeless Circadian Regulator | 1.09 |
| 3107 | TET1 | Tet Methylcytosine Dioxygenase 1 | 1.09 |
| 3108 | PCDH17 | Protocadherin 17 | 1.09 |
| 3109 | GLS2 | Glutaminase 2 | 1.09 |
| 3110 | MAT1A | Methionine Adenosyltransferase 1A | 1.09 |
| 3111 | LOXL2 | Lysyl Oxidase Like 2 | 1.09 |
| 3112 | SHANK1 | SH3 And Multiple Ankyrin Repeat Domains 1 | 1.09 |
| 3113 | SNORD35A | Small Nucleolar RNA, C/D Box 35A | 1.09 |
| 3114 | BBC3 | BCL2 Binding Component 3 | 1.09 |
| 3115 | ISYNA1 | Inositol-3-Phosphate Synthase 1 | 1.08 |
| 3116 | GABRA5 | Gamma-Aminobutyric Acid Type A Receptor Subunit Alpha5 | 1.08 |
| 3117 | GABRA2 | Gamma-Aminobutyric Acid Type A Receptor Subunit Alpha2 | 1.08 |
| 3118 | TDO2 | Tryptophan 2,3-Dioxygenase | 1.08 |
| 3119 | GABRA3 | Gamma-Aminobutyric Acid Type A Receptor Subunit Alpha3 | 1.08 |
| 3120 | MBD4 | Methyl-CpG Binding Domain 4, DNA Glycosylase | 1.08 |
| 3121 | UBE2K | Ubiquitin Conjugating Enzyme E2 K | 1.08 |
| 3122 | ITIH3 | Inter-Alpha-Trypsin Inhibitor Heavy Chain 3 | 1.08 |
| 3123 | SAMD12 | Sterile Alpha Motif Domain Containing 12 | 1.08 |
| 3124 | KCTD13 | Potassium Channel Tetramerization Domain Containing 13 | 1.08 |
| 3125 | MT-TA | Mitochondrially Encoded TRNA-Ala (GCN) | 1.08 |
| 3126 | KCNMB3 | Potassium Calcium-Activated Channel Subfamily M Regulatory Beta Subunit 3 | 1.08 |
| 3127 | RFNG | RFNG O-Fucosylpeptide 3-Beta-N-Acetylglucosaminyltransferase | 1.08 |
| 3128 | FAAP100 | FA Core Complex Associated Protein 100 | 1.08 |
| 3129 | NUB1 | Negative Regulator Of Ubiquitin Like Proteins 1 | 1.08 |
| 3130 | PRDX4 | Peroxiredoxin 4 | 1.08 |
| 3131 | FBXO3 | F-Box Protein 3 | 1.08 |
| 3132 | VPS13D | Vacuolar Protein Sorting 13 Homolog D | 1.08 |
| 3133 | MIR338 | MicroRNA 338 | 1.08 |
| 3134 | NR2C2 | Nuclear Receptor Subfamily 2 Group C Member 2 | 1.08 |
| 3135 | HSPA12B | Heat Shock Protein Family A (Hsp70) Member 12B | 1.08 |
| 3136 | PPIH | Peptidylprolyl Isomerase H | 1.07 |
| 3137 | IGF2BP1 | Insulin Like Growth Factor 2 MRNA Binding Protein 1 | 1.07 |
| 3138 | CELSR2 | Cadherin EGF LAG Seven-Pass G-Type Receptor 2 | 1.07 |
| 3139 | RARS1 | Arginyl-TRNA Synthetase 1 | 1.07 |
| 3140 | PCSK7 | Proprotein Convertase Subtilisin/Kexin Type 7 | 1.07 |
| 3141 | MMP16 | Matrix Metallopeptidase 16 | 1.07 |
| 3142 | STAG3L4 | Stromal Antigen 3-Like 4 (Pseudogene) | 1.07 |
| 3143 | NSUN2 | NOP2/Sun RNA Methyltransferase 2 | 1.07 |
| 3144 | RPL11 | Ribosomal Protein L11 | 1.06 |
| 3145 | CEP170 | Centrosomal Protein 170 | 1.06 |
| 3146 | NAPRT | Nicotinate Phosphoribosyltransferase | 1.06 |
| 3147 | DUS1L | Dihydrouridine Synthase 1 Like | 1.06 |
| 3148 | SERPINB7 | Serpin Family B Member 7 | 1.06 |
| 3149 | BCAM | Basal Cell Adhesion Molecule (Lutheran Blood Group) | 1.06 |
| 3150 | NME8 | NME/NM23 Family Member 8 | 1.06 |
| 3151 | CCT8 | Chaperonin Containing TCP1 Subunit 8 | 1.05 |
| 3152 | LDHC | Lactate Dehydrogenase C | 1.05 |
| 3153 | RBMX | RNA Binding Motif Protein X-Linked | 1.05 |
| 3154 | ADAM12 | ADAM Metallopeptidase Domain 12 | 1.05 |
| 3155 | PLCB2 | Phospholipase C Beta 2 | 1.05 |
| 3156 | ASRGL1 | Asparaginase And Isoaspartyl Peptidase 1 | 1.05 |
| 3157 | ASPM | Assembly Factor For Spindle Microtubules | 1.05 |
| 3158 | HIVEP3 | HIVEP Zinc Finger 3 | 1.04 |
| 3159 | POGZ | Pogo Transposable Element Derived With ZNF Domain | 1.04 |
| 3160 | TICAM1 | Toll Like Receptor Adaptor Molecule 1 | 1.04 |
| 3161 | NOVA2 | NOVA Alternative Splicing Regulator 2 | 1.04 |
| 3162 | CNTNAP5 | Contactin Associated Protein Family Member 5 | 1.04 |
| 3163 | LACTB | Lactamase Beta | 1.04 |
| 3164 | SF3B1 | Splicing Factor 3b Subunit 1 | 1.04 |
| 3165 | NTN4 | Netrin 4 | 1.04 |
| 3166 | USP16 | Ubiquitin Specific Peptidase 16 | 1.04 |
| 3167 | MAPK13 | Mitogen-Activated Protein Kinase 13 | 1.04 |
| 3168 | SERPINA12 | Serpin Family A Member 12 | 1.04 |
| 3169 | SMARCAL1 | SWI/SNF Related, Matrix Associated, Actin Dependent Regulator Of Chromatin, Subfamily A Like 1 | 1.04 |
| 3170 | EXOSC2 | Exosome Component 2 | 1.03 |
| 3171 | GGA1 | Golgi Associated, Gamma Adaptin Ear Containing, ARF Binding Protein 1 | 1.03 |
| 3172 | MIRLET7B | MicroRNA Let-7b | 1.03 |
| 3173 | PNPLA2 | Patatin Like Phospholipase Domain Containing 2 | 1.02 |
| 3174 | TUBG1 | Tubulin Gamma 1 | 1.02 |
| 3175 | NAT1 | N-Acetyltransferase 1 | 1.02 |
| 3176 | TMPO | Thymopoietin | 1.02 |
| 3177 | GPR1 | G Protein-Coupled Receptor 1 | 1.02 |
| 3178 | NEIL2 | Nei Like DNA Glycosylase 2 | 1.02 |
| 3179 | USF2 | Upstream Transcription Factor 2, C-Fos Interacting | 1.02 |
| 3180 | ZMYND8 | Zinc Finger MYND-Type Containing 8 | 1.02 |
| 3181 | SLC7A4 | Solute Carrier Family 7 Member 4 | 1.02 |
| 3182 | SEMA3C | Semaphorin 3C | 1.02 |
| 3183 | BUB3 | BUB3 Mitotic Checkpoint Protein | 1.02 |
| 3184 | IGBP1 | Immunoglobulin Binding Protein 1 | 1.01 |
| 3185 | ZYX | Zyxin | 1.01 |
| 3186 | HBD | Hemoglobin Subunit Delta | 1.01 |
| 3187 | WDR13 | WD Repeat Domain 13 | 1.01 |
| 3188 | FOXE1 | Forkhead Box E1 | 1.01 |
| 3189 | STX11 | Syntaxin 11 | 1.01 |
| 3190 | OMP | Olfactory Marker Protein | 1.01 |
| 3191 | SERPINA5 | Serpin Family A Member 5 | 1.01 |
| 3192 | ZCCHC13 | Zinc Finger CCHC-Type Containing 13 | 1.01 |
| 3193 | MCM6 | Minichromosome Maintenance Complex Component 6 | 1.01 |
| 3194 | RAB4A | RAB4A, Member RAS Oncogene Family | 1.01 |
| 3195 | HCAR2 | Hydroxycarboxylic Acid Receptor 2 | 1.01 |
| 3196 | MIR29B2 | MicroRNA 29b-2 | 1 |
| 3197 | THADA | THADA Armadillo Repeat Containing | 1 |
| 3198 | KNTC1 | Kinetochore Associated 1 | 1 |
| 3199 | SNAI3 | Snail Family Transcriptional Repressor 3 | 1 |
| 3200 | MTCL1 | Microtubule Crosslinking Factor 1 | 1 |
| 3201 | FAM166B | Family With Sequence Similarity 166 Member B | 1 |
| 3202 | DNAJC3 | DnaJ Heat Shock Protein Family (Hsp40) Member C3 | 1 |
| 3203 | A2ML1 | Alpha-2-Macroglobulin Like 1 | 1 |
| 3204 | CIC | Capicua Transcriptional Repressor | 1 |
| 3205 | CPS1 | Carbamoyl-Phosphate Synthase 1 | 1 |
| 3206 | WASF1 | WASP Family Member 1 | 1 |
| 3207 | SLC22A2 | Solute Carrier Family 22 Member 2 | 1 |
| 3208 | H3-3A | H3.3 Histone A | 1 |
| 3209 | H3-3B | H3.3 Histone B | 1 |
| 3210 | PPP1CA | Protein Phosphatase 1 Catalytic Subunit Alpha | 1 |
| 3211 | BDH1 | 3-Hydroxybutyrate Dehydrogenase 1 | 0.99 |
| 3212 | ASH1L | ASH1 Like Histone Lysine Methyltransferase | 0.99 |
| 3213 | RUSF1 | RUS Family Member 1 | 0.99 |
| 3214 | ZIC4 | Zic Family Member 4 | 0.99 |
| 3215 | ANP32A | Acidic Nuclear Phosphoprotein 32 Family Member A | 0.99 |
| 3216 | AKAP13 | A-Kinase Anchoring Protein 13 | 0.99 |
| 3217 | FLRT2 | Fibronectin Leucine Rich Transmembrane Protein 2 | 0.99 |
| 3218 | UNC13D | Unc-13 Homolog D | 0.99 |
| 3219 | CCDC115 | Coiled-Coil Domain Containing 115 | 0.99 |
| 3220 | RPL7 | Ribosomal Protein L7 | 0.99 |
| 3221 | FGF13 | Fibroblast Growth Factor 13 | 0.99 |
| 3222 | CCDC57 | Coiled-Coil Domain Containing 57 | 0.99 |
| 3223 | RABL2B | RAB, Member Of RAS Oncogene Family Like 2B | 0.99 |
| 3224 | LINC01588 | Long Intergenic Non-Protein Coding RNA 1588 | 0.99 |
| 3225 | RNU6-189P | RNA, U6 Small Nuclear 189, Pseudogene | 0.99 |
| 3226 | ENSG00000265678 |  | 0.99 |
| 3227 | ENSG00000282885 |  | 0.99 |
| 3228 | lnc-NEMF-2 |  | 0.99 |
| 3229 | RAB25 | RAB25, Member RAS Oncogene Family | 0.99 |
| 3230 | CYTH1 | Cytohesin 1 | 0.98 |
| 3231 | CYP2A6 | Cytochrome P450 Family 2 Subfamily A Member 6 | 0.98 |
| 3232 | DPP7 | Dipeptidyl Peptidase 7 | 0.98 |
| 3233 | LIG3 | DNA Ligase 3 | 0.98 |
| 3234 | CCNI | Cyclin I | 0.98 |
| 3235 | CLDN3 | Claudin 3 | 0.98 |
| 3236 | SEC23IP | SEC23 Interacting Protein | 0.97 |
| 3237 | EDAR | Ectodysplasin A Receptor | 0.97 |
| 3238 | NTPCR | Nucleoside-Triphosphatase, Cancer-Related | 0.97 |
| 3239 | CREM | CAMP Responsive Element Modulator | 0.97 |
| 3240 | PYGM | Glycogen Phosphorylase, Muscle Associated | 0.97 |
| 3241 | NDUFB3 | NADH:Ubiquinone Oxidoreductase Subunit B3 | 0.97 |
| 3242 | DNAH11 | Dynein Axonemal Heavy Chain 11 | 0.97 |
| 3243 | HERC2 | HECT And RLD Domain Containing E3 Ubiquitin Protein Ligase 2 | 0.97 |
| 3244 | HLA-E | Major Histocompatibility Complex, Class I, E | 0.97 |
| 3245 | HLA-C | Major Histocompatibility Complex, Class I, C | 0.97 |
| 3246 | RBM14 | RNA Binding Motif Protein 14 | 0.96 |
| 3247 | DNM3 | Dynamin 3 | 0.96 |
| 3248 | ELAVL2 | ELAV Like RNA Binding Protein 2 | 0.96 |
| 3249 | KLK7 | Kallikrein Related Peptidase 7 | 0.96 |
| 3250 | MT-TR | Mitochondrially Encoded TRNA-Arg (CGN) | 0.96 |
| 3251 | IL1R2 | Interleukin 1 Receptor Type 2 | 0.96 |
| 3252 | THUMPD2 | THUMP Domain Containing 2 | 0.96 |
| 3253 | CALU | Calumenin | 0.96 |
| 3254 | UBA2 | Ubiquitin Like Modifier Activating Enzyme 2 | 0.95 |
| 3255 | PARD6G | Par-6 Family Cell Polarity Regulator Gamma | 0.95 |
| 3256 | SV2A | Synaptic Vesicle Glycoprotein 2A | 0.95 |
| 3257 | NMNAT2 | Nicotinamide Nucleotide Adenylyltransferase 2 | 0.94 |
| 3258 | MCPH1 | Microcephalin 1 | 0.94 |
| 3259 | DCUN1D1 | Defective In Cullin Neddylation 1 Domain Containing 1 | 0.94 |
| 3260 | ABCD2 | ATP Binding Cassette Subfamily D Member 2 | 0.94 |
| 3261 | TRIM2 | Tripartite Motif Containing 2 | 0.94 |
| 3262 | MIR6084 | MicroRNA 6084 | 0.94 |
| 3263 | GPX4 | Glutathione Peroxidase 4 | 0.94 |
| 3264 | HSD17B13 | Hydroxysteroid 17-Beta Dehydrogenase 13 | 0.94 |
| 3265 | DIABLO | Diablo IAP-Binding Mitochondrial Protein | 0.94 |
| 3266 | PPARGC1B | PPARG Coactivator 1 Beta | 0.93 |
| 3267 | NPC1L1 | NPC1 Like Intracellular Cholesterol Transporter 1 | 0.93 |
| 3268 | CNDP1 | Carnosine Dipeptidase 1 | 0.93 |
| 3269 | ANKRD13A | Ankyrin Repeat Domain 13A | 0.93 |
| 3270 | PPFIA2 | PTPRF Interacting Protein Alpha 2 | 0.93 |
| 3271 | CYHR1 | Cysteine And Histidine Rich 1 | 0.93 |
| 3272 | DIP2A | Disco Interacting Protein 2 Homolog A | 0.93 |
| 3273 | SPEN | Spen Family Transcriptional Repressor | 0.93 |
| 3274 | ELOA | Elongin A | 0.93 |
| 3275 | SFN | Stratifin | 0.93 |
| 3276 | CDK9 | Cyclin Dependent Kinase 9 | 0.93 |
| 3277 | USF1 | Upstream Transcription Factor 1 | 0.93 |
| 3278 | CARS2 | Cysteinyl-TRNA Synthetase 2, Mitochondrial | 0.93 |
| 3279 | SPTBN2 | Spectrin Beta, Non-Erythrocytic 2 | 0.93 |
| 3280 | GEMIN4 | Gem Nuclear Organelle Associated Protein 4 | 0.93 |
| 3281 | ATCAY | ATCAY Kinesin Light Chain Interacting Caytaxin | 0.93 |
| 3282 | AP4E1 | Adaptor Related Protein Complex 4 Subunit Epsilon 1 | 0.93 |
| 3283 | CHST7 | Carbohydrate Sulfotransferase 7 | 0.93 |
| 3284 | SLC9A7 | Solute Carrier Family 9 Member A7 | 0.93 |
| 3285 | PWP1 | PWP1 Homolog, Endonuclein | 0.93 |
| 3286 | ZBTB1 | Zinc Finger And BTB Domain Containing 1 | 0.93 |
| 3287 | ATXN1L | Ataxin 1 Like | 0.93 |
| 3288 | DESI1 | Desumoylating Isopeptidase 1 | 0.93 |
| 3289 | UQCC3 | Ubiquinol-Cytochrome C Reductase Complex Assembly Factor 3 | 0.93 |
| 3290 | FAAP24 | FA Core Complex Associated Protein 24 | 0.93 |
| 3291 | MMADHC-DT | MMADHC Divergent Transcript | 0.93 |
| 3292 | SDCCAG8 | SHH Signaling And Ciliogenesis Regulator SDCCAG8 | 0.93 |
| 3293 | RAB11FIP2 | RAB11 Family Interacting Protein 2 | 0.93 |
| 3294 | METAP2 | Methionyl Aminopeptidase 2 | 0.92 |
| 3295 | VGF | VGF Nerve Growth Factor Inducible | 0.92 |
| 3296 | ALDH3A2 | Aldehyde Dehydrogenase 3 Family Member A2 | 0.92 |
| 3297 | CDKN2C | Cyclin Dependent Kinase Inhibitor 2C | 0.92 |
| 3298 | RACK1 | Receptor For Activated C Kinase 1 | 0.92 |
| 3299 | CAMK4 | Calcium/Calmodulin Dependent Protein Kinase IV | 0.92 |
| 3300 | PRDM9 | PR/SET Domain 9 | 0.92 |
| 3301 | ADCY1 | Adenylate Cyclase 1 | 0.92 |
| 3302 | CCT6A | Chaperonin Containing TCP1 Subunit 6A | 0.92 |
| 3303 | GART | Phosphoribosylglycinamide Formyltransferase, Phosphoribosylglycinamide Synthetase, Phosphoribosylaminoimidazole Synthetase | 0.92 |
| 3304 | TSHR | Thyroid Stimulating Hormone Receptor | 0.92 |
| 3305 | GPR55 | G Protein-Coupled Receptor 55 | 0.92 |
| 3306 | WASL | WASP Like Actin Nucleation Promoting Factor | 0.92 |
| 3307 | EEF1D | Eukaryotic Translation Elongation Factor 1 Delta | 0.92 |
| 3308 | PSMD12 | Proteasome 26S Subunit, Non-ATPase 12 | 0.92 |
| 3309 | B3GAT3 | Beta-1,3-Glucuronyltransferase 3 | 0.91 |
| 3310 | EIF3I | Eukaryotic Translation Initiation Factor 3 Subunit I | 0.91 |
| 3311 | STIP1 | Stress Induced Phosphoprotein 1 | 0.91 |
| 3312 | TBX22 | T-Box Transcription Factor 22 | 0.91 |
| 3313 | CKAP4 | Cytoskeleton Associated Protein 4 | 0.91 |
| 3314 | CASP14 | Caspase 14 | 0.91 |
| 3315 | GHRHR | Growth Hormone Releasing Hormone Receptor | 0.91 |
| 3316 | GNAI2 | G Protein Subunit Alpha I2 | 0.91 |
| 3317 | DSTN | Destrin, Actin Depolymerizing Factor | 0.91 |
| 3318 | MAP4 | Microtubule Associated Protein 4 | 0.9 |
| 3319 | WIF1 | WNT Inhibitory Factor 1 | 0.9 |
| 3320 | HS3ST1 | Heparan Sulfate-Glucosamine 3-Sulfotransferase 1 | 0.9 |
| 3321 | PRDM2 | PR/SET Domain 2 | 0.9 |
| 3322 | LDHB | Lactate Dehydrogenase B | 0.9 |
| 3323 | GANAB | Glucosidase II Alpha Subunit | 0.9 |
| 3324 | TRADD | TNFRSF1A Associated Via Death Domain | 0.89 |
| 3325 | DOCK2 | Dedicator Of Cytokinesis 2 | 0.89 |
| 3326 | NINJ2 | Ninjurin 2 | 0.89 |
| 3327 | PRKCI | Protein Kinase C Iota | 0.89 |
| 3328 | FBXL18 | F-Box And Leucine Rich Repeat Protein 18 | 0.89 |
| 3329 | DSCR8 | Down Syndrome Critical Region 8 | 0.89 |
| 3330 | G3BP1 | G3BP Stress Granule Assembly Factor 1 | 0.89 |
| 3331 | DDX5 | DEAD-Box Helicase 5 | 0.89 |
| 3332 | MYOT | Myotilin | 0.89 |
| 3333 | GPR101 | G Protein-Coupled Receptor 101 | 0.89 |
| 3334 | SIGLEC1 | Sialic Acid Binding Ig Like Lectin 1 | 0.88 |
| 3335 | LRRC59 | Leucine Rich Repeat Containing 59 | 0.88 |
| 3336 | GJC2 | Gap Junction Protein Gamma 2 | 0.88 |
| 3337 | IQSEC1 | IQ Motif And Sec7 Domain ArfGEF 1 | 0.88 |
| 3338 | ABR | ABR Activator Of RhoGEF And GTPase | 0.88 |
| 3339 | CACUL1 | CDK2 Associated Cullin Domain 1 | 0.88 |
| 3340 | AATK | Apoptosis Associated Tyrosine Kinase | 0.87 |
| 3341 | SLC38A10 | Solute Carrier Family 38 Member 10 | 0.87 |
| 3342 | OXLD1 | Oxidoreductase Like Domain Containing 1 | 0.87 |
| 3343 | TEPSIN | TEPSIN Adaptor Related Protein Complex 4 Accessory Protein | 0.87 |
| 3344 | LINC01599 | Long Intergenic Non-Protein Coding RNA 1599 | 0.87 |
| 3345 | ENSG00000235426 |  | 0.87 |
| 3346 | lnc-TMEM105-1 |  | 0.87 |
| 3347 | AP3D1 | Adaptor Related Protein Complex 3 Subunit Delta 1 | 0.87 |
| 3348 | SUPT3H | SPT3 Homolog, SAGA And STAGA Complex Component | 0.87 |
| 3349 | STAU1 | Staufen Double-Stranded RNA Binding Protein 1 | 0.87 |
| 3350 | HLCS | Holocarboxylase Synthetase | 0.87 |
| 3351 | TNFRSF6B | TNF Receptor Superfamily Member 6b | 0.87 |
| 3352 | KPNA1 | Karyopherin Subunit Alpha 1 | 0.87 |
| 3353 | SNRPA1 | Small Nuclear Ribonucleoprotein Polypeptide A' | 0.87 |
| 3354 | IK | IK Cytokine | 0.86 |
| 3355 | RCAN2 | Regulator Of Calcineurin 2 | 0.86 |
| 3356 | CHRNA1 | Cholinergic Receptor Nicotinic Alpha 1 Subunit | 0.86 |
| 3357 | SERPINA10 | Serpin Family A Member 10 | 0.86 |
| 3358 | APOA4 | Apolipoprotein A4 | 0.86 |
| 3359 | B4GALT6 | Beta-1,4-Galactosyltransferase 6 | 0.86 |
| 3360 | CDH9 | Cadherin 9 | 0.86 |
| 3361 | NMT1 | N-Myristoyltransferase 1 | 0.85 |
| 3362 | RUNX1T1 | RUNX1 Partner Transcriptional Co-Repressor 1 | 0.85 |
| 3363 | COPB2 | COPI Coat Complex Subunit Beta 2 | 0.85 |
| 3364 | HARS2 | Histidyl-TRNA Synthetase 2, Mitochondrial | 0.85 |
| 3365 | ELOVL5 | ELOVL Fatty Acid Elongase 5 | 0.85 |
| 3366 | PTPRD | Protein Tyrosine Phosphatase Receptor Type D | 0.85 |
| 3367 | SLC2A2 | Solute Carrier Family 2 Member 2 | 0.85 |
| 3368 | ANGPTL3 | Angiopoietin Like 3 | 0.85 |
| 3369 | FUT1 | Fucosyltransferase 1 (H Blood Group) | 0.85 |
| 3370 | KLK11 | Kallikrein Related Peptidase 11 | 0.85 |
| 3371 | LTA4H | Leukotriene A4 Hydrolase | 0.85 |
| 3372 | GRHL3 | Grainyhead Like Transcription Factor 3 | 0.85 |
| 3373 | NRF1 | Nuclear Respiratory Factor 1 | 0.84 |
| 3374 | GCKR | Glucokinase Regulator | 0.84 |
| 3375 | MTDH | Metadherin | 0.84 |
| 3376 | ATP6V1G3 | ATPase H+ Transporting V1 Subunit G3 | 0.84 |
| 3377 | GTPBP3 | GTP Binding Protein 3, Mitochondrial | 0.84 |
| 3378 | PPP2R3C | Protein Phosphatase 2 Regulatory Subunit B''Gamma | 0.84 |
| 3379 | TOR1B | Torsin Family 1 Member B | 0.84 |
| 3380 | VWA1 | Von Willebrand Factor A Domain Containing 1 | 0.84 |
| 3381 | IARS1 | Isoleucyl-TRNA Synthetase 1 | 0.84 |
| 3382 | SERPINB6 | Serpin Family B Member 6 | 0.84 |
| 3383 | CTF1 | Cardiotrophin 1 | 0.84 |
| 3384 | CNTN1 | Contactin 1 | 0.83 |
| 3385 | ATG4C | Autophagy Related 4C Cysteine Peptidase | 0.83 |
| 3386 | PDE1A | Phosphodiesterase 1A | 0.83 |
| 3387 | STXBP2 | Syntaxin Binding Protein 2 | 0.83 |
| 3388 | FOLR1 | Folate Receptor Alpha | 0.83 |
| 3389 | H3C1 | H3 Clustered Histone 1 | 0.83 |
| 3390 | DNAJA1 | DnaJ Heat Shock Protein Family (Hsp40) Member A1 | 0.83 |
| 3391 | SIM1 | SIM BHLH Transcription Factor 1 | 0.83 |
| 3392 | PRKAR2A | Protein Kinase CAMP-Dependent Type II Regulatory Subunit Alpha | 0.83 |
| 3393 | RPL23A | Ribosomal Protein L23a | 0.82 |
| 3394 | NPFF | Neuropeptide FF-Amide Peptide Precursor | 0.82 |
| 3395 | ACAT2 | Acetyl-CoA Acetyltransferase 2 | 0.82 |
| 3396 | SLC25A27 | Solute Carrier Family 25 Member 27 | 0.82 |
| 3397 | CHRNA2 | Cholinergic Receptor Nicotinic Alpha 2 Subunit | 0.82 |
| 3398 | SLC5A3 | Solute Carrier Family 5 Member 3 | 0.82 |
| 3399 | MIA2 | MIA SH3 Domain ER Export Factor 2 | 0.82 |
| 3400 | ARID2 | AT-Rich Interaction Domain 2 | 0.82 |
| 3401 | CBR3 | Carbonyl Reductase 3 | 0.82 |
| 3402 | ITK | IL2 Inducible T Cell Kinase | 0.82 |
| 3403 | FCER2 | Fc Fragment Of IgE Receptor II | 0.82 |
| 3404 | NR2C2AP | Nuclear Receptor 2C2 Associated Protein | 0.82 |
| 3405 | SUMO4 | Small Ubiquitin Like Modifier 4 | 0.81 |
| 3406 | AHCY | Adenosylhomocysteinase | 0.81 |
| 3407 | FKBP4 | FKBP Prolyl Isomerase 4 | 0.81 |
| 3408 | ANXA4 | Annexin A4 | 0.81 |
| 3409 | SMURF2 | SMAD Specific E3 Ubiquitin Protein Ligase 2 | 0.81 |
| 3410 | COG7 | Component Of Oligomeric Golgi Complex 7 | 0.81 |
| 3411 | DCDC2 | Doublecortin Domain Containing 2 | 0.81 |
| 3412 | CCDC103 | Coiled-Coil Domain Containing 103 | 0.81 |
| 3413 | DNAAF4 | Dynein Axonemal Assembly Factor 4 | 0.81 |
| 3414 | CASP4 | Caspase 4 | 0.81 |
| 3415 | GCK | Glucokinase | 0.8 |
| 3416 | ZFHX3 | Zinc Finger Homeobox 3 | 0.8 |
| 3417 | HNRNPU | Heterogeneous Nuclear Ribonucleoprotein U | 0.8 |
| 3418 | CBR1 | Carbonyl Reductase 1 | 0.8 |
| 3419 | EIF2S3 | Eukaryotic Translation Initiation Factor 2 Subunit Gamma | 0.8 |
| 3420 | SLIT1 | Slit Guidance Ligand 1 | 0.8 |
| 3421 | LTB4R | Leukotriene B4 Receptor | 0.8 |
| 3422 | PLCE1 | Phospholipase C Epsilon 1 | 0.8 |
| 3423 | ZNF385D | Zinc Finger Protein 385D | 0.79 |
| 3424 | PTPMT1 | Protein Tyrosine Phosphatase Mitochondrial 1 | 0.79 |
| 3425 | MGST1 | Microsomal Glutathione S-Transferase 1 | 0.79 |
| 3426 | ARRDC4 | Arrestin Domain Containing 4 | 0.79 |
| 3427 | RPS14 | Ribosomal Protein S14 | 0.79 |
| 3428 | CMIP | C-Maf Inducing Protein | 0.78 |
| 3429 | CKS1B | CDC28 Protein Kinase Regulatory Subunit 1B | 0.78 |
| 3430 | PDGFRL | Platelet Derived Growth Factor Receptor Like | 0.78 |
| 3431 | EFHC1 | EF-Hand Domain Containing 1 | 0.78 |
| 3432 | NPSR1 | Neuropeptide S Receptor 1 | 0.78 |
| 3433 | MYO1H | Myosin IH | 0.78 |
| 3434 | C2orf49 | Chromosome 2 Open Reading Frame 49 | 0.78 |
| 3435 | MRM2 | Mitochondrial RRNA Methyltransferase 2 | 0.78 |
| 3436 | CRHBP | Corticotropin Releasing Hormone Binding Protein | 0.78 |
| 3437 | PACSIN2 | Protein Kinase C And Casein Kinase Substrate In Neurons 2 | 0.78 |
| 3438 | ACP3 | Acid Phosphatase 3 | 0.78 |
| 3439 | KLF7 | Kruppel Like Factor 7 | 0.78 |
| 3440 | PABPC1 | Poly(A) Binding Protein Cytoplasmic 1 | 0.78 |
| 3441 | FGF21 | Fibroblast Growth Factor 21 | 0.78 |
| 3442 | BMP1 | Bone Morphogenetic Protein 1 | 0.78 |
| 3443 | EXOSC8 | Exosome Component 8 | 0.78 |
| 3444 | ALDH1L2 | Aldehyde Dehydrogenase 1 Family Member L2 | 0.78 |
| 3445 | VAT1 | Vesicle Amine Transport 1 | 0.78 |
| 3446 | METTL18 | Methyltransferase Like 18 | 0.78 |
| 3447 | ACSL3 | Acyl-CoA Synthetase Long Chain Family Member 3 | 0.78 |
| 3448 | MARCKSL1 | MARCKS Like 1 | 0.78 |
| 3449 | SEMA6D | Semaphorin 6D | 0.77 |
| 3450 | CKMT2 | Creatine Kinase, Mitochondrial 2 | 0.77 |
| 3451 | SLC5A8 | Solute Carrier Family 5 Member 8 | 0.77 |
| 3452 | LONRF3 | LON Peptidase N-Terminal Domain And Ring Finger 3 | 0.77 |
| 3453 | PGA3 | Pepsinogen A3 | 0.77 |
| 3454 | MACROD2 | Mono-ADP Ribosylhydrolase 2 | 0.77 |
| 3455 | LIMS1 | LIM Zinc Finger Domain Containing 1 | 0.77 |
| 3456 | GET1 | Guided Entry Of Tail-Anchored Proteins Factor 1 | 0.77 |
| 3457 | DNAJC1 | DnaJ Heat Shock Protein Family (Hsp40) Member C1 | 0.77 |
| 3458 | CACNG8 | Calcium Voltage-Gated Channel Auxiliary Subunit Gamma 8 | 0.77 |
| 3459 | CACNG7 | Calcium Voltage-Gated Channel Auxiliary Subunit Gamma 7 | 0.77 |
| 3460 | THSD1 | Thrombospondin Type 1 Domain Containing 1 | 0.77 |
| 3461 | IRAK4 | Interleukin 1 Receptor Associated Kinase 4 | 0.76 |
| 3462 | WARS2 | Tryptophanyl TRNA Synthetase 2, Mitochondrial | 0.76 |
| 3463 | TRIM32 | Tripartite Motif Containing 32 | 0.76 |
| 3464 | NUP160 | Nucleoporin 160 | 0.76 |
| 3465 | CAMK1 | Calcium/Calmodulin Dependent Protein Kinase I | 0.76 |
| 3466 | STX12 | Syntaxin 12 | 0.76 |
| 3467 | DGKA | Diacylglycerol Kinase Alpha | 0.76 |
| 3468 | S100A7 | S100 Calcium Binding Protein A7 | 0.76 |
| 3469 | ABL2 | ABL Proto-Oncogene 2, Non-Receptor Tyrosine Kinase | 0.76 |
| 3470 | KIR2DS2 | Killer Cell Immunoglobulin Like Receptor, Two Ig Domains And Short Cytoplasmic Tail 2 | 0.75 |
| 3471 | ITSN1 | Intersectin 1 | 0.75 |
| 3472 | FASTKD2 | FAST Kinase Domains 2 | 0.75 |
| 3473 | HSPA6 | Heat Shock Protein Family A (Hsp70) Member 6 | 0.75 |
| 3474 | HSPA14 | Heat Shock Protein Family A (Hsp70) Member 14 | 0.75 |
| 3475 | AMPD1 | Adenosine Monophosphate Deaminase 1 | 0.75 |
| 3476 | UNC93B1 | Unc-93 Homolog B1, TLR Signaling Regulator | 0.75 |
| 3477 | ANKRD36B | Ankyrin Repeat Domain 36B | 0.75 |
| 3478 | SLC25A13 | Solute Carrier Family 25 Member 13 | 0.74 |
| 3479 | SORBS3 | Sorbin And SH3 Domain Containing 3 | 0.74 |
| 3480 | CDC42EP2 | CDC42 Effector Protein 2 | 0.74 |
| 3481 | GCLC | Glutamate-Cysteine Ligase Catalytic Subunit | 0.74 |
| 3482 | RPS26 | Ribosomal Protein S26 | 0.74 |
| 3483 | SMURF1 | SMAD Specific E3 Ubiquitin Protein Ligase 1 | 0.73 |
| 3484 | PSMB5 | Proteasome 20S Subunit Beta 5 | 0.73 |
| 3485 | PC | Pyruvate Carboxylase | 0.73 |
| 3486 | PSMG1 | Proteasome Assembly Chaperone 1 | 0.73 |
| 3487 | TUBA3C | Tubulin Alpha 3c | 0.73 |
| 3488 | DCAF1 | DDB1 And CUL4 Associated Factor 1 | 0.73 |
| 3489 | MTHFD2 | Methylenetetrahydrofolate Dehydrogenase (NADP+ Dependent) 2, Methenyltetrahydrofolate Cyclohydrolase | 0.73 |
| 3490 | SYNE1 | Spectrin Repeat Containing Nuclear Envelope Protein 1 | 0.73 |
| 3491 | MAP1LC3A | Microtubule Associated Protein 1 Light Chain 3 Alpha | 0.73 |
| 3492 | ADSS2 | Adenylosuccinate Synthase 2 | 0.73 |
| 3493 | KAT8 | Lysine Acetyltransferase 8 | 0.72 |
| 3494 | ATP9B | ATPase Phospholipid Transporting 9B (Putative) | 0.72 |
| 3495 | RNF40 | Ring Finger Protein 40 | 0.72 |
| 3496 | MRPL12 | Mitochondrial Ribosomal Protein L12 | 0.72 |
| 3497 | FCHSD1 | FCH And Double SH3 Domains 1 | 0.72 |
| 3498 | ZNF646 | Zinc Finger Protein 646 | 0.72 |
| 3499 | ZNF689 | Zinc Finger Protein 689 | 0.72 |
| 3500 | ZNF764 | Zinc Finger Protein 764 | 0.72 |
| 3501 | CEP131 | Centrosomal Protein 131 | 0.72 |
| 3502 | GPRIN3 | GPRIN Family Member 3 | 0.72 |
| 3503 | SYF2 | SYF2 Pre-MRNA Splicing Factor | 0.72 |
| 3504 | ZNF785 | Zinc Finger Protein 785 | 0.72 |
| 3505 | CCDC137 | Coiled-Coil Domain Containing 137 | 0.72 |
| 3506 | FBRS | Fibrosin | 0.72 |
| 3507 | PRR14 | Proline Rich 14 | 0.72 |
| 3508 | L3HYPDH | Trans-L-3-Hydroxyproline Dehydratase | 0.72 |
| 3509 | ZCCHC24 | Zinc Finger CCHC-Type Containing 24 | 0.72 |
| 3510 | ZNF747 | Zinc Finger Protein 747 | 0.72 |
| 3511 | ZNF688 | Zinc Finger Protein 688 | 0.72 |
| 3512 | ZNF629 | Zinc Finger Protein 629 | 0.72 |
| 3513 | TMEM105 | TMEM105 Long Non-Coding RNA | 0.72 |
| 3514 | DELE1 | DAP3 Binding Cell Death Enhancer 1 | 0.72 |
| 3515 | ECRG4 | ECRG4 Augurin Precursor | 0.72 |
| 3516 | CCDC189 | Coiled-Coil Domain Containing 189 | 0.72 |
| 3517 | NDUFAF8 | NADH:Ubiquinone Oxidoreductase Complex Assembly Factor 8 | 0.72 |
| 3518 | CFAP54 | Cilia And Flagella Associated Protein 54 | 0.72 |
| 3519 | LOC105375744 | Uncharacterized LOC105375744 | 0.72 |
| 3520 | HMGN2P19 | High Mobility Group Nucleosomal Binding Domain 2 Pseudogene 19 | 0.72 |
| 3521 | SFTPD-AS1 | SFTPD Antisense RNA 1 | 0.72 |
| 3522 | WASHC5-AS1 | WASHC5 Antisense RNA 1 | 0.72 |
| 3523 | RF00017-4973 |  | 0.72 |
| 3524 | RF00017-4976 |  | 0.72 |
| 3525 | RF00017-4975 |  | 0.72 |
| 3526 | lnc-ARF6-10 |  | 0.72 |
| 3527 | lnc-ARF6-2 |  | 0.72 |
| 3528 | lnc-SFTPA2-6 |  | 0.72 |
| 3529 | RF00697-002 |  | 0.72 |
| 3530 | ARHGAP29 | Rho GTPase Activating Protein 29 | 0.72 |
| 3531 | KIR2DL2 | Killer Cell Immunoglobulin Like Receptor, Two Ig Domains And Long Cytoplasmic Tail 2 | 0.72 |
| 3532 | MCM3 | Minichromosome Maintenance Complex Component 3 | 0.72 |
| 3533 | EIF3A | Eukaryotic Translation Initiation Factor 3 Subunit A | 0.72 |
| 3534 | AZGP1 | Alpha-2-Glycoprotein 1, Zinc-Binding | 0.72 |
| 3535 | PAF1 | PAF1 Homolog, Paf1/RNA Polymerase II Complex Component | 0.72 |
| 3536 | ICMT | Isoprenylcysteine Carboxyl Methyltransferase | 0.72 |
| 3537 | MIR613 | MicroRNA 613 | 0.71 |
| 3538 | AAAS | Aladin WD Repeat Nucleoporin | 0.71 |
| 3539 | TRIO | Trio Rho Guanine Nucleotide Exchange Factor | 0.71 |
| 3540 | SF3B2 | Splicing Factor 3b Subunit 2 | 0.71 |
| 3541 | COPA | COPI Coat Complex Subunit Alpha | 0.71 |
| 3542 | MTO1 | Mitochondrial TRNA Translation Optimization 1 | 0.71 |
| 3543 | AP3B2 | Adaptor Related Protein Complex 3 Subunit Beta 2 | 0.71 |
| 3544 | DGKI | Diacylglycerol Kinase Iota | 0.71 |
| 3545 | CXXC5 | CXXC Finger Protein 5 | 0.71 |
| 3546 | PDE7B | Phosphodiesterase 7B | 0.71 |
| 3547 | STARD7 | StAR Related Lipid Transfer Domain Containing 7 | 0.71 |
| 3548 | IRGC | Immunity Related GTPase Cinema | 0.71 |
| 3549 | STMN4 | Stathmin 4 | 0.71 |
| 3550 | ZNF346 | Zinc Finger Protein 346 | 0.71 |
| 3551 | MBD6 | Methyl-CpG Binding Domain Protein 6 | 0.71 |
| 3552 | TMED4 | Transmembrane P24 Trafficking Protein 4 | 0.71 |
| 3553 | SLC25A41 | Solute Carrier Family 25 Member 41 | 0.71 |
| 3554 | KDF1 | Keratinocyte Differentiation Factor 1 | 0.71 |
| 3555 | SIMC1 | SUMO Interacting Motifs Containing 1 | 0.71 |
| 3556 | SMIM19 | Small Integral Membrane Protein 19 | 0.71 |
| 3557 | MTARC2 | Mitochondrial Amidoxime Reducing Component 2 | 0.71 |
| 3558 | EP400P1 | EP400 Pseudogene 1 | 0.71 |
| 3559 | NUTM2B-AS1 | NUTM2B Antisense RNA 1 | 0.71 |
| 3560 | LINC02384 | Long Intergenic Non-Protein Coding RNA 2384 | 0.71 |
| 3561 | HSPA2 | Heat Shock Protein Family A (Hsp70) Member 2 | 0.71 |
| 3562 | HNRNPM | Heterogeneous Nuclear Ribonucleoprotein M | 0.71 |
| 3563 | MIR19B1 | MicroRNA 19b-1 | 0.71 |
| 3564 | CKB | Creatine Kinase B | 0.7 |
| 3565 | SMPD3 | Sphingomyelin Phosphodiesterase 3 | 0.7 |
| 3566 | RPS9 | Ribosomal Protein S9 | 0.7 |
| 3567 | RAD50 | RAD50 Double Strand Break Repair Protein | 0.7 |
| 3568 | RPL27A | Ribosomal Protein L27a | 0.7 |
| 3569 | PFDN5 | Prefoldin Subunit 5 | 0.7 |
| 3570 | EIF3K | Eukaryotic Translation Initiation Factor 3 Subunit K | 0.7 |
| 3571 | TRIB3 | Tribbles Pseudokinase 3 | 0.7 |
| 3572 | HTR1F | 5-Hydroxytryptamine Receptor 1F | 0.69 |
| 3573 | SYNM | Synemin | 0.69 |
| 3574 | TNFAIP6 | TNF Alpha Induced Protein 6 | 0.69 |
| 3575 | EEF1G | Eukaryotic Translation Elongation Factor 1 Gamma | 0.69 |
| 3576 | SMOX | Spermine Oxidase | 0.69 |
| 3577 | CNTNAP1 | Contactin Associated Protein 1 | 0.69 |
| 3578 | PRKRA | Protein Activator Of Interferon Induced Protein Kinase EIF2AK2 | 0.69 |
| 3579 | SCN7A | Sodium Voltage-Gated Channel Alpha Subunit 7 | 0.69 |
| 3580 | TNNI3K | TNNI3 Interacting Kinase | 0.69 |
| 3581 | PRRT2 | Proline Rich Transmembrane Protein 2 | 0.69 |
| 3582 | INTS6 | Integrator Complex Subunit 6 | 0.69 |
| 3583 | KIF20B | Kinesin Family Member 20B | 0.69 |
| 3584 | OR1E1 | Olfactory Receptor Family 1 Subfamily E Member 1 | 0.69 |
| 3585 | NPY4R | Neuropeptide Y Receptor Y4 | 0.69 |
| 3586 | DDX10 | DEAD-Box Helicase 10 | 0.69 |
| 3587 | PCOLCE | Procollagen C-Endopeptidase Enhancer | 0.69 |
| 3588 | ANXA6 | Annexin A6 | 0.69 |
| 3589 | HAT1 | Histone Acetyltransferase 1 | 0.68 |
| 3590 | PCSK2 | Proprotein Convertase Subtilisin/Kexin Type 2 | 0.68 |
| 3591 | GZMA | Granzyme A | 0.68 |
| 3592 | ELMO1 | Engulfment And Cell Motility 1 | 0.68 |
| 3593 | PPM1B | Protein Phosphatase, Mg2+/Mn2+ Dependent 1B | 0.68 |
| 3594 | PSMC5 | Proteasome 26S Subunit, ATPase 5 | 0.68 |
| 3595 | UBAP2L | Ubiquitin Associated Protein 2 Like | 0.68 |
| 3596 | SMG1 | SMG1 Nonsense Mediated MRNA Decay Associated PI3K Related Kinase | 0.68 |
| 3597 | ENSG00000282278 |  | 0.67 |
| 3598 | CACNG4 | Calcium Voltage-Gated Channel Auxiliary Subunit Gamma 4 | 0.67 |
| 3599 | RPS25 | Ribosomal Protein S25 | 0.67 |
| 3600 | CENPJ | Centromere Protein J | 0.67 |
| 3601 | SRPX | Sushi Repeat Containing Protein X-Linked | 0.67 |
| 3602 | TFB2M | Transcription Factor B2, Mitochondrial | 0.67 |
| 3603 | BSN | Bassoon Presynaptic Cytomatrix Protein | 0.67 |
| 3604 | FRYL | FRY Like Transcription Coactivator | 0.67 |
| 3605 | RSAD2 | Radical S-Adenosyl Methionine Domain Containing 2 | 0.67 |
| 3606 | PCBP3 | Poly(RC) Binding Protein 3 | 0.67 |
| 3607 | RUVBL2 | RuvB Like AAA ATPase 2 | 0.67 |
| 3608 | HNRNPD | Heterogeneous Nuclear Ribonucleoprotein D | 0.67 |
| 3609 | RIPK4 | Receptor Interacting Serine/Threonine Kinase 4 | 0.67 |
| 3610 | PIK3C2G | Phosphatidylinositol-4-Phosphate 3-Kinase Catalytic Subunit Type 2 Gamma | 0.66 |
| 3611 | BAG6 | BAG Cochaperone 6 | 0.66 |
| 3612 | CSE1L | Chromosome Segregation 1 Like | 0.66 |
| 3613 | SNRPD2 | Small Nuclear Ribonucleoprotein D2 Polypeptide | 0.66 |
| 3614 | PSRC1 | Proline And Serine Rich Coiled-Coil 1 | 0.66 |
| 3615 | FOXK1 | Forkhead Box K1 | 0.66 |
| 3616 | SAA2 | Serum Amyloid A2 | 0.65 |
| 3617 | FABP5 | Fatty Acid Binding Protein 5 | 0.65 |
| 3618 | YARS2 | Tyrosyl-TRNA Synthetase 2 | 0.65 |
| 3619 | PARVB | Parvin Beta | 0.65 |
| 3620 | ITPR2 | Inositol 1,4,5-Trisphosphate Receptor Type 2 | 0.65 |
| 3621 | MIR128-2 | MicroRNA 128-2 | 0.65 |
| 3622 | ABCC5 | ATP Binding Cassette Subfamily C Member 5 | 0.65 |
| 3623 | ACADM | Acyl-CoA Dehydrogenase Medium Chain | 0.65 |
| 3624 | PABPC4 | Poly(A) Binding Protein Cytoplasmic 4 | 0.64 |
| 3625 | RPL3 | Ribosomal Protein L3 | 0.64 |
| 3626 | SLCO3A1 | Solute Carrier Organic Anion Transporter Family Member 3A1 | 0.64 |
| 3627 | HSPA1B | Heat Shock Protein Family A (Hsp70) Member 1B | 0.64 |
| 3628 | CD83 | CD83 Molecule | 0.64 |
| 3629 | AKAP1 | A-Kinase Anchoring Protein 1 | 0.64 |
| 3630 | PRMT1 | Protein Arginine Methyltransferase 1 | 0.63 |
| 3631 | HNRNPAB | Heterogeneous Nuclear Ribonucleoprotein A/B | 0.63 |
| 3632 | CTSE | Cathepsin E | 0.63 |
| 3633 | POLR2C | RNA Polymerase II Subunit C | 0.63 |
| 3634 | TIAL1 | TIA1 Cytotoxic Granule Associated RNA Binding Protein Like 1 | 0.63 |
| 3635 | HSD17B12 | Hydroxysteroid 17-Beta Dehydrogenase 12 | 0.63 |
| 3636 | RAB1A | RAB1A, Member RAS Oncogene Family | 0.62 |
| 3637 | HNRNPR | Heterogeneous Nuclear Ribonucleoprotein R | 0.62 |
| 3638 | PFDN1 | Prefoldin Subunit 1 | 0.62 |
| 3639 | SNRPF | Small Nuclear Ribonucleoprotein Polypeptide F | 0.62 |
| 3640 | CCAR2 | Cell Cycle And Apoptosis Regulator 2 | 0.62 |
| 3641 | GCN1 | GCN1 Activator Of EIF2AK4 | 0.62 |
| 3642 | PAPPA2 | Pappalysin 2 | 0.62 |
| 3643 | GATD3A | Glutamine Amidotransferase Like Class 1 Domain Containing 3A | 0.62 |
| 3644 | CACNA1B | Calcium Voltage-Gated Channel Subunit Alpha1 B | 0.62 |
| 3645 | FHL1 | Four And A Half LIM Domains 1 | 0.62 |
| 3646 | GPBAR1 | G Protein-Coupled Bile Acid Receptor 1 | 0.62 |
| 3647 | CCL15 | C-C Motif Chemokine Ligand 15 | 0.62 |
| 3648 | GRINA | Glutamate Ionotropic Receptor NMDA Type Subunit Associated Protein 1 | 0.62 |
| 3649 | TRMT11 | TRNA Methyltransferase 11 Homolog | 0.62 |
| 3650 | NEDD1 | NEDD1 Gamma-Tubulin Ring Complex Targeting Factor | 0.62 |
| 3651 | TCF12 | Transcription Factor 12 | 0.62 |
| 3652 | HEPACAM | Hepatic And Glial Cell Adhesion Molecule | 0.62 |
| 3653 | PHB2 | Prohibitin 2 | 0.62 |
| 3654 | CD207 | CD207 Molecule | 0.62 |
| 3655 | NECTIN1 | Nectin Cell Adhesion Molecule 1 | 0.62 |
| 3656 | NCAPG2 | Non-SMC Condensin II Complex Subunit G2 | 0.61 |
| 3657 | PDE4C | Phosphodiesterase 4C | 0.61 |
| 3658 | POLR2E | RNA Polymerase II, I And III Subunit E | 0.61 |
| 3659 | EFHD2 | EF-Hand Domain Family Member D2 | 0.61 |
| 3660 | ABCF1 | ATP Binding Cassette Subfamily F Member 1 | 0.61 |
| 3661 | IPO9 | Importin 9 | 0.61 |
| 3662 | FLOT1 | Flotillin 1 | 0.61 |
| 3663 | RNF126 | Ring Finger Protein 126 | 0.61 |
| 3664 | ATG101 | Autophagy Related 101 | 0.61 |
| 3665 | ESD | Esterase D | 0.61 |
| 3666 | SCN1B | Sodium Voltage-Gated Channel Beta Subunit 1 | 0.61 |
| 3667 | PNPLA3 | Patatin Like Phospholipase Domain Containing 3 | 0.61 |
| 3668 | ATAD3A | ATPase Family AAA Domain Containing 3A | 0.61 |
| 3669 | KIF13B | Kinesin Family Member 13B | 0.61 |
| 3670 | ZFYVE21 | Zinc Finger FYVE-Type Containing 21 | 0.61 |
| 3671 | SLCO1C1 | Solute Carrier Organic Anion Transporter Family Member 1C1 | 0.6 |
| 3672 | HRC | Histidine Rich Calcium Binding Protein | 0.6 |
| 3673 | LILRB2 | Leukocyte Immunoglobulin Like Receptor B2 | 0.6 |
| 3674 | DELEC1 | Deleted In Esophageal Cancer 1 | 0.6 |
| 3675 | KPNA2 | Karyopherin Subunit Alpha 2 | 0.6 |
| 3676 | RFC4 | Replication Factor C Subunit 4 | 0.6 |
| 3677 | NXF1 | Nuclear RNA Export Factor 1 | 0.6 |
| 3678 | GFRA2 | GDNF Family Receptor Alpha 2 | 0.59 |
| 3679 | CCT5 | Chaperonin Containing TCP1 Subunit 5 | 0.59 |
| 3680 | CCT2 | Chaperonin Containing TCP1 Subunit 2 | 0.59 |
| 3681 | CCT4 | Chaperonin Containing TCP1 Subunit 4 | 0.59 |
| 3682 | TUBB4B | Tubulin Beta 4B Class IVb | 0.59 |
| 3683 | TUBB1 | Tubulin Beta 1 Class VI | 0.59 |
| 3684 | SF3A1 | Splicing Factor 3a Subunit 1 | 0.59 |
| 3685 | GBF1 | Golgi Brefeldin A Resistant Guanine Nucleotide Exchange Factor 1 | 0.59 |
| 3686 | OSCP1 | Organic Solute Carrier Partner 1 | 0.59 |
| 3687 | CTDP1 | CTD Phosphatase Subunit 1 | 0.58 |
| 3688 | SRP14 | Signal Recognition Particle 14 | 0.58 |
| 3689 | SEC61B | SEC61 Translocon Subunit Beta | 0.58 |
| 3690 | PRKACB | Protein Kinase CAMP-Activated Catalytic Subunit Beta | 0.58 |
| 3691 | ACER2 | Alkaline Ceramidase 2 | 0.58 |
| 3692 | SPATA6L | Spermatogenesis Associated 6 Like | 0.58 |
| 3693 | TGM3 | Transglutaminase 3 | 0.58 |
| 3694 | KCND2 | Potassium Voltage-Gated Channel Subfamily D Member 2 | 0.58 |
| 3695 | SLC4A3 | Solute Carrier Family 4 Member 3 | 0.57 |
| 3696 | H2BC5 | H2B Clustered Histone 5 | 0.57 |
| 3697 | MTUS1 | Microtubule Associated Scaffold Protein 1 | 0.57 |
| 3698 | MLYCD | Malonyl-CoA Decarboxylase | 0.57 |
| 3699 | RANBP9 | RAN Binding Protein 9 | 0.57 |
| 3700 | TOM1L2 | Target Of Myb1 Like 2 Membrane Trafficking Protein | 0.57 |
| 3701 | BRI3 | Brain Protein I3 | 0.57 |
| 3702 | LRPPRC | Leucine Rich Pentatricopeptide Repeat Containing | 0.57 |
| 3703 | SERBP1 | SERPINE1 MRNA Binding Protein 1 | 0.56 |
| 3704 | RCAN3 | RCAN Family Member 3 | 0.56 |
| 3705 | ZNF175 | Zinc Finger Protein 175 | 0.56 |
| 3706 | RPS8 | Ribosomal Protein S8 | 0.56 |
| 3707 | SESN2 | Sestrin 2 | 0.56 |
| 3708 | MAT2B | Methionine Adenosyltransferase 2B | 0.56 |
| 3709 | TANK | TRAF Family Member Associated NFKB Activator | 0.56 |
| 3710 | CDC37 | Cell Division Cycle 37, HSP90 Cochaperone | 0.56 |
| 3711 | CARM1 | Coactivator Associated Arginine Methyltransferase 1 | 0.56 |
| 3712 | SH3GL1 | SH3 Domain Containing GRB2 Like 1, Endophilin A2 | 0.56 |
| 3713 | LHCGR | Luteinizing Hormone/Choriogonadotropin Receptor | 0.56 |
| 3714 | LIMA1 | LIM Domain And Actin Binding 1 | 0.56 |
| 3715 | SLC16A2 | Solute Carrier Family 16 Member 2 | 0.55 |
| 3716 | IDI1 | Isopentenyl-Diphosphate Delta Isomerase 1 | 0.55 |
| 3717 | CPNE1 | Copine 1 | 0.55 |
| 3718 | SEC24A | SEC24 Homolog A, COPII Coat Complex Component | 0.55 |
| 3719 | SIM2 | SIM BHLH Transcription Factor 2 | 0.55 |
| 3720 | ACOT13 | Acyl-CoA Thioesterase 13 | 0.55 |
| 3721 | HTR3E | 5-Hydroxytryptamine Receptor 3E | 0.55 |
| 3722 | MTUS2 | Microtubule Associated Scaffold Protein 2 | 0.55 |
| 3723 | MIEF1 | Mitochondrial Elongation Factor 1 | 0.55 |
| 3724 | RIPOR2 | RHO Family Interacting Cell Polarization Regulator 2 | 0.55 |
| 3725 | NRON | Non-Coding Repressor Of NFAT | 0.55 |
| 3726 | MIR802 | MicroRNA 802 | 0.55 |
| 3727 | AP2A1 | Adaptor Related Protein Complex 2 Subunit Alpha 1 | 0.55 |
| 3728 | PCSK1 | Proprotein Convertase Subtilisin/Kexin Type 1 | 0.55 |
| 3729 | VAMP1 | Vesicle Associated Membrane Protein 1 | 0.55 |
| 3730 | SPTB | Spectrin Beta, Erythrocytic | 0.55 |
| 3731 | CPM | Carboxypeptidase M | 0.55 |
| 3732 | MIR34C | MicroRNA 34c | 0.55 |
| 3733 | SNF8 | SNF8 Subunit Of ESCRT-II | 0.55 |
| 3734 | DDX39A | DExD-Box Helicase 39A | 0.55 |
| 3735 | METTL23 | Methyltransferase Like 23 | 0.55 |
| 3736 | MED1 | Mediator Complex Subunit 1 | 0.55 |
| 3737 | CLK1 | CDC Like Kinase 1 | 0.55 |
| 3738 | HNRNPF | Heterogeneous Nuclear Ribonucleoprotein F | 0.55 |
| 3739 | CCL25 | C-C Motif Chemokine Ligand 25 | 0.55 |
| 3740 | ENSG00000253392 |  | 0.54 |
| 3741 | POLR2G | RNA Polymerase II Subunit G | 0.54 |
| 3742 | IQGAP2 | IQ Motif Containing GTPase Activating Protein 2 | 0.54 |
| 3743 | ACTN2 | Actinin Alpha 2 | 0.54 |
| 3744 | HMGCS1 | 3-Hydroxy-3-Methylglutaryl-CoA Synthase 1 | 0.54 |
| 3745 | PARVG | Parvin Gamma | 0.54 |
| 3746 | PREX1 | Phosphatidylinositol-3,4,5-Trisphosphate Dependent Rac Exchange Factor 1 | 0.54 |
| 3747 | FRK | Fyn Related Src Family Tyrosine Kinase | 0.54 |
| 3748 | NUDC | Nuclear Distribution C, Dynein Complex Regulator | 0.53 |
| 3749 | ACTN3 | Actinin Alpha 3 | 0.53 |
| 3750 | HCRTR1 | Hypocretin Receptor 1 | 0.53 |
| 3751 | PDHA1 | Pyruvate Dehydrogenase E1 Subunit Alpha 1 | 0.53 |
| 3752 | EIF4A1 | Eukaryotic Translation Initiation Factor 4A1 | 0.53 |
| 3753 | NPTXR | Neuronal Pentraxin Receptor | 0.52 |
| 3754 | SLC25A5 | Solute Carrier Family 25 Member 5 | 0.52 |
| 3755 | CKM | Creatine Kinase, M-Type | 0.52 |
| 3756 | HLA-F | Major Histocompatibility Complex, Class I, F | 0.52 |
| 3757 | H2AZ2 | H2A.Z Variant Histone 2 | 0.52 |
| 3758 | INSIG1 | Insulin Induced Gene 1 | 0.52 |
| 3759 | TRAF3IP1 | TRAF3 Interacting Protein 1 | 0.52 |
| 3760 | THRA | Thyroid Hormone Receptor Alpha | 0.52 |
| 3761 | RARG | Retinoic Acid Receptor Gamma | 0.52 |
| 3762 | HSPA1L | Heat Shock Protein Family A (Hsp70) Member 1 Like | 0.52 |
| 3763 | SRP9 | Signal Recognition Particle 9 | 0.52 |
| 3764 | WWP2 | WW Domain Containing E3 Ubiquitin Protein Ligase 2 | 0.51 |
| 3765 | TTN | Titin | 0.51 |
| 3766 | TM9SF2 | Transmembrane 9 Superfamily Member 2 | 0.51 |
| 3767 | LEF1 | Lymphoid Enhancer Binding Factor 1 | 0.51 |
| 3768 | RTRAF | RNA Transcription, Translation And Transport Factor | 0.51 |
| 3769 | TUFM | Tu Translation Elongation Factor, Mitochondrial | 0.51 |
| 3770 | PRKAR2B | Protein Kinase CAMP-Dependent Type II Regulatory Subunit Beta | 0.51 |
| 3771 | PSMD2 | Proteasome 26S Subunit, Non-ATPase 2 | 0.51 |
| 3772 | PFDN4 | Prefoldin Subunit 4 | 0.51 |
| 3773 | PDCD5 | Programmed Cell Death 5 | 0.51 |
| 3774 | METTL21A | Methyltransferase Like 21A | 0.51 |
| 3775 | EIF3E | Eukaryotic Translation Initiation Factor 3 Subunit E | 0.5 |
| 3776 | SERPINB12 | Serpin Family B Member 12 | 0.5 |
| 3777 | NEGR1 | Neuronal Growth Regulator 1 | 0.5 |
| 3778 | NCKAP1 | NCK Associated Protein 1 | 0.5 |
| 3779 | DECR1 | 2,4-Dienoyl-CoA Reductase 1 | 0.49 |
| 3780 | ATAD2 | ATPase Family AAA Domain Containing 2 | 0.49 |
| 3781 | RPL12 | Ribosomal Protein L12 | 0.49 |
| 3782 | MT1A | Metallothionein 1A | 0.49 |
| 3783 | NUDT21 | Nudix Hydrolase 21 | 0.49 |
| 3784 | EIF2B2 | Eukaryotic Translation Initiation Factor 2B Subunit Beta | 0.49 |
| 3785 | MAP3K4 | Mitogen-Activated Protein Kinase Kinase Kinase 4 | 0.49 |
| 3786 | RHOU | Ras Homolog Family Member U | 0.49 |
| 3787 | AP2M1 | Adaptor Related Protein Complex 2 Subunit Mu 1 | 0.49 |
| 3788 | TSBP1 | Testis Expressed Basic Protein 1 | 0.48 |
| 3789 | SEPTIN9 | Septin 9 | 0.48 |
| 3790 | TRAF5 | TNF Receptor Associated Factor 5 | 0.47 |
| 3791 | ANKRD2 | Ankyrin Repeat Domain 2 | 0.47 |
| 3792 | HMGB2 | High Mobility Group Box 2 | 0.47 |
| 3793 | RBBP4 | RB Binding Protein 4, Chromatin Remodeling Factor | 0.46 |
| 3794 | TRIM65 | Tripartite Motif Containing 65 | 0.46 |
| 3795 | MCM5 | Minichromosome Maintenance Complex Component 5 | 0.46 |
| 3796 | NCALD | Neurocalcin Delta | 0.46 |
| 3797 | NPY2R | Neuropeptide Y Receptor Y2 | 0.46 |
| 3798 | BET1 | Bet1 Golgi Vesicular Membrane Trafficking Protein | 0.46 |
| 3799 | LAT | Linker For Activation Of T Cells | 0.46 |
| 3800 | LDHD | Lactate Dehydrogenase D | 0.46 |
| 3801 | ARNT2 | Aryl Hydrocarbon Receptor Nuclear Translocator 2 | 0.46 |
| 3802 | ADAM28 | ADAM Metallopeptidase Domain 28 | 0.46 |
| 3803 | COPG2 | COPI Coat Complex Subunit Gamma 2 | 0.46 |
| 3804 | CNTN5 | Contactin 5 | 0.46 |
| 3805 | MS | Multiple Sclerosis | 0.45 |
| 3806 | NAXE | NAD(P)HX Epimerase | 0.45 |
| 3807 | RCL1 | RNA Terminal Phosphate Cyclase Like 1 | 0.45 |
| 3808 | DMRT2 | Doublesex And Mab-3 Related Transcription Factor 2 | 0.45 |
| 3809 | PDZRN4 | PDZ Domain Containing Ring Finger 4 | 0.45 |
| 3810 | SRSF3 | Serine And Arginine Rich Splicing Factor 3 | 0.44 |
| 3811 | NTM | Neurotrimin | 0.44 |
| 3812 | DHX40 | DEAH-Box Helicase 40 | 0.44 |
| 3813 | EIF4G2 | Eukaryotic Translation Initiation Factor 4 Gamma 2 | 0.44 |
| 3814 | RASGRF1 | Ras Protein Specific Guanine Nucleotide Releasing Factor 1 | 0.44 |
| 3815 | DNAJB11 | DnaJ Heat Shock Protein Family (Hsp40) Member B11 | 0.44 |
| 3816 | ESYT1 | Extended Synaptotagmin 1 | 0.44 |
| 3817 | NUP205 | Nucleoporin 205 | 0.44 |
| 3818 | FAM162A | Family With Sequence Similarity 162 Member A | 0.44 |
| 3819 | ACACB | Acetyl-CoA Carboxylase Beta | 0.44 |
| 3820 | EHF | ETS Homologous Factor | 0.44 |
| 3821 | POMK | Protein O-Mannose Kinase | 0.44 |
| 3822 | TSPAN14 | Tetraspanin 14 | 0.44 |
| 3823 | SFRP4 | Secreted Frizzled Related Protein 4 | 0.44 |
| 3824 | DST | Dystonin | 0.44 |
| 3825 | SERPINE3 | Serpin Family E Member 3 | 0.44 |
| 3826 | NPAS2 | Neuronal PAS Domain Protein 2 | 0.44 |
| 3827 | RPL4 | Ribosomal Protein L4 | 0.44 |
| 3828 | HECTD2 | HECT Domain E3 Ubiquitin Protein Ligase 2 | 0.44 |
| 3829 | ATG13 | Autophagy Related 13 | 0.43 |
| 3830 | IFNGR1 | Interferon Gamma Receptor 1 | 0.43 |
| 3831 | EDF1 | Endothelial Differentiation Related Factor 1 | 0.43 |
| 3832 | ZNF423 | Zinc Finger Protein 423 | 0.43 |
| 3833 | PLCD3 | Phospholipase C Delta 3 | 0.43 |
| 3834 | MACF1 | Microtubule Actin Crosslinking Factor 1 | 0.42 |
| 3835 | REEP3 | Receptor Accessory Protein 3 | 0.42 |
| 3836 | MIR1296 | MicroRNA 1296 | 0.42 |
| 3837 | PSD3 | Pleckstrin And Sec7 Domain Containing 3 | 0.42 |
| 3838 | SAE1 | SUMO1 Activating Enzyme Subunit 1 | 0.42 |
| 3839 | POLR2F | RNA Polymerase II, I And III Subunit F | 0.42 |
| 3840 | GTF2F1 | General Transcription Factor IIF Subunit 1 | 0.42 |
| 3841 | LSM14A | LSM14A MRNA Processing Body Assembly Factor | 0.42 |
| 3842 | C6 | Complement C6 | 0.42 |
| 3843 | SERPINA2 | Serpin Family A Member 2 (Gene/Pseudogene) | 0.42 |
| 3844 | AKR7A2 | Aldo-Keto Reductase Family 7 Member A2 | 0.42 |
| 3845 | PPP4C | Protein Phosphatase 4 Catalytic Subunit | 0.42 |
| 3846 | RRBP1 | Ribosome Binding Protein 1 | 0.42 |
| 3847 | BCL7C | BAF Chromatin Remodeling Complex Subunit BCL7C | 0.42 |
| 3848 | CALCR | Calcitonin Receptor | 0.41 |
| 3849 | PDIA3 | Protein Disulfide Isomerase Family A Member 3 | 0.41 |
| 3850 | SLC24A4 | Solute Carrier Family 24 Member 4 | 0.41 |
| 3851 | MMP19 | Matrix Metallopeptidase 19 | 0.41 |
| 3852 | ESRRG | Estrogen Related Receptor Gamma | 0.41 |
| 3853 | PRKAG1 | Protein Kinase AMP-Activated Non-Catalytic Subunit Gamma 1 | 0.4 |
| 3854 | TARBP2 | TARBP2 Subunit Of RISC Loading Complex | 0.4 |
| 3855 | RLF | RLF Zinc Finger | 0.4 |
| 3856 | ZNF180 | Zinc Finger Protein 180 | 0.4 |
| 3857 | TXNL4B | Thioredoxin Like 4B | 0.4 |
| 3858 | UACA | Uveal Autoantigen With Coiled-Coil Domains And Ankyrin Repeats | 0.4 |
| 3859 | WDR83OS | WD Repeat Domain 83 Opposite Strand | 0.4 |
| 3860 | SUPT4H1 | SPT4 Homolog, DSIF Elongation Factor Subunit | 0.4 |
| 3861 | IPO7 | Importin 7 | 0.4 |
| 3862 | TMX1 | Thioredoxin Related Transmembrane Protein 1 | 0.4 |
| 3863 | GTF3C4 | General Transcription Factor IIIC Subunit 4 | 0.4 |
| 3864 | PRRC2B | Proline Rich Coiled-Coil 2B | 0.4 |
| 3865 | LINC00520 | Long Intergenic Non-Protein Coding RNA 520 | 0.4 |
| 3866 | HIPK1 | Homeodomain Interacting Protein Kinase 1 | 0.4 |
| 3867 | CPA6 | Carboxypeptidase A6 | 0.4 |
| 3868 | TRAF4 | TNF Receptor Associated Factor 4 | 0.4 |
| 3869 | PLXNA4 | Plexin A4 | 0.4 |
| 3870 | PRSS2 | Serine Protease 2 | 0.4 |
| 3871 | BAG3 | BAG Cochaperone 3 | 0.4 |
| 3872 | DHODH | Dihydroorotate Dehydrogenase (Quinone) | 0.4 |
| 3873 | EIF2B5 | Eukaryotic Translation Initiation Factor 2B Subunit Epsilon | 0.4 |
| 3874 | PFKFB1 | 6-Phosphofructo-2-Kinase/Fructose-2,6-Biphosphatase 1 | 0.4 |
| 3875 | PCYT1B | Phosphate Cytidylyltransferase 1, Choline, Beta | 0.4 |
| 3876 | DNAJA2 | DnaJ Heat Shock Protein Family (Hsp40) Member A2 | 0.4 |
| 3877 | RXRG | Retinoid X Receptor Gamma | 0.39 |
| 3878 | APCDD1 | APC Down-Regulated 1 | 0.39 |
| 3879 | KRT18P42 | Keratin 18 Pseudogene 42 | 0.39 |
| 3880 | GNG4 | G Protein Subunit Gamma 4 | 0.39 |
| 3881 | CPSF6 | Cleavage And Polyadenylation Specific Factor 6 | 0.39 |
| 3882 | TRIM47 | Tripartite Motif Containing 47 | 0.39 |
| 3883 | FAM98A | Family With Sequence Similarity 98 Member A | 0.39 |
| 3884 | RB1CC1 | RB1 Inducible Coiled-Coil 1 | 0.39 |
| 3885 | UBAP2 | Ubiquitin Associated Protein 2 | 0.38 |
| 3886 | CPT1A | Carnitine Palmitoyltransferase 1A | 0.38 |
| 3887 | ASPSCR1 | ASPSCR1 Tether For SLC2A4, UBX Domain Containing | 0.38 |
| 3888 | BST2 | Bone Marrow Stromal Cell Antigen 2 | 0.38 |
| 3889 | HRK | Harakiri, BCL2 Interacting Protein | 0.38 |
| 3890 | COPB1 | COPI Coat Complex Subunit Beta 1 | 0.38 |
| 3891 | UGGT1 | UDP-Glucose Glycoprotein Glucosyltransferase 1 | 0.38 |
| 3892 | RPN1 | Ribophorin I | 0.38 |
| 3893 | DNAJB9 | DnaJ Heat Shock Protein Family (Hsp40) Member B9 | 0.37 |
| 3894 | SLC1A5 | Solute Carrier Family 1 Member 5 | 0.37 |
| 3895 | CDK15 | Cyclin Dependent Kinase 15 | 0.37 |
| 3896 | RBMS1 | RNA Binding Motif Single Stranded Interacting Protein 1 | 0.37 |
| 3897 | CTAGE1 | Cutaneous T Cell Lymphoma-Associated Antigen 1 | 0.37 |
| 3898 | ST8SIA6 | ST8 Alpha-N-Acetyl-Neuraminide Alpha-2,8-Sialyltransferase 6 | 0.37 |
| 3899 | PLCD4 | Phospholipase C Delta 4 | 0.37 |
| 3900 | NCEH1 | Neutral Cholesterol Ester Hydrolase 1 | 0.37 |
| 3901 | DUT | Deoxyuridine Triphosphatase | 0.37 |
| 3902 | E2F7 | E2F Transcription Factor 7 | 0.36 |
| 3903 | SAR1A | Secretion Associated Ras Related GTPase 1A | 0.36 |
| 3904 | DSC1 | Desmocollin 1 | 0.36 |
| 3905 | SPINT1 | Serine Peptidase Inhibitor, Kunitz Type 1 | 0.36 |
| 3906 | PAFAH1B3 | Platelet Activating Factor Acetylhydrolase 1b Catalytic Subunit 3 | 0.36 |
| 3907 | NCOA1 | Nuclear Receptor Coactivator 1 | 0.36 |
| 3908 | TIGAR | TP53 Induced Glycolysis Regulatory Phosphatase | 0.36 |
| 3909 | TPPP2 | Tubulin Polymerization Promoting Protein Family Member 2 | 0.36 |
| 3910 | SLC25A20 | Solute Carrier Family 25 Member 20 | 0.36 |
| 3911 | PDE8A | Phosphodiesterase 8A | 0.35 |
| 3912 | CPSF1 | Cleavage And Polyadenylation Specific Factor 1 | 0.35 |
| 3913 | HM13 | Histocompatibility Minor 13 | 0.35 |
| 3914 | ADCK5 | AarF Domain Containing Kinase 5 | 0.35 |
| 3915 | NCAPH2 | Non-SMC Condensin II Complex Subunit H2 | 0.35 |
| 3916 | ASCC2 | Activating Signal Cointegrator 1 Complex Subunit 2 | 0.35 |
| 3917 | RASGEF1A | RasGEF Domain Family Member 1A | 0.35 |
| 3918 | PSD2 | Pleckstrin And Sec7 Domain Containing 2 | 0.35 |
| 3919 | LRRC14 | Leucine Rich Repeat Containing 14 | 0.35 |
| 3920 | SYT16 | Synaptotagmin 16 | 0.35 |
| 3921 | TMEM50B | Transmembrane Protein 50B | 0.35 |
| 3922 | MAP1LC3B | Microtubule Associated Protein 1 Light Chain 3 Beta | 0.35 |
| 3923 | SYPL1 | Synaptophysin Like 1 | 0.35 |
| 3924 | SGCZ | Sarcoglycan Zeta | 0.35 |
| 3925 | VTI1B | Vesicle Transport Through Interaction With T-SNAREs 1B | 0.35 |
| 3926 | ROBO2 | Roundabout Guidance Receptor 2 | 0.35 |
| 3927 | STK16 | Serine/Threonine Kinase 16 | 0.35 |
| 3928 | PPP4R3A | Protein Phosphatase 4 Regulatory Subunit 3A | 0.35 |
| 3929 | CCNT1 | Cyclin T1 | 0.34 |
| 3930 | EIF3G | Eukaryotic Translation Initiation Factor 3 Subunit G | 0.34 |
| 3931 | MPP2 | Membrane Palmitoylated Protein 2 | 0.34 |
| 3932 | PPP2R5B | Protein Phosphatase 2 Regulatory Subunit B'Beta | 0.34 |
| 3933 | POLR2M | RNA Polymerase II Subunit M | 0.34 |
| 3934 | PDHB | Pyruvate Dehydrogenase E1 Subunit Beta | 0.34 |
| 3935 | EIF3F | Eukaryotic Translation Initiation Factor 3 Subunit F | 0.34 |
| 3936 | PLIN3 | Perilipin 3 | 0.34 |
| 3937 | PSMD11 | Proteasome 26S Subunit, Non-ATPase 11 | 0.34 |
| 3938 | DHX9 | DExH-Box Helicase 9 | 0.34 |
| 3939 | SRP72 | Signal Recognition Particle 72 | 0.34 |
| 3940 | IQGAP3 | IQ Motif Containing GTPase Activating Protein 3 | 0.34 |
| 3941 | PRMT6 | Protein Arginine Methyltransferase 6 | 0.34 |
| 3942 | PSMD13 | Proteasome 26S Subunit, Non-ATPase 13 | 0.34 |
| 3943 | NACA | Nascent Polypeptide Associated Complex Subunit Alpha | 0.34 |
| 3944 | PSMD6 | Proteasome 26S Subunit, Non-ATPase 6 | 0.34 |
| 3945 | ESYT2 | Extended Synaptotagmin 2 | 0.34 |
| 3946 | APOO | Apolipoprotein O | 0.34 |
| 3947 | HNRNPUL2 | Heterogeneous Nuclear Ribonucleoprotein U Like 2 | 0.34 |
| 3948 | EXOC4 | Exocyst Complex Component 4 | 0.34 |
| 3949 | RSPH4A | Radial Spoke Head Component 4A | 0.34 |
| 3950 | OCA2 | OCA2 Melanosomal Transmembrane Protein | 0.34 |
| 3951 | TNFAIP1 | TNF Alpha Induced Protein 1 | 0.34 |
| 3952 | NKX2-8 | NK2 Homeobox 8 | 0.33 |
| 3953 | PLK2 | Polo Like Kinase 2 | 0.33 |
| 3954 | NPAS4 | Neuronal PAS Domain Protein 4 | 0.33 |
| 3955 | CAND1 | Cullin Associated And Neddylation Dissociated 1 | 0.33 |
| 3956 | ADCYAP1R1 | ADCYAP Receptor Type I | 0.33 |
| 3957 | GNB4 | G Protein Subunit Beta 4 | 0.33 |
| 3958 | METTL1 | Methyltransferase Like 1 | 0.33 |
| 3959 | PSMC3 | Proteasome 26S Subunit, ATPase 3 | 0.33 |
| 3960 | CCT6B | Chaperonin Containing TCP1 Subunit 6B | 0.33 |
| 3961 | RAB8B | RAB8B, Member RAS Oncogene Family | 0.33 |
| 3962 | EXOSC1 | Exosome Component 1 | 0.33 |
| 3963 | SRP68 | Signal Recognition Particle 68 | 0.33 |
| 3964 | TUBGCP2 | Tubulin Gamma Complex Associated Protein 2 | 0.33 |
| 3965 | GSDMA | Gasdermin A | 0.33 |
| 3966 | MMS19 | MMS19 Homolog, Cytosolic Iron-Sulfur Assembly Component | 0.33 |
| 3967 | TMX4 | Thioredoxin Related Transmembrane Protein 4 | 0.33 |
| 3968 | RAB33A | RAB33A, Member RAS Oncogene Family | 0.33 |
| 3969 | THOC3 | THO Complex 3 | 0.33 |
| 3970 | RAB12 | RAB12, Member RAS Oncogene Family | 0.33 |
| 3971 | GRWD1 | Glutamate Rich WD Repeat Containing 1 | 0.33 |
| 3972 | CCDC7 | Coiled-Coil Domain Containing 7 | 0.33 |
| 3973 | FAM86C1P | Family With Sequence Similarity 86 Member C1, Pseudogene | 0.33 |
| 3974 | EEF1AKMT3 | EEF1A Lysine Methyltransferase 3 | 0.33 |
| 3975 | CDK5RAP3 | CDK5 Regulatory Subunit Associated Protein 3 | 0.32 |
| 3976 | MAB21L1 | Mab-21 Like 1 | 0.32 |
| 3977 | CFAP300 | Cilia And Flagella Associated Protein 300 | 0.32 |
| 3978 | COBL | Cordon-Bleu WH2 Repeat Protein | 0.32 |
| 3979 | SLC2A8 | Solute Carrier Family 2 Member 8 | 0.31 |
| 3980 | HNRNPUL1 | Heterogeneous Nuclear Ribonucleoprotein U Like 1 | 0.31 |
| 3981 | TENM4 | Teneurin Transmembrane Protein 4 | 0.31 |
| 3982 | C9orf78 | Chromosome 9 Open Reading Frame 78 | 0.31 |
| 3983 | WDR70 | WD Repeat Domain 70 | 0.31 |
| 3984 | CCDC124 | Coiled-Coil Domain Containing 124 | 0.31 |
| 3985 | PRKD3 | Protein Kinase D3 | 0.31 |
| 3986 | UCP3 | Uncoupling Protein 3 | 0.31 |
| 3987 | PARD6A | Par-6 Family Cell Polarity Regulator Alpha | 0.31 |
| 3988 | TNKS2 | Tankyrase 2 | 0.31 |
| 3989 | CELF2 | CUGBP Elav-Like Family Member 2 | 0.31 |
| 3990 | RHD | Rh Blood Group D Antigen | 0.31 |
| 3991 | NAP1L1 | Nucleosome Assembly Protein 1 Like 1 | 0.31 |
| 3992 | MSI1 | Musashi RNA Binding Protein 1 | 0.31 |
| 3993 | ZFAT | Zinc Finger And AT-Hook Domain Containing | 0.31 |
| 3994 | RTCB | RNA 2',3'-Cyclic Phosphate And 5'-OH Ligase | 0.31 |
| 3995 | RAVER1 | Ribonucleoprotein, PTB Binding 1 | 0.31 |
| 3996 | GPRC6A | G Protein-Coupled Receptor Class C Group 6 Member A | 0.31 |
| 3997 | GZMK | Granzyme K | 0.31 |
| 3998 | AKAP17A | A-Kinase Anchoring Protein 17A | 0.31 |
| 3999 | SLC25A5P2 | Solute Carrier Family 25 Member 5 Pseudogene 2 | 0.31 |
| 4000 | PGRMC1 | Progesterone Receptor Membrane Component 1 | 0.3 |
| 4001 | PDE7A | Phosphodiesterase 7A | 0.3 |
| 4002 | C4BPB | Complement Component 4 Binding Protein Beta | 0.3 |
| 4003 | H3-4 | H3.4 Histone | 0.3 |
| 4004 | MDM4 | MDM4 Regulator Of P53 | 0.3 |
| 4005 | MT4 | Metallothionein 4 | 0.3 |
| 4006 | JRKL | JRK Like | 0.3 |
| 4007 | FLJ22447 | Uncharacterized LOC400221 | 0.3 |
| 4008 | LOC100128993 | Uncharacterized LOC100128993 | 0.3 |
| 4009 | CDK8 | Cyclin Dependent Kinase 8 | 0.29 |
| 4010 | MAPRE1 | Microtubule Associated Protein RP/EB Family Member 1 | 0.29 |
| 4011 | MARK2 | Microtubule Affinity Regulating Kinase 2 | 0.29 |
| 4012 | PA2G4 | Proliferation-Associated 2G4 | 0.29 |
| 4013 | KCNIP4 | Potassium Voltage-Gated Channel Interacting Protein 4 | 0.29 |
| 4014 | PPP1R9A | Protein Phosphatase 1 Regulatory Subunit 9A | 0.29 |
| 4015 | CYTIP | Cytohesin 1 Interacting Protein | 0.29 |
| 4016 | THSD7B | Thrombospondin Type 1 Domain Containing 7B | 0.29 |
| 4017 | CYLC2 | Cylicin 2 | 0.29 |
| 4018 | ANKRD7 | Ankyrin Repeat Domain 7 | 0.29 |
| 4019 | CYYR1 | Cysteine And Tyrosine Rich 1 | 0.29 |
| 4020 | UBBP1 | Ubiquitin B Pseudogene 1 | 0.29 |
| 4021 | RPL12P34 | Ribosomal Protein L12 Pseudogene 34 | 0.29 |
| 4022 | RPL18P1 | Ribosomal Protein L18 Pseudogene 1 | 0.29 |
| 4023 | RPL22P22 | Ribosomal Protein L22 Pseudogene 22 | 0.29 |
| 4024 | RPS2P38 | Ribosomal Protein S2 Pseudogene 38 | 0.29 |
| 4025 | RPL21P99 | Ribosomal Protein L21 Pseudogene 99 | 0.29 |
| 4026 | RPSAP43 | Ribosomal Protein SA Pseudogene 43 | 0.29 |
| 4027 | UBQLN4P2 | Ubiquilin 4 Pseudogene 2 | 0.29 |
| 4028 | LCN1 | Lipocalin 1 | 0.28 |
| 4029 | MIR193B | MicroRNA 193b | 0.28 |
| 4030 | PDCD7 | Programmed Cell Death 7 | 0.28 |
| 4031 | STAMBP | STAM Binding Protein | 0.28 |
| 4032 | PDE1B | Phosphodiesterase 1B | 0.28 |
| 4033 | RPL9 | Ribosomal Protein L9 | 0.28 |
| 4034 | SERPINB13 | Serpin Family B Member 13 | 0.28 |
| 4035 | MYBPC1 | Myosin Binding Protein C1 | 0.28 |
| 4036 | WWC1 | WW And C2 Domain Containing 1 | 0.28 |
| 4037 | PGD | Phosphogluconate Dehydrogenase | 0.27 |
| 4038 | KCNN2 | Potassium Calcium-Activated Channel Subfamily N Member 2 | 0.27 |
| 4039 | PDAP1 | PDGFA Associated Protein 1 | 0.27 |
| 4040 | SRD5A2 | Steroid 5 Alpha-Reductase 2 | 0.27 |
| 4041 | ABCF2 | ATP Binding Cassette Subfamily F Member 2 | 0.26 |
| 4042 | SH3BGRL2 | SH3 Domain Binding Glutamate Rich Protein Like 2 | 0.26 |
| 4043 | RPL15 | Ribosomal Protein L15 | 0.26 |
| 4044 | CRBN | Cereblon | 0.26 |
| 4045 | PAPSS1 | 3'-Phosphoadenosine 5'-Phosphosulfate Synthase 1 | 0.26 |
| 4046 | BMP3 | Bone Morphogenetic Protein 3 | 0.26 |
| 4047 | OPCML | Opioid Binding Protein/Cell Adhesion Molecule Like | 0.26 |
| 4048 | SPOCK3 | SPARC (Osteonectin), Cwcv And Kazal Like Domains Proteoglycan 3 | 0.26 |
| 4049 | RPL24P9 | RPL24 Pseudogene 9 | 0.26 |
| 4050 | UBE2E2 | Ubiquitin Conjugating Enzyme E2 E2 | 0.25 |
| 4051 | PILRA | Paired Immunoglobin Like Type 2 Receptor Alpha | 0.25 |
| 4052 | IPMK | Inositol Polyphosphate Multikinase | 0.25 |
| 4053 | ATP6V1H | ATPase H+ Transporting V1 Subunit H | 0.24 |
| 4054 | CDYL | Chromodomain Y Like | 0.24 |
| 4055 | RGS6 | Regulator Of G Protein Signaling 6 | 0.24 |
| 4056 | DCHS1 | Dachsous Cadherin-Related 1 | 0.24 |
| 4057 | LILRA2 | Leukocyte Immunoglobulin Like Receptor A2 | 0.24 |
| 4058 | TARBP1 | TAR (HIV-1) RNA Binding Protein 1 | 0.24 |
| 4059 | CUEDC2 | CUE Domain Containing 2 | 0.24 |
| 4060 | AEBP2 | AE Binding Protein 2 | 0.24 |
| 4061 | TDRD1 | Tudor Domain Containing 1 | 0.24 |
| 4062 | POLDIP3 | DNA Polymerase Delta Interacting Protein 3 | 0.24 |
| 4063 | PPM1E | Protein Phosphatase, Mg2+/Mn2+ Dependent 1E | 0.24 |
| 4064 | ZCCHC2 | Zinc Finger CCHC-Type Containing 2 | 0.24 |
| 4065 | MAP7D1 | MAP7 Domain Containing 1 | 0.24 |
| 4066 | C1orf174 | Chromosome 1 Open Reading Frame 174 | 0.24 |
| 4067 | EQTN | Equatorin | 0.24 |
| 4068 | MEIS3P1 | Meis Homeobox 3 Pseudogene 1 | 0.24 |
| 4069 | RPL31P12 | Ribosomal Protein L31 Pseudogene 12 | 0.24 |
| 4070 | GNMT | Glycine N-Methyltransferase | 0.24 |
| 4071 | CAPN10 | Calpain 10 | 0.24 |
| 4072 | C4BPA | Complement Component 4 Binding Protein Alpha | 0.24 |
| 4073 | CHI3L2 | Chitinase 3 Like 2 | 0.24 |
| 4074 | RPL14 | Ribosomal Protein L14 | 0.24 |
| 4075 | DHX15 | DEAH-Box Helicase 15 | 0.24 |
| 4076 | NUPR1 | Nuclear Protein 1, Transcriptional Regulator | 0.24 |
| 4077 | H1-5 | H1.5 Linker Histone, Cluster Member | 0.24 |
| 4078 | MIR302A | MicroRNA 302a | 0.24 |
| 4079 | CADM2 | Cell Adhesion Molecule 2 | 0.23 |
| 4080 | TNFAIP2 | TNF Alpha Induced Protein 2 | 0.23 |
| 4081 | SCG3 | Secretogranin III | 0.23 |
| 4082 | ARF5 | ADP Ribosylation Factor 5 | 0.22 |
| 4083 | PRICKLE2 | Prickle Planar Cell Polarity Protein 2 | 0.22 |
| 4084 | DNAJC7 | DnaJ Heat Shock Protein Family (Hsp40) Member C7 | 0.22 |
| 4085 | AGBL1 | ATP/GTP Binding Protein Like 1 | 0.22 |
| 4086 | TMEM132D | Transmembrane Protein 132D | 0.22 |
| 4087 | H2BC13 | H2B Clustered Histone 13 | 0.22 |
| 4088 | H2BC14 | H2B Clustered Histone 14 | 0.22 |
| 4089 | PSMD14 | Proteasome 26S Subunit, Non-ATPase 14 | 0.22 |
| 4090 | TAOK1 | TAO Kinase 1 | 0.22 |
| 4091 | UVRAG | UV Radiation Resistance Associated | 0.22 |
| 4092 | FKBP8 | FKBP Prolyl Isomerase 8 | 0.22 |
| 4093 | RAD54B | RAD54 Homolog B | 0.22 |
| 4094 | SEC13 | SEC13 Homolog, Nuclear Pore And COPII Coat Complex Component | 0.22 |
| 4095 | SORBS1 | Sorbin And SH3 Domain Containing 1 | 0.22 |
| 4096 | MAPKBP1 | Mitogen-Activated Protein Kinase Binding Protein 1 | 0.22 |
| 4097 | SF3B3 | Splicing Factor 3b Subunit 3 | 0.22 |
| 4098 | DPYSL4 | Dihydropyrimidinase Like 4 | 0.22 |
| 4099 | AKR7A3 | Aldo-Keto Reductase Family 7 Member A3 | 0.22 |
| 4100 | ANKMY1 | Ankyrin Repeat And MYND Domain Containing 1 | 0.22 |
| 4101 | PPIL4 | Peptidylprolyl Isomerase Like 4 | 0.22 |
| 4102 | PSPC1 | Paraspeckle Component 1 | 0.22 |
| 4103 | CHCHD6 | Coiled-Coil-Helix-Coiled-Coil-Helix Domain Containing 6 | 0.22 |
| 4104 | ZRANB1 | Zinc Finger RANBP2-Type Containing 1 | 0.22 |
| 4105 | FXYD4 | FXYD Domain Containing Ion Transport Regulator 4 | 0.22 |
| 4106 | PHACTR2 | Phosphatase And Actin Regulator 2 | 0.22 |
| 4107 | ANKRD13D | Ankyrin Repeat Domain 13D | 0.22 |
| 4108 | MIS18A | MIS18 Kinetochore Protein A | 0.22 |
| 4109 | DNAJC28 | DnaJ Heat Shock Protein Family (Hsp40) Member C28 | 0.22 |
| 4110 | DBF4B | DBF4 Zinc Finger B | 0.22 |
| 4111 | LHFPL2 | LHFPL Tetraspan Subfamily Member 2 | 0.22 |
| 4112 | TSPYL5 | TSPY Like 5 | 0.22 |
| 4113 | PSME3IP1 | Proteasome Activator Subunit 3 Interacting Protein 1 | 0.22 |
| 4114 | SFTA1P | Surfactant Associated 1, LncRNA | 0.22 |
| 4115 | SERPINB9P1 | Serpin Family B Member 9 Pseudogene 1 | 0.22 |
| 4116 | AP2A2 | Adaptor Related Protein Complex 2 Subunit Alpha 2 | 0.22 |
| 4117 | SEPTIN2 | Septin 2 | 0.2 |
| 4118 | IGF2BP3 | Insulin Like Growth Factor 2 MRNA Binding Protein 3 | 0.19 |
| 4119 | GSTM3 | Glutathione S-Transferase Mu 3 | 0.19 |
| 4120 | CTDSPL | CTD Small Phosphatase Like | 0.19 |
| 4121 | ZWINT | ZW10 Interacting Kinetochore Protein | 0.19 |
| 4122 | MELK | Maternal Embryonic Leucine Zipper Kinase | 0.19 |
| 4123 | LONP1 | Lon Peptidase 1, Mitochondrial | 0.19 |
| 4124 | PEA15 | Proliferation And Apoptosis Adaptor Protein 15 | 0.19 |
| 4125 | DDX42 | DEAD-Box Helicase 42 | 0.19 |
| 4126 | NFRKB | Nuclear Factor Related To KappaB Binding Protein | 0.19 |
| 4127 | PNN | Pinin, Desmosome Associated Protein | 0.19 |
| 4128 | ARMH3 | Armadillo Like Helical Domain Containing 3 | 0.19 |
| 4129 | SERPINB8P1 | Serpin Family B Member 8 Pseudogene 1 | 0.19 |
| 4130 | NMS | Neuromedin S | 0.18 |
| 4131 | EIF4A2 | Eukaryotic Translation Initiation Factor 4A2 | 0.17 |
| 4132 | MAPRE2 | Microtubule Associated Protein RP/EB Family Member 2 | 0.17 |
| 4133 | DYNLRB1 | Dynein Light Chain Roadblock-Type 1 | 0.17 |
| 4134 | IGSF8 | Immunoglobulin Superfamily Member 8 | 0.17 |
| 4135 | SCRN1 | Secernin 1 | 0.17 |
| 4136 | ALG10 | ALG10 Alpha-1,2-Glucosyltransferase | 0.17 |
| 4137 | SERPINB3 | Serpin Family B Member 3 | 0.17 |
| 4138 | REG3A | Regenerating Family Member 3 Alpha | 0.17 |
| 4139 | SLC25A3 | Solute Carrier Family 25 Member 3 | 0.15 |
| 4140 | CPT1B | Carnitine Palmitoyltransferase 1B | 0.15 |
| 4141 | HMGCS2 | 3-Hydroxy-3-Methylglutaryl-CoA Synthase 2 | 0.15 |
| 4142 | ADARB1 | Adenosine Deaminase RNA Specific B1 | 0.15 |
| 4143 | BRSK2 | BR Serine/Threonine Kinase 2 | 0.15 |
| 4144 | KMT5A | Lysine Methyltransferase 5A | 0.15 |
| 4145 | RNF123 | Ring Finger Protein 123 | 0.15 |
| 4146 | LINC00461 | Long Intergenic Non-Protein Coding RNA 461 | 0.15 |
| 4147 | PGM1 | Phosphoglucomutase 1 | 0.13 |
| 4148 | PRKAB2 | Protein Kinase AMP-Activated Non-Catalytic Subunit Beta 2 | 0.13 |
| 4149 | RNASEL | Ribonuclease L | 0.13 |
| 4150 | PRKAG3 | Protein Kinase AMP-Activated Non-Catalytic Subunit Gamma 3 | 0.13 |
| 4151 | ALDOC | Aldolase, Fructose-Bisphosphate C | 0.13 |
| 4152 | ERP44 | Endoplasmic Reticulum Protein 44 | 0.13 |
| 4153 | TP53INP1 | Tumor Protein P53 Inducible Nuclear Protein 1 | 0.13 |
| 4154 | SF3A3 | Splicing Factor 3a Subunit 3 | 0.13 |
| 4155 | PCDHB10 | Protocadherin Beta 10 | 0.13 |
| 4156 | LSM12 | LSM12 Homolog | 0.13 |
| 4157 | SEPTIN5 | Septin 5 | 0.13 |
| 4158 | PLAAT4 | Phospholipase A And Acyltransferase 4 | 0.13 |
| 4159 | ANXA2P2 | Annexin A2 Pseudogene 2 | 0.13 |

**References:**

[1]. Xiao-meng SUN, Hai-qing YE, Jing-bo LIU, et al., "Assessment of anti-diabetic activity of peanut shell polyphenol extracts," J Zhejiang Univ Sci B, vol: 19, no: 10, pp. 764-775, 2018.
